# Control and Regulation of Substrate Selection in Cytoplasmic and Mitochondrial Catabolic Networks. A Systems Biology Analysis

**DOI:** 10.3389/fphys.2019.00201

**Published:** 2019-03-08

**Authors:** Sonia Cortassa, Miguel A. Aon, Steven J. Sollott

**Affiliations:** Laboratory of Cardiovascular Science, National Institute on Aging, National Institutes of Health, Baltimore, MD, United States

**Keywords:** glucose and fatty acids, computational modeling, metabolic control analysis, central catabolism, pyruvate dehydrogenase regulation, control coefficients

## Abstract

Appropriate substrate selection between fats and glucose is associated with the success of interventions that maintain health such as exercise or caloric restriction, or with the severity of diseases such as diabetes or other metabolic disorders. Although the interaction and mutual inhibition between glucose and fatty-acids (FAs) catabolism has been studied for decades, a quantitative and integrated understanding of the control and regulation of substrate selection through central catabolic pathways is lacking. We addressed this gap here using a computational model representing cardiomyocyte catabolism encompassing glucose (Glc) utilization, pyruvate transport into mitochondria and oxidation in the tricarboxylic acid (TCA) cycle, β-oxidation of palmitate (Palm), oxidative phosphorylation, ion transport, pH regulation, and ROS generation and scavenging in cytoplasmic and mitochondrial compartments. The model is described by 82 differential equations and 119 enzymatic, electron transport and substrate transport reactions accounting for regulatory mechanisms and key players, namely pyruvate dehydrogenase (PDH) and its modulation by multiple effectors. We applied metabolic control analysis to the network operating with various Glc to Palm ratios. The flux and metabolites’ concentration control were visualized through heat maps providing major insights into main control and regulatory nodes throughout the catabolic network. Metabolic pathways located in different compartments were found to reciprocally control each other. For example, glucose uptake and the ATP demand exert control on most processes in catabolism while TCA cycle activities and membrane-associated energy transduction reactions exerted control on mitochondrial processes namely β-oxidation. PFK and PDH, two highly regulated enzymes, exhibit opposite behavior from a control perspective. While PFK activity was a main rate-controlling step affecting the whole network, PDH played the role of a major regulator showing high sensitivity (elasticity) to substrate availability and key activators/inhibitors, a trait expected from a flexible substrate selector strategically located in the metabolic network. PDH regulated the rate of Glc and Palm consumption, consistent with its high sensitivity toward AcCoA, CoA, and NADH. Overall, these results indicate that the control of catabolism is highly distributed across the metabolic network suggesting that fuel selection between FAs and Glc goes well beyond the mechanisms traditionally postulated to explain the glucose-fatty-acid cycle.

## Introduction

The profile of substrates consumption has been the focus of great interest in medical research due to their associations with diverse health conditions, such as metabolic disorders, diabetes, heart failure and cancer that display altered patterns of glucose and fats utilization. The interplay between fats and glucose utilization has been intensely studied in the context of fed-fast transitions, caloric restriction, starvation and insulin insensitivity in metabolic disorders (McGarry and Foster, 1980; Randle, 1998; Soeters et al., 2012; Perry et al., 2018). Organs in the human body behave in specific ways with respect to substrates that fuel their function (e.g., glucose, fats, ketone bodies, amino acids) (Cahill, 2006; Puchalska and Crawford, 2017). Cardiomyocytes obtain most of their energy from fats, although, a balance in the supply between glucose and fats should be maintained to sustain healthy heart function (Aon et al., 2015; Roul and Recchia, 2015; Sung et al., 2015). On the other hand, skeletal muscle will use preferentially glucose from glycogen stores, however, the extent and the pathway (either oxidative or homolactic fermentation) depends upon fiber type (Egan and Zierath, 2013; Aon et al., 2014).

Glucose and FAs are metabolized through central catabolic pathways, a universal biochemical backbone from prokaryotes to eukaryotes. Glucose and FAs catabolism converges at the level of AcCoA, which can be generated via pyruvate dehydrogenase from glucose-derived pyruvate, or from mitochondrial β-oxidation. Central catabolism provides all the precursors (sugars, lipids, amino acids) for cellular biomass (Cortassa et al., 2012), as well as the donors of specific posttranslational modifications such as acetylation, methylation, redox, phosphorylation (Guan and Xiong, 2011; Foster et al., 2013; Aon et al., 2016). Many important sensors of metabolic status at the origin of signaling pathways (e.g., AMPK, mTOR) (Hardie et al., 2016; Saxton and Sabatini, 2017; Lin and Hardie, 2018) or posttranslational modifications (PTMs) correspond to intermediates of energy metabolism (Rossetti and Giaccari, 1990; Williams and Elmquist, 2012; Mitacchione et al., 2014; Kumar Jha et al., 2015; Stark et al., 2015; Baeza et al., 2016; Parodi-Rullan et al., 2017). However, an integrative and quantitative approach to study systemically the control and regulation of central catabolism, important for addressing modulation of fuel selection (e.g., glucose and fats) under substrate excess occurring in insulin resistance or overfeeding, has not been developed so far.

Metabolic control analysis (MCA) has been mainly used in biotechnological studies aimed at rationally optimizing processes leading to commercially valuable products, or eliminating undesirable chemicals, e.g., from the environment (Cortassa et al., 2012). In fundamental research, it has been utilized to study control in isolated pathways (e.g., glycolysis, oxidative phosphorylation (OxPhos), tryptophan metabolism (Groen et al., 1982; Tager et al., 1983; Heinrich, 1985; Niederberger et al., 1992; Kashiwaya et al., 1994; Aon and Cortassa, 1998; Heinrich and Schuster, 1998). However, there is great but untapped potential in MCA for producing valuable quantitative knowledge about metabolic behavior such as substrate fuel selection, a highly relevant subject for metabolic disorders such as obesity and diabetes where glucose and fats are in excess, the functional energy deficits in certain forms of heart failure (Lopaschuk et al., 2010; Doenst et al., 2013; Peterzan et al., 2017) or for lifespan-extending and health-span-improving strategies such as caloric restriction (Mitchell et al., 2016, 2018) or time-restricted feeding (Panda, 2016; Mattson et al., 2017; Marosi et al., 2018) to name just a few. MCA can help identify control and regulatory steps in a metabolic network, pointing out targets amenable to pharmacological, genetic or interventional (diet, exercise) strategies, leading to disease prevention, health promotion, or healthy aging.

Metabolic control quantifies through flux and metabolite concentration control coefficients to what extent a certain flux through a metabolic pathway varies upon changes in enzymatic and/or transport activity (i.e., via up- or down-modulation of protein expression, PTMs or regulatory feedback). To distinguish control from regulation has practical implications because it provides crucial clues about strategies to modulate metabolism (Fell, 1992; Cortassa et al., 2009a). On the one hand, a failure in an enzymatic regulatory step will not be solved by increasing the protein expression but by modulating the effectors. On the other hand, a desirable increase or decrease in flux through an enzymatic or transport process will not be improved by changing the level of effectors but by affecting the increase or decrease in protein level of enzyme or transporter, respectively, using promoting or interfering strategies of gene expression or PTMs leading to the desired effect.

In the present work, we apply an integrative and quantitative approach to study the control and regulation of substrate selection in a computational model of central catabolism comprising the utilization and processing of glucose and FAs in cytoplasmic and mitochondrial compartments. This strategy is amenable for systems biology approaches integrating comprehensive –“omics” data (e.g., metabolomics and fluxomics) combined with computational modeling and bioinformatics (Cortassa et al., 2018a). The present computational model includes a novel step per step description of glycolysis, glycogenolysis, polyol and pentose phosphate pathways, and mitochondrial β-oxidation, TCA cycle together with ionic and redox-energy transducing processes, namely respiratory electron transport, ATP synthesis, and ROS generation and ROS scavenging in mitochondrial and cytoplasmic compartments. This is the first time that a validated model of central catabolism of this magnitude, including many known regulatory mechanisms, has been developed, and a systemic quantitative analysis of control and regulation of substrate selection at different ratios of glucose and FA attempted. Although the model utilized has been optimized and parameterized for cardiac muscle cells, it can be readily adapted to other cell types and tissues.

## Materials and Methods

**FIGURE 1:**
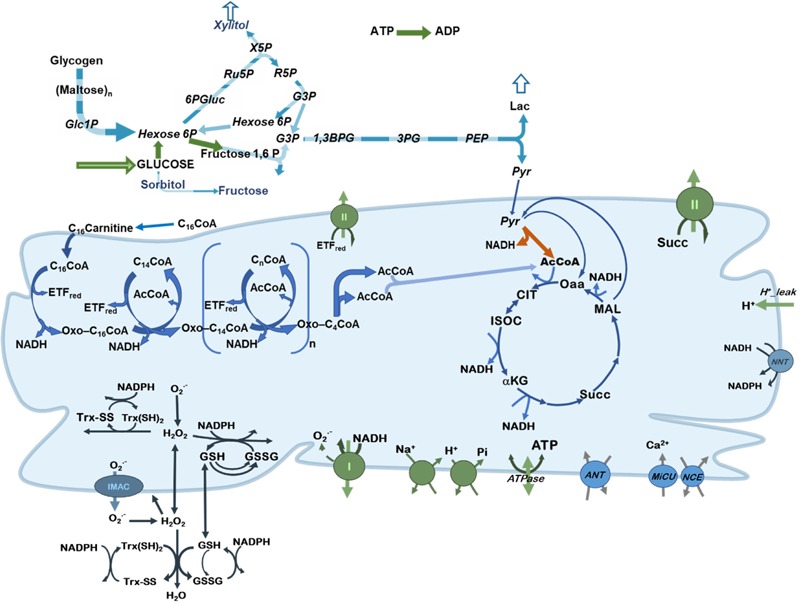
Scheme of central catabolism fueled by glucose and fatty acids oxidation pathways. Depicted are the processes included in the model of central catabolism from glucose and FAs (PCoA), in cytoplasmic and mitochondrial compartments. Highlighted in green, are the processes that share a relatively large control on network fluxes and substrate selection. Pyruvate dehydrogenase (PDH) is indicated with an orange arrow to highlight its regulatory role.

To study the control and regulation of substrate selection in central catabolism fueled by different combinations of Glc and the FA Palm, a computational model was formulated encompassing main catabolic pathways involved in the utilization of glucose and palmitoylCoA (PCoA), the activated form of Palm (Figure 1).

The computational model is, in part, based on previous work where modeling and validation of FA oxidation (Cortassa et al., 2017a,b), glucose catabolism (Cortassa et al., 2015) under normal or pathological conditions (Cortassa et al., 2018a) was performed. The model encompasses glucose transport, glycolysis, glycogenolysis, pentose phosphate and polyol pathways. In the model, pyruvate generated by glycolysis can be converted to lactate or transported into mitochondria where it will be subjected to either dehydrogenation by PDH providing acetylCoA (AcCoA) or carboxylation to supply oxaloacetate anaplerotically to the TCA cycle. Fatty acids catabolism is started from PCoA transport into mitochondria and degraded via β-oxidation to generate AcCoA, NADH and FADH_2_ in the prosthetic group from Electron Transfer Flavoprotein (ETF). AcCoA will be further degraded in the TCA cycle generating NADH and succinate that supply electrons to the respiratory chain. ATP synthesis, ion transport (Ca^2+^, Pi, Na^+^, and H^+^) (Wei et al., 2011), H^+^ leak currents, ROS generation in the respiratory chain and scavenging both in mitochondrial matrix and cytoplasm (Kembro et al., 2013) are all included in the model formulation. A previously published version of the computational model encompassed fatty acids and glucose oxidation pathways in a more aggregated or lumped form (Cortassa et al., 2018a).

The model comprises 82 state variables and 119 enzymatic, electron transport and substrate and ion transport reactions. Model simulations were run with a code written in Matlab (The Mathworks, Natick, MA, United States) using the ODEs15 integrator. MCA was performed using a program written in Matlab and its algebraic toolbox.

A description of the model in terms of ordinary differential equations (ODEs), rate equations describing the kinetics of each of the processes included in the metabolic network depicted in Figure 1, as well as parameters, are listed in Supplementary Tables S1–S16. Results reported correspond to steady state behavior, when the relative time derivative of each variable is <1 × 10^-10^ sec^-1^.

## Results

### Steady State Behavior of Central Catabolism Fueled by Glucose and Palmitate

**FIGURE 2:**
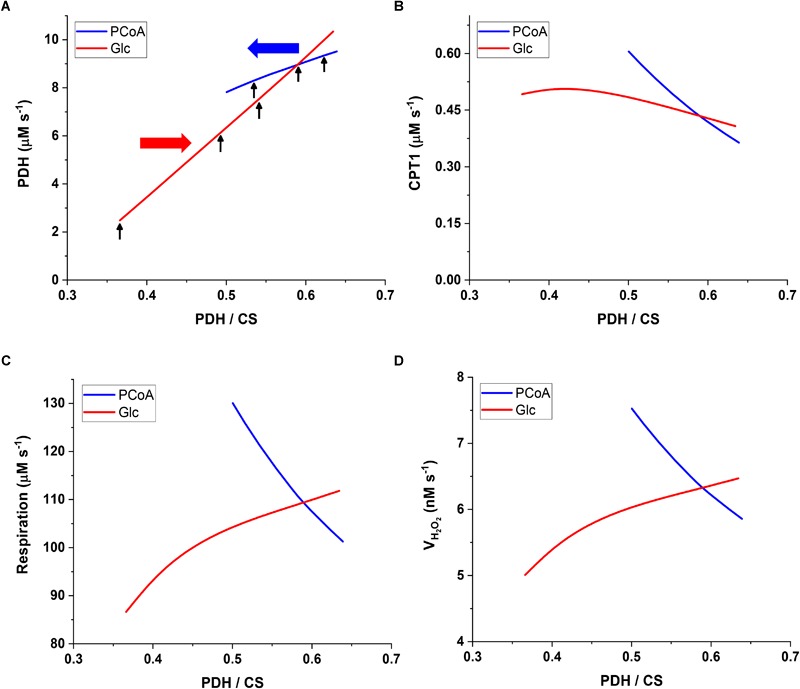
Interacting mitochondrial metabolic fluxes from glucose (Glc) and palmitoyl CoA (PCoA) catabolism. The plots show the flux through **(A)** pyruvate dehydrogenase (PDH), **(B)** carnitine palmitoyl transferase I (CPT1), **(C)** respiratory electron transport [respiration] and **(D)** mitochondrial H_2_O_2_ emission (V_H2O2em_) as a function of the ratio of PDH over CS fluxes that quantifies the relative amount of the TCA cycle substrate AcCoA derived from pyruvate and β-oxidation, respectively. The value of PDH/CS is determined by the range of PCoA and Glc concentrations utilized. PCoA was varied from 0.5 to 60 μM at constant 10mM Glc (blue line). The red line corresponds to the change in Glc from 5 to 11 mM at constant 10 μM PCoA. Respiration encompasses the sum of the NADH from TCA cycle and β-oxidation plus the succinate dehydrogenase (SDH)- and the electron transfer protein (ETF)-associated electron transport from β-oxidation **(C)**. Each of the electron fluxes is associated to the generation of ROS that is either scavenged by the antioxidant systems in the mitochondrial matrix or exits the mitochondria as H_2_O_2_ **(D)**. The red and blue horizontal arrows in **(A)** represent the sense of exogenous substrate increase, Glc or PCoA, respectively. The vertical black arrows in **(A)** indicate the PDH/CS ratios at which MCA was performed: 10 μM PCoA with Glc variable at 5, 8, 9, or 10 mM, or 10 mM Glc with PCoA variable at 1, 10, or 40 μM.

To characterize the computational model steady state behavior, we performed simulations with either increasing glucose concentration in the extracellular compartment at fixed 10 μM PCoA or augmenting PCoA concentration at constant 10 mM Glc. Figure 2 displays the results of the simulations for each condition.

The flux ratio of PDH over citrate synthase (CS) (PDH/CS) quantifies the relative amount of mitochondrial energy derived from Glc with respect to FA oxidation (Figure 2A,B). AcCoA is at the convergence of glucose and FAs oxidation (Figure 1). Since it is produced by PDH when derived from carbohydrate oxidation, and is consumed by CS in the TCA cycle, the ratio PDH/CS represents which fraction of the carbon directed to mitochondrial OxPhos is derived from Glc *vs*. FAs. For example, a 0.5 PDH/CS flux ratio indicates that half of the AcCoA derives from PCoA oxidation in mitochondria although the flux sustained by PDH is several-fold larger than the FA uptake via carnitine palmitoyl transferase 1 (CPT1) (Figure 2A,B). The latter can be explained by the fact that 8 AcCoA are contributed by PCoA degradation via β-oxidation, and by a “dilution” term corresponding to the CPT1 flux expressed in cytoplasmic terms, (i.e., a 4-fold larger cytoplasmic than mitochondrial volume, thus its flux will be “concentrated” 4-fold upon entering mitochondria). The magnitude of the PDH/CS ratio varied between ∼0.35 and ∼0.65, a range similar to what has been reported (Perry et al., 2018) for skeletal muscle in the fed state, at least for the steady state obtained at 10 mM Glc, and very low PCoA (0.5 μM). This is also consistent with the fact that FAs are main fuels in the heart mainly at rest, and in skeletal muscle, to a certain extent.

The flux through PDH varies more than 5-fold when extracellular Glc increases from 5 to 11 mM at constant 10 μM PCoA (Figure 2A, red line). On the other hand, clamping Glc at 10 mM and varying PCoA from 0.5 to 60 μM, result in less than 20% change in PDH flux (Figure 2A, blue line). The increase in PCoA diminishes the carbohydrate contribution to the TCA cycle flux from PDH/CS = 0.65 to 0.5. These trends confirm the function of the glucose-fatty acid cycle, i.e., defined as the interaction between the pathways degrading both substrates, while adding a quantitative dimension to the effects originally described qualitatively more than 50 years ago (Randle et al., 1963). On the other hand, the flux through CPT1 augments, as expected, at increasing levels of PCoA (Figure 2B). Interestingly, also the variation of Glc at fixed PCoA leads to changes in CPT1 activity decreasing from 0.5 to 0.4 μM s^-1^ (Figure 2B). Surprisingly, at low 5 to 6 mM Glc levels, the flux sustained by CPT1 is enhanced by Glc, which can be explained by the anaplerotic role of pyruvate carboxylase, required for faster TCA cycling.

Regarding global bioenergetic behavior, simulations show that respiration is enhanced concomitant with H_2_O_2_ emission from mitochondria under both conditions, i.e., augmenting Glc or PCoA (Figure 2C,D). However, the largest percentage increase in respiratory flux is attained at low glucose, again due to the increased replenishment of TCA cycle intermediates, enabling a faster cycling flux thus more rapid delivery of NADH to the respiratory chain. As expected, due to the abundant supply of redox equivalents both as NADH and ETFH_2_, the levels of respiration are higher with PCoA than Glc. As a caveat, the activated FA, PCoA, exerts a significant uncoupling effect, first studied when β-oxidation was introduced into our modeling scheme (Cortassa et al., 2017a).

Overall, the PDH/CS ratio, as a metric of substrate selection, quantifies the relative contribution of glucose and FA catabolism to the AcCoA pool that feeds the TCA cycle, at changing relative levels between both substrates (Glc and PCoA). Under these conditions, we found that substrate selection is more sensitive to glucose than PCoA variation, and although the changes in substrate levels reciprocally affect each other, both substrates, to a greater or lesser extent, feed mitochondrial OxPhos. In the next section, we analyze the main rate-controlling steps of the flux through central catabolic pathways of Glc and PCoA.

### Flux Control of Substrate Selection When Both Glc and PCoA Fuel Cell Function

We calculated the overall flux control throughout central catabolism at different steady states of the computational model, obtained in simulations depicted in Figure 2.

**FIGURE 3:**
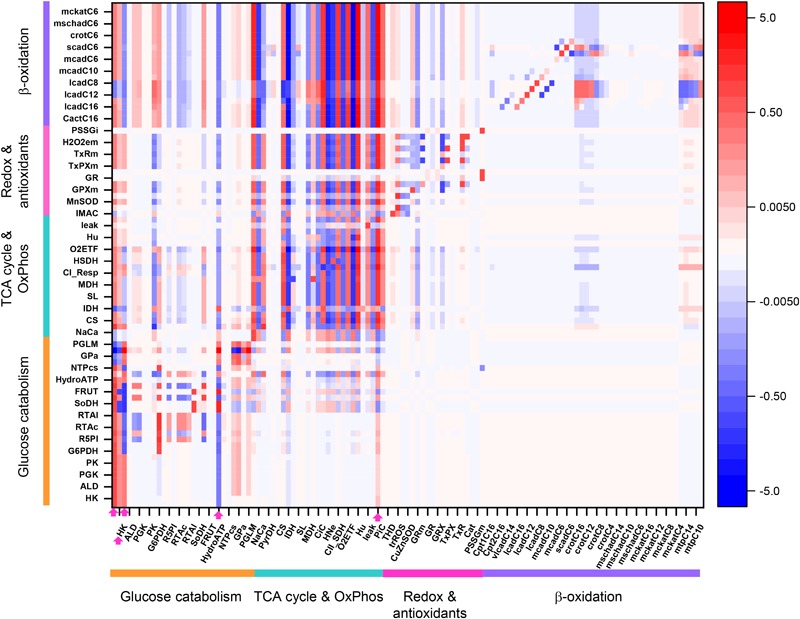
Overview heat map of flux control coefficients in the catabolic network fueled by Glc and PCoA. The values of the flux control coefficients exerted by individual enzymes/transport processes over metabolic reaction steps in the catabolic network present in the *x*- and *y*-axes, respectively, can be positive (red) or negative (blue) as depicted by the color scale on the right. Only steps for which the sum of the control coefficients falls within the range 0.5 – 2.0 are included (i.e., the summation theorem of MCA states that the sum of flux control coefficients should equal 1.0). The key to the fluxes and activities is indicated in Table 1. For visualization, the block of cytoplasmic (mainly glucose catabolism), mitochondrial (TCA cycle and Oxphos), redox and antioxidant reactions, and lipid oxidation, are indicated by colored bars. The order of the reactions will be the same for all heat maps presented in this work. Although, for clarity, only one out of two reaction labels are displayed in the *x*-axis, the control coefficients with respect to each one of the activities participating in the model are shown in the heat map. The heat map displayed was obtained from the MCA applied to the steady state simulation at 10 mM Glc and 10 μM PCoA (i.e., convergence point between Glc or PCoA variable condition shown in Figure 2A). Magenta arrows point to main rate-controlling steps of the flux. The numerical values of the flux control coefficients are shown in Supplementary Table S21.

Figure 3 shows an overview heat map of the profile of flux control coefficients obtained under 10 mM Glc and 10 μM PCoA that represents a crossing point between both substrates when changing their concentrations with respect to one another, while one of them is kept constant (Figure 2A,B). On the *x*-axis are the activities that control the flux through the processes on the *y*-axis (i.e., transport, enzymes, electrochemical gradient). Importantly, the red-blue columns at the extreme left of the heat map display the large and highly distributed control exerted throughout the catabolic network by glucose transport (Glut4), hexokinase (HK), and phosphofructokinase (PFK) steps from glycolysis (for clarity only HK is specified on the *x*-axis although the three activities are included) (Figure 3, magenta arrows on *x*-axis). Further downstream, the rate of cytoplasmic ATP hydrolysis (HydroATP), representing all energy-demand processes in the cytoplasmic compartment (e.g., myocytes’ contraction, ion transport, energy buffering reactions, such as creatine kinase and adenylate kinase), and the mitochondrial phosphate carrier (PiC), also exert a widespread control throughout the catabolic network (Figure 3, magenta arrows on *x*-axis).

**Table 1:** Abbreviations used in plots and heatmaps.

Acronym	Full name
GLUT4	Glucose transport
HK	Hexokinase
PFK	Phosphofructokinase
ALD	Aldolase
GAPD	Glyceraldehyde 3 phosphate dehydrogenase
PGK	Phosphoglycerate kinase
ENOL	Enolase
PK	Pyruvate kinase
LDH	Lactate dehydrogenase
G6PDH	Glucose 6 phosphate dehydrogenase
P6GDH	6 phosphogluconate dehydrogenase
R5PI	Ribose 5 phosphate isomerase
Ru5PE	Ribulose 5 phosphate Epimerase
RTAc	Transketolase I
RTAc2	Transketolase 2
RTAl	Transaldolase
ALDR1	Aldose reductase
SoDH	Sorbitol dehydrogenase
XyDH	Xylitol dehydrogenase
FRUT	Fructose transport
XyOHT	Xylitol transport
HydroATP	Cytoplasmic ATP hydrolysis
NDPcs	NADH consumption
NTPcs	NADPH consumption
Gno_is	Glycogen debranching enzyme
GPa	Glycogen phosphorylase a
GPb	Glycogen phosphorylase b
PGLM	Phosphoglucomutase
Uni	Mitochondrial Ca^2+^ uniporter
NaCa	Na^+^ Ca^2+^ exchanger
PyrC	Pyruvate Carrier
PyrDH	Pyruvate dehydrogenase
PyrCb	Pyruvate carboxylase
CS	Citrate synthase
ACO	Aconitase
IDH	Isocitrate dehydrogenase (NAD^+^ dependent)
KGDH	α-ketoglutarate dehydrogenase
SL	Succinate lyase
FH	Fumarate hydratase
MDH	Malate dehydrogenase
AAT	Aspartate aminotransferase
CiC	Citrate carrier
CI_Resp	Complex I linked electron transport
HNe	H^+^ transport linked to Complex I-III-IV
HSDH	H^+^ transport linked to Complex II-III-IV
CII_SDH	Succinate dehydrogenase electron transport
VETFH	ETF-linked proton transport
O2ETF	ETF-linked electron transport
ATPsynthase	ATP synthase
Hu	H^+^ transport linked to ATP synthase
ANT	Adenine nucleotide translocator
leak	H^+^ leak
NaH	Na^+^ H^+^ exchanger
PiC	Phosphate carrier
IDH_NADP	Isocitrate dehydrogenase (NADP^+^ dependent)
THD	Transhydrogenase
IMAC	Inner membrane anion channel
trROS	ROS transport linked to IMAC
MnSOD	Mn superoxide dismutase (mitochondrial)
CuZnSOD	CuZn superoxide dismutase (cytoplasmic)
GPXm	Glutathione peroxidase (mitochondrial)
GRm	Glutathione reductase (mitochondrial)
GPX	Glutathione peroxidase (cytoplasmic)
GR	Glutathione reductase (cytoplasmic)
GRXm	Glutaredoxin (mitochondrial)
GRX	Glutaredoxin (cytoplasmic)
TxPXm	Thioredoxin peroxidase (mitochondrial)
TxPX	Thioredoxin peroxidase (cytoplasmic)
TxRm	Thioredoxin reductase (mitochondrial)
TxR	Thioredoxin reductase (cytoplasmic)
H2O2em	H2O2 emission from mitochondria
Cat	Catalase
GST	Glutathione transport
PSSGm	Protein glutathionylation (mitochondrial)
PSSGi	Protein glutathionylation (cytoplasmic)
Cpt1C16	Carnitine palmitoyl transferase I
CactC16	Carnitine palmitoyl carnitine translocase
Cpt2C16	Carnitine palmitoyl transferase II
vlcadC16	Very long fatty acylCoA dehydrogenase
vlcadC14	Very long fatty acylCoA dehydrogenase
vlcadC12	Very long fatty acylCoA dehydrogenase
lcadC16	Long fatty acylCoA dehydrogenase
lcadC14	Long fatty acylCoA dehydrogenase
lcadC12	Long fatty acylCoA dehydrogenase
lcadC10	Long fatty acylCoA dehydrogenase
lcadC8	Long fatty acylCoA dehydrogenase
mcadC12	Medium fatty acylCoA dehydrogenase
mcadC10	Medium fatty acylCoA dehydrogenase
mcadC8	Medium fatty acylCoA dehydrogenase
mcadC6	Medium fatty acylCoA dehydrogenase
mcadC4	Medium fatty acylCoA dehydrogenase
scadC6	Short fatty acylCoA dehydrogenase
scadC4	Short fatty acylCoA dehydrogenase
crotC16	Fatty acid enoyl-CoA hydratase (Crotonase)
crotC14	Fatty acid enoyl-CoA hydratase (Crotonase)
crotC12	Fatty acid enoyl-CoA hydratase (Crotonase)
crotC10	Fatty acid enoyl-CoA hydratase (Crotonase)
crotC8	Fatty acid enoyl-CoA hydratase (Crotonase)
crotC6	Fatty acid enoyl-CoA hydratase (Crotonase)
crotC4	Fatty acid enoyl-CoA hydratase (Crotonase)
mschadC16	Medium/short chain hydroxyacylCoA dehydrogenase
mschadC14	Medium/short chain hydroxyacylCoA dehydrogenase
mschadC12	Medium/short chain hydroxyacylCoA dehydrogenase
mschadC10	Medium/short chain hydroxyacylCoA dehydrogenase
mschadC8	Medium/short chain hydroxyacylCoA dehydrogenase
mschadC6	Medium/short chain hydroxyacylCoA dehydrogenase
mschadC4	Medium/short chain hydroxyacylCoA dehydrogenase
mckatC16	Medium-chain ketoacylCoA thiolase
mckatC14	Medium-chain ketoacylCoA thiolase
mckatC12	Medium-chain ketoacylCoA thiolase
mckatC10	Medium-chain ketoacylCoA thiolase
mckatC8	Medium-chain ketoacylCoA thiolase
mckatC6	Medium-chain ketoacylCoA thiolase
mckatC4	Medium-chain ketoacylCoA thiolase
mtpC16	Mitochondrial trifunctional protein
mtpC14	Mitochondrial trifunctional protein
mtpC12	Mitochondrial trifunctional protein
mtpC10	Mitochondrial trifunctional protein
mtpC8	Mitochondrial trifunctional protein
Glci	Intracellular glucose
H6P	Glucose 6 phosphate + fructose 6 phosphate
FbP	Fructose 1,6 bisphosphate
G3P	Glyceraldehyde 3 phosphate
BPG	1,3 biphosphoglycerate
3PG	3 phosphoglycerate
PEP	Phosphoenolpyruvate
Pyr	Pyruvate (cytoplasm)
Sor	Sorbitol
Fru	Fructose
6PG	6 phosphogluconate dehydrogenase
Ru5P	Ribulose 5 phosphate
R5P	Ribose 5 phosphate
X5P	Xylulose 5 phosphate
E4P	Erythrose 4 phosphate
S7P	Sedoheptulose 7 phosphate
Mal	Maltosides
G1P	Glucose 1 phosphate
Pyr_m_	Mitochondrial pyruvate
Dpsim	Mitochondrial membrane potential
ISOC	Isocitrate
αKG	α-ketoglutarate
SCoA	SuccinylCoA
Suc	Succinate
Fum	Fumarate
MAL	Malate
OAA	Oxalacetate
CIT	Citrate
C16Carn_i_	Palmitoyl carnitine (cytoplasmic)
C16Carn_m_	Palmitoyl carnitine (mitochondrial)
C16CoA_m_	Mitochondrial PalmitoylCoA
C16enoylCoA	Palmitoyl enoylCoA
C16OHCoA	HydroxypalmitoylCoA
C16ketoCoA	KetopalmitoylCoA
C14CoA	MyristoylCoA
C14enoylCoA	Myristoyl enoylCoA
C14OHCoA	HydroxymyristoylCoA
C14ketoCoA	KetomyristoylCoA
C12CoA	LauroylCoA
C12enoylCoA	Lauroyl enoylCoA
C12OHCoA	HydroxylauroylCoA
C12ketoCoA	KetolauroylCoA
C10CoA	DecanoylCoA
C10enoylCoA	Decanoyl enoylCoA
C10OHCoA	HydroxydecanoylCoA
C10ketoCoA	KetodecanoylCoA
C8CoA	DecanoylCoA
C8enoylCoA	Decanoyl enoylCoA
C8OHCoA	HydroxyoctanoylCoA
C8ketoCoA	KetooctanoylCoA
C6CoA	HexanoylCoA
C6enoylCoA	Hexanoylenoyl CoA
C6OHCoA	HydroxyhexanoylCoA
C6ketoCoA	KetohexanoylCoA
C4CoA	ButanoylCoA
C4enoylCoA	Butanoyl enoylCoA
C4OHCoA	HydroxybutanoylCoA
C4ketoCoA	KetobutanoylCoA
AcCoA	AcetylCoA
SO2m	Mitochondrial superoxide anion
SO2i	Cytoplasmic superoxide anion
H2O2m	Mitochondrial hydrogen peroxide
H2O2i	Cytoplasmic hydrogen peroxide
GSHm	Mitochondrial reduced glutathione
GSHi	Cytoplasmic reduced glutathione
GSSGm	Mitochondrial oxidized glutathione
GSSGi	Cytoplasmic oxidized glutathione
TxRm	Mitochondrial thioredoxin
TxRi	Cytoplasmic thioredoxin
PSSGm	Mitochondrial glutathionylated protein
PSSGi	Cytoplasmic glutathionylated protein

Interestingly, “redox and antioxidants” processes involved in redox (e.g., transhydrogenase, THD, a mitochondrial NADPH replenishing enzyme, NADP^+^-dependent isocitrate dehydrogenase, IDH) and antioxidant metabolism [e.g., glutathione (GR)- and thioredoxin (TxR)- reductases] cluster together, indicating that they mainly exert control over themselves while negligibly controlling other pathways in the network. Activities operating inside mitochondria, e.g., aconitase (ACO), IDH NAD^+^- dependent, malate dehydrogenase (MDH), aspartate-amino transferase (AAT) and membrane-associated processes (respiratory electron transport, ATP synthase and ADP- and phosphate-transport) all exert control on the TCA cycle and OxPhos along with other mitochondrial matrix processes such as β-oxidation and antioxidant reaction fluxes. Reaction steps from FA oxidation mainly control fluxes through β-oxidation and exert some quantifiable control over TCA cycle and OxPhos.

**FIGURE 4:**
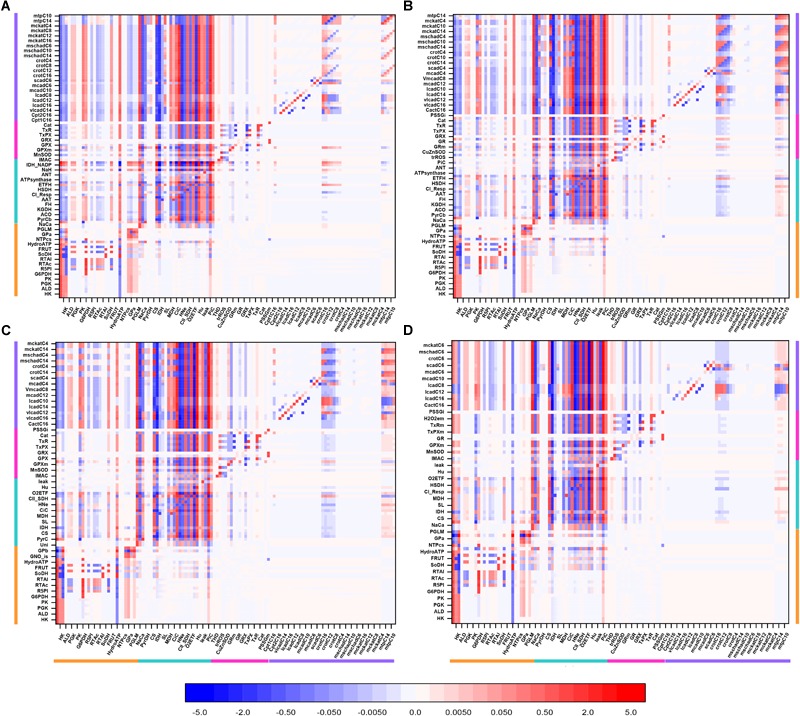
Overview heat maps of flux control coefficients in the catabolic network as function of increasing Glc concentration at constant 10 μM PCoA. Flux control coefficients obtained by MCA of the steady states represented in Figure 2 are displayed. The conditions for these simulations were constant 10 μM PCoA, and variable glucose in the extracellular medium (in mM): **(A)** 5; **(B)** 8; **(C)** 9; **(D)** 10. The red-blue scale represents the magnitude of the flux control coefficients, and is the same for all panels, including that shown in Figure 3. The key to the processes is the same as the one in Figure 3, the heat map of which is also included in this figure for the sake of comparison with lower Glc concentrations. The numerical values of the flux control coefficients are shown in Supplementary Tables S18–S21.

**FIGURE 5:**
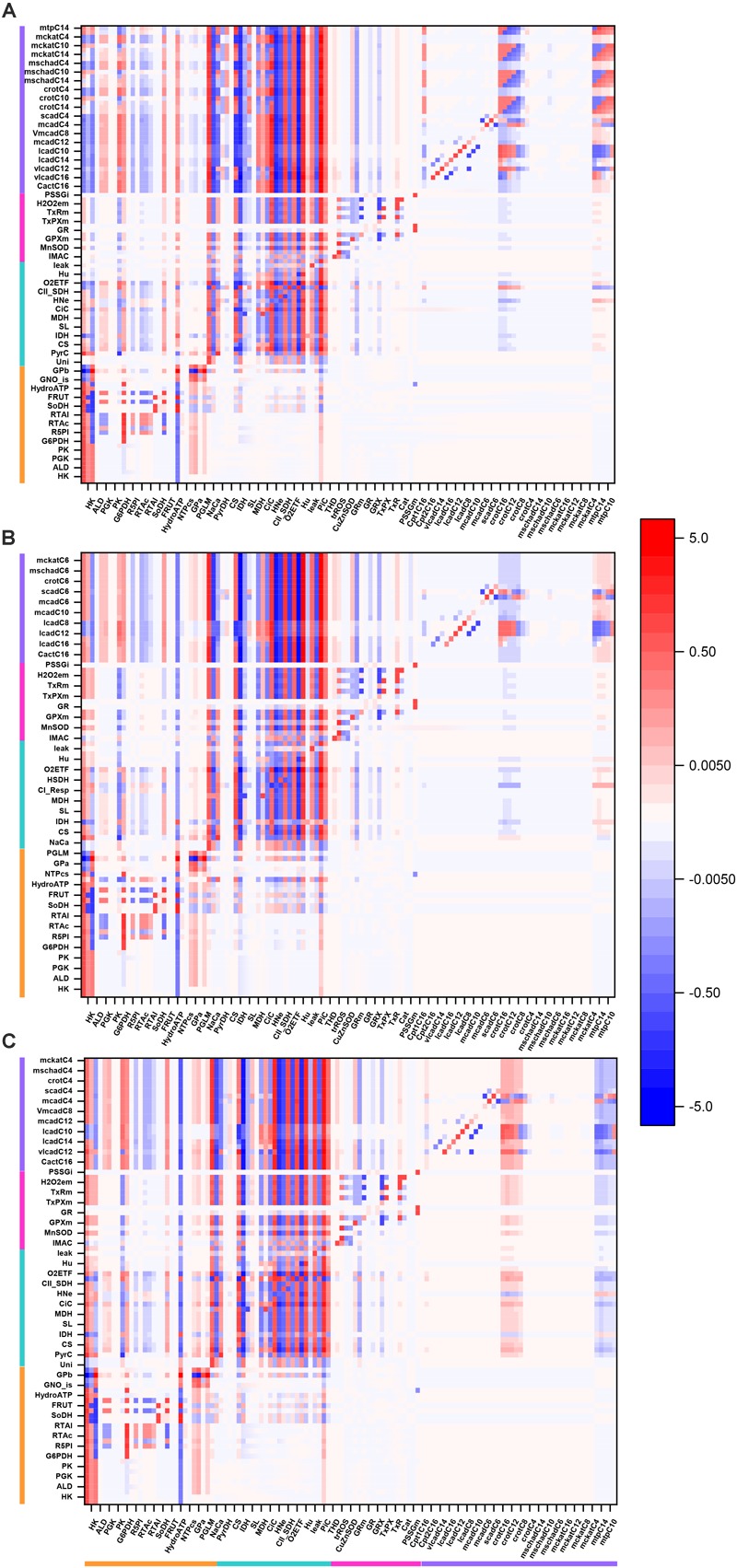
Overview heat maps of flux control coefficients in the catabolic network as function of increasing PCoA concentration at constant 10 mM Glc. Flux control coefficients obtained by MCA of the steady states represented in Figure 2 are displayed. The conditions for these simulations were 10 mM Glc, and variable PCoA (in μM): **(A)** 1; **(B)** 10; **(C)** 40. The red-blue scale represents the magnitude of the flux control coefficients, and is the same for all panels, including that shown in Figure 3. The key to the processes is the same as the one shown in Figure 3, the heat map of which is also included in this figure (**B**: 10 μM PCoA – 10 mM Glc) for the sake of comparison with lower or higher PCoA concentrations. The numerical values of the flux control coefficients are shown in Supplementary Tables S21–S23.

Qualitatively, the same general pattern of control was corroborated at various Glc levels, although the magnitude and sign of the control coefficients changed with Glc concentration (Figure 4). As an example, the negative control exerted by Glc uptake on the fluxes through the TCA cycle and OxPhos at 5- and 8-mM glucose (Figure 4A,B) becomes positive when Glc increases to 10 mM (Figure 4D). Similarly, the control by cytoplasmic ATP demand on mitochondrial processes is positive at low Glc but becomes negative at 10 mM Glc. When glucose is held constant but PCoA is increased 40-fold the strength of control changes quantitatively while, qualitatively, keeps the same pattern of control, i.e., similar rate-controlling steps over the same target fluxes (Figure 5).

Together, the data show that when either Glc or PCoA is held constant as the other substrate is varied, qualitatively, a similar pattern of control happens although, quantitatively, the extent of control exerted by the main rate-controlling steps changes.

### Differences Between Rate-Controlling and Regulatory Steps of the Flux Under Glc and PCoA Catabolism

The differences between “control” and “regulation” are nicely exemplified by PDH and PFK, two enzymes with many physiological modulators of their activity yet displaying opposite behavior with respect to the extent of their control and regulatory properties. Specifically, under the conditions explored in this work (Figure 2–5), compatible with the behavior of healthy mouse hearts, the upstream glycolytic enzyme PFK, modulated by AMP, ATP and citrate (see Supplementary Material, rate expression in equation S85), displays low sensitivity (“elasticity” in terms of MCA) toward its effectors but exerts relatively high flux control. Oppositely, at the “cross-road” of substrate selection, the mitochondrial PDH complex, modulated by Ca^2+^, pyruvate, NADH, NAD, ATP, ADP, AcCoA and CoA, displays high sensitivity (elasticity) notably to AcCoA and CoA (Cortassa et al., 2017b).

Under all conditions analyzed, PFK exerts relatively high flux control but with low elasticity, while PDH displays high elasticity but low flux control. The high elasticity displayed by PDH is consistent with both flexibility, as expected from its key role in substrate selection, and lack of flux control under any of the conditions explored herein. Beyond their different positioning in the catabolic network, the different elasticities exhibited by PFK and PDH explain their distinct abilities to control or regulate the flux, respectively.

### Sharing of Flux Control Through Pyruvate Kinase (Glycolysis) and CPT1 (β-Oxidation) in Cytoplasmic and Mitochondrial Compartments Under Different Relative Levels of Glc and PCoA

Next, we determined the distribution of control and the main rate-controlling steps throughout the catabolic network. The fluxes through pyruvate kinase (PK) from the lower part of glycolysis, and CPT1 from β-oxidation, that catalyzes the transfer of palmitoyl carnitine from the cytoplasm to the mitochondrial matrix, were studied as representative steps from Glc and FA oxidation pathways, respectively.

**FIGURE 6:**
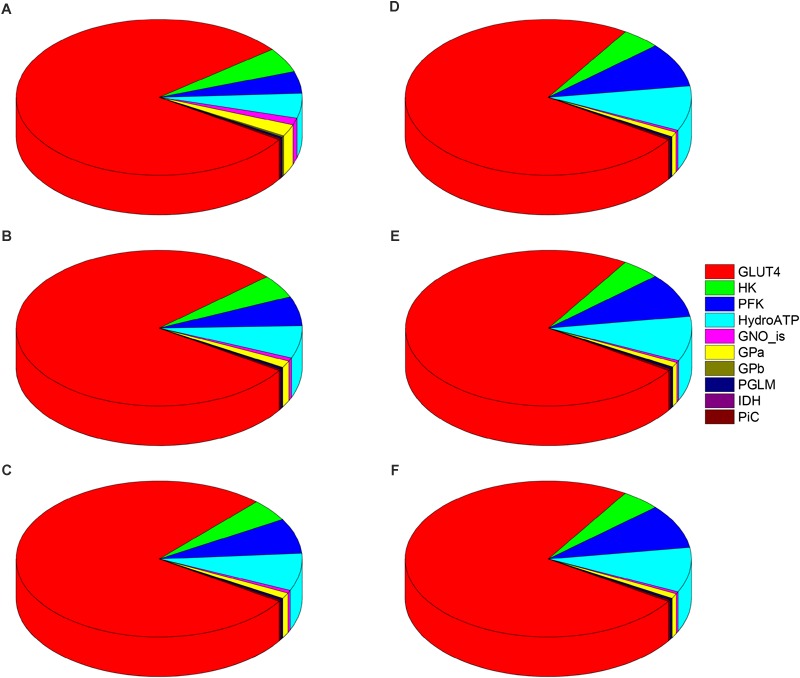
Control distribution of the flux through pyruvate kinase (PK). The pies represent the relative share of the control (as % of the total, denoted by the size of the slice of pie) exerted by the indicated enzymatic activities on the flux through PK as a representative flux of glucose metabolism. The flux control coefficients distribution is shown for the following sets of Glc and PCoA: **(A)** 5 mM, 10 μM; **(B)** 8 mM, 10 μM; **(C)** 9 mM, 10 μM; **(D)** 10 mM, 1 μM; **(E)** 10 mM; 10 μM, and **(F)** 10 mM, 40 μM. The key to the enzyme activities is provided in the colored legend on the right, and the definition of the acronyms used in the legend is given in Table 1.

Figure 6 shows the distribution of control coefficients, positive or negative, on the flux through PK. The pie chart representations A-C (Figure 6), corresponding to increasing Glc levels at constant PCoA, show a slight trend to decreasing control by the glucose uptake (i.e., pie slice in red), from 80, 79, to 77% share of the total control at 5, 8, and 9 mM Glc, respectively. Concomitantly, the control exerted by other processes on the glycolytic flux increased (Figure 6A–C). This pattern of control of PK was very similar under 1, 10, and 40 μM PCoA at constant 10 mM Glc (Figure 6D–F).

The control by ATP demand from cytoplasmic processes (pie slice in cyan) increased with the level of glucose. At 5mM Glc, control by the ATP demand and PFK increased from ∼5 and 4.5%, respectively, while at 10mM Glc both activities’ control augmented to ∼ 9%, irrespective of PCoA level (Figure 6). Likely, this trend is due to ATP being at the same time the substrate of ATP demand and a substrate/modulator of PFK activity. On the other hand, the control of glycolysis by the enzyme activities involved in glycogen degradation decreased with increasing external Glc concentration (magenta and yellow pie slices) as expected, since external glucose uptake and glycogen degradation supply carbohydrates for energy provision, thus when external glucose is scarce the relative contribution of glycogen to carbohydrate catabolism increases.

**FIGURE 7:**
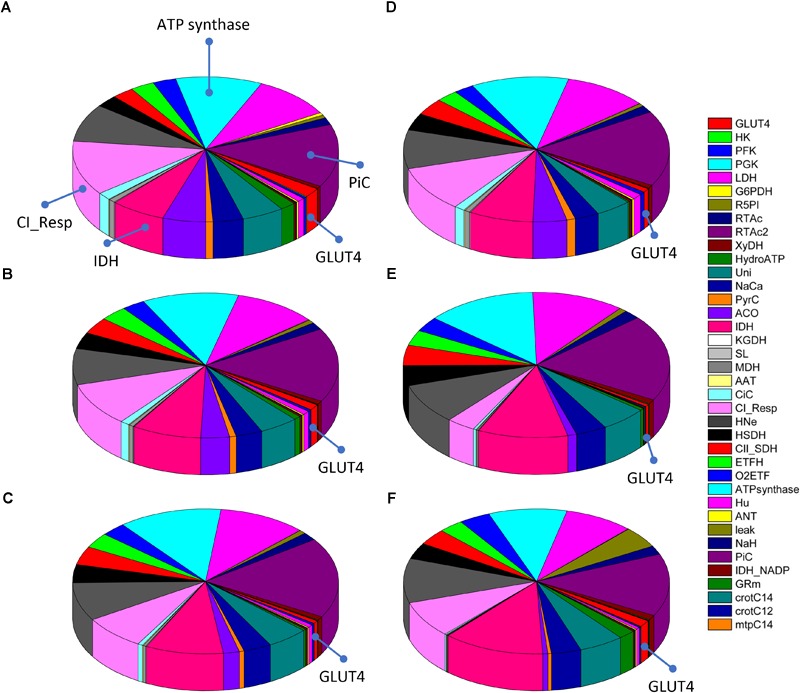
Control distribution of the flux through carnitine palmitoyl transferase 1 (CPT1). The pies represent the relative share of the control (as % of the total, denoted by the size of the slice of pie) exerted by the indicated enzymatic activities on the flux through CPT1 as a representative flux of FA metabolism. The flux control coefficients distribution is shown for the following sets of Glc and PCoA: **(A)** 5 mM, 10 μM; **(B)** 8 mM, 10 μM; **(C)** 9 mM, 10 μM; **(D)** 10 mM, 1 μM; **(E)** 10 mM; 10 μM, and **(F)** 10 mM, 40 μM. The key to the enzyme activities is provided in the colored legend on the right. Some indicators of the reactions were added in **(A)** to ease the recognition of the process involved, and the definition of the acronyms used in the legend is given in Table 1.

Figure 7 shows the distribution of control coefficients of the flux through CPT1. Under the conditions studied, OxPhos activities, including ATP synthase, phosphate transport, and respiratory electron transport complexes from electron donors NADH, Succinate or FADH_2_ (i.e., ETF generated in β-oxidation), are the most rate-controlling steps of the FAs oxidation flux. Other mitochondrial activities such as the TCA cycle, H^+^ leak and ion transport (Ca^2+^, and Na^+^), also exert control over β-oxidation. As expected, control by the H^+^ leak, which is dependent on the level of PCoA (acting as an uncoupler at high concentrations) (Cortassa et al., 2017a), increases with FA availability. The control by cytoplasmic processes is rather small (<10% adding up all cytoplasmic activities), and only glucose uptake and ATP demand exert more than 1% of the control at 5 mM Glc. Under all conditions analyzed, the mitochondrial membrane energy transduction and ion transport processes exert more than 75% of the control over FA oxidation.

Together, the data presented show that the quantitative distribution of the flux control throughout central catabolism fueled by Glc and Palm depends upon the relative levels of both substrates; increasing Glc concentrations have higher effects than enhanced FA levels. However, qualitatively, a similar pattern of control was found throughout all conditions analyzed. Glucose uptake and ATP demand are main rate-controlling steps of the flux through glucose catabolism in the cytoplasm whereas steps from OxPhos and ion transport pathways control the flux of FA oxidation in mitochondria.

### The Control of Metabolite Concentrations in the Catabolic Network

Significant biological processes such as signaling (e.g., AMP for AMPK signaling; H_2_O_2_ for redox signaling) or epigenetic (e.g., *S*-adenosyl methionine for DNA methylation; acetylCoA for acetylation) pathways, depend on metabolites or ions concentration for triggering (Aon et al., 2016). Consequently, knowledge about how metabolites’/ions’ concentration are controlled in metabolic networks is crucial for understanding the physiological status of cells, organs and organisms.

**FIGURE 8:**
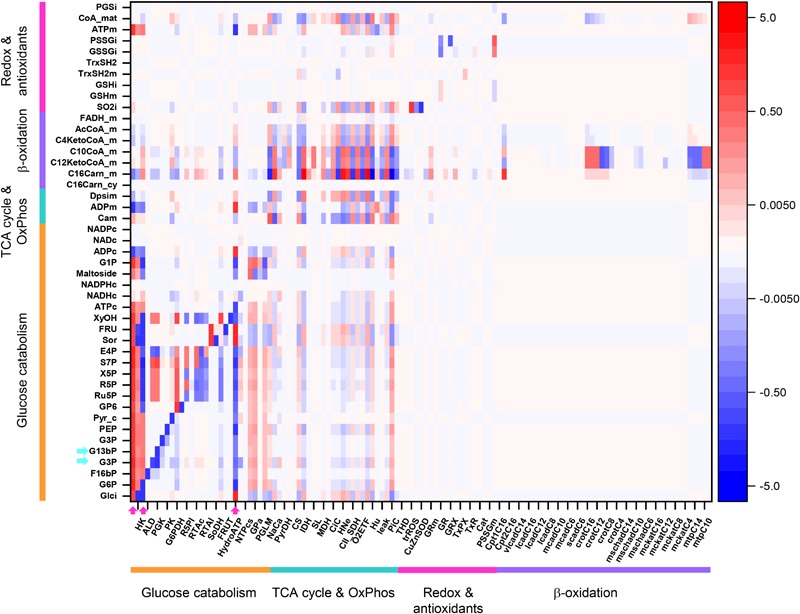
Overview heat map of metabolite control coefficients in the catabolic network of glucose and PCoA oxidation pathways. The control of metabolite concentrations (in *y*-axis) at 10 mM Glc and 10 μM PCoA (see also Figure 3 heat map for flux control coefficients) is represented as a function of the activity of the individual enzymes that catalyze metabolic reactions in the network (in *x*-axis). The value of metabolite concentration control coefficients is denoted by the intensity of red (positive control coefficient) or blue (negative control coefficient) as indicated by the color scale on the right. The key to the flux and activity labels is displayed in Table 1. Only metabolites for which the sum of the control coefficients equals less than 0.1 or -0.1 are included (i.e., the summation theorem of MCA states that the sum of concentration control coefficients for metabolites should equal 0). For visualization, the block of cytoplasmic (mainly glucose catabolism), mitochondrial (TCA cycle and OxPhos), redox and antioxidant reactions, and lipid oxidation, are indicated by colored bars. The order of the reactions is the same in all heat maps that have been presented in this work, e.g., Figure 3–5. For clarity, only one label out of two reactions is shown in the *x*-axis. However, the metabolite concentration control coefficients with respect to each one of the activities participating in the model are depicted in the heat map. Cyan arrows on the *y*-axis point to metabolites mentioned in the main text as examples, and arrows on the *x*-axis point out main rate-controlling steps of the flux (see also Figure 2). Dpsim stands for mitochondrial membrane potential, Δψ_m_. The numerical values of the metabolite concentration control coefficients are shown in Supplementary Table S24.

Figure 8 shows an overview heat map representation of concentration control coefficients of the metabolites/ions from the catabolic network at 10 mM Glc and 10 μM PCoA (same conditions as for the heat map of flux control coefficients shown in Figure 3). Displayed on the *y*-axis are metabolites and ions (e.g., protons, phosphate), including the mitochondrial membrane electric potential (Δψ_m_), the level of which is controlled by the steps shown on the *x*-axis.

Some important general control principles of metabolism can be visualized in this heatmap. Metabolite concentrations are controlled by the same rate-controlling steps of the flux through pathways to which these metabolites belong. For example, Glut4, HK, PFK, and HydroATP are main rate-controlling steps of the flux with widespread influence throughout the catabolic network (Figure 3, arrows), and the glycolytic intermediates, e.g., glyceraldehyde 3 phosphate (G3P) or glyceraldehyde 1, 3 bisphosphate (G13bP) are controlled positively by Glut4, HK or PFK (i.e., the higher the glucose uptake the higher the metabolite concentration), and negatively by HydroATP (i.e., the higher the ATP demand, the lower the metabolite concentration) (Figure 8, arrows). Additionally, metabolites are negatively controlled by the enzymes that catalyze the reactions that consume them, as observed in the blue main diagonal comprising glucose catabolic pathways (Figure 8, bottom left). Notably, the control of G3P concentration is shared by several enzymes, among them the G3P consuming glyceraldehyde 3 phosphate dehydrogenase (GAPD), which, in turn, is regulated by adenine nucleotides, ATP, ADP, and AMP, explaining that steps from OxPhos and mitochondrial respiration do exert control on G3P concentration. The heat maps of metabolite concentration control coefficients corresponding to the other combinations of glucose and PCoA concentrations explored in this work are shown in Supplementary Figures S1, S2.

Together, the data presented show that although the share of the control of cellular metabolites concentration is highly distributed, general principles apply such as the key role played by the main rate-controlling steps of the flux, and, locally, by the enzymes that consume those metabolites, enabling a comprehensive understanding of a complex but crucial aspect of metabolism and physiology.

## Discussion

In this work we have analyzed for the first time the control and regulatory properties of central catabolism fueled by glucose and fatty acids, using a comprehensive, integrative computational model, allied with a quantitative systems biology approach. Control analysis under different relative supply levels of glucose and palmitate showed the interdependence and mutual inhibition between degradation pathways, consistent with reported data (Randle et al., 1963; Hue and Taegtmeyer, 2009). Using overview heat maps of flux and metabolites’ concentration control coefficients, we found that the flux control: (i) by glucose uptake, phosphorylation (PFK and HK), cytoplasmic ATP demand, and PiC (Figure 2) is widespread, extending beyond specific pathways and influencing the whole catabolic network, and (ii) through the FA oxidation pathway is under the control of the TCA cycle, respiratory electron transport and OxPhos steps, all novel findings that open new opportunities for further research. Importantly, despite quantitative variations in substrates level, the same pattern of control and regulation was obtained under all substrate combinations tested. This both unexpected as well as original finding ascertains once more the inherent interdependence of multiple pathways distributed in different cellular compartments.

A remarkable and rather unexpected result is the relatively large control exerted by the mitochondrial PiC over β-oxidation, and its broad influence throughout central catabolism (Figure 3–5, 7). Deficiencies in the human mitochondrial PiC (SLC25A3, OMIM 600370), leading to homozygous-lethality, have been associated with inborn errors of metabolism^1^ (Mayr et al., 2007), and its impact on mitochondrial and cellular energetics is just starting to be recognized (Seifert et al., 2016). The rather unexplored impact of the PiC deserves further research.

Flux control coefficients quantitate network systemic properties that heat maps help visualize as overview patterns of control coefficients (flux and metabolite concentrations) in central catabolism. Heat maps also enable a quick localization of the most rate-controlling processes in the network, while highlighting the distributed nature of the control. For example, the control of successive reaction steps displays the same control coefficients because all steady state fluxes are equal in a linear pathway. On the contrary, at branching points the sign of the control coefficients is inverted because the steps in one branch negatively control the fluxes on the other branch. This is illustrated by PFK that controls positively glycolysis and negatively the pentose phosphate pathway (Figure 3–5). Also, the fact that many processes control themselves can also be visualized in heat maps as red cells along the main diagonal of the graph.

The widespread control exerted by steps upstream of glucose metabolism (Figure 3) depends upon the exogenous glucose concentration, according to our model simulations. For example, an inversion of the flux control coefficients from negative to positive occurs at 9 mM Glc (compared to lower Glc concentrations), for the flux control exerted by glycolytic activities on, e.g., the TCA cycle (Figure 4, compare panel Figure 4D with Figure 4A,B). This shift in control can be explained by the speeding of mitochondrial catabolic reactions due to a larger provision of anaplerotic intermediates to the TCA cycle. Likewise, the shift to negative control coefficients among the acylCoA dehydrogenases in β-oxidation is due to several dehydrogenases “competing” for the same substrate, e.g., lauroylCoA (C12CoA), which is substrate of the very long-, long- and medium-chain acylCoA dehydrogenases (vlcad, lcad, and mcad), all of which will catalyze its transformation into C12enoylCoA. Other authors have highlighted the control of β-oxidation by the activities of acetylCoA carboxylase and malonylCoA decarboxylase that modulate malonylCoA level (Lopaschuk and Stanley, 2006; Koves et al., 2008; Lopaschuk et al., 2010), a pathway that is also target of AMPK signaling which senses cellular energy availability affecting, among others, malonylCoA decarboxylase (Cuthbert and Dyck, 2005; Stanley et al., 2005; Bouzakri et al., 2008).

Previous studies applying MCA have revealed that glucose metabolism in working rat hearts was mainly controlled at the level of glucose uptake and phosphorylation by hexokinase (Kashiwaya et al., 1994). In those studies, the presence of insulin abolished the control by glucose uptake. Our modeling results parameterized under conditions in which mouse hearts were Langendorff-perfused in the absence of insulin, and glucose and FAs were present together with a β-agonist isoproterenol, agree with results from Kashiwaya et al. (1994), in the presence of glucose alone. In the present work, being glucose uptake the main rate-controlling step of the flux through glucose catabolism under all conditions studied, the reported effect of insulin on control of glucose uptake could be explained by its promoting action on translocation of the glucose transporter Glut4 to the plasma membrane (Cushman et al., 1984; Uphues et al., 1994). Numerous reports have described the pervasive insulin effects on metabolism in various mammalian systems, including glycogen storage and lipid metabolism mediated by PI3K/AKT (Goodwin et al., 1995; Schafer et al., 2004; Bouzakri et al., 2008; Courtnay et al., 2015). Considering the magnitude of the control shared by glucose uptake on the flux control throughout cytoplasmic pathways (Figure 3–6), the present results suggest that the insulin action might be, at least in part, mediated by direct action on the GLUT1/4 transporter activity, in addition to other signaling mechanisms.

The divergent behavior of the flux control displayed by PFK and PDH invites revising the concepts of control and regulation (Fell, 1996; Cortassa et al., 2012). PFK and PDH are two enzyme activities highly regulated by a large set of biochemical species (Roche et al., 2001; Sola-Penna et al., 2010; Cortassa et al., 2017b) which led to the notion that they are central control points in mammalian metabolism. The concept of control that refers to the impact of the maximal activity of an enzyme catalyzed step, either through its concentration or catalytic performance, should be distinguished from that of regulation, which denotes the ability of an enzyme to respond to the action of effectors, thus changing the reaction rate. PFK is regulated by ADP, AMP, F2,6bP which activate the enzyme, and ATP and citrate favoring the formation of dimers leading to inhibition of the enzyme activity (Schoneberg et al., 2013; Gibb et al., 2017). In turn, PDH is tightly regulated by reversible phosphorylation and dephosphorylation by PDK and PDP, respectively, of the E1 component of the PDH complex (Li et al., 2009). PDH activation/inhibition in response to a set of effectors have been modeled (Cortassa et al., 2018b), and the resulting rate expression has been incorporated into the model of central catabolism (see eqn. S112 in Supplementary Material). Thus, according to the steady state levels of the effectors, the PDH activity attains different levels (Figure 2). The elasticity of PDH is very large for the ratio AcCoA/CoA under all conditions explored in this work, which is consistent with the low control coefficient (as implied by the connectivity theorem of MCA, see Supplementary Material). On the other hand, PFK is modulated by ATP (inhibitor) and its elasticity value is much smaller in magnitude which, in addition to network localization, explains why this enzyme has a relatively large control coefficient while PDH does not exert control but instead regulates substrate selection by modulating the level of AcCoA supplied by glycolysis through pyruvate or by β-oxidation. Interestingly, a recent report highlighted the regulatory role of PFK2 kinase and phosphatase activities on PFK1 in relation to changes in glycolytic fluxes during exercise (Gibb et al., 2017).

The magnitude of the glucose oxidation inhibition by FAs found in our model simulations, i.e., 15–20% decrease in PDH activity, is much smaller than the 4- to 10-fold decrease observed in working hearts in the presence of low and high fat, respectively, compared to the absence of exogenous fat (Saddik and Lopaschuk, 1991). Under those conditions, the glucose uptake rate would double in the presence of insulin whereas the rate of FA oxidation from exogenous or endogenous origin would be insensitive to the presence of the pancreatic hormone. The discrepancy between our modeling results and reported experimental evidence, could be due to our present model formulation not accounting for mechanisms of insulin action. *In vivo*, other hormones would influence catabolic fluxes, such as glucagon, catecholamines, and leptin. These effects will be considered in future editions of our modeling.

### Limitations of This Study

Important aspects are not considered by the present version of the model such as signaling mechanisms of hormonal origin, like insulin action, which was absent in the *ex vivo* heart perfusion experiments used to parameterize the model. The activity of cytoplasmic ATP citrate lyase, that converts citrate into AcCoA, is not included, while adenylate kinase, that interconverts adenine nucleotides, is only implicitly considered in an aggregated, generalized energy demand (HydroATP). Consequently, cytoplasmic citrate and AMP are not state variables but parameters in the model. Due to the importance of AMP as a modulator of PFK, we investigated the effect of micromolar levels of AMP under 10 mM Glc/10 μM PCoA (see Supplementary Figure S3). Even though the fluxes through glucose catabolism decreased as a function of decreasing AMP concentrations, the control either positive or negative was exerted by the same processes irrespective of the level of AMP. Concerning citrate, even if it were a state variable, its levels in mitochondria vary between 0.8 and 1.1 μM which is much smaller than the inhibitory range of PFK. Other authors (Kauppinen et al., 1986) have demonstrated that the cytoplasmic pool of citrate is 16-fold lower than in mitochondria, suggesting that citrate will likely not operate as a physiological inhibitor under physiological conditions. Neither considered is PFK2 activity that catalyzes the formation of Fru2,6bP, an important regulator of PFK1 that is known to be activated upon ischemia in mammalian hearts (Hue and Taegtmeyer, 2009; Gibb et al., 2017). Another limitation of our model is that malonylCoA is not a state variable since quantitative data characterizing the kinetic properties of both malonylCoA decarboxylase and AcCoA carboxylase are not available.

The size and complexity of the metabolic network described by our computational model encompass processes sustaining widely different fluxes. For example, glucose catabolic pathways vary between 10^-3^ and 10^-5^ mM ms^-1^, whereas ROS and antioxidant pathways operate in the 10^-8^–10^-10^ mM ms^-1^ scale. This broad range of flux values may negatively condition the matrices to be inverted for the control calculations producing inaccurate control coefficients (see Supplementary Material Section 2.1.1). As a control, we utilized an alternative method (finite differences), which has better numerical stability, and compared the results (see Supplementary Table S17). Using this procedure, the flux control coefficient of PFK showed close agreement between both methods (difference <2.5%) for pathways sustaining high fluxes (glucose catabolism) whereas for those displaying intermediate (TCA cycle, β-oxidation) or low (antioxidants) fluxes, the difference was higher but within the same order of magnitude. Taking into account (i) the convenience of the matrix *vs.* the finite difference method for high throughput calculations, and (ii) that pathways such as antioxidant systems and other alternative routes (polyols) exert negligible control over substrate selection but confer robustness to complex networks function under relevant but specific (patho)physiological conditions (oxidative stress, excess substrate), we consider our results acceptable under the conditions described herein. Additional work will be needed to further adapt the analytical tools of MCA to stiff systems that mimic real, complex, biological networks.

## Conclusion

As far as we are aware, this is the first time that a validated, comprehensive computational model of central catabolism distributed in cytoplasmic and mitochondrial compartments, including detailed kinetics and known regulatory mechanisms, is presented and its control and regulatory properties quantitatively analyzed as applied to substrate selection between glucose and FAs. It is shown that both substrates, to a greater or lesser extent, fuel mitochondria. Substrate selection changes as a function of the relative levels of Glc and FA, reciprocally influencing each other. Under these conditions, quantitative rather than qualitative changes in the profile pattern of flux and metabolites’ concentration control coefficients were corroborated, i.e., the main rate-controlling steps of fluxes and metabolite concentrations remained the same.

## Author Contributions

SC, MA, and SS contributed to conception and design of the study. SC designed and analyzed the model simulations, performed the control analysis, and wrote the first draft of the manuscript. MA wrote sections of the manuscript. All authors contributed to manuscript edition, read and approved the submitted version.

## Conflict of Interest Statement

The authors declare that the research was conducted in the absence of any commercial or financial relationships that could be construed as a potential conflict of interest.

## Footnotes

1 https://omim.org/entry/600370
