# Supplementary material for: Control and Regulation of Substrate Selection in Cytoplasmic and Mitochondrial Catabolic Networks. A Systems Biology Analysis
Source: Front Physiol. 2019 Mar 8;10:201. doi: 10.3389/fphys.2019.00201 (PMC6418011; doi:10.3389/fphys.2019.00201)
Supplement: Supplementary file 1 [file Data_Sheet_1.pdf]

Supplementary Material

Control and regulation of substrate selection in cytoplasmic and mitochondrial catabolic networks. A systems biology analysis

Sonia Cortassa, Miguel A. Aon and Steven J. Sollott

* **Correspondence:** Sonia Cortassa: sonia.cortassa@nih.gov

Supplementary Figures

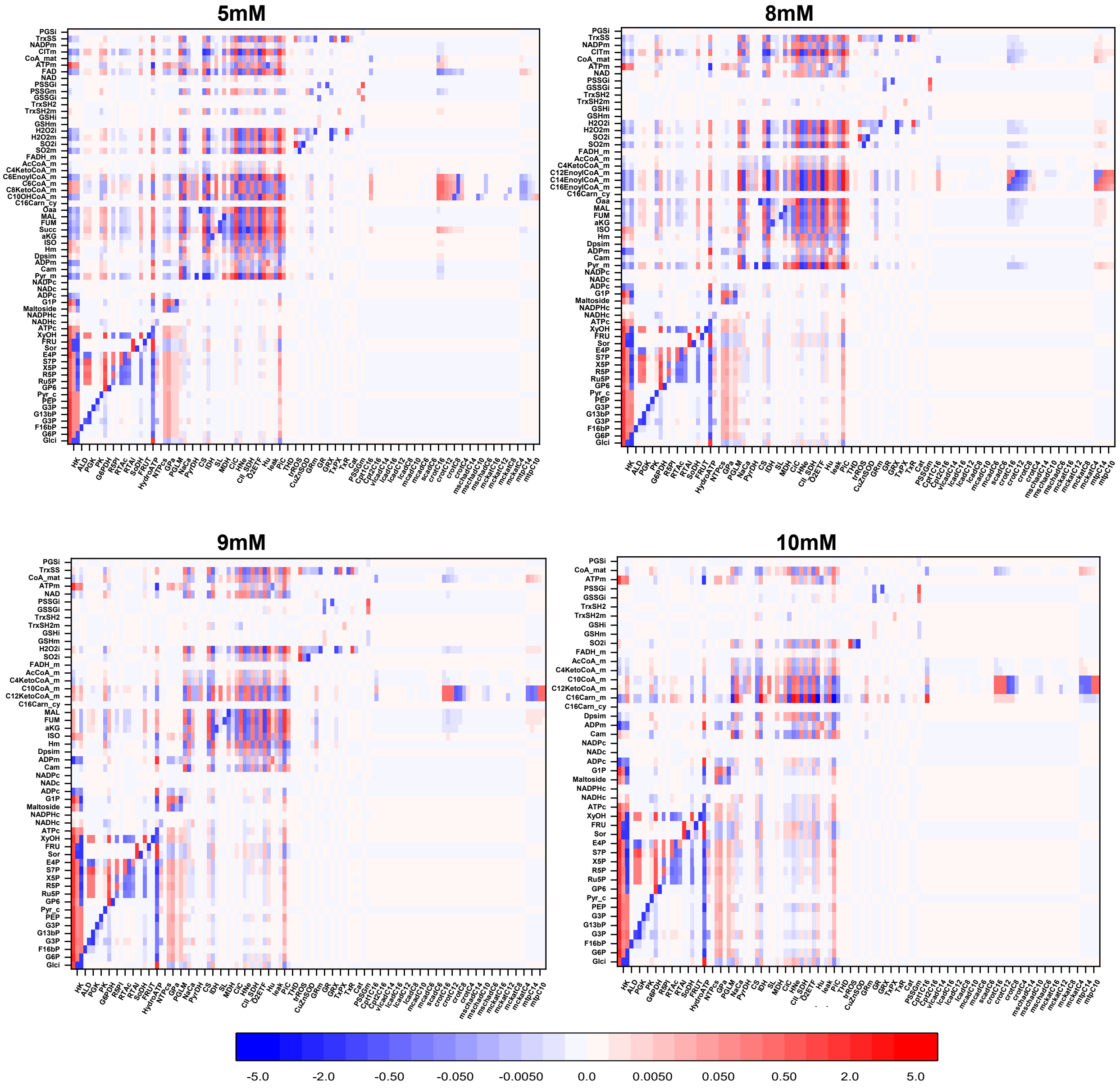

Supplementary Figure S1. Overview heat maps of metabolite concentration control coefficients exerted by individual enzymes/transport processes over metabolic reaction steps in the catabolic network following increasing Glc concentration at constant 10 µM PCoA.

Heatmaps of metabolite concentration control coefficients obtained by metabolic control analysis of the steady states obtained with the catabolic network model from glucose and Palmitoyl CoA. The conditions for these simulations were at constant 10 µM PCoA, and variable glucose in the extracellular medium as indicated above or below the corresponding panel (in mM). The red-blue scale represents the magnitude of the metabolite concentration control coefficients, and is the same for all panels, including the key to the processes, as shown in Figure 2.

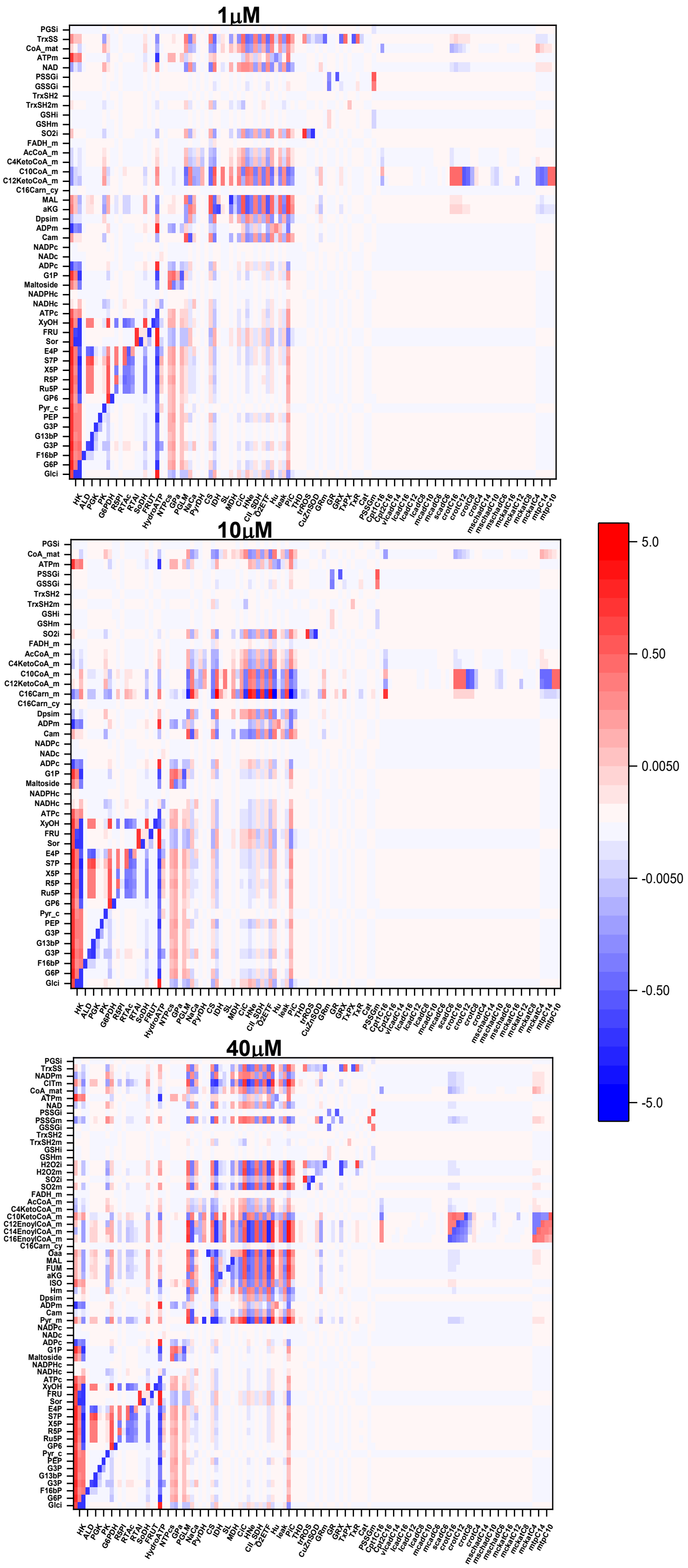

Supplementary Figure S2. Overview heat maps of metabolite concentration control coefficients exhibited by individual enzymes/transport processes over metabolic reaction steps in the catabolic network following increasing PCoA concentration at constant 10mM Glc.

Heatmaps of metabolite concentration control coefficients obtained by metabolic control analysis of the steady states obtained with the catabolic network model using Glc and PCoA. The conditions for these simulations were 10mM Glc, and variable PCoA as indicated on top of each panel (in µM). The red-blue scale represents the magnitude of the flux control coefficients, and is the same for all panels, including the key to the processes, shown in Figure 2.

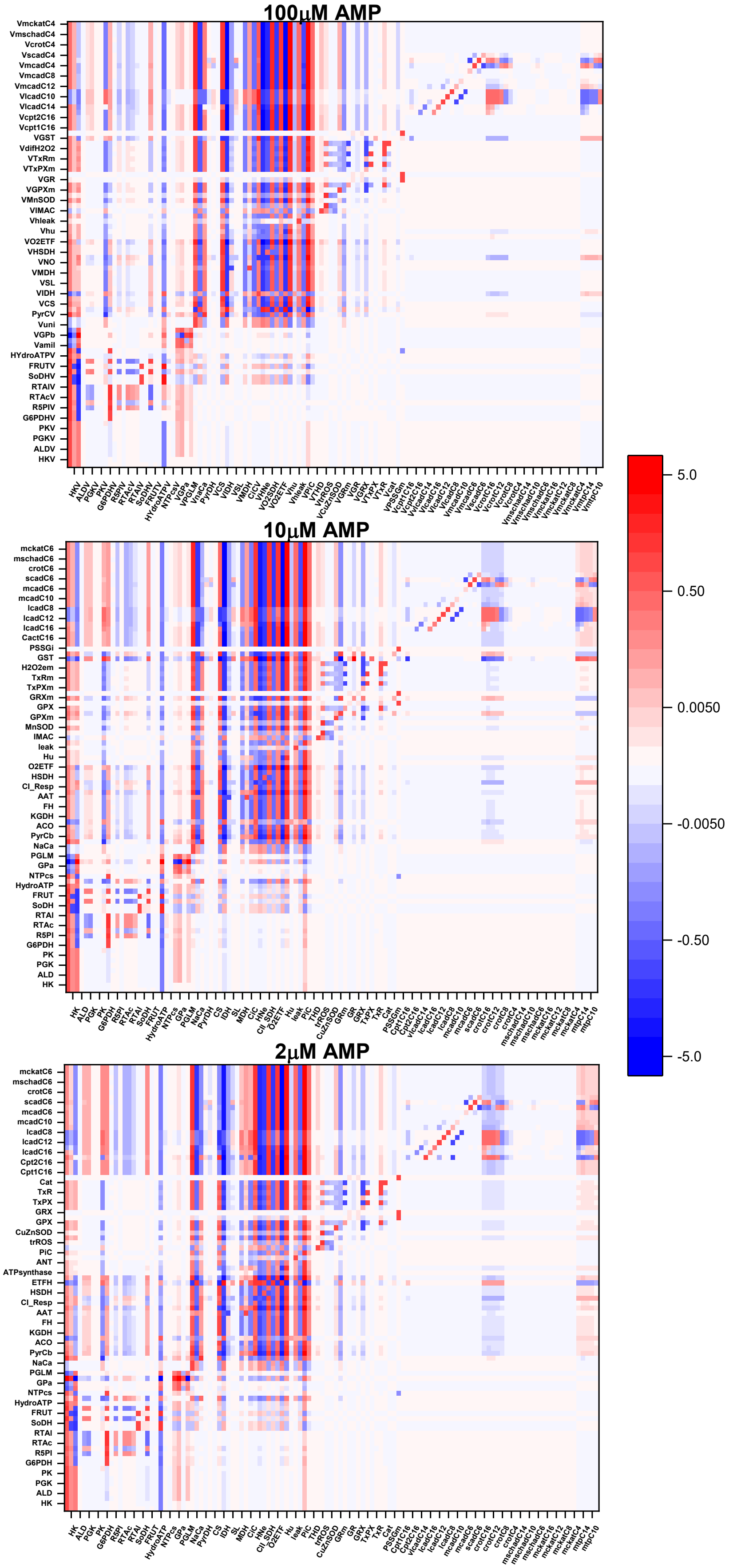

Supplementary Figure S3. Heatmaps of flux control coefficients exerted by individual enzymes/transport processes over metabolic reaction steps in the catabolic network following increasing AMP concentration at constant 10mM Glc and 10 μM PCoA.

Heatmaps of flux control coefficients obtained by metabolic control analysis of the steady states obtained with the catabolic network model. The conditions for these simulations were 10mM Glc, 10 μM PCoA and variable AMP (100, 10 and 2) as indicated on top each panel (in μM). The red-blue scale represents the magnitude of the flux control coefficients, and is the same for all panels, including the key to the processes, shown in Figure 2.

1 Supplementary Section S1. Model Description

1.1 Model ordinary differential equations (ODEs)

$$\frac{d[Glc]_i}{dt} = V_T^{Glc} - V_{HK} - V_{ALDR} \tag{S1}$$

$$\frac{d[H6P]}{dt} = V_{HK} + V_{PGLM} - V_{PFK} - V_{G6PD} + V_{TAL} + V_{TK2} \tag{S2}$$

$$\frac{d[FbP]}{dt} = V_{PFK} - V_{ALD} \tag{S3}$$

$$\frac{d[G3P]}{dt} = 2.V_{ALD} - V_{GAPD} + V_{TK1} - V_{TAL} + V_{TK2} \tag{S4}$$

$$\frac{d[BPG]}{dt} = V_{GAPD} - V_{PGK} \tag{S5}$$

$$\frac{d[3PG]}{dt} = V_{PGK} - V_{Enol} \tag{S6}$$

$$\frac{d[PEP]}{dt} = V_{Enol} - V_{PK} \tag{S7}$$

$$\frac{d[Pyr]}{dt} = V_{PK} - V_{LDH} - V_T^{Pyr} \tag{S8}$$

$$\frac{d[Sor]}{dt} = V_{ALDR} - V_{SoDH} \tag{S9}$$

$$\frac{d[Fru]}{dt} = V_{SoDH} - V_T^{Fru} \tag{S10}$$

$$\frac{d[6PG]}{dt} = V_{G6PD} - V_{6PGO} \tag{S11}$$

$$\frac{d[Ru5P]}{dt} = V_{6PGO} - V_{R5PI} - V_{Ru5PE} \tag{S12}$$

$$\frac{d[R5P]}{dt} = V_{R5PI} - V_{TK1} \tag{S13}$$

$$\frac{d[X5P]}{dt} = V_{Ru5PE} - V_{TK1} - V_{TK2} - V_{XyDH} \tag{S14}$$

$$\frac{d[S7P]}{dt} = V_{TK1} - V_{TAL} \tag{S15}$$

$$\frac{d[E4P]}{dt} = V_{TAL} - V_{TK2} \tag{S16}$$

$$\frac{d[XyOH]}{dt} = V_{XyDH} - V_{T}^{XyOH} \tag{S17}$$

$$\frac{d[Mal]}{dt} = V_{Gno\_is} - 0.4 \cdot V_{GPa} - 0.6 \cdot V_{GPb} \tag{S18}$$

$$\frac{d[G1P]}{dt} = 0.4 \cdot V_{GPa} + 0.6 \cdot V_{GPb} - V_{PGLM} \tag{S19}$$

$$\frac{d[ATP]_i}{dt} = -V_{HK} - V_{PFK} + V_{PGK} + V_{PK} - V_{cATPase} \tag{S20}$$

$$\frac{d[NADH]_i}{dt} = V_{GAPD} - V_{LDH} + V_{SoDH} + V_{XyDH} - V_{NADHo} \tag{S21}$$

$$\frac{d[NADPH]_i}{dt} = V_{G6PD} + V_{6GLO} - V_{ALDR} - V_{NADPHo} \tag{S22}$$

$$\frac{d[Pyr]_m}{dt} = \frac{Vc}{Vm} V_{T}^{Pyr} - V_{PDH} - V_{PCb} + V_{ME} \tag{S23}$$

$$\frac{d[Ca^{2+}]_m}{dt} = \delta_{Ca}\left(V_{uni} - V_{NaCa}\right) \tag{S24}$$

$$\frac{d\,[ADP]_m}{dt} = V_{ANT} - V_{ATPase} - V_{SL} + V_{PCb} \tag{S25}$$

$$\frac{d\Delta\Psi_m}{dt} = \frac{V_{He} + V_{He(SDH)} + V_{He(ETF)} - V_{Hu} - V_{ANT} - V_{Hleak} - V_{NaCa} - V_{uni} - V_{IMAC}}{C_{mito}} \quad \text{(S26)}$$

$$\frac{d[NADH]_m}{dt} = -V_{O_2} + V_{IDH} + V_{KGDH} + V_{MDH} - V_{THD} + V_{mtp}^{C16} + V_{mschad}^{C16} + V_{mtp}^{C14} + V_{mschad}^{C14} + \cdots$$
$$V_{mtp}^{C12} + V_{mschad}^{C12} + V_{mtp}^{C10} + V_{mschad}^{C10} + V_{mtp}^{C8} + V_{mschad}^{C8} + V_{mschad}^{C6} + V_{mschad}^{C4} \quad \text{(S27)}$$

$$\frac{d[H^+]_m}{dt} = \delta_H \left(-V_{He} - V_{He(SDH)} - V_{He(ETF)} + V_{Hu} + V_{NaH} + V_{PiC} + V_{Hleak} \right) \quad \text{(S28)}$$

$$\frac{d[Pi]_m}{dt} = -V_{ATPase} + V_{PiC} - V_{SL} \quad \text{(S29)}$$

$$\frac{d[ISOC]}{dt} = V_{ACO} - V_{IDH} - V_{IDH\_NADP} \quad \text{(S30)}$$

$$\frac{d[\alpha KG]}{dt} = V_{IDH} + V_{IDH\_NADP} - V_{KGDH} + V_{ATT} \quad \text{(S31)}$$

$$\frac{d[SCoA]}{dt} = V_{KGDH} - V_{SL} \quad \text{(S32)}$$

$$\frac{d[Suc]}{dt} = V_{SL} - V_{O_2SDH} \quad \text{(S33)}$$

$$\frac{d[FUM]}{dt} = V_{O_2SDH} - V_{FH} \quad \text{(S34)}$$

$$\frac{d[MAL]}{dt} = V_{FH} - V_{MDH} + V_{CiC} - V_{ME} \quad \text{(S35)}$$

$$\frac{d[OAA]}{dt} = V_{MDH} - V_{CS} - V_{AAT} \quad \text{(S36)}$$

$$\frac{d[CIT]}{dt} = V_{CS} - V_{ACO} - V_{CiC} \quad \text{(S37)}$$

$$\frac{d[C16Carn]_i}{dt} = V_{CPT1} - V_{CACT} \tag{S38}$$

$$\frac{d[C16Carn]_m}{dt} = V_{CACT} - V_{CPT2} \tag{S39}$$

$$\frac{d[C16CoA]_m}{dt} = V_{CPT2} - V_{vlcad}^{C16} - V_{lcad}^{C16} \tag{S40}$$

$$\frac{d[C16enoylCoA]_m}{dt} = V_{vlcad}^{C16} + V_{lcad}^{C16} - V_{cro}^{C16} - V_{mtp}^{C16} \tag{S41}$$

$$\frac{d[C16OHCoA]_m}{dt} = V_{cro}^{C16} - V_{mschad}^{C16} \tag{S42}$$

$$\frac{d[C16ketoCoA]_m}{dt} = V_{mschad}^{C16} - V_{mckat}^{C16} \tag{S43}$$

$$\frac{d[C14CoA]_m}{dt} = V_{mckat}^{C16} + V_{mtp}^{C16} - V_{vlcad}^{C14} - V_{lcad}^{C14} \tag{S44}$$

$$\frac{d[C14enoylCoA]_m}{dt} = V_{vlcad}^{C14} + V_{lcad}^{C14} - V_{cro}^{C14} - V_{mtp}^{C14} \tag{S45}$$

$$\frac{d[C14OHCoA]_m}{dt} = V_{cro}^{C14} - V_{mschad}^{C14} \tag{S46}$$

$$\frac{d[C14ketoCoA]_m}{dt} = V_{mschad}^{C14} - V_{mckat}^{C14} \tag{S47}$$

$$\frac{d[C12CoA]_m}{dt} = V_{mckat}^{C14} + V_{mtp}^{C14} - V_{vlcad}^{C12} - V_{lcad}^{C12} - V_{mcad}^{C12} \tag{S48}$$

$$\frac{d[C12enoylCoA]_m}{dt} = V_{vlcad}^{C12} + V_{lcad}^{C12} + V_{mcad}^{C12} - V_{cro}^{C12} - V_{mtp}^{C12} \tag{S49}$$

$$\frac{d[C12OHCoA]_m}{dt} = V_{cro}^{C12} - V_{mschad}^{C12} \tag{S50}$$

$$\frac{d[C12ketoCoA]_m}{dt} = V_{mschad}^{C12} - V_{mckat}^{C12} \tag{S51}$$

$$\frac{d[C10CoA]_m}{dt} = V_{mckat}^{C12} + V_{mtp}^{C12} - V_{lcad}^{C10} - V_{mcad}^{C10} \tag{S52}$$

$$\frac{d[C10enoylCoA]_m}{dt} = V_{lcad}^{C10} + V_{mcad}^{C10} - V_{cro}^{C10} - V_{mtp}^{C10} \tag{S53}$$

$$\frac{d[C10OHCoA]_m}{dt} = V_{cro}^{C10} - V_{mschad}^{C10} \tag{S54}$$

$$\frac{d[C10ketoCoA]_m}{dt} = V_{mschad}^{C10} - V_{mckat}^{C10} \tag{S55}$$

$$\frac{d[C8CoA]_m}{dt} = V_{mckat}^{C10} + V_{mtp}^{C10} - V_{lcad}^{C8} - V_{mcad}^{C8} \tag{S56}$$

$$\frac{d[C8enoylCoA]_m}{dt} = V_{lcad}^{C8} + V_{mcad}^{C8} - V_{cro}^{C8} - V_{mtp}^{C8} \tag{S57}$$

$$\frac{d[C8OHCoA]_m}{dt} = V_{cro}^{C8} - V_{mschad}^{C8} \tag{S58}$$

$$\frac{d[C8ketoCoA]_m}{dt} = V_{mschad}^{C8} - V_{mckat}^{C8} \tag{S59}$$

$$\frac{d[C6CoA]_m}{dt} = V_{mckat}^{C8} + V_{mtp}^{C8} - V_{scad}^{C6} - V_{mcad}^{C6} \tag{S60}$$

$$\frac{d[C6enoylCoA]_m}{dt} = V_{scad}^{C6} + V_{mcad}^{C6} - V_{cro}^{C6} \tag{S61}$$

$$\frac{d[C6OHCoA]_m}{dt} = V_{cro}^{C6} - V_{mschad}^{C6} \quad \text{(S62)}$$

$$\frac{d[C6ketoCoA]_m}{dt} = V_{mschad}^{C6} - V_{mckat}^{C6} \quad \text{(S63)}$$

$$\frac{d[C4CoA]_m}{dt} = V_{mckat}^{C6} - V_{scad}^{C4} - V_{mcad}^{C4} \quad \text{(S64)}$$

$$\frac{d[C4enoylCoA]_m}{dt} = V_{scad}^{C4} + V_{mcad}^{C4} - V_{cro}^{C4} \quad \text{(S65)}$$

$$\frac{d[C4OHCoA]_m}{dt} = V_{cro}^{C4} - V_{mschad}^{C4} \quad \text{(S66)}$$

$$\frac{d[C4ketoCoA]_m}{dt} = V_{mschad}^{C4} - V_{mckat}^{C4} \quad \text{(S67)}$$

$$\frac{d[AcCoA]_m}{dt} = -V_{CS} + V_{mtp}^{C16} + V_{mckat}^{C16} + V_{mtp}^{C14} + V_{mckat}^{C14} + V_{mtp}^{C12} + V_{mckat}^{C12} + \cdots$$
$$V_{mtp}^{C10} + V_{mckat}^{C10} + V_{mtp}^{C8} + V_{mckat}^{C8} + V_{mckat}^{C6} + 2V_{mckat}^{C4} \quad \text{(S68)}$$

$$\frac{d[FADH_2]_m}{dt} = -V_{O_2}^{ETF} + V_{vlcad}^{C16} + V_{lcad}^{C16} + V_{vlcad}^{C14} + V_{lcad}^{C14} + V_{vlcad}^{C12} + V_{lcad}^{C12} + \cdots$$
$$V_{mcad}^{C12} + V_{lcad}^{C10} + V_{mcad}^{C10} + V_{lcad}^{C8} + V_{mcad}^{C8} + V_{mcad}^{C6} + V_{scad}^{C6} + V_{mcad}^{C4} + V_{scad}^{C4} \quad \text{(S69)}$$

$$\frac{d[NADPH]_m}{dt} = V_{IDH\_NADP} + V_{THD} + V_{ME} - V_{GRm} - V_{TxRm} \quad \text{(S70)}$$

$$\frac{d[O_2^{\bullet-}]_m}{dt} = shunt\left(V_{O_2} + V_{O_2SDH}\right) - V_{MnSOD} - V_{ROS}^{Tr} \quad \text{(S71)}$$

$$\frac{d[O_2^{\bullet-}]_i}{dt} = \frac{v_m}{v_i} V_{ROS}^{Tr} - V_{CuZnSOD} \quad \text{(S72)}$$

$$\frac{d[H_2O_2]_m}{dt} = V_{MnSOD} - V_{difH_2O_2} - V_{GPXm} - V_{TxPXm} \tag{S73}$$

$$\frac{d[H_2O_2]_i}{dt} = V_{CuZnSOD} + \frac{v_m}{v_i} V_{difH_2O_2} - V_{GPXi} - V_{TxPXi} - V_{CAT} \tag{S74}$$

$$\frac{d[GSH]_m}{dt} = V_{GRm} - V_{GPXm} - V_{GRXm} + V_{GST} - V_{PSSGm} \tag{S75}$$

$$\frac{d[GSH]_i}{dt} = V_{GRi} - V_{GPXi} - V_{GRXi} + \frac{v_m}{v_i} V_{GST} - V_{PSSGi} \tag{S76}$$

$$\frac{d[GSSG]_m}{dt} = 0.5\left(V_{GPXm} - V_{GRm}\right) + V_{GRXm} \tag{S77}$$

$$\frac{d[GSSG]_i}{dt} = 0.5\left(V_{GPXi} - V_{GRi}\right) + V_{GRXi} \tag{S78}$$

$$\frac{d[TxR]_m}{dt} = V_{TxRm} - V_{TxPXm} \tag{S79}$$

$$\frac{d[TxR]_i}{dt} = V_{TxRi} - V_{TxPXi} \tag{S80}$$

$$\frac{d[PSSG]_m}{dt} = V_{PSSGm} - V_{GRXm} \tag{S81}$$

$$\frac{d[PSSG]_i}{dt} = V_{PSSGi} - V_{GRXi} \tag{S82}$$

1.2 Rate Equations and parameters included in the model of the catabolic network

1.2.1 Glycolysis

***Glucose transport*:**

$$V_T^{Glc} = k_T^{Glc}\left(Glc_o - Glc_i\right) \tag{S83}$$

Hexokinase (HK)

$$V_{HK} = V_{\max}^{HK} \cdot \frac{\left(\frac{ATP_i \,.\, Glc_i}{K_{M\,HK}^{ATP} . K_{M\,HK}^{Glc}} - \frac{\left(C_T^{ad} - ATP_i \right) . \, H6P}{K_{M\,HK}^{ATP} . K_{M\,HK}^{Glc} K_{HK}^{eq}} \right)}{1 + \frac{Glc_i}{K_{M\,HK}^{Glc}} + \frac{ATP_i}{K_{M\,HK}^{ATP}} + \frac{ATP_i \,.\, Glc_i}{K_{M\,HK}^{ATP} . K_{M\,HK}^{Glc}} + \frac{H6P}{K_{M\,HK}^{H6P}} + \frac{\left(C_T^{ad} - ATP_i \right)}{K_{M\,HK}^{ADP}} + \frac{\left(C_T^{ad} - ATP_i \right) . H6P}{K_{M\,HK}^{ADP} K_{M\,HK}^{H6P}}} \quad \text{(S84)}$$

Phosphofructokinase (PFK)

$$V_{PFK} = \frac{\left(V_{\max,f}^{PFK} \frac{ATP_i \,.\, \mathrm{H6P}}{K_{M\,PFK}^{ATP} . K_{M\,PFK}^{H6P}} - V_{\max,r}^{PFK} \frac{\left(C_T^{ad} - ATP_i \right) . FBP}{K_{M\,PFK}^{ADP} K_{M\,PFK}^{FBP} K_{PFK}^{eq}} \right)}{\Delta} \times \left(\frac{1 + \alpha . L . \left(\frac{\Delta'}{\Delta} \right)^3}{1 + L . \left(\frac{\Delta'}{\Delta} \right)^4} \right) \quad \text{(S85)}$$

$$\Delta = \left(1 + \frac{H6P}{K_{M\,PFK}^{H6P}} \right) . \left(1 + \frac{ATP}{K_{M\,PFK}^{ATP}} \right) + \frac{ADP}{K_{M\,PFK}^{ADP}} + \frac{FBP}{K_{M\,PFK}^{FBP}} \left(1 + \frac{ADP}{K_{M\,PFK}^{ADP}} \right)$$

$$\Delta' = \left(1 + \frac{H6P}{K_{M\,PFK}^{\prime H6P}} \right) . \left(1 + \frac{ATP}{K_{M\,PFK}^{\prime ATP}} \right) + \frac{ADP}{K_{M\,PFK}^{\prime ADP}} + \frac{FBP}{K_{M\,PFK}^{\prime FBP}} \left(1 + \frac{ADP}{K_{M\,PFK}^{\prime ADP}} \right)$$

$$\alpha = \frac{K_{M\,PFK}^{H6P} \; K_{M\,PFK}^{ATP}}{K_{M\,PFK}^{\prime H6P} \; K_{M\,PFK}^{\prime ATP}}$$

$$L = L_o \cdot \left[\left(\frac{1 + \frac{ATP}{K_{i\,PFK}^{ATP}}}{1 + d \, \frac{ATP}{K_{i\,PFK}^{ATP}}} \right) . \left(\frac{1 + e \, \frac{AMP}{K_{a\,PFK}^{AMP}}}{1 + \frac{AMP}{K_{a\,PFK}^{AMP}}} \right) . \left(\frac{1 + \frac{Cit_{cy}}{K_{i\,PFK}^{Cit}}}{1 + f \, \frac{Cit_{cy}}{K_{i\,PFK}^{Cit}}} \right) \right]^4$$

$$V_{\max,\mathrm{r}}^{PFK} = \frac{V_{\max,f}^{PFK} K_{M\,PFK}^{ADP} \; K_{M\,PFK}^{FBP}}{K_{M\,PFK}^{G6P} \; K_{M\,PFK}^{ATP}}$$

Aldolase (ALD)

$$V_{ALD} = V_{\max}^{ALD} \cdot \frac{\left(\frac{FBP}{K_{M\,ALD}^{FBP}} - \frac{(G3P)^2}{K_{M\,ALD}^{FBP} K_{ALD}^{eq}} \right)}{1 + \frac{FBP}{K_{M\,ALD}^{FBP}} + 2\frac{(G3P)}{K_{M\,ALD}^{G3P}}} \quad \text{(S86)}$$

Glyceraldehyde 3 phosphate dehydrogenase (GAPD)

$$V_{GAPD} = \frac{\left(V_{\max,f}^{GAPD} \frac{G3P \ . \ Pi}{K_{M\,GAPD}^{G3P} \ K_{M\,GAPD}^{Pi}} \left(\frac{(C_T^{Pyr} - NADH)}{K_{M\,GAPD}^{NAD}} \right)^{nGD} - V_{\max,r}^{GAPD} \frac{BPG}{K_{M\,GAPD}^{BPG}} \left(\frac{NADH}{K_{M\,GAPD}^{NADH}} \right)^{nGD} \right)}{\Delta_{GAPD}} \quad \text{(S87)}$$

$$V_{\max,r}^{GAPD} = \frac{V_{\max,f}^{GAPD} K_{M\,GAPD}^{NADH} \ K_{M\,GAPD}^{BPG}}{K_{M\,GAPD}^{G3P} \ K_{M\,GAPD}^{NAD} \ K_{M\,GAPD}^{Pi} \ K_{GAPD}^{eq}}$$

$$\Delta_{GAPD} = 1 + \frac{G3P}{K_{M\,GAPD}^{GAP}} + \left(\frac{(C_T^{Pyr} - NADH)}{K_{M\,GAPD}^{NAD}} \right)^{nGD} + \frac{Pi}{K_{M\,GAPD}^{Pi}} + \frac{G3P}{K_{M\,GAPD}^{GAP}} \left(\frac{(C_T^{Pyr} - NADH)}{K_{M\,GAPD}^{NAD}} \right)^{nGD}$$

$$\cdots + \frac{G3P \ . \ Pi}{K_{M\,GAPD}^{GAP} \ K_{M\,GAPD}^{Pi}} \left(\frac{(C_T^{Pyr} - NADH)}{K_{M\,GAPD}^{NAD}} \right)^{nGD} + \frac{BPG}{K_{M\,GAPD}^{BPG}} + \left(\frac{NADH}{K_{M\,GAPD}^{NADH}} \right)^{nGD} + \frac{BPG}{K_{M\,GAPD}^{BPG}} \left(\frac{NADH}{K_{M\,GAPD}^{NADH}} \right)^{nGD}$$

Phosphoglycerate kinase (PGK)

$$V_{PGK} = \frac{\left(V_{\max,f}^{PGK} \frac{BPG}{K_{M\,PGK}^{BPG}} \frac{(C_T^{ad} - ATP)}{K_{M\,PGK}^{ADP}} - V_{\max,r}^{PGK} \frac{3PG}{K_{M\,PGK}^{3PG}} \frac{ATP}{K_{M\,PGK}^{ATP}} \right)}{\Delta_{PGK}} \quad \text{(S88)}$$

$$V_{\max,r}^{PGK} = \frac{V_{\max,f}^{PGK} K_{M\,PGK}^{ATP} \ K_{M\,PGK}^{3PG}}{K_{M\,PGK}^{BPG} \ K_{M\,PGK}^{ADP} \ K_{PGK}^{eq}}$$

$$\Delta_{PGK} = 1 + \frac{BPG}{K_{M\,PGK}^{BPG}} + \frac{(C_T^{ad} - ATP)}{K_{M\,PGK}^{ADP}} + \frac{BPG}{K_{M\,PGK}^{BPG}} \frac{(C_T^{ad} - ATP)}{K_{M\,PGK}^{ADP}} +$$

$$\cdots + \frac{3PG}{K_{M\,PGK}^{3PG}} + \frac{ATP}{K_{M\,PGK}^{ATP}} + \frac{3PG}{K_{M\,PGK}^{3PG}} \frac{ATP}{K_{M\,PGK}^{ATP}}$$

Enolase (Enol)

$$V_{Enol}=\frac{\left(V_{\max,\mathrm{f}}^{Enol}\frac{3PG}{K_{M\,Enol}^{3PG}}-V_{\max,r}^{Enol}\frac{PEP}{K_{M\,Enol}^{PEP}}\right)}{1+\frac{3PG}{K_{M\,Enol}^{3PG}}+\frac{PEP}{K_{M\,Enol}^{PEP}}} \tag{S89}$$

$$V_{\max,\mathrm{r}}^{Enol}=\frac{V_{\max,f}^{Enol}K_{M\,Enol}^{PEP}}{K_{M\,Enol}^{3PG}\;K_{Enol}^{eq}}$$

Pyruvate kinase (PK)

$$V_{PK}=\frac{\left(V_{\max,\mathrm{f}}^{PK}\frac{\left(C_T^{ad}-ATP_i\right)\,.\,\mathrm{PEP}}{K_{M\,PK}^{ADP}.K_{M\,PK}^{PEP}}-V_{\max,r}^{PFK}\frac{ATP_i.Py}{K_{M\,PK}^{ATP}\,K_{M\,PK}^{Py}K_{PK}^{eq}}\right)\cdot\left(\frac{1}{1+L_{PK}}\right)}{1+\frac{\left(C_T^{ad}-ATP_i\right)}{K_{M\,PK}^{ADP}}+\frac{\mathrm{PEP}}{K_{M\,PK}^{PEP}}+\frac{\left(C_T^{ad}-ATP_i\right)\,.\,\mathrm{PEP}}{K_{M\,PK}^{ADP}.K_{M\,PK}^{PEP}}+\frac{ATP_i}{K_{M\,PK}^{ATP}}+\frac{Py}{K_{M\,PK}^{Py}}+\frac{ATP_i.Py}{K_{M\,PK}^{ATP}\,K_{M\,PK}^{Py}}} \tag{S90}$$

$$L_{PK}=\frac{\left(\frac{1.0\times10^{-6.8}}{1.0\times10^{-pH}}\right)}{\left(1+\frac{PEP}{K_{M\,PK}^{PEP}}+\frac{Py}{K_{M\,PK}^{Py}}\right)^4\left(1+\frac{FBP}{K_{M\,PK}^{FBP}}+\frac{GDP}{K_{M\,PK}^{GDP}}\right)^4}$$

$$V_{\max,\mathrm{r}}^{PK}=\frac{V_{\max,f}^{PK}K_{M\,PK}^{ATP}\;K_{M\,PK}^{Py}}{K_{M\,PK}^{PEP}\;K_{M\,PK}^{ADP}}$$

Lactate dehydrogenase (LDH)

$$V_{LDH}=\frac{\left(V_{\max,\mathrm{f}}^{LDH}\frac{NADH\,.\,\mathrm{Py}}{K_{M\,LDH}^{NADH}.K_{M\,LDH}^{Py}}-V_{\max,r}^{LDH}\frac{\left(C_T^{Pyr}-NADH\right).Lac}{K_{M\,LDH}^{NAD}K_{M\,LDH}^{Lac}K_{LDH}^{eq}}\right)}{1+\frac{NADH}{K_{M\,LDH}^{NADH}}+\frac{\mathrm{Py}}{K_{M\,LDH}^{Py}}+\frac{NADH\,.\,\mathrm{Py}}{K_{M\,LDH}^{NADH}.K_{M\,LDH}^{Py}}+\frac{\left(C_T^{Pyr}-NADH\right)}{K_{M\,LDH}^{NAD}}+\frac{Lac}{K_{M\,LDH}^{Lac}}+\frac{\left(C_T^{Pyr}-NADH\right).Lac}{K_{M\,LDH}^{NAD}K_{M\,LDH}^{Lac}}} \tag{S91}$$

$$V_{\max,\mathrm{r}}^{LDH}=\frac{V_{\max,f}^{LDH}K_{M\,LDH}^{NAD}\;K_{M\,LDH}^{Lac}}{K_{M\,LDH}^{Py}\;K_{M\,LDH}^{NADH}}$$

1.2.2 Supplementary Table S1. Parameters used in the glycolysis rate expressions

Symbol	Value	Units	Description	Reference
k_T^{Glc}	0.053	s^{-1}	plasmalemmal transport constant of glucose	(Cortassa et al., 2015)
$[Glc]_o$	10	mM	Concentration of extracellular glucose	(Cortassa et al., 2015)
$V_{\max}^{HK}$	10.4	mM s^{-1}	Maximal rate of HK	(Cortassa et al., 2015)
$K_{M\,HK}^{\;Glc}$	0.11	mM	Michaelis constant for glucose	(Lueck and Fromm, 1974)
$K_{M\,HK}^{\;ATP}$	0.17	mM	Michaelis constant of HK for ATP	(Lueck and Fromm, 1974)
$K_{M\,HK}^{\;G6P}$	1.4×10^{-3}	mM	Michaelis constant of HK for H6P	(Lueck and Fromm, 1974)
$K_{M\,HK}^{\;ADP}$	1.2	mM	Michaelis constant of HK for ADP	(Lueck and Fromm, 1974)
K_{HK}^{eq}	7800	-	Equilibrium constant for HK	(Lueck and Fromm, 1974)
$V_{\max,f}^{PFK}$	0.7	mM s^{-1}	Maximal rate of PFK	(Cortassa et al., 2015)
$K_{M\,PFK}^{\;H6P}$	0.18	mM	Michaelis constant of PFK for H6P	(Lambeth and Kushmerick, 2002)
$K_{M\,PFK}^{\;ATP}$	0.08	mM	Michaelis constant of PFK for ATP	(Lambeth and Kushmerick, 2002)
$K_{M\,PFK}^{\;FBP}$	4.02	mM	Michaelis constant of PFK for FBP	(Lambeth and Kushmerick, 2002)
$K_{M\,PFK}^{\;ADP}$	2.7	mM	Michaelis constant of PFK for ADP	(Lambeth and Kushmerick, 2002)
$K'^{\;H6P}_{M\;PFK}$	20	mM	Michaelis constant of PFK for H6P	(Lambeth and Kushmerick, 2002)
$K'^{\;ATP}_{M\;PFK}$	0.25	mM	Michaelis constant of PFK for ATP	(Lambeth and Kushmerick, 2002)
$K'^{\;FBP}_{M\;PFK}$	4.02	mM	Michaelis constant of PFK for FBP	(Lambeth and Kushmerick, 2002)
$K'^{\;ADP}_{M\;PFK}$	2.7	mM	Michaelis constant of PFK for ADP	(Lambeth and Kushmerick, 2002)
L_o	13	mM	Alosteric constant of PFK for ADP	(Lambeth and Kushmerick, 2002)
$K_{a\;PFK}^{\;AMP}$	0.015	mM	Activation constant of PFK for AMP	(Lambeth and Kushmerick, 2002)
$K_{i\;PFK}^{\;ATP}$	0.87	mM	Inhibition constant of PFK for ATP	(Lambeth and Kushmerick, 2002)
$K_{i\;PFK}^{\;Cit}$	0.03	mM	Inhibition constant of PFK for Cit	(Lambeth and Kushmerick, 2002)
d	0.01		Factor for ATP inhibition of PFK	(Lambeth and Kushmerick, 2002)
e	0.01		Factor for AMP activation of PFK	(Lambeth and Kushmerick, 2002)
f	0.01		Factor for citrate inhibition of PFK	(Lambeth and Kushmerick, 2002)

Symbol	Value	Units	Description	Reference
AMP	0.01	mM	Concentration of AMP	((Randle and Tubbs, 1979)
Cit_{cy}	0.01	mM	Cytoplasmic concentration of Citrate	(Randle and Tubbs, 1979)
K_{PFK}^{eq}	242.0		Equilibrium constant	(Lambeth and Kushmerick, 2002)
C_T^{ad}	14.0	mM	Total adenine nucleotides pool	(Randle and Tubbs, 1979)
$V_{\max}^{ALD}$	2.1	mM s^{-1}	Maximal rate of aldolase	(Cortassa et al., 2015)
$K_{M\,ALD}^{FBP}$	0.05	mM	Michaelis constant for FBP	(Lambeth and Kushmerick, 2002)
$K_{M\,ALD}^{FBP}$	0.05	mM	Michaelis constant for FBP	(Lambeth and Kushmerick, 2002)
$K_{M\,ALD}^{G3P}$	2.1	mM	Michaelis constant for GAP	(Lambeth and Kushmerick, 2002)
K_{ALD}^{eq}	0.12		Equilibrium constant	(Lambeth and Kushmerick, 2002)
$V_{\max,(f)(r)}^{GAPD}$	4.0	mM s^{-1}	Maximal rate of GAPD (forward or reverse)	(Cortassa et al., 2015)
$K_{M\,GAPD}^{NAD}$	9.0×10^{-3}	mM	Michaelis constant for NAD	(Lambeth and Kushmerick, 2002)
$K_{M\,GAPD}^{NADH}$	3.3×10^{-3}	mM	Michaelis constant for NADH	(Lambeth and Kushmerick, 2002)
$K_{M\,GAPD}^{GAP}$	2.5×10^{-3}	mM	Michaelis constant for GAP	(Lambeth and Kushmerick, 2002)
$K_{M\,GAPD}^{BPG}$	8.0×10^{-4}	mM	Michaelis constant for BPG	(Lambeth and Kushmerick, 2002)
$K_{M\,GAPD}^{Pi}$	0.29	mM	Michaelis constant for Pi	(Lambeth and Kushmerick, 2002)
Pi	3.0	mM	Concentration of phosphate	
nGD	0.64	mM	Cooperativity coefficient	(Lambeth and Kushmerick, 2002)
C_T^{Pyr}	1.0	mM	Total pyridine nucleotides pool	(Randle and Tubbs, 1979)
K_{GAPD}^{eq}	0.089		Equilibrium constant	(Lambeth and Kushmerick, 2002)
$V_{\max,(f)(r)}^{PGK}$	2.5	mM s^{-1}	Maximal rate of PGK	(Cortassa et al., 2015)
$K_{M\,PGK}^{BPG}$	2.2×10^{-3}	mM	Michaelis constant of PGK for BPG	(Lambeth and Kushmerick, 2002)
$K_{M\,PGK}^{3PG}$	1.2	mM	Michaelis constant of PGK for 3PG	(Lambeth and Kushmerick, 2002)
$K_{M\,PGK}^{ADP}$	8.0×10^{-4}	mM	Michaelis constant of PGK for ADP	(Lambeth and Kushmerick, 2002)
$K_{M\,PGK}^{ATP}$	0.35	mM	Michaelis constant of PGK for ATP	(Lambeth and Kushmerick, 2002)
K_{PGK}^{eq}	57109		Equilibrium constant	(Lambeth and Kushmerick, 2002)

Symbol	Value	Units	Description	Reference
$V_{max,(f)(r)}^{Enol}$	2.2	mM s^{-1}	Maximal rate of Enol	(Cortassa et al., 2015)
$K_{M\,Enol}^{3PG}$	0.12	mM	Michaelis constant for BPG	(Lambeth and Kushmerick, 2002)
$K_{M\,Enol}^{PEP}$	0.37	mM	Michaelis constant for 3PG	(Lambeth and Kushmerick, 2002)
K_{Enol}^{eq}	0.49		Equilibrium constant	(Lambeth and Kushmerick, 2002)
$V_{max,(f)(r)}^{PK}$	416	mM s^{-1}	Maximal rate of PK	(Cortassa et al., 2015)
$K_{M\,PK}^{PEP}$	0.08	mM	Michaelis constant for PEP	(Lambeth and Kushmerick, 2002)
$K_{M\,PK}^{Py}$	7.05	mM	Michaelis constant for Py	(Lambeth and Kushmerick, 2002)
$K_{M\,PK}^{ADP}$	0.3	mM	Michaelis constant for ADP	(Lambeth and Kushmerick, 2002)
$K_{M\,PK}^{ATP}$	1.13	mM	Michaelis constant for ATP	(Lambeth and Kushmerick, 2002)
$K_{M\,PK}^{FBP}$	0.005	mM	Michaelis constant for FBP	(Nishino et al., 2009)
$K_{M\,PK}^{GDP}$	0.1	mM	Michaelis constant for GDP	(Nishino et al., 2009)
GDP	0.1	mM	Concentration of GDP	(Nishino et al., 2009)
K_{PGK}^{eq}	10304		Equilibrium constant	(Lambeth and Kushmerick, 2002)
$V_{max,(f)(r)}^{LDH}$	5.4	mM s^{-1}	Maximal rate of LDH	(Cortassa et al., 2015)
$K_{M\,LDH}^{NADH}$	2.0×10^{-3}	mM	Michaelis constant for NADH	(Lambeth and Kushmerick, 2002)
$K_{M\,LDH}^{NAD}$	0.849	mM	Michaelis constant for NAD	(Lambeth and Kushmerick, 2002)
$K_{M\,LDH}^{Py}$	3.35×10^{-2}	mM	Michaelis constant for Py	(Lambeth and Kushmerick, 2002)
$K_{M\,LDH}^{Lac}$	17	mM	Michaelis constant for BPG	(Lambeth and Kushmerick, 2002)
K_{LDH}^{eq}	16198		Equilibrium constant	(Lambeth and Kushmerick, 2002)

1.2.3 Glycogenolysis, pentose phosphate and polyol pathways

Glycogen debranching enzyme (Gno_is)

$$V_{Gno\_is} = V_{max}^{Gno\_is} \cdot \frac{Glc_n}{Glc_n + K_{M\,Gno\_is}^{Gno}} \quad \text{(S92)}$$

Glycogen Phosphorylase (GP)

$$V_{GPa}=\frac{\left(V_{\max,f}^{GPa}\frac{Mal\ .\ \mathrm{Pi}}{K_{i\ GPa,f}^{Mal}.K_{M\ GPa}^{Pi}}-V_{\max,r}^{GPa}\frac{Mal.G1P}{K_{a\ GPa,b}^{Mal}K_{a\ GPa}^{G1P}}\right)}{1+\frac{Mal}{K_{i\ GPa,f}^{Mal}}+\frac{Pi}{K_{iGPa}^{Pi}}+\frac{Mal}{K_{a\ GPa,b}^{Mal}}+\frac{\mathrm{G}1P}{K_{a\ GPa}^{G1P}}+\frac{Mal}{K_{i\ GPa,f}^{Mal}}\frac{Pi}{K_{iGPa}^{Pi}}+\frac{Mal}{K_{i\ GPa,b}^{Mal}}\frac{\mathrm{G}1P}{K_{a\ GPa}^{G1P}}} \quad \text{(S93)}$$

$$V_{GPb}=\frac{\left(V_{\max,f}^{GPb}\frac{Mal\ .\ Pi}{K_{i\ GPb,f}^{Mal}.K_{M\ GPb}^{Pi}}-V_{\max,r}^{GPb}\frac{Mal.G1P}{K_{iGPb,b}^{Mal}K_{iGPb}^{G1P}}\right)}{1+\frac{Mal}{K_{i\ GPb,f}^{Mal}}+\frac{Pi}{K_{iGPb}^{Pi}}+\frac{Mal}{K_{i\ GPb,b}^{Mal}}+\frac{\mathrm{G}1P}{K_{iGPb}^{G1P}}+\frac{Mal}{K_{i\ GPb,f}^{Mal}}\frac{Pi}{K_{iGPb}^{Pi}}+\frac{Mal}{K_{i\ GPb,b}^{Mal}}\frac{\mathrm{G}1P}{K_{iGPb}^{G1P}}}\cdot\left(\frac{\frac{AMP^{nH}}{K_{M\ GPb}^{\prime AMP}}}{1+\frac{AMP^{nH}}{K_{M\ GPb}^{\prime AMP}}}\right) \quad \text{(S94)}$$

$$V_{\max,\mathrm{r}}^{GP(a)(b)}=\frac{V_{\max,f}^{GP(a)(b)}K_{iGP,b}^{Mal}\ K_{iGP}^{G1P}}{K_{iGP,f}^{Mal}\ K_{M\ GP}^{Pi}K_{GP}^{eq}}$$

Phosphoglucomutase (PGLM)

$$V_{PGLM}=V_{\max,\mathrm{f}}^{PGLM}\frac{\left(\frac{G1P}{K_{M\ PGLM}^{G1P}}-\frac{G6P}{K_{M\ PGLM}^{G1P}\ K_{PGLM}^{eq}}\right)}{1+\frac{G1P}{K_{M\ PGLM}^{G1P}}+\frac{G6P}{K_{M\ PGLM}^{G6P}}} \quad \text{(S95)}$$

Glucose 6 Phosphate dehydrogenase (G6PD)

$$V_{G6PD}=\frac{V_{\max}^{G6PD}\frac{H6P\ .\ (\mathrm{C}_T^{PyP}-\mathrm{NADPH})}{K_{M\ G6PD}^{H6P}.K_{M\,G6PD}^{NADP}}}{1+\frac{(\mathrm{C}_T^{PyP}-\mathrm{NADPH})}{K_{M\,G6PD}^{NADP}}\left(1+\frac{\mathrm{H6P}}{K_{M\ G6PD}^{H6P}}\right)+\frac{NADPH}{K_{M\,G6PD}^{NADPH}}+\frac{ATP}{K_{M\,G6PD}^{ATP}}} \quad \text{(S96)}$$

6 Phosphogluconate dehydrogenase (6PGO)

$$V_{6PGO}=E_{6PGO}\cdot\frac{\left(k_1k_3k_5k_7k_9(C_T^{PyP}-NADPH)\ .\ 6PG-\ k_2k_4k_6k_8k_{10}\ NADPH\ .\ Ru5P\ \right)}{\left(\begin{array}{l}k_2k_9(k_4k_6+k_5k_6+k_5k_7)+k_1k_9(k_4k_6+k_5k_6+k_5k_7)(C_T^{PyP}-NADPH)+k_3k_5k_7k_9.6PG\\ \ldots+k_2k_4k_6k_8\ Ru5P+k_2k_{10}(k_4k_6+k_5k_6+k_5k_7)NADPH+k_1k_3(k_5k_7+k_5k_9+k_6k_9\\ \ldots+k_7k_9)(C_T^{PyP}-NADPH).6PG+k_1k_4k_6k_8(C_T^{PyP}-NADPH).Ru5P\\ \ldots+k_3k_5k_7k_{10}.6PG.NADPH+k_8k_{10}(k_2k_4+k_2k_5+k_2k_6+k_4k_6).Ru5P.NADPH\\ \ldots+k_1k_3k_8(k_5+k_6)(C_T^{PyP}-NADPH).6PG.Ru5P+k_3k_8k_{10}(k_5+k_6).6PG.Ru5P.NADP\end{array}\right)} \quad \text{(S97)}$$

Ribose 5P Isomerase (R5PI)

$$V_{R5PI}=E_{R5PI}\frac{\left(\dfrac{\dfrac{k_3\,Ru5P}{(k_2+k_3)}}{k_1}-\dfrac{\dfrac{k_2R5P}{(k_2+k_3)}}{k_4}\right)}{1+\dfrac{Ru5P}{\dfrac{(k_2+k_3)}{k_1}}+\dfrac{R5P}{\dfrac{(k_2+k_3)}{k_4}}} \tag{S98}$$

Ribulose 5P Epimerase (Ru5PE)

$$V_{Ru5PE}=E_{Ru5PE}\frac{\left(\dfrac{\dfrac{k_3\,Ru5P}{(k_2+k_3)}}{k_1}-\dfrac{\dfrac{k_2X5P}{(k_2+k_3)}}{k_4}\right)}{1+\dfrac{Ru5P}{\dfrac{(k_2+k_3)}{k_1}}+\dfrac{X5P}{\dfrac{(k_2+k_3)}{k_4}}} \tag{S99}$$

Transketolase 1 (TK1)

$$V_{TK1}=E_{TK1}\cdot\frac{\left(k_1k_3k_5k_7.X5P\ .\ R5P-\ k_2k_4k_6k_8\ .G3P\ .\ S7P\ \right)}{\left(\begin{array}{l}k_1k_3(k_6+k_7).X5P+k_5k_7(k_2+k_3).R5P+k_2k_4(k_6+k_7)\,G3P\\ \ldots+k_6k_8\,(k_2+k_3)S7P+k_1k_5(k_3+k_7).X5P.R5P\\ \ldots+k_4k_8(k_2+k_6).G3P.S7P+k_5k_8(k_2+k_3).R5P.S7P\\ \ldots+k_1k_4(k_6+k_7).X5P.G3P\end{array}\right)} \tag{S100}$$

Transaldolase (TAL)

$$V_{TAL}=E_{TAL}\cdot\frac{\left(k_1k_3k_5k_7.G3P\ .\ S7P-\ k_2k_4k_6k_8\ .E4P\ .\ H6P\ \right)}{\left(\begin{array}{l}k_1k_3(k_6+k_7).S7P+k_5k_7(k_2+k_3).G3P+k_2k_4(k_6+k_7)\,E4P\\ \ldots+k_6k_8\,(k_2+k_3)H6P+k_1k_5(k_3+k_7).S7P.G3P+k_4k_8(k_2+k_6).E4P.H6P\\ \ldots+k_5k_8(k_2+k_3).S7P.H6P+k_1k_4(k_6+k_7).G3P.E4P\end{array}\right)} \tag{S101}$$

Transketolase 2 (TK2)

$$V_{TK2}=E_{TK2}\cdot\frac{\left(k_1k_3k_5k_7.X5P\ .\ E4P-\ k_2k_4k_6k_8\ .G3P\ .\ H6P\ \right)}{\left(\begin{array}{l}k_1k_3(k_6+k_7).X5P+k_5k_7(k_2+k_3).E4P+k_2k_4(k_6+k_7)\,G3P\\ \ldots+k_6k_8\,(k_2+k_3)H6P+k_1k_5(k_3+k_7).X5P.E4P\\ \ldots+k_4k_8(k_2+k_6).G3P.H6P+k_5k_8(k_2+k_3).X5P.H6P+k_1k_4(k_6+k_7).E4P.G3P\end{array}\right)} \tag{S102}$$

Aldose Reductase (ALDR)

$$V_{ALDR}=E_{ALDR}.\frac{\left(k_1.NADPH.Glc_i-\dfrac{k_2.(C_T^{PyP}-NADPH).Sor}{K_{ALDR}^{eq}}\right)}{V_{ALDR}^{DENOMINATOR}} \qquad (S103)$$

$$V_{ALDR}^{DENOMINATOR}=\left(\begin{array}{l} k_2k_{ia}k_b+k_2k_b.NADPH+k_2k_a\,Glc_i+\dfrac{k_1k_q}{K_{ALDR}^{eq}}Sor+\dfrac{k_1k_p}{K_{ALDR}^{eq}}(C_T^{PyP}-NADPH) \\ \quad ...+k_2.Glc_i.NADPH+\dfrac{k_1k_q}{k_{ia}K_{ALDR}^{eq}}Sor.(C_T^{PyP}-NADPH) \\ \quad ...+\dfrac{k_2k_a}{k_{iq}}.Glc_i.(C_T^{PyP}-NADPH)+\dfrac{k_1}{K_{ALDR}^{eq}}\mathrm{Sor}.NADPH \\ \quad ...+\dfrac{k_2}{k_{ip}}.Glc_i.NADPH.Sor+\dfrac{k_1}{k_{ib}K_{ALDR}^{eq}}Glc_i.(C_T^{PyP}-NADPH).Sor \end{array}\right)$$

$$V_{ALDR}^{eq}=\frac{k_1k_pk_{iq}}{k_2\,k_bk_{ia}}$$

Sorbitol dehydrogenase (SoDH)

$$V_{SoDH}=V_{\max,f}^{SoDH}\frac{\left(C_T^{Pyr}-NADH\right).Sor}{K_{i\,SoDH}^{NAD}.K_{M\,SoDH}^{Sor}+K_{M\,SoDH}^{Sor}\left(C_T^{Pyr}-NADH\right)+K_{M\,SoDH}^{NAD}.Sor+\left(C_T^{Pyr}-NADH\right).Sor} \qquad (S104)$$
$$\ldots-V_{\max,r}^{SoDH}\frac{NADH.Fru}{K_{i\,SoDH}^{NADH}.K_{M\,SoDH}^{Fru}+K_{M\,SoDH}^{Fru}.NADH+K_{M\,SoDH}^{NADH}.Fru+NADH.Fru}$$

$$V_{\max,r}^{SoDH}=\frac{V_{\max,f}^{SoDH}K_{M\,SoDH}^{Fru}\,K_{M\,SoDH}^{NADH}}{K_{M\,SoDH}^{Sor}\,K_{M\,SoDH}^{NAD}\,K_{SoDH}^{eq}}$$

Xylitol dehydrogenase (XyDH)

$$V_{XyDH}=V_{\max,f}^{XDH}\frac{NADH\,.X5P}{K_{i\,XDH}^{NADH}.K_{M\,XDH}^{Xy}+K_{M\,XDH}^{Xy}.NADH+K_{M\,XDH}^{NADH}.X5P+NADH.X5P}$$
$$\ldots-V_{\max,r}^{XDH}\frac{\left(C_T^{Pyr}-NADH\right).XyOH}{\left(\begin{array}{l}K_{i\,XDH}^{NAD}.K_{M\,XDH}^{XyOH}+K_{M\,XDH}^{XyOH}.\left(C_T^{Pyr}-NADH\right)+K_{M\,XDH}^{NAD}.XyOH \\ \quad\ldots+\left(C_T^{Pyr}-NADH\right).XyOH\end{array}\right)} \qquad (S105)$$

$$V_{\max,r}^{XDH}=\frac{V_{\max,f}^{XDH}K_{M\,XDH}^{XyOH}\,K_{M\,XDH}^{NAD}}{K_{M\,XDH}^{Xy}\,K_{M\,XDH}^{NADH}\,K_{XDH}^{eq}}$$

1.2.4 Supplementary Table S2. Parameter values used in the glycogenolysis, pentose phosphate and polyol pathway rate expressions

Symbol	Value	Units	Description	Reference
$V_{\max}^{Gno\_is}$	0.038	mM s^{-1}	Maximal rate of glycogen breakdown	(Lambeth and Kushmerick, 2002)
$K_{M\,Gno\_is}^{Gno}$	5.0	mM	Michaelis constant for Glycogen	(Lambeth and Kushmerick, 2002)
Glc_n	110	mM	Glycogen levels cardiac muscle	(Cortassa et al., 2015)
$V_{\max,f}^{PGa}$	2.0	mM s^{-1}	Maximal rate of GPa, forward	(Cortassa et al., 2015)
$V_{\max,f}^{PGb}$	1.25	mM s^{-1}	Maximal rate of GPb, forward	(Cortassa et al., 2015)
$K_{iGPa,f}^{Mal}$	2.0	mM	Michaelis constant for Malt	(Lambeth and Kushmerick, 2002)
$K_{a\,GPa,b}^{Mal}$	1.7	mM	Michaelis constant for Malt	(Lambeth and Kushmerick, 2002)
$K_{a\,GPa}^{G1P}$	10.1	mM	Michaelis constant for ADP	(Lambeth and Kushmerick, 2002)
$K_{iGPa,b}^{Mal}$	0.15	mM	Michaelis constant for ATP	(Lambeth and Kushmerick, 2002)
$K_{M\,GPa}^{Pi}$	0.4	mM	Michaelis constant for FBP	(Lambeth and Kushmerick, 2002)
K_{iGPa}^{Pi}	4.7	mM	Michaelis constant for GDP	(Lambeth and Kushmerick, 2002)
$K_{iGPb,f}^{Mal}$	15	mM	Michaelis constant for Mal	(Lambeth and Kushmerick, 2002)
K_{iGPb}^{G1P}	7.4	mM	Michaelis constant for ADP	(Lambeth and Kushmerick, 2002)
$K_{iGPb,b}^{Mal}$	4.4	mM	Michaelis constant for ATP	(Lambeth and Kushmerick, 2002)
$K_{M\,GPb}^{Pi}$	0.02	mM	Michaelis constant for FBP	(Lambeth and Kushmerick, 2002)
K_{iGPb}^{Pi}	4.6	mM	Michaelis constant for GDP	(Lambeth and Kushmerick, 2002)
$K'^{\,AMP}_{M\;GPb}$	1.9×10^{-6}	mM	Activation constant of GPb for AMP	(Lambeth and Kushmerick, 2002)
nH	1.75		Allosteric coefficient for AMP	(Lambeth and Kushmerick, 2002)
K_{GP}^{eq}	0.42		Equilibrium constant	(Lambeth and Kushmerick, 2002)
$V_{\max,f}^{PGLM}$	0.14	mM s^{-1}	Maximal rate of PGLM	(Cortassa et al., 2015)
$K_{M\,PGLM}^{G1P}$	0.063	mM	Michaelis constant for G1P	(Lambeth and Kushmerick, 2002)
$K_{M\,PGLM}^{G6P}$	0.03	mM	Michaelis constant for G6P	(Lambeth and Kushmerick, 2002)

Symbol	Value	Units	Description	Reference
K_{PGLM}^{eq}	16.62		Equilibrium constant	(Lambeth and Kushmerick, 2002)
$V_{\max}^{G6PD}$	0.004	mM s^{-1}	Maximal rate of G6DH (forward or reverse)	(Cortassa et al., 2015)
$K_{M\,G6PD}^{H6P}$	6.67×10^{-3}	mM	Michaelis constant for H6P	(Lambeth and Kushmerick, 2002)
$K_{M\,G6PD}^{NADP}$	3.67×10^{-3}	mM	Michaelis constant for NADP	(Lambeth and Kushmerick, 2002)
$K_{M\,G6PD}^{NADPH}$	3.12×10^{-3}	mM	Michaelis constant for NADPH	(Lambeth and Kushmerick, 2002)
C_T^{PyP}	0.05	mM	Total phosphopyridine nucleotides pool	(Schafer and Buettner, 2001)
$K_{M\,G6PD}^{ATP}$	0.749	mM	Michaelis constant for BPG	(Lambeth and Kushmerick, 2002)
E_{6PGO}	0.01	mM	Maximal concentration of 6PGO	(Cortassa et al., 2015)
k_1	2.4×10^3	s^{-1}	6PGO rate constant	(McIntyre et al., 1989)
k_2	4.1×10^2	s^{-1}	6PGO rate constant	(McIntyre et al., 1989)
k_3	2.0×10^6	$mM^{-1}s^{-1}$	6PGO rate constant	(McIntyre et al., 1989)
k_4	26	$mM^{-1}s^{-1}$	6PGO rate constant	(McIntyre et al., 1989)
k_5	48.0	s^{-1}	6PGO rate constant	(McIntyre et al., 1989)
k_6	30.0	s^{-1}	6PGO rate constant	(McIntyre et al., 1989)
k_7	6.3×10^2	s^{-1}	6PGO rate constant	(McIntyre et al., 1989)
k_8	36	$mM^{-1}s^{-1}$	6PGO rate constant	(McIntyre et al., 1989)
k_9	8.0×10^2	s^{-1}	6PGO rate constant	(McIntyre et al., 1989)
k_{10}	2.25×10^2	$mM^{-1}s^{-1}$	6PGO rate constant	(McIntyre et al., 1989)
E_{R5PI}	0.0036	mM	Maximal concentration of R5PI	(Cortassa et al., 2015)
k_1	60.9	$mM^{-1}s^{-1}$	R5PI rate constant	(McIntyre et al., 1989)
k_2	33.3	s^{-1}	R5PI rate constant	(McIntyre et al., 1989)
k_3	14.2	$mM^{-1}s^{-1}$	R5PI rate constant	(McIntyre et al., 1989)
k_4	21.6	$mM^{-1}s^{-1}$	R5PI rate constant	(McIntyre et al., 1989)
E_{Ru5PE}	0.034	mM	Maximal concentration of Ru5PE	(Cortassa et al., 2015)

Symbol	Value	Units	Description	Reference
k_1	3.91×10^3	$mM^{-1}s^{-1}$	Ru5PE rate constant	(McIntyre et al., 1989)
k_2	4.38×10^2	s^{-1}	Ru5PE rate constant	(McIntyre et al., 1989)
k_3	3.05×10^2	s^{-1}	Ru5PE rate constant	(McIntyre et al., 1989)
k_4	1.49×10^3	$mM^{-1}s^{-1}$	Ru5PE rate constant	(McIntyre et al., 1989)
E_{TK1}	0.0027	mM	Maximal concentration of TK1	(Cortassa et al., 2015)
k_1	2.16×10^2	$mM^{-1}s^{-1}$	TK1 rate constant	(McIntyre et al., 1989)
k_2	38.0	s^{-1}	TK1 rate constant	(McIntyre et al., 1989)
k_3	34.0	s^{-1}	TK1 rate constant	(McIntyre et al., 1989)
k_4	1.56×10^2	$mM^{-1}s^{-1}$	TK1 rate constant	(McIntyre et al., 1989)
k_5	3.29×10^2	$mM^{-1}s^{-1}$	TK1 rate constant	(McIntyre et al., 1989)
k_6	1.75×10^2	s^{-1}	TK1 rate constant	(McIntyre et al., 1989)
k_7	40	s^{-1}	TK1 rate constant	(McIntyre et al., 1989)
k_8	44.8	$mM^{-1}s^{-1}$	TK1 rate constant	(McIntyre et al., 1989)
E_{TAL}	0.058	mM	Maximal concentration of TAL	(Cortassa et al., 2015)
k_1	2.16×10^2	$mM^{-1}s^{-1}$	TAL rate constant	(McIntyre et al., 1989)
k_2	4.53	s^{-1}	TAL rate constant	(McIntyre et al., 1989)
k_3	16.3	s^{-1}	TAL rate constant	(McIntyre et al., 1989)
k_4	30.0	$mM^{-1}s^{-1}$	TAL rate constant	(McIntyre et al., 1989)
k_5	4.9×10^2	$mM^{-1}s^{-1}$	TAL rate constant	(McIntyre et al., 1989)
k_6	60.0	s^{-1}	TAL rate constant	(McIntyre et al., 1989)
k_7	17.0	s^{-1}	TAL rate constant	(McIntyre et al., 1989)
k_8	79.0	$mM^{-1}s^{-1}$	TAL rate constant	(McIntyre et al., 1989)
E_{TK2}	0.005	mM	Maximal concentration of TK2	(Cortassa et al., 2015)
k_1	2.16×10^2	$mM^{-1}s^{-1}$	TK2 rate constant	(McIntyre et al., 1989)
k_2	38.0	s^{-1}	TK2 rate constant	(McIntyre et al., 1989)

Symbol	Value	Units	Description	Reference
k_3	34.0	s^{-1}	TK2 rate constant	(McIntyre et al., 1989)
k_4	1.56×10^{2}	$mM^{-1}s^{-1}$	TK2 rate constant	(McIntyre et al., 1989)
k_5	3.29×10^{2}	$mM^{-1}s^{-1}$	TK2 rate constant	(McIntyre et al., 1989)
k_6	1.75×10^{2}	s^{-1}	TK2 rate constant	(McIntyre et al., 1989)
k_7	40	s^{-1}	TK2 rate constant	(McIntyre et al., 1989)
k_8	44.8	$mM^{-1}s^{-1}$	TK2 rate constant	(McIntyre et al., 1989)
E_{ALDR}	3.4×10^{-5}	mM	Maximal concentration of ALDR	(Cortassa et al., 2015)
k_1	0.33	s^{-1}	ALDR rate constant	(Kubiseski et al., 1992)
k_2	0.037	s^{-1}	ALDR rate constant	(Kubiseski et al., 1992)
k_a	6.9×10^{-4}	mM	ALDR Michaelis constant for NADPH	(Kubiseski et al., 1992)
k_b	46	mM	ALDR Michaelis constant for Glc	(Kubiseski et al., 1992)
k_p	3.8×10^{2}	mM	ALDR Michaelis constant for Sor	(Kubiseski et al., 1992)
k_q	1.5×10^{-2}	mM	ALDR Michaelis constant for NADP	(Kubiseski et al., 1992)
k_{ia}	4.1×10^{-4}	mM	ALDR dissociation constant for NADPH	(Kubiseski et al., 1992)
k_{ib}	9.2×10^{-2}	mM	ALDR dissociation constant for Glc	(Kubiseski et al., 1992)
k_{ip}	1.3×10^{3}	mM	ALDR dissociation constant for Sor	(Kubiseski et al., 1992)
k_{iq}	8.3×10^{-3}	mM	ALDR dissociation constant for NADP	(Kubiseski et al., 1992)
$V_{\max,f}^{SoDH}$	6.0×10^{-4}	$mM\ s^{-1}$	Maximal concentration of SoDH	(Cortassa et al., 2015)
$K_{M\,SoDH}^{NADH}$	1.0×10^{-2}	mM	SoDH Michaelis constant for NADH	(Karacaoglan and Ozer, 2005)
$K_{M\,SoDH}^{Fru}$	1.0×10^{3}	mM	SoDH Michaelis constant for Fru	(Karacaoglan and Ozer, 2005)
$K_{M\,SoDH}^{Sor}$	9.6	mM	SoDH Michaelis constant for Sor	(Karacaoglan and Ozer, 2005)
$K_{M\,SoDH}^{NAD}$	2.0	mM	SoDH Michaelis constant for NAD	(Karacaoglan and Ozer, 2005)
$K_{i\,SoDH}^{NADH}$	1.0×10^{-2}	mM	SoDH dissociation constant for NADH	(Karacaoglan and Ozer, 2005)
$K_{i\,SoDH}^{NAD}$	2.0	mM	SoDH dissociation constant for NAD	(Karacaoglan and Ozer, 2005)
K_{SoDH}^{eq}	9.25×10^{-2}		SoDH equilibrium constant	(Karacaoglan and Ozer, 2005)

Symbol	Value	Units	Description	Reference
$V_{\max,f}^{XDH}$	0.3	mM s^{-1}	Maximal concentration of XDH	(Cortassa et al., 2015)
$K_{M\,XDH}^{NADH}$	1.0×10^{-2}	mM	XDH Michaelis constant for NADH	(Karacaoglan and Ozer, 2005)
$K_{M\,XDH}^{XyOH}$	8.08×10^{-1}	mM	XDH Michaelis constant for XyOH	(O'Brien et al., 1983)
$K_{M\,XDH}^{Xy}$	20.0	mM	XDH Michaelis constant for X5P	(O'Brien et al., 1983)
$K_{M\,XDH}^{NAD}$	2.0	mM	XDH Michaelis constant for NAD	(Karacaoglan and Ozer, 2005)
$K_{i\,XDH}^{NADH}$	1.0×10^{-2}	mM	XDH dissociation constant for NADH	(Karacaoglan and Ozer, 2005)
$K_{i\,XDH}^{NAD}$	2.0	mM	XDH dissociation constant for NAD	(Karacaoglan and Ozer, 2005)
K_{XDH}^{eq}	0.24		XDH equilibrium constant	(Karacaoglan and Ozer, 2005)

2.5.5. ATPase, non-glycolytic NADH consumption, NADPH consumption, polyol transport

The rate expressions corresponding to NADH, NADPH and ATP consumption as well as polyol transport have been added to the present computational model to enable steady state behavior. Standard hyperbolic relations were assumed for each of the rate expressions.

$$V_{ATPase} = V_{\max}^{ATPase} \frac{ATP}{K_{M\,ATPase}^{ATP} + ATP} \quad \text{(S106)}$$

$$V_{NADHc} = V_{\max}^{NADHc} \cdot \frac{\dfrac{NADH}{K_{M\,NADHc}^{NADH}} - \dfrac{(C_T^{Pyr} - NADH)}{K_{M\,NADHc}^{NAD}}}{1 + \dfrac{NADH}{K_{M\,NADHc}^{NADH}} + \dfrac{(C_T^{Pyr} - NADH)}{K_{M\,NADHc}^{NAD}}} \quad \text{(S107)}$$

$$V_{NADPHc} = V_{\max}^{NADPHc} \cdot \frac{\dfrac{NADPH}{K_{M\,NADPHc}^{NADPH}} - \dfrac{(C_T^{PyP} - NADPH)}{K_{M\,NAPDHc}^{NADP}}}{1 + \dfrac{NADPH}{K_{M\,NADPHc}^{NADPH}} + \dfrac{(C_T^{PyP} - NADPH)}{K_{M\,NADPHc}^{NADP}}} \quad \text{(S108)}$$

$$V_{FruT} = k_{FruT} \frac{Fru}{K_{FruT}^{Fru} + Fru} \quad \text{(S109)}$$

$$V_{XyOHT} = k_{XyOHT} \frac{XyOH}{K_{XyOHT}^{XyOH} + XyOH} \quad \text{(S110)}$$

1.2.5 Supplementary Table S3. Parameters of ATP hydrolysis, NAD(P)H consumption and polyol transport reactions

Symbol	Value	Units	Description
$V_{\max}^{ATPase}$	3.0	mM s^{-1}	Maximal Rate of ATPase
$K_{M\ ATPase}^{ATP}$	9.0	mM	Michaelis constant for ATP
$V_{\max}^{NADHc}$	0.2	mM s^{-1}	Maximal rate of NADH consumption
$K_{M\ NADHc}^{NADH}$	0.05	mM	Michaelis constant for NADH
$K_{M\ NADHc}^{NAD}$	0.7	mM	Michaelis constant for NAD
$V_{\max}^{NADPHc}$	320	mM s^{-1}	Maximal rate of NADPH consumption
$K_{M\ NADPHc}^{NADPH}$	2.5×10^{-2}	mM	Michaelis constant for NADPH
$K_{M\ NADPHc}^{NADP}$	7.5×10^{-3}	mM	Michaelis constant for NADP
k_{FruT}	0.001	mM s^{-1}	Rate constant of Fru transport
K_{FruT}^{Fru}	3.0	mM	Michaelis constant for Fru
k_{XyOHT}	0.005	mM s^{-1}	Rate constant of XyOH transport
K_{XyOHT}^{XyOH}	1.5×10^{-5}	mM	Michaelis constant for XyOH

1.2.6 Rate equations of mitochondrial energy metabolism

The equations and parameters reproduced in this and the following sections are from our previous work (Wei et al., 2011; Kembro et al., 2013a; Cortassa et al., 2017a; 2017b). Details about the model parameterization are based on the original publications above mentioned.

Pyruvate carrier (PyrC)

$$V_{PyrC} = \frac{1}{1+\dfrac{[H^+]_m}{k_{h,1pc}}} \frac{V_{\max}^{PC}[Pyr]_c}{[Pyr]_c + K_{M\_PC}^{Pyr}} \tag{S111}$$

Pyruvate dehydrogenase (PDH)

$$V_{PDH} = \frac{V_{PDH}^{\max}\,\alpha_{PDH}\left(\frac{NAD^{+}}{K_{M\_PDH}^{NAD}}\right)\left(\frac{Pyr}{K_{M\_PDH}^{Pyr}}\right)\left(\frac{CoA}{K_{M\_PDH}^{CoA}}\right)}{\left(1+\frac{Pyr}{K_{M\_PDH}^{Pyr}}\right)\left(\frac{1+\frac{CoA}{K_{M\_PDH}^{CoA}}+\frac{CoA_{T}}{K_{D\_PDH}^{CoA}}\left(1+\frac{CoA}{CoA_{T}}\right)}{1+\frac{NAD^{+}}{K_{M\_PDH}^{NAD}}+\frac{NAD_{T}}{K_{D\_PDH}^{NAD}}\left(1+\frac{NAD^{+}}{NAD_{T}}\right)}\right)} \qquad \text{(S112)}$$

$$\alpha_{PDH} = \frac{\left(1+\frac{Ca_{m}^{2+}}{K_{DP}^{Ca}}\right)\left(\left(\frac{NAD^{+}}{NADH}\right)^{n\_NAD} F_{DP\_NAD}+1\right)}{\left(\begin{array}{l}\left(1+\frac{Ca_{m}^{2+}}{K_{DP}^{Ca}}\right)\left(\left(\frac{NAD^{+}}{NADH}\right)^{n\_NAD} F_{DP\_NAD}+1\right)\\ \ldots+\left(1+\frac{ATP}{K_{PDK}^{ATP}}\right)\left(1+\frac{K_{PDK}^{ADP}}{ADP}\right)\left(1+\frac{K_{PDK}^{Pyr}}{Pyr}\right)\left(\left(\frac{AcCoA}{CoA}\right)^{n\_CoA} F_{PDK\_AcCoA}+1\right)\end{array}\right)}$$

Pyruvate carboxylase (PyrCb)

$$V_{PCb} = \frac{1}{1+\frac{[H^{+}]_{m}}{k_{h,1p}}+\frac{k_{h,2p}}{[H^{+}]_{m}}}\,\frac{V_{\max}^{PCb}[Pyr]_{m}.[MgATP^{-2}]_{m}}{K_{M\_PCb}^{Pyr}[MgATP^{-2}]+[Pyr]_{m}.K_{M\_PCb}^{MgATP}+[Pyr]_{m}.[MgATP^{-2}]} \qquad \text{(S113)}$$

Citrate synthase (CS)

$$\mathrm{V_{CS}} = \frac{\mathrm{k_{cat}^{CS}\ E_{T}^{CS}}}{\left(1+\frac{\mathrm{K_{M}^{AcCoA}}}{\mathrm{[AcCoA]}}\right)\left(1+\frac{\mathrm{K_{M}^{OAA}}}{\mathrm{[OAA]}}\right)} \qquad \text{(S114)}$$

Aconitase (ACO)

$$\mathrm{V_{ACO}} = \mathrm{k_{f}^{ACO}}\left(\mathrm{[CIT]} - \frac{\mathrm{[ISOC]}}{\mathrm{K_{E}^{ACO}}}\right) \qquad \text{(S115)}$$

Isocitrate dehydrogenase (IDH)

$$V_{IDH} = k_{cat}^{IDH}\,E_{T}^{IDH}\left[\begin{array}{l}\left(1+\frac{[H^{+}]_{m}}{k_{h,1}}+\frac{k_{h,2}}{[H^{+}]_{m}}\right)+f_{i}^{IDH}\left(\frac{K_{Midh}^{NAD}}{[NAD]}\right)+\ldots\\ f_{a}^{IDH}\left(\frac{K_{M}^{ISOC}}{[ISOC]}\right)^{ni}+f_{a}^{IDH}f_{i}^{IDH}\left(\frac{K_{M}^{ISOC}}{[ISOC]}\right)^{ni}\left(\frac{K_{Midh}^{NAD}}{[NAD]}\right)\end{array}\right]^{-1} \qquad \text{(S116)}$$

$$f_a^{IDH} = \left[\left(1+\frac{[ADP^{3-}]_m}{K_{ADP}^a}\right)\left(1+\frac{[Ca^{2+}]_m}{K_{Ca}^a}\right)\right]^{-1}$$

$$f_i^{IDH} = \left(1+\frac{[NADH]}{K_{i,NADH}}\right)$$

***α*-ketoglutarate dehydrogenase (KGDH)**

$$V_{KGDH} = \frac{k_{cat}^{KGDH} E_T^{KGDH}}{1+\frac{[H^+]_m}{k_{h,1a}}+\frac{k_{h,2a}}{[H^+]_m}+f_a^{KGDH}\left(\frac{k_M^{\alpha KG}}{[\alpha KG]}\right)^{n_{\alpha KG}}+f_a^{KGDH}\frac{k_{M\_kgdh}^{NAD}}{[NAD]}} \quad \text{(S117)}$$

$$f_a^{KGDH} = \left[\left(1+\frac{[Mg^{2+}]}{K_D^{Mg^{2+}}}\right)\left(1+\frac{[Ca^{2+}]_m}{K_D^{Ca^{2+}}}\right)\right]^{-1}$$

Succinate lyase (SL)

$$V_{SL} = k_f^{SL}\left([SCoA][ADP]_m[Pi]_m - \frac{[Suc][ATP]_m[CoA]}{K_{E,app}^{SL}}\right) \quad \text{(S118)}$$

$$K_{E,app}^{SL} = K_{Eq}^{SL}\frac{P_{SUC}P_{ATP}}{P_{Pi}P_{ADP}}$$

Succinate dehydrogenase (SDH, CII_SDH))

$$V_{O_2SDH} = 0.5\rho^{res(SDH)}\frac{\left(r_a + r_{c1}e^{\left(\frac{4F\Delta\Psi_B}{RT}\right)}\right)e^{\left(\frac{FA_{RSDH}}{RT}\right)} - r_a e^{\left(\frac{g4F\Delta\mu_H}{RT}\right)} + r_{c2}e^{\left(\frac{FA_{RSDH}}{RT}\right)}e^{\left(\frac{g4F\Delta\mu_H}{RT}\right)}}{\left(1+r_1 e^{\left(\frac{FA_{RSDH}}{RT}\right)}\right)e^{\left(\frac{4F\Delta\Psi_B}{RT}\right)}+\left(r_2+r_3 e^{\left(\frac{FA_{RSDH}}{RT}\right)}\right)e^{\left(\frac{g4F\Delta\mu_H}{RT}\right)}}\left(\frac{1}{1+\frac{[OAA]}{K_i^{OAA}}}\right) \quad \text{(S119)}$$

$$A_{RSDH} = \frac{RT}{F}\ln\left(K_{RSDH,app}\sqrt{\frac{[SUC]}{[FUM]}}\right)$$

$$K_{RSDH,app} = \frac{K_{res(SDH)}}{P_{SUC}}$$

Fumarate hydratase (FH)

$$V_{FH} = k_f^{FH}\left([FUM] - \frac{[MAL]}{K_E^{FH}}\right) \qquad \text{(S120)}$$

Malate dehydrogenase (MDH)

$$V_{MDH} = \frac{k_{cat}^{MDH} E_T^{MDH} f_{h,a} f_{h,i}}{1 + \frac{K_M^{MAL}}{[MAL]}\left(1 + \frac{[OAA]}{K_i^{OAA}}\right) + \frac{K_M^{NAD}}{[NAD]} + \frac{K_M^{MAL}}{[MAL]}\left(1 + \frac{[OAA]}{K_i^{OAA}}\right)\frac{K_M^{NAD}}{[NAD]}} \qquad \text{(S121)}$$

$$f_{h,a} = \left(1 + \frac{[H^+]}{k_{h1}} + \frac{[H^+]^2}{k_{h1}k_{h2}}\right)^{-1} + k_{offset}$$

$$f_{h,i} = \left(1 + \frac{k_{h3}}{[H^+]} + \frac{k_{h3}k_{h4}}{[H^+]^2}\right)$$

Aspartate amino transferase (AAT)

$$V_{AAT} = k_f^{AAT}[OAA][GLU]\frac{k_{ASP}K_E^{ATT}}{\left(k_{ASP}K_E^{AAT} + [\alpha KG]k_f^{AAT}\right)} \qquad \text{(122)}$$

1.2.7 Supplementary Table S4. Parameter values of the tricarboxylic acid cycle

Symbol	Value	Units	Description	References
$V_{\max}^{PC}$	0.4125	mM s-1	Pyruvate carrier (PC) maximal rate	(Cortassa et al., 2017b)(Cortassa et al., 2017b)
$K_{M\_PC}^{Pyr}$	0.15	mM	PC Michaelis constant for Pyr	(Halestrap, 1978)
$k_{h,1pc}$	7.5×10^{-6}		PC ionization constant	(Halestrap, 1978)
$V_{PDH}^{\max}$	7.5	mM s-1	PDH maximal rate	(Cortassa et al., 2017b)
$K_{M\_PDH}^{NAD}$	0.05	mM	Michaelis constant (K_M) of PDH for NAD	(Pettit et al., 1975)
$K_{M\_PDH}^{Pyr}$	0.1	mM	K_M of PDH for pyruvate	(Cortassa et al., 2017b)
$K_{M\_PDH}^{CoA}$	0.006	mM	K_M of PDH for Coenzyme A (CoA)	(Pettit et al., 1975)

Symbol	Value	Units	Description	References
$K_{D\_PDH}^{CoA}$	0.03	mM	Dissociation constant for CoA	(Pettit et al., 1975)
$K_{D\_PDH}^{NAD}$	0.04	mM	Dissociation constant for NAD	(Pettit et al., 1975)
K_{DP}^{Ca}	0.001	mM	Activation constant of PDP for Ca2+	(Cortassa et al., 2017b)
K_{PDK}^{ATP}	0.2	mM	Activation constant of PDK for ATP	(Cortassa et al., 2017b)
K_{PDK}^{ADP}	0.05	mM	Inhibition constant of PDK for ADP	(Cortassa et al., 2017b)
K_{PDK}^{Pyr}	0.1	mM	Inhibition constant of PDK for pyruvate	(Cortassa et al., 2017b)
F_{DP_NAD}	6.7	-	NADH inhibitory factor of PDP	(Cortassa et al., 2017b)
F_{PDK_AcCoA}	5.3	-	AcCoA activation factor of PDK	(Cortassa et al., 2017b)
n_NAD	0.2	-	Exponential coefficient of NADH/NAD ratio	(Cortassa et al., 2017b)
n_CoA	0.6	-	Exponential coefficient of AcCoA/CoA ratio	(Cortassa et al., 2017b)
$V_{\max}^{PCb}$	0.5	mM.s-1	Maximal rate of Pyr carboxylase (PyrCb)	
$K_{M\_PCb}^{Pyr}$	0.23	mM	PyrCb Michaelis constant for Pyr	(Rowan et al., 1978)
$K_{M\_PCb}^{MgATP}$	0.25	mM	PyrCb Michaelis constant for MgATP	(Rowan et al., 1978)
$k_{h,1p}$	2.51×10^{-5}	mM	PyrCb ionization constant	(Rowan et al., 1978)
$k_{h,2p}$	1.26×10^{-5}	mM	PyrCb ionization constant	(Rowan et al., 1978)
k_{cat}^{CS}	2.35×10^{-4}	ms^{-1}	Catalytic constant of citrate synthase (CS)	(Cortassa et al., 2006)
E_{T}^{CS}	0.4	mM	CS concentration	(Cortassa et al., 2006)
K_{M}^{AcCoA}	0.0126	mM	CS Michaelis constant for AcCoA	(Cortassa et al., 2006)
K_{M}^{OAA}	6.4×10^{-4}	mM	CS Michaelis constant for OAA	(Cortassa et al., 2006)
$C_{k\,\mathrm{int}}$	1.3	mM	Sum of TCA cycle intermediates	(Cortassa et al., 2006)
C_{PN}	1.0	mM	Sum of pyridine nucleotides	(Cortassa et al., 2006)
k_{f}^{ACO}	1.17×10^{-4}	ms^{-1}	Forward rate constant of aconitase (ACO)	(Cortassa et al., 2006)
K_{E}^{ACO}	2.22		ACO equilibrium constant	(Cortassa et al., 2006)
$K_{i,NADH}$	0.19	mM	Inhibition constant by NADH	(Cortassa et al., 2006)
k_{cat}^{IDH}	1.188	ms^{-1}	Rate constant of isocitrate dehydrogenase (IDH)	(Cortassa et al., 2006)

Symbol	Value	Units	Description	References
E_T^{IDH}	0.109	mM	IDH concentration	(Cortassa et al., 2006)
$k_{h,1}$	1×10^{-5}	mM	IDH ionization constant	(Cortassa et al., 2006)
$k_{h,2}$	9×10^{-4}	mM	IDH ionization constant	(Cortassa et al., 2006)
K_M^{ISOC}	1.52	mM	IDH Michaelis constant for isocitrate	(Cortassa et al., 2006)
n_i	2.0		IDH cooperativity for isocitrate	(Cortassa et al., 2006)
K_{Midh}^{NAD}	0.923	mM	Michaelis constant for NAD^+	(Cortassa et al., 2006)
K_{ADP}^a	0.62	mM	Activation constant by ADP	(Cortassa et al., 2006)
K_{Ca}^a	5×10^{-4}	mM	IDH activation constant for Ca^{2+}	(Cortassa et al., 2006)
E_T^{KGDH}	0.5	mM	α-ketoglutarate dehydrogenase (KGDH) concentration	(Cortassa et al., 2006)
k_{cat}^{KDGH}	0.0132	ms^{-1}	KGDH rate constant	(Cortassa et al., 2006)
$k_M^{\alpha KG}$	30	mM	KGDH Michaelis constant for alpha-ketoglutarate (αKG)	(Cortassa et al., 2006)
$K_{M\_kgdh}^{NAD}$	38.7	mM	KGDH Michaelis constant for NAD^+	(Cortassa et al., 2006)
$k_{h,1a}$	4×10^{-5}	mM	KGDH ionization constant	(Cortassa et al., 2006)
$k_{h,2a}$	7×10^{-5}	mM	KGDH ionization constant	(Cortassa et al., 2006)
$K_D^{Mg^{2+}}$	0.0308	mM	Activation constant for Mg^{2+}	(Cortassa et al., 2006)
$K_D^{Ca^{2+}}$	1.5×10^{-4}	mM	Activation constant for Ca^{2+}	(Cortassa et al., 2006)
$n_{\alpha KG}$	1.2		KGDH Hill coefficient for α KG	(Cortassa et al., 2006)
$[Mg^{2+}]_m$	0.4	mM	Mg^{2+} concentration in mitochondria	(Cortassa et al., 2006)
k_f^{SL}	2.8×10^{-3}	mM^{-1} ms^{-1}	Forward rate constant of succinate lyase (SL)	(Cortassa et al., 2006)
K_E^{SL}	3.115		SL equilibrium constant	(Cortassa et al., 2006)
k_f^{FH}	8.3×10^{-3}	ms^{-1}	Forward rate constant for fumarate hydratase (FH)	(Cortassa et al., 2006)
K_E^{FH}	1.0		FH equilibrium constant	(Cortassa et al., 2006)
k_{h1}	1.131×10^{-5}	mM	Ionization constant of malate dehydrogenase (MDH)	(Cortassa et al., 2006)
k_{h2}	26.7	mM	MDH ionization constant	(Cortassa et al., 2006)

Symbol	Value	Units	Description	References
k_{h3}	6.68×10^{-9}	mM	MDH ionization constant	(Cortassa et al., 2006)
k_{h4}	5.62×10^{-6}	mM	MDH ionization constant	(Cortassa et al., 2006)
k_{offset}	3.99×10^{-2}		Offset of MDH pH activation factor	(Cortassa et al., 2006)
k_{cat}^{MDH}	0.1242	ms^{-1}	MDH rate constant	(Cortassa et al., 2006)
E_T^{MDH}	0.154	mM	Total MDH concentration	(Cortassa et al., 2006)
K_M^{MAL}	1.493	mM	MDH Michaelis constant for malate	(Cortassa et al., 2006)
K_i^{OAA}	0.031	mM	Inhibition constant for oxalacetate	(Cortassa et al., 2006)
K_M^{NAD}	0.2244	mM	MDH Michaelis constant for NAD$^+$	(Cortassa et al., 2006)
[GLU]	1×10^{-3}	mM	Glutamate concentration	(Cortassa et al., 2006)
k_f^{AAT}	0.0214	ms^{-1}	Forward rate constant of aspartate amino transferase (AAT)	(Cortassa et al., 2006)
K_E^{AAT}	6.6		AAT equilibrium constant	(Cortassa et al., 2006)
k_{ASP}	1.5×10^{-6}	ms^{-1}	Rate constant of aspartate consumption	(Cortassa et al., 2006)

1.2.8 Oxidative Phosphorylation rate equations

Complex I-linked electron transport

$$V_{O_2} = 0.5\rho^{res} \frac{\left(r_a + r_{c1} e^{\left(\frac{6F\Delta\Psi_B}{RT}\right)} \right) e^{\left(\frac{FA_{res}}{RT}\right)} - r_a e^{\left(\frac{g6F\Delta\mu_H}{RT}\right)} + r_{c2} e^{\left(\frac{FA_{res}}{RT}\right)} e^{\left(\frac{g6F\Delta\mu_H}{RT}\right)}}{\left(1 + r_1 e^{\left(\frac{FA_{res}}{RT}\right)}\right) e^{\left(\frac{6F\Delta\Psi_B}{RT}\right)} + \left(r_2 + r_3 e^{\left(\frac{FA_{res}}{RT}\right)} \right) e^{\left(\frac{g6F\Delta\mu_H}{RT}\right)}} \quad \text{(S123)}$$

$$V_{He} = 6\rho^{res} \frac{\left(r_a e^{\left(\frac{A_{res}F}{RT}\right)} - (r_a + r_b) e^{\left(\frac{g6F\Delta\mu_H}{RT}\right)} \right)}{\left(1 + r_1 e^{\left(\frac{FA_{res}}{RT}\right)}\right) e^{\left(\frac{6F\Delta\Psi_B}{RT}\right)} + \left(r_2 + r_3 e^{\left(\frac{FA_{res}}{RT}\right)} \right) e^{\left(\frac{g6F\Delta\mu_H}{RT}\right)}}$$

$$A_{res} = \frac{RT}{F}\ln\left(K_{res}\sqrt{\frac{[NADH]}{[NAD^+]}}\right)$$

H+ transport linked to Succinate dehydrogenase (HSDH)

$$V_{He(SDH)} = 4\rho^{res(SDH)}\frac{\left(r_a e^{\left(\frac{A_{RSDH}F}{RT}\right)} - (r_a + r_b)e^{\left(\frac{g4F\Delta\mu_H}{RT}\right)}\right)}{\left(1 + r_1 e^{\left(\frac{FA_{RSDH}}{RT}\right)}\right)e^{\left(\frac{4F\Delta\Psi_B}{RT}\right)} + \left(r_2 + r_3 e^{\left(\frac{FA_{RSDH}}{RT}\right)}\right)e^{\left(\frac{g4F\Delta\mu_H}{RT}\right)}}\left(\frac{1}{1+\frac{[OAA]}{K_i^{OAA}}}\right) \quad \text{(S124)}$$

$$A_{RSDH} = \frac{RT}{F}\ln\left(K_{RSDH,app}\sqrt{\frac{[SUC]}{[FUM]}}\right)$$

$$K_{RSDH,app} = \frac{K_{res(SDH)}}{P_{SUC}}$$

Complex II linked electron transport from ETF

$$V_{O_2}^{ETF} = 0.5\rho^{res(ETF)}\frac{\left(r_a + r_{c1}e^{\left(\frac{4F\Delta\Psi_B}{RT}\right)}\right)e^{\left(\frac{FA_{ETF}}{RT}\right)} - r_a e^{\left(\frac{g4F\Delta\mu_H}{RT}\right)} + r_{c2}e^{\left(\frac{FA_{ETF}}{RT}\right)}e^{\left(\frac{g4F\Delta\mu_H}{RT}\right)}}{\left(1 + r_1 e^{\left(\frac{FA_{ETF}}{RT}\right)}\right)e^{\left(\frac{4F\Delta\Psi_B}{RT}\right)} + \left(r_2 + r_3 e^{\left(\frac{FA_{ETF}}{RT}\right)}\right)e^{\left(\frac{g4F\Delta\mu_H}{RT}\right)}} \quad \text{(S125)}$$

$$V_{He(ETF)} = 4\rho^{res(ETF)}\frac{\left(r_a e^{\left(\frac{A_{ETF}F}{RT}\right)} - (r_a + r_b)e^{\left(\frac{g4F\Delta\mu_H}{RT}\right)}\right)}{\left(1 + r_1 e^{\left(\frac{FA_{ETF}}{RT}\right)}\right)e^{\left(\frac{4F\Delta\Psi_B}{RT}\right)} + \left(r_2 + r_3 e^{\left(\frac{FA_{ETF}}{RT}\right)}\right)e^{\left(\frac{g4F\Delta\mu_H}{RT}\right)}}$$

$$A_{ETF} = \frac{RT}{F}\ln\left(K_{ETF,app}\sqrt{\frac{[FADH2]}{[FAD]}}\right)$$

$$K_{ETF,app} = \frac{K_{res(FAD)}}{P_{H_2O}}$$

ATP synthase (ATPase)

$$V_{ATPase} = -\rho^{F1} \frac{\left(100p_a + p_{c1}\exp(3F\Delta\Psi_B / RT)\right)\exp(A_{F1}F/RT) - \begin{pmatrix} p_a \exp(3F\Delta\mu_H / RT) \\ \cdots + p_{c2}\exp(A_{F1}F/RT)\exp(3F\Delta\mu_H / RT) \end{pmatrix}}{\left(1 + p_1\exp(A_{F1}F/RT)\right)\exp(3F\Delta\Psi_B / RT) + \left(p_2 + p_3\exp(A_{F1}F/RT)\right)\exp(3F\Delta\mu_H / RT)} \quad \text{(S126)}$$

$$V_{Hu} = -3\rho^{F1} \frac{p_a\left(1 + \exp(A_{F1}F/RT)\right) - \left(p_a + p_b\right)\exp(3F\Delta\mu_H / RT)}{\left(1 + p_1\exp(A_{F1}F/RT)\right)\exp(3F\Delta\Psi_B / RT) + \left(p_2 + p_3\exp(A_{F1}F/RT)\right)\exp(3F\Delta\mu_H / RT)}$$

$$A_{F1} = \frac{RT}{F}\ln(K_{app}^{ATPase}\frac{[MgATP^{2-}]}{[ADP_{free}][Pi_{total}]})$$

$$K_{app}^{ATPase} = K_{eq}^{ATPase}[H^+]^1 \frac{P_{ATP}P_{H_2O}}{P_{ADP}P_{Pi}}$$

Adenine nucleotide translocator (ANT)

$$V_{ANT} = \mathrm{V}_{\mathrm{maxANT}} \frac{\left(1 - \frac{[ATP^{4-}]_i \times [ADP^{3-}]_m}{[ADP^{3-}]_i \times [ATP^{4-}]_m}\right)\exp(-F\Delta\Psi_m / RT)}{\left(1 + \frac{[ATP^{4-}]_i}{[ADP^{3-}]_i}\exp(-hF\Delta\Psi / RT)\right)\left(1 + \frac{[ADP^{3-}]_m}{[ATP^{4-}]_m}\right)} \quad \text{(S127)}$$

Citrate carrier

$$V_{CiC} = \frac{V_{\max}^{CiC}\left(\frac{MAL_c\ .\ Cit_m}{K_{M\_CiC}^{MAL_c}.K_{M\_CiC}^{Cit_m}} - \frac{MAL_m\ .\ Cit_c}{K_{M\_CiC}^{MAL_m}.K_{M\_CiC}^{Cit_c}}\right)}{1 + \frac{MAL_m\ .\ Cit_c}{K_{M\_CiC}^{MAL_m}.K_{M\_CiC}^{Cit_c}} + \frac{MAL_c}{K_{M\_CiC}^{MAL_c}} + \frac{Cit_m}{K_{M\_CiC}^{Cit_m}} + \frac{MAL_c\ .\ Cit_m}{K_{M\_CiC}^{MAL_c}.K_{M\_CiC}^{Cit_m}} + \frac{MAL_m}{K_{M\_CiC}^{MALm}} + \frac{Cit_c}{K_{M\_CiC}^{Cit_c}}}. \quad \text{(S128)}$$

$$\left(1 + \frac{H^+}{K_{h1,c}} + \frac{K_{h2,c}}{H^+}\right)^{-1}$$

1.2.9 Supplementary Table S5. Parameter values of oxidative phosphorylation

Symbol	Value	Units	Description	Reference
r_a	6.394×10^{-13}	ms^{-1}	Sum of products of rate constants	(Cortassa et al., 2003)
r_b	1.762×10^{-16}	ms^{-1}	Sum of products of rate constants	(Cortassa et al., 2003)
r_{c1}	2.656×10^{-22}	ms^{-1}	Sum of products of rate constants	(Cortassa et al., 2003)
r_{c2}	8.632×10^{-30}	ms^{-1}	Sum of products of rate constants	(Cortassa et al., 2003)
r_1	2.077×10^{-18}		Sum of products of rate constants	(Cortassa et al., 2003)
r_2	1.728×10^{-9}		Sum of products of rate constants	(Cortassa et al., 2003)
r_3	1.059×10^{-26}		Sum of products of rate constants	(Cortassa et al., 2003)
ρ^{res}	0.2	mM	Concentration of electron carriers (respiratory complexes I-III-IV)	(Cortassa et al., 2003)
K_{res}	1.35×10^{18}		Equilibrium constant of respiration	(Cortassa et al., 2003)
$\rho^{res(SDH)}$	0. 017	mM	Concentration of electron carriers (respiratory complexes II-III-IV)	(Cortassa et al., 2003)
$\Delta\Psi_B$	50	mV	Phase boundary potential	(Cortassa et al., 2003)
g	0.85		Correction factor for voltage	(Cortassa et al., 2003)
K_i^{OAA}	0.15		Inhibition constant for OAA	(Cortassa et al., 2003)
$K_{res(SDH)}$	5.765×10^{13}		Equilibrium constant of SDH	(Cortassa et al., 2003)
$\rho^{res(ETF)}$	0. 75	mM	Concentration of electron carriers (respiratory complexes II-III-IV)	(Cortassa et al., 2003)
$K_{res(FAD)}$	6.949×10^{16}		Equilibrium constant of $FADH_2$	(Cortassa et al., 2003)
p_a	1.656×10^{-8}	ms^{-1}	Sum of products of rate constants	(Cortassa et al., 2003)
p_b	3.373×10^{-10}	ms^{-1}	Sum of products of rate constants	(Cortassa et al., 2003)
p_{c1}	9.651×10^{-17}	ms^{-1}	Sum of products of rate constants	(Cortassa et al., 2003)
p_{c2}	4.585×10^{-17}	ms^{-1}	Sum of products of rate constants	(Cortassa et al., 2003)
p_1	1.346×10^{-4}		Sum of products of rate constants	(Cortassa et al., 2003)
p_2	7.739×10^{-7}		Sum of products of rate constants	(Cortassa et al., 2003)
p_3	6.65×10^{-15}		Sum of products of rate constants	(Cortassa et al., 2003)

Symbol	Value	Units	Description	Reference
ρ^{F1}	1.5	mM	Concentration of F_1F_0-ATPase	(Cortassa et al., 2003)
K_{eq}^{ATPase}	1.71×10^{6}		Equilibrium constant of ATP synthesis	(Cortassa et al., 2003)
V_{maxANT}	3.15	mM. ms^{-1}	Maximal rate of the ANT	(Cortassa et al., 2003)
h^{ANT}	0.5		Fraction of $\Delta\Psi_B$	(Cortassa et al., 2003)
$V_{\max}^{CiC}$	0.01	mM. ms^{-1}	Maximal rate of citrate carrier	(Bisaccia et al., 1990)
$K_{M\_CiC}^{MALc}$	0.17	mM	CiC Michaelis constant for cyto malate	(Bisaccia et al., 1990)
$K_{M\_CiC}^{MALm}$	0.057	mM	CiC Michaelis constant for mito malate	(Bisaccia et al., 1990)
$K_{M\_CiC}^{Cit\,c}$	0.036	mM	CiC Michaelis constant for cyto citrate	(Bisaccia et al., 1990)
$K_{M\_CiC}^{Cit\,m}$	0.288	mM	CiC Michaelis constant for mito citrate	(Bisaccia et al., 1990)
$k_{h1,c}$	2.0×10^{-4}		Ionization constant for CiC	(Bisaccia et al., 1990)
$k_{h2,c}$	7.5×10^{-6}		Ionization constant for CiC	(Bisaccia et al., 1990)

1.2.10 Acid-base equilibria of adenine nucleotides and phosphate

$$[ATP^{4-}]_m = \frac{[ATP_{total}]_m}{\left(1 + \frac{[H^+]_m}{K_{a,ATP}} + \frac{[Mg^{2+}]_m}{K_{Mg,ATP}}\right)} \tag{S129}$$

$$[HATP^{3-}]_m = \frac{[ATP^{4-}]_m[H^+]_m}{K_{a,ATP}} \qquad [MgATP^{2-}]_m = \frac{[ATP^{4-}]_m[Mg^{2+}]_m}{K_{Mg,ATP}}$$

$$[ADP^{3-}]_m = \frac{[ADP_{total}]_m}{\left(1 + \frac{[H^+]_m}{K_{a,ADP}} + \frac{[Mg^{2+}]_m}{K_{Mg,ADP}}\right)}$$

$$[HADP^{2-}]_m = \frac{[ADP^{3-}]_m[H^+]_m}{K_{a,ADP}} \qquad [MgADP^{-}]_m = \frac{[ADP^{3-}]_m[Mg^{2+}]_m}{K_{Mg,ADP}}$$

$$[H_2PO_4^-]_m = \frac{[Pi]_{total}}{1+\frac{[H^+]_m}{K_{a,Pi}}} \tag{S130}$$

$$[HPO_4^{2-}]_m = \frac{[H_2PO_4^-]_m K_{a,Pi}}{[H^+]_m}$$

$$[ATP^{4-}]_i = \frac{[ATP_{total}]_i}{\left(1+\frac{[H^+]_i}{K_{a,ATP}}+\frac{[Mg^{2+}]_i}{K_{Mg,ATP}}\right)} \qquad [ADP^{3-}]_i = \frac{[ADP_{total}]_i}{\left(1+\frac{[H^+]_i}{K_{a,ADP}}+\frac{[Mg^{2+}]_i}{K_{Mg,ADP}}\right)} \tag{S131}$$

$$\begin{aligned} &[ATP_{total}] = [ATP^{4-}] + [HATP^{3-}] + [MgATP^{-}] \\ &[ATP_{free}] = [ATP^{4-}] + [HATP^{3-}] \\ &[ADP_{free}] = [ADP^{3-}] + [HADP^{2-}] \\ &[Pi_{total}] = [H_2Pi^{-}] + [H\ Pi^{2-}] \end{aligned} \tag{S132}$$

1.2.11 Polynomials for species undergoing acid-base equilibrium, ionic gradients, and conservation relations

$$P_{ATP} = 1+\frac{[H^+]_m}{K_{a,ATP}}+\frac{[Mg^{2+}]_m}{K_{Mg,ATP}} \tag{S133}$$

$$\begin{aligned} &P_{ADP} = 1+\frac{[H^+]_m}{K_{a,ADP}}+\frac{[Mg^{2+}]_m}{K_{Mg,ADP}} \\ &P_{Pi} = 1+\frac{[H^+]_m}{K_{a,Pi}} \\ &P_{SUC} = 1+\frac{[H^+]_m}{K_{a,SUC}} \\ &P_{H_2O} = 1+\frac{[H^+]_m}{K_{a,H_2O}} \end{aligned} \tag{S134}$$

$$\begin{aligned} &\Delta\mu_H = -2.303\frac{RT}{F}\Delta pH + \Delta\Psi_m \\ &\Delta pH = pH_i - pH_m \\ &\Delta\Psi_m = \Psi_i - \Psi_m \end{aligned} \tag{S135}$$

$$[NAD^+] = C_{PN} - [NADH] \tag{S136}$$

$$[ATP_{total}] = C_A - [ADP_{total}] \tag{S137}$$

1.2.12 Supplementary Table S6. Parameters used in acid base equilibria and conservation relations

Symbol	Value	Units	Description	Reference
$K_{a,ADP}$	4.17×10^{-7}		ADP dissociation constant	(Wei et al., 2011)
$K_{a,ATP}$	3.31×10^{-7}		ATP dissociation constant	(Wei et al., 2011)
$K_{a,Pi}$	1.78×10^{-7}		Pi dissociation constant	(Wei et al., 2011)
$K_{Mg,ATP}$	6.46×10^{-5}		Mg^{2+}ATP dissociation constant	(Wei et al., 2011)
$K_{Mg,ADP}$	5.62×10^{-4}		Mg^{2+}ADP dissociation constant	(Wei et al., 2011)
$K_{a,SUC}$	6.3×10^{-6}		Ka of succinate dissociation constant	(Wei et al., 2011)
K_{a,H_2O}	1×10^{-14}	M	Dissociation constant for water	(Wei et al., 2011)
C_A	1.5	mM	Total sum of adenine nucleotides	(Wei et al., 2011)
$[ADP]_i$	0.005-0.1	mM	Cytoplasmic ADP concentration	(Wei et al., 2011)
C_{PN}	1.0	mM	Sum of pyridine nucleotides	(Wei et al., 2011)

1.2.13 Ionic fluxes rate equations

$$V_{uni} = V_{\max}^{uni} \frac{\dfrac{[Ca^{2+}]_i}{K_{trans}}\left(1+\dfrac{[Ca^{2+}]_i}{K_{trans}}\right)^3 \dfrac{2F\left(\Delta\Psi_m - \Delta\Psi^{\circ}\right)}{RT}}{\left(\left(1+\dfrac{[Ca^{2+}]_i}{K_{trans}}\right)^4 + \dfrac{L}{\left(1+\dfrac{[Ca^{2+}]_i}{K_{act}}\right)^{n_a}}\right)\left(1-e^{\left(\frac{-2F\left(\Delta\Psi_m-\Delta\Psi^{\circ}\right)}{RT}\right)}\right)} \tag{S138}$$

$$V_{NaCa} = V_{\max}^{NaCa} \frac{e^{\left(\frac{bF\left(\Delta\Psi_m-\Delta\Psi^{\circ}\right)}{RT}\right)} e^{\left(\ln\frac{[Ca^{2+}]_m}{[Ca^{2+}]_i}\right)}}{\left(1+\dfrac{K_{Na}}{[Na^+]_i}\right)^n \left(1+\dfrac{K_{Ca}}{[Ca^{2+}]_m}\right)} \tag{S139}$$

$$J_{NHE} = c_{NHE} \frac{\dfrac{\beta_1^+\beta_2^+ - \beta_1^-\beta_2^-}{\beta_1^+ + \beta_1^- + \beta_2^+ + \beta_2^-}}{1+10^{n_i(pH_i - pK_i)}} \tag{S140}$$

$$\beta_1^+ = \frac{k_1^+ K_{H\_NHE}[Na^+]_m}{K_{H\_NHE}[Na^+]_m + K_{H\_NHE}K_{Na\_NHE} + K_{Na\_NHE}[H^+]_m}$$

$$\beta_2^+ = \frac{k_2^+ K_{Na\_NHE}[H^+]_i}{K_{H\_NHE}[Na^+]_i + K_{H\_NHE}K_{Na\_NHE} + K_{Na\_NHE}[H^+]_i}$$

$$\beta_1^- = \frac{k_1^- K_{H\_NHE}[Na^+]_i}{K_{H\_NHE}[Na^+]_i + K_{H\_NHE}K_{Na\_NHE} + K_{Na\_NHE}[H^+]_i}$$

$$\beta_2^- = \frac{k_2^- K_{Na\_NHE}[H^+]_m}{K_{H\_NHE}[Na^+]_m + K_{H\_NHE}K_{Na\_NHE} + K_{Na\_NHE}[H^+]_m}$$

$$J_{PIC} = c_{PiC}\frac{V_{PIC,f}\dfrac{[HPO_4^{2-}]_i[OH^-]_m}{K_{Pi,i}K_{OH,m}} - V_{PIC,b}\dfrac{[HPO_4^{2-}]_m[OH^-]_i}{K_{Pi,m}K_{OH,i}}}{\left(\begin{array}{l} 1+\dfrac{[HPO_4^{2-}]_i}{K_{Pi,i}}+\dfrac{[OH^-]_m}{K_{OH,m}}+\dfrac{[HPO_4^{2-}]_m}{K_{Pi,m}}+\ldots \\ \dfrac{[OH^-]_i}{K_{OH,i}}+\dfrac{[HPO_4^{2-}]_m[OH^-]_i}{K_{Pi,m}K_{OH,i}}+\dfrac{[HPO_4^{2-}]_i[OH^-]_m}{K_{Pi,i}K_{OH,m}} \end{array}\right)} \tag{S141}$$

$$V_{Hleak} = \mathrm{g_H}\left(1+\frac{F_{FA}.C16CoA_c^{\ 4}}{(C16CoA_c + C16CoA_{Ref})^4}\right)\Delta\mu_{\mathrm{H}} \tag{S142}$$

1.2.14 Supplementary Table S7. Parameters for the mitochondrial ion handling equations

Symbol	Value	Units	Description	Reference
V_{max}^{uni}	4.459×10^{-3}	mM ms^{-1}	V_{max} Ca^{2+} uniporter	(Cortassa et al., 2003)
$\Delta\Psi^{\circ}$	91	mV	Offset membrane potential	(Cortassa et al., 2003)
K_{act}	3.8×10^{-4}	mM	Activation constant	(Cortassa et al., 2003)
K_{trans}	0.019	mM	K_d for translocated Ca^{2+}	(Cortassa et al., 2003)
L	110.0		K_{eq} for conformational transitions in uniporter	(Cortassa et al., 2003)
n_a	2.8		Uniporter activation cooperativity	(Cortassa et al., 2003)
V_{max}^{NaCa}	1.833×10^{-4}	mM ms^{-1}	V_{max} of Na^+/Ca^{2+} exchanger	(Cortassa et al., 2003)
b	0.5		$\Delta\Psi_m$ dependence on Na^+/Ca^{2+} exchanger	(Cortassa et al., 2003)
K_{Na}	9.4	mM	Exchanger Na^{2+} constant	(Cortassa et al., 2003)
K_{Ca}	3.75×10^{-4}	mM	Exchanger Ca^{2+} constant	(Cortassa et al., 2003)
n	3.0		Na^+/Ca^{2+} exchanger cooperativity	(Cortassa et al., 2003)
δ_{Ca}	3×10^{-4}		Fraction of free $[Ca^{2+}]_m$	(Cortassa et al., 2003)

Symbol	Value	Units	Description	Reference
δ_H	1×10^{-5}		mitochondria H^+ buffering capacity	(Wei et al., 2011)
k_1^+	0.0252	ms^{-1}	NHE forward rate constant	(Wei et al., 2011)
k_1^-	0.0429	ms^{-1}	NHE backward rate constant	(Wei et al., 2011)
k_4^+	0.16	ms^{-1}	NHE forward rate constant	(Wei et al., 2011)
k_4^-	0.0939	ms^{-1}	NHE backward rate constant	(Wei et al., 2011)
K_{Na_NHE}	24	mM	Na^+ dissociation constant	(Wei et al., 2011)
K_{H_NHE}	1.585×10^{-4}	mM	H^+ dissociation constant	(Wei et al., 2011)
pK_i	8.52		Proton inhibitory constant	(Wei et al., 2011)
n_i_NHE	3		Hill coefficient for H^+ binding	(Wei et al., 2011)
c_{NHE}	0.00785	mM	NHE concentration	(Wei et al., 2011)
$K_{Pi,i}$	11.06	mM	Extra-matrix Pi binding constant	(Wei et al., 2011)
$K_{Pi,m}$	11.06	mM	Mitochondrial matrix Pi binding constant	(Wei et al., 2011)
$K_{OH,m}$	4.08×10^{-5}	mM	Mitochondrial matrix OH-binding constant	(Wei et al., 2011)
$K_{OH,i}$	4.08×10^{-5}	mM	Extra-matrix OH- binding constant	(Wei et al., 2011)
$V_{PIC,f}$	7.35×10^{-3}	mM ms^{-1}	Forward V_{max} of phosphate carrier	(Wei et al., 2011)
$V_{PIC,b}$	7.35×10^{-3}	mM ms^{-1}	Backward V_{max} of phosphate carrier	(Wei et al., 2011)
$[Pi]_i$	3.0	mM	Cytoplasmic phosphate concentration	(Wei et al., 2011)
g_H	2.0×10^{-6}	mM ms^{-1} mV^{-1}	Ionic conductance of the inner membrane	(Wei et al., 2011)
FFA	1.0×10^{2}		Leak-activation factor by PCoA	(Cortassa et al., 2017a)
$C16CoA_{Ref}$	35	µM	Reference concentration of PCoA	(Cortassa et al., 2017a)
$[H^+]_i$	1×10^{-4}	mM	Cytoplasmic H^+ concentration	(Wei et al., 2011)
$[Na^+]_i$	10.0	mM	Cytoplasmic Na^+ concentration	(Wei et al., 2011)
$[Ca^{2+}]_i$	1×10^{-4}	mM	Cytoplasmic Ca^{2+} concentration	(Cortassa et al., 2003)
C_{mito}	1.812×10^{-3}	mM mV^{-1}	Inner membrane capacitance	(Cortassa et al., 2003)

1.2.15 Transport of activated fatty acid into the mitochondrial matrix

Carnitine palmitoyl transferase I (Eq. S143), Carnitine Acyl carnitine translocase (Eq. S144) and Carnitine Palmitoyl transferase II (Eq. S145). The enzymes' rate expressions were modelled as bi-reactant reversible reactions, with CPT1 being inhibited by cytoplasmic Malonyl Coenzyme A (van Eunen et al., 2013). Because of the three successive steps catalyzed by the above-mentioned transporters/enzymes, PCoA is transported from the cytoplasmic into the mitochondrial matrix compartment where β-oxidation takes place.

$$V_{cpt1}^{C16} = sf_{cpt1}^{C16} \cdot V_{cpt1} \frac{\left(\frac{C16CoA_c}{K_{M\,cpt1}^{C16CoAc}} \cdot \frac{Carn_c}{K_{M\,cpt1}^{Carn\,c}}\right) - \left(\frac{C16Carn_c}{K_{M\,cpt1}^{C16CoAc}} \cdot \frac{CoA_c}{K_{M\,cpt1}^{Carn\,c} K_{EQ}^{cpt1}}\right)}{\left(1 + \frac{C16CoA_c}{K_{M\,cpt1}^{C16CoAc}} + \frac{C16Carn_c}{K_{M\,cpt1}^{C16Carn\,c}} + \left(\frac{MalCoA_c}{K_{i\,cpt1}^{MalCoAc}}\right)^{n\,cpt1}\right)\left(1 + \frac{Carn_c}{K_{M\,cpt1}^{Carn\,c}} + \frac{CoA_c}{K_{M\,cpt1}^{CoAc}}\right)} \tag{S143}$$

$$V_{cact}^{C16} = V_{f\,cact} \frac{\left(C16Carn_c . Carn_m\right) - \left(C16Carn_m \cdot \frac{Carn_c}{K_{EQ}^{cact1}}\right)}{\left(\begin{array}{l} C16Carn_c . Carn_m + K_{M\,cact}^{Carn\,m} C16Carn_c + K_{M\,cact}^{C16Carn\,c} . Carn_m \left(1 + \frac{Carn_c}{K_{i\,cact}^{Carn\,c}}\right) + \cdots \\ \frac{V_{f\,cact}}{V_{r\,cact} K_{EQ}^{cact}} \left(K_{M\,cact}^{Carn\,c} C16Carn_m \left(1 + \frac{C16Carn_c}{K_{i\,cact}^{C16Carn\,c}}\right) + Carn_c . \left(K_{M\,cact}^{C16Carn\,m} + C16Carn_m\right)\right) \end{array}\right)} \tag{S144}$$

$$V_{cpt2}^{C16} = sf_{cpt2}^{C16} \cdot V_{cpt2} \frac{\left(\frac{C16Carn_m}{K_{M\,cpt2}^{C16Carn\,m}} \cdot \frac{CoA_m}{K_{M\,cpt2}^{CoA\,m}}\right) - \left(\frac{C16CoA_m}{K_{M\,cpt2}^{C16Carn\,m}} \cdot \frac{Carn_m}{K_{M\,cpt2}^{CoA\,m} K_{EQ}^{cpt2}}\right)}{\left(1 + \frac{C16Carn_m}{K_{M\,cpt2}^{C16Carn\,m}} + \frac{C16CoA_m}{K_{M\,cpt2}^{C16CoA\,m}}\right)\left(1 + \frac{Carn_m}{K_{M\,cpt2}^{Carn\,m}} + \frac{CoA_m}{K_{M\,cpt2}^{CoA\,m}}\right)} \tag{S145}$$

1.2.16 Supplementary Table S8. Parameters for fatty acids transport into the mitochondrial matrix

Symbol	Value	Units	Description
sf_{cpt1}^{C16}	1.0		Scale factor of CPT1 for PalmitoylCarnitine (Pcarnit)
V_{cpt1}	1.0×10^{-3}	mM ms^{-1}	Maximal rate of CPT1
$K_{M\,cpt1}^{C16CoAc}$	13.8	µM	CPT1 Michaelis constant (K_M) for PCoA
$K_{M\,cpt1}^{Carn\,c}$	125	µM	CPT1 K_M for Carnitine
$K_{i\,cpt1}^{MalCoAc}$	9.1	µM	CPT1 inhibition constant for Malonyl CoA
$K_{M\,cpt1}^{C16Carn\,c}$	136	µM	CPT1 K_M for Pcarnit

Symbol	Value	Units	Description
$K_{M\,cpt1}^{CoA\,c}$	40.7	µM	CPT1 K_M for Coenzyme A (CoA)
$C16CoA_c$	0-40	µM	Concentration of cytoplasmic PCoA
$MalCoA_c$	0.0	µM	Extra-matrix concentration of Malonyl CoA
$n\,cpt1$	2.48		CPT1 Hill Coefficient for MalCoAc
K_{EQ}^{cpt1}	0.45		CPT1 equilibrium constant
$V_{f\,cact}$	0.7	mM ms^{-1}	Forward maximal rate of carnitine acyl carnitine translocase (CACT)
$V_{r\,cact}$	0.7	mM ms^{-1}	CACT reverse rate
$K_{M\,cact}^{Carn\,m}$	0.13	mM	CACT K_M for Carnitine (mito)
$K_{M\,cact}^{Carn\,c}$	1.3	mM	CACT K_M for Carnitine (cyto)
$K_{i\,cact}^{C16Carn\,c}$	15	µM	CACT inhibition constant for Pcarnit
$K_{i\,cact}^{Carn\,c}$	0.2	mM	CACT inhibition constant for Carnitine
$K_{M\,cact}^{C16Carn\,m}$	0.15	mM	CACT K_M for Pcarnit (mito)
$K_{M\,cact}^{C16Carn\,c}$	0.56	mM	CACT K_M for Pcarnit (cyto)
$Carn_c$	0.4	mM	Cytoplasmic Carnitine
$Carn_m$	0.95	mM	Mitochondrial Carnitine
K_{EQ}^{cact1}	1	-	CACT equilibrium constant
sf_{cpt2}^{C16}	0.85		Scale factor of carnitine palmitoyl transferase2 (CPT2) for Pcarnit
V_{cpt2}	1.955 10^{-2}	mM ms^{-1}	CPT2 forward maximal rate
$K_{M\,cpt2}^{C16Carn\,m}$	51	µM	CPT2 K_M for Pcarnit (mito)
$K_{M\,cpt2}^{C16CoA\,m}$	38	µM	CPT2 K_M for PCoA (mito)
$K_{M\,cpt2}^{CoA\,m}$	30		CPT2 K_M for CoA (mito)
$K_{M\,cpt2}^{Carn\,m}$	0.35	mM	CPT2 K_M for Carnitine (mito)
K_{EQ}^{cpt2}	2.22		CPT2 equilibrium constant

Parameter values were taken from (Cortassa et al., 2017a) and (van Eunen et al., 2013) with the exception of V_{cpt1}, $V_{f\,cact}$, $V_{r\,cact}$ and V_{cpt2} that were adjusted on the basis of fluxes of palmitate oxidation reported in adult cardiomyocytes (Mazumder et al., 2004).

Mitochondrial matrix fatty acyl-CoA dehydrogenases

These dehydrogenases catalyze the oxidation of AcylCoA using FAD as electron acceptor. We lumped the dehydrogenase step with the reduction of the FAD group in the electron transfer protein. The FAD group will be re-oxidized by complex II in the respiratory chain as indicated in equation S122 (see below).

$$V_{vlcad}^{C16} = sf_{vlcad}^{C16} \cdot V_{vlcad} \frac{\left(\frac{C16CoA_m}{K_{M\,vlcad}^{C16CoA\,m}} \cdot \frac{ETF_m}{K_{M\,vlcad}^{ETFm}}\right) - \left(\frac{C16enoCoA_m}{K_{M\,vlcad}^{C16CoA\,m}} \cdot \frac{ETFH_{2\,m}}{K_{M\,vlcad}^{ETF\,m}\,K_{EQ}^{vlcad}}\right)}{V_{vlcad}^{DENOMINATOR}} \quad \text{(S146)}$$

$$V_{vlcad}^{C14} = sf_{vlcad}^{C14} \cdot V_{vlcad} \frac{\left(\frac{C14CoA_m}{K_{M\,vlcad}^{C14CoA\,m}} \cdot \frac{ETF_m}{K_{M\,vlcad}^{ETFm}}\right) - \left(\frac{C14enoCoA_m}{K_{M\,vlcad}^{C14CoA\,m}} \cdot \frac{ETFH_{2\,m}}{K_{M\,vlcad}^{ETFm}\,K_{EQ}^{vlcad}}\right)}{V_{vlcad}^{DENOMINATOR}} \quad \text{(S147)}$$

$$V_{vlcad}^{C12} = sf_{vlcad}^{C12} \cdot V_{vlcad} \frac{\left(\frac{C12CoA_m}{K_{M\,vlcad}^{C12CoA\,m}} \cdot \frac{ETF_m}{K_{M\,vlcad}^{ETFm}}\right) - \left(\frac{C12enoCoA_m}{K_{M\,vlcad}^{C12CoA\,m}} \cdot \frac{ETFH_{2\,m}}{K_{M\,vlcad}^{ETFm}\,K_{EQ}^{vlcad}}\right)}{V_{vlcad}^{DENOMINATOR}} \quad \text{(S148)}$$

$$V_{vlcad}^{DENOMINATOR} = \begin{pmatrix} 1 + \frac{C16CoA_m}{K_{M\,vlcad}^{C16CoA\,m}} + \frac{C16enoCoA_m}{K_{M\,vlcad}^{C16enoCoA\,m}} + \frac{C14CoA_m}{K_{M\,vlcad}^{C14CoA\,m}} + \cdots \\ \frac{C14enoCoA_m}{K_{M\,vlcad}^{C14enoCoA\,m}} + \frac{C12CoA_m}{K_{M\,vlcad}^{C12CoA\,m}} + \frac{C12enoCoA_m}{K_{M\,vlcad}^{C12enoCoA\,m}} \end{pmatrix} \cdot \left(1 + \frac{ETF_m}{K_{M\,vlcad}^{ETFm}} + \frac{ETFH_{2\,m}}{K_{M\,vlcad}^{ETFH_2\,m}\,K_{EQ}^{vlcad}}\right)$$

$$V_{lcad}^{C16} = sf_{lcad}^{C16} \cdot V_{lcad} \frac{\left(\frac{C16CoA_m}{K_{M\,lcad}^{C16CoA\,m}} \cdot \frac{ETF_m}{K_{M\,lcad}^{ETFm}}\right) - \left(\frac{C16enoCoA_m}{K_{M\,lcad}^{C16CoA\,m}} \cdot \frac{ETFH_{2\,m}}{K_{M\,lcad}^{ETFm}\,K_{EQ}^{lcad}}\right)}{V_{lcad}^{DENOMINATOR}} \quad \text{(S149)}$$

$$V_{lcad}^{C14} = sf_{lcad}^{C14} \cdot V_{lcad} \frac{\left(\frac{C14CoA_m}{K_{M\,lcad}^{C14CoA\,m}} \cdot \frac{ETF_m}{K_{M\,lcad}^{ETFm}}\right) - \left(\frac{C14enoCoA_m}{K_{M\,lcad}^{C14CoA\,m}} \cdot \frac{ETFH_{2\,m}}{K_{M\,lcad}^{ETFm}\,K_{EQ}^{lcad}}\right)}{V_{lcad}^{DENOMINATOR}} \quad \text{(S150)}$$

$$V_{lcad}^{C12} = sf_{lcad}^{C12} \cdot V_{lcad} \frac{\left(\frac{C12CoA_m}{K_{M\,lcad}^{C12CoA\,m}} \cdot \frac{ETF_m}{K_{M\,lcad}^{ETFm}}\right) - \left(\frac{C12enoCoA_m}{K_{M\,lcad}^{C12CoA\,m}} \cdot \frac{ETFH_{2\,m}}{K_{M\,lcad}^{ETFm}\,K_{EQ}^{lcad}}\right)}{V_{lcad}^{DENOMINATOR}} \quad \text{(S151)}$$

$$V_{lcad}^{C10} = sf_{lcad}^{C10} \cdot V_{lcad} \frac{\left(\frac{C10CoA_m}{K_{M\,lcad}^{C10CoAm}} \cdot \frac{FAD}{K_{M\,lcad}^{ETFm}} \right) - \left(\frac{C10enoCoA_m}{K_{M\,lcad}^{C10CoAm}} \cdot \frac{FADH_2}{K_{M\,lcad}^{ETFm}\, K_{EQ}^{lcad}} \right)}{V_{lcad}^{DENOMINATOR}} \quad \text{(S152)}$$

$$V_{lcad}^{C8} = sf_{lcad}^{C8} \cdot V_{lcad} \frac{\left(\frac{C8CoA_m}{K_{M\,lcad}^{C8CoAm}} \cdot \frac{FAD_m}{K_{M\,lcad}^{ETFm}} \right) - \left(\frac{C8enoCoA_m}{K_{M\,lcad}^{C8CoAm}} \cdot \frac{FADH_{2\,m}}{K_{M\,lcad}^{ETFm}\, K_{EQ}^{lcad}} \right)}{V_{lcad}^{DENOMINATOR}} \quad \text{(S153)}$$

$$V_{lcad}^{DENOMINATOR} = \left(\begin{array}{c} 1 + \frac{C16CoA_m}{K_{M\,lcad}^{C16CoAm}} + \frac{C16enoCoA_m}{K_{M\,lcad}^{C16enoCoAm}} + \frac{C14CoA_m}{K_{M\,lcad}^{C14CoAm}} + \frac{C14enoCoA_m}{K_{M\,lcad}^{C14enoCoAm}} + \cdots \\ \frac{C12CoA_m}{K_{M\,lcad}^{C12CoAm}} + \frac{C12enoCoA_m}{K_{M\,lcad}^{C12enoCoAm}} + \frac{C10CoA_m}{K_{M\,lcad}^{C10CoAm}} + \cdots \\ \frac{C10enoCoA_m}{K_{M\,lcad}^{C10enoCoAm}} + \frac{C8CoA_m}{K_{M\,lcad}^{C8CoAm}} + \frac{C8enoCoA_m}{K_{M\,lcad}^{C8enoCoAm}} \end{array} \right) \cdot \left(1 + \frac{ETF_m}{K_{M\,lcad}^{ETFm}} + \frac{ETFH_{2\,m}}{K_{M\,lcad}^{ETFH_2 m}\, K_{EQ}^{lcad}} \right)$$

$$V_{mcad}^{C12} = sf_{mcad}^{C12} \cdot V_{mcad} \frac{\left(\frac{C12CoA_m}{K_{M\,mcad}^{C12CoAm}} \cdot \frac{FAD}{K_{M\,mcad}^{ETFm}} \right) - \left(\frac{C12enoCoA_m}{K_{M\,mcad}^{C12CoAm}} \cdot \frac{FADH_2}{K_{M\,mcad}^{ETFm}\, K_{EQ}^{mcad}} \right)}{V_{mcad}^{DENOMINATOR}} \quad \text{(S154)}$$

$$V_{mcad}^{C10} = sf_{mcad}^{C10} \cdot V_{mcad} \frac{\left(\frac{C10CoA_m}{K_{M\,mcad}^{C10CoAm}} \cdot \frac{ETF_m}{K_{M\,mcad}^{ETFm}} \right) - \left(\frac{C10enoCoA_m}{K_{M\,mcad}^{C10CoAm}} \cdot \frac{ETFH_{2\,m}}{K_{M\,mcad}^{ETFm}\, K_{EQ}^{mcad}} \right)}{V_{mcad}^{DENOMINATOR}} \quad \text{(S155)}$$

$$V_{mcad}^{C8} = sf_{mcad}^{C8} \cdot V_{mcad} \frac{\left(\frac{C8CoA_m}{K_{M\,mcad}^{C8CoAm}} \cdot \frac{ETF_m}{K_{M\,mcad}^{ETFm}} \right) - \left(\frac{C8enoCoA_m}{K_{M\,mcad}^{C8CoAm}} \cdot \frac{ETFH_{2\,m}}{K_{M\,mcad}^{ETFm}\, K_{EQ}^{mcad}} \right)}{V_{mcad}^{DENOMINATOR}} \quad \text{(S156)}$$

$$V_{mcad}^{C6} = sf_{mcad}^{C6} \cdot V_{mcad} \frac{\left(\frac{C6CoA_m}{K_{M\,mcad}^{C6CoAm}} \cdot \frac{ETF_m}{K_{M\,mcad}^{ETFm}} \right) - \left(\frac{C6enoCoA_m}{K_{M\,mcad}^{C6CoAm}} \cdot \frac{ETFH_{2\,m}}{K_{M\,mcad}^{ETFm}\, K_{EQ}^{mcad}} \right)}{V_{mcad}^{DENOMINATOR}} \quad \text{(S157)}$$

$$V_{mcad}^{C4} = sf_{mcad}^{C4} \cdot V_{mcad} \frac{\left(\frac{C4CoA_m}{K_{M\,mcad}^{C4CoAm}} \cdot \frac{ETF_m}{K_{M\,mcad}^{ETFm}} \right) - \left(\frac{C4enoCoA_m}{K_{M\,mcad}^{C4CoAm}} \cdot \frac{ETFH_{2\,m}}{K_{M\,mcad}^{ETFm}\, K_{EQ}^{mcad}} \right)}{V_{mcad}^{DENOMINATOR}} \quad \text{(S158)}$$

$$V_{mcad}^{DENOMINATOR}=\left(\begin{array}{c}1+\frac{C12CoA_m}{K_{M\,mcad}^{C12CoAm}}+\frac{C12enoCoA_m}{K_{M\,mcad}^{C12enoCoAm}}+\frac{C10CoA_m}{K_{M\,mcad}^{C10CoAm}}+\frac{C10enoCoA_m}{K_{M\,mcad}^{C10enoCoAm}}+\frac{C8CoA_m}{K_{M\,mcad}^{C8CoAm}}+\cdots\\ \frac{C8enoCoA_m}{K_{M\,mcad}^{C8enoCoAm}}+\frac{C6CoA_m}{K_{M\,lcad}^{C6CoAm}}+\frac{C6enoCoA_m}{K_{M\,mcad}^{C6enoCoAm}}+\frac{C4CoA_m}{K_{M\,mcad}^{C4CoAm}}+\frac{C4enoCoA_m}{K_{M\,mcad}^{C4enoCoAm}}\end{array}\right)\cdot\left(1+\frac{FAD_m}{K_{M\,mcad}^{ETFm}}+\frac{FADH_{2\,m}}{K_{M\,mcad}^{ETFH_2m}\,K_{EQ}^{mcad}}\right)$$

$$V_{scad}^{C6}=sf_{scad}^{C6}\cdot V_{scad}\,\frac{\left(\frac{C6CoA_m}{K_{M\,scad}^{C6CoAm}}\cdot\frac{ETF_m}{K_{M\,scad}^{ETFm}}\right)-\left(\frac{C6enoCoA_m}{K_{M\,scad}^{C6CoAm}}\cdot\frac{ETFH_{2\,m}}{K_{M\,scad}^{ETFm}\,K_{EQ}^{scad}}\right)}{V_{scad}^{DENOMINATOR}} \quad \text{(S159)}$$

$$V_{scad}^{C4}=sf_{scad}^{C4}\cdot V_{scad}\,\frac{\left(\frac{C4CoA_m}{K_{M\,scad}^{C4CoAm}}\cdot\frac{ETF_m}{K_{M\,scad}^{ETFm}}\right)-\left(\frac{C4enoCoA_m}{K_{M\,scad}^{C4CoAm}}\cdot\frac{ETFH_{2\,m}}{K_{M\,scad}^{ETFm}\,K_{EQ}^{scad}}\right)}{V_{scad}^{DENOMINATOR}} \quad \text{(S160)}$$

$$V_{scad}^{DENOMINATOR}=\left(1+\frac{C6CoA_m}{K_{M\,scad}^{C6CoAm}}+\frac{C6enoCoA_m}{K_{M\,scad}^{C6enoCoAm}}+\frac{C4CoA_m}{K_{M\,scad}^{C4CoAm}}+\frac{C4enoCoA_m}{K_{M\,scad}^{C4enoCoAm}}\right)\cdot\left(1+\frac{ETF_m}{K_{M\,scad}^{ETFm}}+\frac{ETFH_{2\,m}}{K_{M\,scad}^{ETFH_2m}\,K_{EQ}^{scad}}\right)$$

1.2.17 Supplementary Table S9. Parameters for fatty acyl-CoA dehydrogenases (cad)

Symbol	Value	Units	Description
sf_{vlcad}^{C16}	1		Scaling factor of very long acyl CoA dehydrogenase (vlcad) for PCoA
sf_{vlcad}^{C14}	0.8		vlcad scaling factor for C14CoA
sf_{vlcad}^{C12}	0.42		vlcad scaling factor for C12CoA
V_{vlcad}	2.0×10^{-3}	mM ms^{-1}	vlcad maximal rate
$K_{M\,vlcad}^{ETFm}$	0.12	µM	vlcad Michaelis constant (K_M) for FAD
$K_{M\,vlcad}^{ETFH_2m}$	24.2	µM	vlcad K_M for $FADH_2$
$K_{M\,vlcad}^{C16CoAm}$	6.5	µM	vlcad K_M for PCoA
$K_{M\,vlcad}^{C16enoCoAm}$	1.08	µM	vlcad K_M for C16enoyl-CoA
$K_{M\,vlcad}^{C14CoAm}$	4.0	µM	vlcad K_M for C14-CoA
$K_{M\,vlcad}^{C14enoCoAm}$	1.08	µM	vlcad K_M for C14enoyl-CoA
$K_{M\,vlcad}^{C12CoAm}$	2.7	µM	vlcad K_M for C12-CoA
$K_{M\,vlcad}^{C12enoCoAm}$	1.08	µM	vlcad K_M for C12enoyl-CoA
K_{EQ}^{vlcad}	6.0		vlcad equilibrium constant

Symbol	Value	Units	Description
sf_{lcad}^{C16}	0.9		Scaling factor of long-chain acyl CoA dehydrogenase (lcad) for PCoA
sf_{lcad}^{C14}	1		lcad scaling factor for C14CoA
sf_{lcad}^{C12}	0.95		lcad scaling factor for C12CoA
sf_{lcad}^{C10}	0.85		lcad scaling factor for C10CoA
sf_{lcad}^{C8}	0.4		lcad scaling factor for C8CoA
V_{lcad}	$2.5.10^{-3}$	mM ms^{-1}	lcad maximal rate
$K_{M\,lcad}^{ETFm}$	1.2	nM	lcad K_M for FAD
$K_{M\,lcad}^{ETFH_2m}$	24.2	µM	lcad K_M for $FADH_2$
$K_{M\,lcad}^{C16CoAm}$	2.5	µM	lcad K_M for PCoA
$K_{M\,lcad}^{C16enoCoAm}$	1.08	µM	lcad K_M for C16Enoyl-CoA
$K_{M\,lcad}^{C14CoAm}$	7.4	µM	lcad K_M for C14-CoA
$K_{M\,lcad}^{C14enoCoAm}$	1.08	µM	lcad K_M for C14enoyl-CoA
$K_{M\,lcad}^{C12CoAm}$	9.0	µM	lcad K_M for C12-CoA
$K_{M\,lcad}^{C12enoCoAm}$	1.08	µM	lcad K_M for C12enoyl-CoA
$K_{M\,vlcad}^{C10CoAm}$	24.3	µM	lcad K_M for C10-CoA
$K_{M\,vlcad}^{C10enoCoAm}$	1.08	µM	lcad K_M for C10enoyl-CoA
$K_{M\,vlcad}^{C8CoAm}$	12.3	µM	lcad K_M for C8-CoA
$K_{M\,vlcad}^{C8enoCoAm}$	1.08	µM	lcad K_M for C8-enoyl-CoA
K_{EQ}^{lcad}	6.0		lcad equilibrium constant
sf_{mcad}^{C12}	0.68		Scaling factor of medium-chain acyl CoA dehydrogenase (mcad) for C12CoA
sf_{mcad}^{C10}	0.8		mcad scaling factor for C10CoA
sf_{mcad}^{C8}	0.87		mcad scaling factor for C8CoA
sf_{mcad}^{C6}	1.0		mcad scaling factor for C6CoA
sf_{mcad}^{C4}	0.12		mcad scaling factor for C4CoA

Symbol	Value	Units	Description
V_{mcad}	0.25	mM ms^{-1}	mcad maximal rate
$K_{M\,mcad}^{ETFm}$	1.2	nM	mcad K_M for FAD
$K_{M\,mcad}^{ETFH_2m}$	24.2	µM	mcad K_M for $FADH_2$
$K_{M\,mcad}^{C12CoAm}$	5.7	µM	mcad K_M for C12CoA
$K_{M\,mcad}^{C12enoCoAm}$	1.08	µM	mcad K_M for C12Enoyl-CoA
$K_{M\,mcad}^{C10CoAm}$	5.4	µM	mcad K_M for C10-CoA
$K_{M\,mcad}^{C10enoCoAm}$	1.08	µM	mcad K_M for C10enoyl-CoA
$K_{M\,mcad}^{C8CoAm}$	4.0	µM	mcad K_M for C8-CoA
$K_{M\,mcad}^{C8enoCoAm}$	1.08	µM	mcad K_M for C8enoyl-CoA
$K_{M\,mcad}^{C6CoAm}$	9.4	µM	mcad K_M for C6-CoA
$K_{M\,mcad}^{C6enoCoAm}$	1.08	µM	mcad K_M for C6enoyl-CoA
$K_{M\,mcad}^{C4CoAm}$	135	µM	mcad K_M for C4-CoA
$K_{M\,mcad}^{C4enoCoAm}$	1.08	µM	mcad K_M for C4enoyl-CoA
K_{EQ}^{mcad}	6.0		mcad equilibrium constant
sf_{scad}^{C6}	0.3		Scaling factor of short-chain acyl CoA dehydrogenase (scad) for C6CoA
sf_{scad}^{C4}	1.0		scad scaling factor for C4CoA
V_{scad}	0.25	mM ms^{-1}	scad maximal rate
$K_{M\,scad}^{ETFm}$	0.12	µM	scad K_M for FAD
$K_{M\,scad}^{ETFH_2m}$	24.2	µM	scad K_M for $FADH_2$
$K_{M\,scad}^{C6CoAm}$	285	µM	scad K_M for C6-CoA
$K_{M\,mcad}^{C6enoCoAm}$	1.08	µM	scad K_M for C6Enoyl-CoA
$K_{M\,scad}^{C4CoAm}$	10.7	µM	scad K_M for C4-CoA
$K_{M\,scad}^{C4enoCoAm}$	1.08	µM	scad K_M for C4Enoyl-CoA
K_{EQ}^{scad}	6.0		scad equilibrium constant

V_{vlcad} , V_{lcad} , V_{mcad} and V_{scad} were adjusted following the criteria that they should reproduce palmitate oxidation fluxes reported in adult cardiomyocytes (Mazumder et al., 2004).

Fatty acid enoyl-CoA hydratase (Crotonase, CRO)

The enzyme rate expressions were modelled as single substrate reversible reactions, with the various enoyl CoA of different chain length and hydroxyacyl CoA competing with one another (van Eunen et al., 2013).

$$V_{cro}^{C16} = sf_{cro}^{C16} . V_{cro} \frac{\left(\frac{C16enoCoA_m}{K_{M\,cro}^{C16enoCoAm}}\right) - \left(\frac{C16OHCoA_m}{K_{M\,cro}^{C16enoCoAm} K_{EQ}^{cro}}\right)}{V_{cro}^{DENOMINATOR}} \tag{S161}$$

$$V_{cro}^{C14} = sf_{cro}^{C14} . V_{cro} \frac{\left(\frac{C14enoCoA_m}{K_{M\,cro}^{C14enoCoAm}}\right) - \left(\frac{C14OHCoA_m}{K_{M\,cro}^{C14enoCoAm} K_{EQ}^{cro}}\right)}{V_{cro}^{DENOMINATOR}} \tag{S162}$$

$$V_{cro}^{C12} = sf_{cro}^{C12} . V_{cro} \frac{\left(\frac{C12enoCoA_m}{K_{M\,cro}^{C12enoCoAm}}\right) - \left(\frac{C12OHCoA_m}{K_{M\,cro}^{C12enoCoAm} K_{EQ}^{cro}}\right)}{V_{cro}^{DENOMINATOR}} \tag{S163}$$

$$V_{cro}^{C10} = sf_{cro}^{C10} . V_{cro} \frac{\left(\frac{C10enoCoA_m}{K_{M\,cro}^{C10enoCoAm}}\right) - \left(\frac{C10OHCoA_m}{K_{M\,cro}^{C10enoCoAm} K_{EQ}^{cro}}\right)}{V_{cro}^{DENOMINATOR}} \tag{S164}$$

$$V_{cro}^{C8} = sf_{cro}^{C8} . V_{cro} \frac{\left(\frac{C8enoCoA_m}{K_{M\,cro}^{C8enoCoAm}}\right) - \left(\frac{C8OHCoA_m}{K_{M\,cro}^{C8enoCoAm} K_{EQ}^{cro}}\right)}{V_{cro}^{DENOMINATOR}} \tag{S165}$$

$$V_{cro}^{C6} = sf_{cro}^{C6} . V_{cro} \frac{\left(\frac{C6enoCoA_m}{K_{M\,cro}^{C6enoCoAm}}\right) - \left(\frac{C6OHCoA_m}{K_{M\,cro}^{C6enoCoAm} K_{EQ}^{cro}}\right)}{V_{cro}^{DENOMINATOR}} \tag{S166}$$

$$V_{cro}^{C4} = sf_{cro}^{C4} . V_{cro} \frac{\left(\frac{C4enoCoA_m}{K_{M\,cro}^{C4enoCoAm}}\right) - \left(\frac{C4OHCoA_m}{K_{M\,cro}^{C4enoCoAm} K_{EQ}^{cro}}\right)}{V_{cro}^{DENOMINATOR}} \tag{S167}$$

$$V_{cro}^{DENOMINATOR} = \begin{pmatrix} 1 + \frac{C16enoCoA_m}{K_{M\,cro}^{C16enoCoAm}} + \frac{C16OHCoA_m}{K_{M\,cro}^{C16OHCoAm}} + \frac{C14enoCoA_m}{K_{M\,cro}^{C14enoCoAm}} + \frac{C14OHCoA_m}{K_{M\,cro}^{C14OHCoAm}} + \cdots \\ \frac{C12enoCoA_m}{K_{M\,cro}^{C12enoCoAm}} + \frac{C12OHCoA_m}{K_{M\,cro}^{C12OHCoAm}} + \frac{C10enoCoA_m}{K_{M\,cro}^{C10enoCoAm}} + \frac{C10OHCoA_m}{K_{M\,cro}^{C10OHCoAm}} + \cdots \\ \frac{C8enoCoA_m}{K_{M\,cro}^{C8enoCoAm}} + \frac{C8OHCoA_m}{K_{M\,cro}^{C8OHCoAm}} + \frac{C6enoCoA_m}{K_{M\,cro}^{C6enoCoAm}} + \frac{C6OHCoA_m}{K_{M\,cro}^{C6OHCoAm}} + \cdots \\ \frac{C4enoCoA_m}{K_{M\,cro}^{C4enoCoAm}} + \frac{C4OHCoA_m}{K_{M\,cro}^{C4OHCoAm}} \end{pmatrix} + \frac{AceAcCoA_m}{K_{i\,cro}^{AcAcCoAm}}$$

1.2.18 Supplementary Table S10. Parameters for fatty acylenoyl-CoA hydratase (cro)

Symbol	Value	Units	Description
sf_{cro}^{C16}	0.13		cro scaling factor for C16enoylCoA
sf_{cro}^{C14}	0.2		cro scaling factor for C14enoylCoA
sf_{cro}^{C12}	0.25		cro scaling factor for C12enoylCoA
sf_{cro}^{C10}	0.33		cro scaling factor for C10enoylCoA
sf_{cro}^{C8}	0.58		cro scaling factor for C8enoylCoA
sf_{cro}^{C6}	0.8		cro scaling factor for C6enoylCoA
sf_{cro}^{C4}	1.0		cro scaling factor for C4enoylCoA
V_{cro}	0.27	mM ms^{-1}	cro maximal rate
$K_{M\,cro}^{C16enoCoAm}$	0.15	mM	cro Michaelis constant (K_M) for C16Enoyl-CoA
$K_{M\,cro}^{C16OHCoAm}$	45	µM	cro K_M for C16OH-CoA
$K_{M\,cro}^{C14enoCoAm}$	0.1	mM	cro K_M for C14Enoyl-CoA
$K_{M\,cro}^{C14OHCoAm}$	45	µM	cro K_M for C14OH-CoA
$K_{M\,cro}^{C12enoCoAm}$	25	µM	cro K_M for C12Enoyl-CoA
$K_{M\,cro}^{C12OHCoAm}$	45	µM	cro K_M for C12OH-CoA
$K_{M\,cro}^{C10enoCoAm}$	25	µM	cro K_M for C10Enoyl-CoA
$K_{M\,cro}^{C10OHCoAm}$	45	µM	cro K_M for C10OH-CoA
$K_{M\,cro}^{C8enoCoAm}$	25	µM	cro K_M for C8Enoyl-CoA

Symbol	Value	Units	Description
$K_{M\,cro}^{C8OHCoAm}$	45	μM	cro K_M for C8OH-CoA
$K_{M\,cro}^{C6enoCoAm}$	25	μM	cro K_M for C6Enoyl-CoA
$K_{M\,cro}^{C6OHCoAm}$	45	μM	cro K_M for C6OH-CoA
$K_{M\,cro}^{C4enoCoAm}$	40	μM	cro K_M for C4Enoyl-CoA
$K_{M\,cro}^{C4OHCoAm}$	45	μM	cro K_M for C4OH-CoA
K_{EQ}^{cro}	3.13		cro equilibrium constant
$K_{i\,cro}^{AcAcCoAm}$	1.6	μM	cro inhibition constant for AcetoAcCoA

All parameter values were taken from(Cortassa et al., 2017a) and (van Eunen et al., 2013).

Medium/short-chain hydroxyacyl-CoA dehydrogenase (mschad)

The enzyme's rate expressions were modelled as bi-reactant reversible reactions, coupled to the reduction of NAD$^+$ and with the various hydroxyacyl CoA and ketoacyl CoA competing for the same enzyme pool (van Eunen et al., 2013).

$$V_{mschad}^{C16} = sf_{mschad}^{C16} \cdot V_{mschad} \frac{\left(\frac{C16OHCoA_m}{K_{M\,mschad}^{C16OHCoAm}} \cdot \frac{NAD_m}{K_{M\,mschad}^{NADm}}\right) - \left(\frac{C16ketoCoA_m}{K_{M\,mschad}^{C16OHCoAm}} \cdot \frac{NADH_m}{K_{M\,mschad}^{NADm}\, K_{EQ}^{mschad}}\right)}{V_{MSCHAD}^{DENOMINATOR}} \quad \text{(S168)}$$

$$V_{mschad}^{C14} = sf_{mschad}^{C14} \cdot V_{mschad} \frac{\left(\frac{C14OHCoA_m}{K_{M\,mschad}^{C14OHCoAm}} \cdot \frac{NAD_m}{K_{M\,mschad}^{NADm}}\right) - \left(\frac{C14ketoCoA_m}{K_{M\,mschad}^{C14OHCoAm}} \cdot \frac{NADH_m}{K_{M\,mschad}^{NADm}\, K_{EQ}^{mschad}}\right)}{V_{MSCHAD}^{DENOMINATOR}} \quad \text{(S169)}$$

$$V_{mschad}^{C12} = sf_{mschad}^{C12} \cdot V_{mschad} \frac{\left(\frac{C12OHCoA_m}{K_{M\,mschad}^{C12OHCoAm}} \cdot \frac{NAD_m}{K_{M\,mschad}^{NADm}}\right) - \left(\frac{C12ketoCoA_m}{K_{M\,mschad}^{C12OHCoAm}} \cdot \frac{NADH_m}{K_{M\,mschad}^{NADm}\, K_{EQ}^{mschad}}\right)}{V_{MSCHAD}^{DENOMINATOR}} \quad \text{(S170)}$$

$$V_{mschad}^{C10} = sf_{mschad}^{C10} \cdot V_{mschad} \frac{\left(\frac{C10OHCoA_m}{K_{M\,mschad}^{C10OHCoAm}} \cdot \frac{NAD_m}{K_{M\,mschad}^{NADm}}\right) - \left(\frac{C10ketoCoA_m}{K_{M\,mschad}^{C10OHCoAm}} \cdot \frac{NADH_m}{K_{M\,mschad}^{NADm}\, K_{EQ}^{mschad}}\right)}{V_{MSCHAD}^{DENOMINATOR}} \quad \text{(S171)}$$

$$V_{mschad}^{C8} = sf_{mschad}^{C8} \cdot V_{mschad} \frac{\left(\frac{C8OHCoA_m}{K_{M\,mschad}^{C8OHCoAm}} \cdot \frac{NAD_m}{K_{M\,mschad}^{NADm}}\right) - \left(\frac{C8ketoCoA_m}{K_{M\,mschad}^{C8OHCoAm}} \cdot \frac{NADH_m}{K_{M\,mschad}^{NADm}\, K_{EQ}^{mschad}}\right)}{V_{MSCHAD}^{DENOMINATOR}} \quad \text{(S172)}$$

$$V_{mschad}^{C6} = sf_{mschad}^{C6} \cdot V_{mschad} \frac{\left(\frac{C6OHCoA_m}{K_{M\ mschad}^{C6OHCoAm}} \cdot \frac{NAD_m}{K_{M\ mschad}^{NADm}}\right) - \left(\frac{C6ketoCoA_m}{K_{M\ mschad}^{C6OHCoAm}} \cdot \frac{NADH_m}{K_{M\ mschad}^{NADm} K_{EQ}^{mschad}}\right)}{V_{MSCHAD}^{DENOMINATOR}} \tag{S173}$$

$$V_{mschad}^{C4} = sf_{mschad}^{C4} \cdot V_{mschad} \frac{\left(\frac{C4OHCoA_m}{K_{M\ mschad}^{C4OHCoAm}} \cdot \frac{NAD_m}{K_{M\ mschad}^{NADm}}\right) - \left(\frac{C4ketoCoA_m}{K_{M\ mschad}^{C4OHCoAm}} \cdot \frac{NADH_m}{K_{M\ mschad}^{NADm} K_{EQ}^{mschad}}\right)}{V_{MSCHAD}^{DENOMINATOR}} \tag{S174}$$

$$V_{MSCHAD}^{DENOMINATOR} = \left(\begin{array}{l} 1 + \frac{C16OHCoA_m}{K_{M\ mschad}^{C16OHCoAm}} + \frac{C16ketoCoA_m}{K_{M\ mschad}^{C16ketoCoAm}} + \frac{C14OHCoA_m}{K_{M\ mschad}^{C14OHCoAm}} + \frac{C14ketoCoA_m}{K_{M\ mschad}^{C14ketoCoAm}} + \cdots \\ \frac{C12OHCoA_m}{K_{M\ mschad}^{C12OHCoAm}} + \frac{C12ketoCoA_m}{K_{M\ mschad}^{C12ketoCoAm}} + \frac{C10OHCoA_m}{K_{M\ mschad}^{C10OHCoAm}} + \frac{C10ketoCoA_m}{K_{M\ mschad}^{C10ketoCoAm}} + \cdots \\ \frac{C8OHCoA_m}{K_{M\ mschad}^{C8OHCoAm}} + \frac{C8ketoCoA_m}{K_{M\ mschad}^{C8ketoCoAm}} + \frac{C6OHCoA_m}{K_{M\ mschad}^{C6OHCoAm}} + \frac{C6ketoCoA_m}{K_{M\ mschad}^{C6ketoCoAm}} + \cdots \\ \frac{C4OHCoA_m}{K_{M\ mschad}^{C4OHCoAm}} + \frac{C4ketoCoA_m}{K_{M\ mschad}^{C4ketoCoAm}} \end{array}\right) \left(1 + \frac{NAD_m}{K_{M\ mschad}^{NADm}} + \frac{NADH_m}{K_{M\ mschad}^{NADHm}}\right)$$

1.2.19 Supplementary Table S11. Parameter values for medium/short-chain hydroxyacyl-CoA dehydrogenase (mschad)

Symbol	Value	Units	Description
sf_{mschad}^{C16}	0.6		Scaling factor of mschad for C16OHCoA
sf_{mschad}^{C14}	0.5		Scaling factor of mschad for C14OHCoA
sf_{mschad}^{C12}	0.43		Scaling factor of mschad for C12OHCoA
sf_{mschad}^{C10}	0.64		Scaling factor of mschad for C10OHCoA
sf_{mschad}^{C8}	0.89		Scaling factor of mschad for C8OHCoA
sf_{mschad}^{C6}	1.0		Scaling factor of mschad for C6OHCoA
sf_{mschad}^{C4}	0.67		Scaling factor of mschad for C4OHCoA
V_{mschad}	0.5	mM ms^{-1}	mschad maximal rate
$K_{M\ mschad}^{C16OHCoAm}$	1.5	μM	mschad K_M for C16OHCoA
$K_{M\ mschad}^{C16ketoCoAm}$	1.4	μM	mschad K_M for C16ketoCoA
$K_{M\ mschad}^{C14OHCoAm}$	1.8	μM	mschad K_M for C14OHCoA
$K_{M\ mschad}^{C14ketoCoAm}$	1.4	μM	mschad K_M for C14ketoCoA

Symbol	Value	Units	Description
$K_{M\,mschad}^{C12OHCoAm}$	3.7	µM	mschad K_M for C12OHCoA
$K_{M\,mschad}^{C12ketoCoAm}$	1.6	µM	mschad K_M for C12ketoCoA
$K_{M\,mschad}^{C10OHCoAm}$	8.8	µM	mschad K_M for C10OHCoA
$K_{M\,mschad}^{C10ketoCoAm}$	2.3	µM	mschad K_M for C10ketoCoA
$K_{M\,mschad}^{C8OHCoAm}$	16.3	µM	mschad K_M for C8OHCoA
$K_{M\,mschad}^{C8ketoCoAm}$	4.1	µM	mschad K_M for C8ketoCoA
$K_{M\,mschad}^{C6OHCoAm}$	28.6	µM	mschad K_M for C6OHCoA
$K_{M\,mschad}^{C6ketoCoAm}$	5.8	µM	mschad K_M for C6ketoCoA
$K_{M\,mschad}^{C4OHCoAm}$	69.9	µM	mschad K_M for C4OHCoA
$K_{M\,mschad}^{C4ketoCoAm}$	16.9	µM	mschad K_M for C4ketoCoA
$K_{M\,mschad}^{NADm}$	58.5	µM	mschad K_M for NAD^+
$K_{M\,mschad}^{NADHm}$	5.4	µM	mschad K_M for NADH
K_{EQ}^{mschad}	2.17×10^{-4}		mschad equilibrium constant

V_{mschad} was adjusted following the same criteria indicated in Supplementary Material Table 9. Other parameters were taken from (van Eunen et al., 2013).

Medium-chain ketoacyl-CoA thiolase (MCKAT)

The cleavage step rate expressions were modelled as bi-reactant reversible reactions, producing Acyl CoA and requiring CoASH as substrate (van Eunen et al., 2013).

$$V_{mckat}^{C16} = sf_{mckat}^{C16} \cdot V_{mckat} \frac{\left(\frac{C16ketoCoA_m}{K_{M\,mckat}^{C16ketoCoAm}} \cdot \frac{CoA_m}{K_{M\,mckat}^{CoAm}}\right) - \left(\frac{C14CoA_m}{K_{M\,mckat}^{C16ketoCoAm}} \cdot \frac{AcCoA_m}{K_{M\,mckat}^{CoAm}\,K_{EQ}^{mckat}}\right)}{V_{MCKAT}^{DENOMINATOR}} \quad \text{(S175)}$$

$$V_{mckat}^{C14} = sf_{mckat}^{C14} \cdot V_{mckat} \frac{\left(\frac{C14ketoCoA_m}{K_{M\,mckat}^{C14ketoCoAm}} \cdot \frac{CoA_m}{K_{M\,mckat}^{CoAm}}\right) - \left(\frac{C12CoA_m}{K_{M\,mckat}^{C14ketoCoAm}} \cdot \frac{AcCoA_m}{K_{M\,mckat}^{CoAm}\,K_{EQ}^{mckat}}\right)}{V_{MCKAT}^{DENOMINATOR}} \quad \text{(S176)}$$

$$V_{mckat}^{C12} = sf_{mckat}^{C12} \cdot V_{mckat} \frac{\left(\frac{C12ketoCoA_m}{K_{M\,mckat}^{C12ketoCoAm}} \cdot \frac{CoA_m}{K_{M\,mckat}^{CoAm}}\right) - \left(\frac{C10CoA_m}{K_{M\,mckat}^{C12ketoCoAm}} \cdot \frac{AcCoA_m}{K_{M\,mckat}^{CoAm} K_{EQ}^{mckat}}\right)}{V_{MCKAT}^{DENOMINATOR}} \quad \text{(S177)}$$

$$V_{mckat}^{C10} = sf_{mckat}^{C10} \cdot V_{mckat} \frac{\left(\frac{C10ketoCoA_m}{K_{M\,mckat}^{C10ketoCoAm}} \cdot \frac{CoA_m}{K_{M\,mckat}^{CoAm}}\right) - \left(\frac{C8CoA_m}{K_{M\,mckat}^{C10ketoCoAm}} \cdot \frac{AcCoA_m}{K_{M\,mckat}^{CoAm} K_{EQ}^{mckat}}\right)}{V_{MCKAT}^{DENOMINATOR}} \quad \text{(S178)}$$

$$V_{mckat}^{C8} = sf_{mckat}^{C8} \cdot V_{mckat} \frac{\left(\frac{C8ketoCoA_m}{K_{M\,mckat}^{C8ketoCoAm}} \cdot \frac{CoA_m}{K_{M\,mckat}^{CoAm}}\right) - \left(\frac{C6CoA_m}{K_{M\,mckat}^{C8ketoCoAm}} \cdot \frac{AcCoA_m}{K_{M\,mckat}^{CoAm} K_{EQ}^{mckat}}\right)}{V_{MCKAT}^{DENOMINATOR}} \quad \text{(S179)}$$

$$V_{mckat}^{C6} = sf_{mckat}^{C6} \cdot V_{mckat} \frac{\left(\frac{C6ketoCoA_m}{K_{M\,mckat}^{C6ketoCoAm}} \cdot \frac{CoA_m}{K_{M\,mckat}^{CoAm}}\right) - \left(\frac{C4CoA_m}{K_{M\,mckat}^{C6ketoCoAm}} \cdot \frac{AcCoA_m}{K_{M\,mckat}^{CoAm} K_{EQ}^{mckat}}\right)}{V_{MCKAT}^{DENOMINATOR}} \quad \text{(S180)}$$

$$V_{mckat}^{C4} = sf_{mckat}^{C4} \cdot V_{mckat} \frac{\left(\frac{C4ketoCoA_m}{K_{M\,mckat}^{C4ketoCoAm}} \cdot \frac{CoA_m}{K_{M\,mckat}^{CoAm}}\right) - \left(\frac{AcCoA_m}{K_{M\,mckat}^{C4ketoCoAm}} \cdot \frac{AcCoA_m}{K_{M\,mckat}^{CoAm} K_{EQ}^{mckat}}\right)}{V_{MCKAT}^{DENOMINATOR}} \quad \text{(S181)}$$

$$V_{MCKAT}^{DENOMINATOR} = \left(\begin{array}{l} 1 + \frac{C16ketoCoA_m}{K_{M\,mckat}^{C16ketoCoAm}} + \frac{C14CoA_m}{K_{M\,mckat}^{C14CoAm}} + \frac{C14ketoCoA_m}{K_{M\,mckat}^{C14ketoCoAm}} + \frac{C12CoA_m}{K_{M\,mckat}^{C12CoAm}} + \cdots \\ \frac{C12ketoCoA_m}{K_{M\,mckat}^{C12ketoCoAm}} + \frac{C10CoA_m}{K_{M\,mckat}^{C10CoAm}} + \frac{C10ketoCoA_m}{K_{M\,mckat}^{C10ketoCoAm}} + \frac{C8CoA_m}{K_{M\,mckat}^{C8CoAm}} + \cdots \\ \frac{C8ketoCoA_m}{K_{M\,mckat}^{C8ketoCoAm}} + \frac{C6CoA_m}{K_{M\,mckat}^{C6CoAm}} + \frac{C6ketoCoA_m}{K_{M\,mckat}^{C6ketoCoAm}} + \frac{C4CoA_m}{K_{M\,mckat}^{C4CoAm}} + \cdots \\ \frac{C4ketoCoA_m}{K_{M\,mckat}^{C4ketoCoAm}} + \frac{AcCoA_m}{K_{M\,mckat}^{AcCoAm}} \end{array}\right) \left(1 + \frac{CoA_m}{K_{M\,mckat}^{CoAm}} + \frac{AcCoA_m}{K_{M\,mckat}^{AcCoAm}}\right)$$

1.2.20 Supplementary Table S12. Parameters for medium-chain ketoacyl-CoA thiolase (mckat)

Symbol	Value	Units	Description
sf_{mckat}^{C16}	0.2		mckat scaling factor for C16ketoCoA
sf_{mckat}^{C14}	0.2		mckat scaling factor for C14ketoCoA
sf_{mckat}^{C12}	0.38		mckat scaling factor for C12ketoCoA
sf_{mckat}^{C10}	0.75		mckat scaling factor for C10ketoCoA

Symbol	Value	Units	Description
sf^{C8}_{mckat}	0.81		mckat scaling factor for C8ketoCoA
sf^{C6}_{mckat}	1.0		mckat scaling factor for C6ketoCoA
sf^{C4}_{mckat}	0.49		mckat scaling factor for C4ketoCoA
V_{mckat}	0.1884	mM ms^{-1}	mckat maximal rate
$K^{CoAm}_{M\ mckat}$	26.6	μM	mckat Michaelis constant (K_M) for CoA
$K^{AcCoAm}_{M\ mckat}$	30		mckat K_M for AcCoA
$K^{C16ketoCoAm}_{M\ mckat}$	1.1	μM	mckat K_M for C16ketoCoA
$K^{C14CoAm}_{M\ mckat}$	13.83	μM	mckat K_M for C14CoA
$K^{C14ketoCoAm}_{M\ mckat}$	1.2	μM	mckat K_M for C14ketoCoA
$K^{C12CoAm}_{M\ mckat}$	13.83	μM	mckat K_M for C12CoA
$K^{C12ketoCoAm}_{M\ mckat}$	1.3	μM	mckat K_M for C12ketoCoA
$K^{C10CoAm}_{M\ mckat}$	13.83	μM	mckat K_M for C10CoA
$K^{C10ketoCoAm}_{M\ mckat}$	2.1	μM	mckat K_M for C10ketoCoA
$K^{C8CoAm}_{M\ mckat}$	13.83	μM	mckat K_M for C8CoA
$K^{C8ketoCoAm}_{M\ mckat}$	3.2	μM	mckat K_M for C8ketoCoA
$K^{C6CoAm}_{M\ mckat}$	13.83	μM	mckat K_M for C6CoA
$K^{C6ketoCoAm}_{M\ mckat}$	6.7	μM	mckat K_M for C6ketoCoA
$K^{C4CoAm}_{M\ mckat}$	13.83	μM	mckat K_M for C4CoA
$K^{C4ketoCoAm}_{M\ mckat}$	12.4	μM	mckat K_M for C4ketoCoA
K^{mckat}_{EQ}	1051		mckat equilibrium constant

V_{mschad} was adjusted following the same criteria indicated in Supplementary Material Table 9. Other parameters were taken from (van Eunen et al., 2013).

Mitochondrial trifunctional protein (mtp)

The multienzyme complex encompassing the steps catalyzed by acylenoyl-CoA hydratase, hydroxyacyl-CoA dehydrogenase and the ketoacyl-CoA thiolase was modeled as a terreactant

reversible reaction, using enoyl CoA intermediates, NAD$^+$ and CoASH and generating AcylCoA, NADH and AcCoA.

$$V_{mtp}^{C16} = sf_{mtp}^{C16}.V_{mtp}\frac{\left(\frac{C16enoCoA_m}{K_{M\,mtp}^{C16enoCoAm}}.\frac{CoA_m}{K_{M\,mtp}^{CoAm}}.\frac{NAD_m}{K_{M\,mtp}^{NADm}}\right)-\left(\frac{C14CoA_m}{K_{M\,mtp}^{C16enoCoAm}}.\frac{NADH_m}{K_{M\,mtp}^{NADm}}.\frac{AcCoA_m}{K_{M\,mtp}^{CoAm}K_{EQ}^{mtp}}\right)}{V_{MTP}^{DENOMINATOR}} \quad (S182)$$

$$V_{mtp}^{C14} = sf_{mtp}^{C14}.V_{mtp}\frac{\left(\frac{C14enoCoA_m}{K_{M\,mtp}^{C14enoCoAm}}.\frac{CoA_m}{K_{M\,mtp}^{CoAm}}.\frac{NAD_m}{K_{M\,mtp}^{NADm}}\right)-\left(\frac{C12CoA_m}{K_{M\,mtp}^{C14enoCoAm}}.\frac{NADH_m}{K_{M\,mtp}^{NADm}}.\frac{AcCoA_m}{K_{M\,mtp}^{CoAm}K_{EQ}^{mtp}}\right)}{V_{MTP}^{DENOMINATOR}} \quad (S183)$$

$$V_{mtp}^{C12} = sf_{mtp}^{C12}.V_{mtp}\frac{\left(\frac{C12enoCoA_m}{K_{M\,mtp}^{C12enoCoAm}}.\frac{CoA_m}{K_{M\,mtp}^{CoAm}}.\frac{NAD_m}{K_{M\,mtp}^{NADm}}\right)-\left(\frac{C10CoA_m}{K_{M\,mtp}^{C12enoCoAm}}.\frac{NADH_m}{K_{M\,mtp}^{NADm}}.\frac{AcCoA_m}{K_{M\,mtp}^{CoAm}K_{EQ}^{mtp}}\right)}{V_{MTP}^{DENOMINATOR}} \quad (S184)$$

$$V_{mtp}^{C10} = sf_{mtp}^{C10}.V_{mtp}\frac{\left(\frac{C10enoCoA_m}{K_{M\,mtp}^{C10enoCoAm}}.\frac{CoA_m}{K_{M\,mtp}^{CoAm}}.\frac{NAD_m}{K_{M\,mtp}^{NADm}}\right)-\left(\frac{C8CoA_m}{K_{M\,mtp}^{C10enoCoAm}}.\frac{NADH_m}{K_{M\,mtp}^{NADm}}.\frac{AcCoA_m}{K_{M\,mtp}^{CoAm}K_{EQ}^{mtp}}\right)}{V_{MTP}^{DENOMINATOR}} \quad (S185)$$

$$V_{mtp}^{C8} = sf_{mtp}^{C8}.V_{mtp}\frac{\left(\frac{C8enoCoA_m}{K_{M\,mtp}^{C8enoCoAm}}.\frac{CoA_m}{K_{M\,mtp}^{CoAm}}.\frac{NAD_m}{K_{M\,mtp}^{NADm}}\right)-\left(\frac{C6CoA_m}{K_{M\,mtp}^{C8enoCoAm}}.\frac{NADH_m}{K_{M\,mtp}^{NADm}}.\frac{AcCoA_m}{K_{M\,mtp}^{CoAm}K_{EQ}^{mtp}}\right)}{V_{MTP}^{DENOMINATOR}} \quad (S186)$$

$$V_{MTP}^{DENOMINATOR} = \left(\begin{array}{l} 1+\frac{C16enoCoA_m}{K_{M\,mtp}^{C16enoCoAm}}+\frac{C14CoA_m}{K_{M\,mtp}^{C14CoAm}}+\frac{C14enoCoA_m}{K_{M\,mtp}^{C14enoCoAm}}+\cdots \\ \frac{C12CoA_m}{K_{M\,mtp}^{C12CoAm}}+\frac{C12enoCoA_m}{K_{M\,mtp}^{C12enoCoAm}}+\frac{C10CoA_m}{K_{M\,mtp}^{C10CoAm}}+\cdots \\ \frac{C10enoCoA_m}{K_{M\,mtp}^{C10enoCoAm}}+\frac{C8CoA_m}{K_{M\,mtp}^{C8CoAm}}\frac{C8enoCoA_m}{K_{M\,mtp}^{C8enoCoAm}}+\cdots \\ \frac{C6CoA_m}{K_{M\,mtp}^{C6CoAm}}+\frac{AcetoAcCoA_m}{K_{i\,mtp}^{AcAcCoAm}} \end{array}\right)\left(1+\frac{CoA_m}{K_{M\,mtp}^{CoAm}}+\frac{AcCoA_m}{K_{M\,mtp}^{AcCoAm}}\right)\left(1+\frac{NAD_m}{K_{M\,mtp}^{NADm}}+\frac{NADH_m}{K_{M\,mtp}^{NADH\,m}}\right)$$

1.2.21 Supplementary Table S13. Parameters for mitochondrial trifunctional protein (mtp)

Symbol	Value	Units	Description
sf_{mtp}^{C16}	1.5		mtp scaling factor for C16enoylCoA
sf_{mto}^{C14}	0.9		mtp scaling factor for C14enoylCoA

Symbol	Value	Units	Description
sf_{mtp}^{C12}	0.81		mtp scaling factor for C12enoylCoA
sf_{mtp}^{C10}	0.73		mtp scaling factor for C10enoylCoA
sf_{mtp}^{C8}	0.64		mtp scaling factor for C8enoylCoA
V_{mtp}	0.142	mM ms^{-1}	mtp maximal rate
$K_{M\,mtp}^{NAD\ m}$	60	μM	mtp Michaelis constant (K_M) for NAD^+
$K_{M\,mtp}^{NADH\,m}$	50	μM	mtp K_M for NADH
$K_{M\,mtp}^{CoA\,m}$	30	μM	mtp K_M for CoA
$K_{M\,mtp}^{AcCoA\,m}$	30	μM	mtp K_M for AcCoA
$K_{M\,mtp}^{C16enoCoA\,m}$	25	μM	mtp K_M for C16 enoylCoA
$K_{M\,mtp}^{C14enoCoA\,m}$	25	μM	mtp K_M for C14 enoylCoA
$K_{M\,mtp}^{C12enoCoA\,m}$	25	μM	mtp K_M for C12 enoylCoA
$K_{M\,mtp}^{C10enoCoA\,m}$	25	μM	mtp K_M for C10 enoylCoA
$K_{M\,mtp}^{C8enoCoA\,m}$	25	μM	mtp K_M for C8 enoylCoA
$K_{M\,mtp}^{C14CoA\,m}$	13.83	μM	mtp K_M for C14 CoA
$K_{M\,mtp}^{C12CoA\,m}$	13.83	μM	mtp K_M for C12 CoA
$K_{M\,mtp}^{C10CoA\,m}$	13.83	μM	mtp K_M for C10 CoA
$K_{M\,mtp}^{C8CoA\,m}$	13.83	μM	mtp K_M for C8 CoA
$K_{M\,mtp}^{C6CoA\,m}$	13.83	μM	mtp K_M for C6 CoA
$K_{M\,mtp}^{AcAcCoA\,m}$	30	μM	mtp inhibition constant by AcAcCoA
K_{EQ}^{mtp}	0.71		mtp equilibrium constant
$CoASH_T$	1.0	mM	Sum of mitochondrial CoA species
FAD_T	0.7	mM	Total concentration of flavin in ETF

V_{mtp} was adjusted following the same criteria indicated in Supplementary Material Table 9. Other parameters were taken from (van Eunen et al., 2013).

In addition to the conservation relation for NAD$^+$/NADH, NADP$^+$/NADPH and antioxidant intermediates glutathione and thioredoxin (see below), β-oxidation introduces two conservation relations for species carrying a Coenzyme A group and FAD/FADH2, indicated as follows:

$$\begin{aligned} CoASH = CoASH_T - AcCoA - SCoA - C16CoA - C16enoCoA - C16OHCoA - C16ketoCoA \cdots \\ - C14CoA - C14enoCoA - C14OHCoA - C14ketoCoA - C12CoA - C12enoCoA \cdots \\ - C12OHCoA - C12ketoCoA - C10CoA - C10enoCoA - C10OHCoA - C10ketoCoA \cdots \\ - C8CoA - C8enoCoA - C8OHCoA - C8ketoCoA - C6CoA - C6enoCoA - C6OHCoA \cdots \\ - C6ketoCoA - C4CoA - C4enoCoA - C4OHCoA - C4ketoCoA \end{aligned} \tag{S187}$$

$$FAD = FAD_T - FADH_2 \tag{S188}$$

1.2.22 Redox balance, ROS transport and scavenging

$$V_{IMAC} = \left(a + \frac{b}{1 + \frac{K_{cc}}{[\mathrm{O}_2^{\bullet -}]_i}} \right) \left(GL + \frac{G_{max}}{1 + e^{\left(K\left(\Delta\Psi_m^b \right) + \Delta\Psi_m \right)}} \right) \Delta\Psi_m \tag{S189}$$

$$V_{ROS}^{Tr} = j \frac{V_{IMAC}}{\Delta\Psi_m} \left(-\Delta\Psi_m - \frac{RT}{F} \log\left(\frac{[\mathrm{O}_2^{\bullet -}]_m}{[\mathrm{O}_2^{\bullet -}]_i} \right) \right) \tag{S190}$$

1.2.23 Supplementary Table S14. Parameters used in ROS transport

Symbol	Value	Units	Description	Reference
a	1×10^{-3}		Basal IMAC conductance	(Cortassa et al., 2004)
b	1×10^{4}		Activation factor by cytoplasmic $O_2^{\cdot -}$	(Cortassa et al., 2004)
K_{cc}	1×10^{-2}	mM	Activation constant by cytoplasmic $O_2^{\cdot -}$	(Cortassa et al., 2004)
GL	3.5×10^{-8}		IMAC integral conductance	(Cortassa et al., 2004)
G_{max}	3.9085×10^{-6}		IMAC leak conductance at saturation	(Cortassa et al., 2004)
K	7.0×10^{-2}	mV^{-1}	Steepness factor	(Cortassa et al., 2004)
$\Delta\Psi_m^b$	4	mV	Potential at half saturation	(Cortassa et al., 2004)
j	0.1		Fraction of IMAC conductance	(Cortassa et al., 2004)
$\frac{RT}{F}$	26.730818	mV		

$$V_{MnSOD}=\frac{2\,k^{1}_{SOD}\;k^{5}_{SOD}\left(k^{1}_{SOD}+k^{3}_{SOD}\left(1+\frac{[H_2O_2]_m}{K_i^{H_2O_2}}\right)\right)E^{T}_{MnSOD}\,[O_2^{\bullet-}]_m}{k^{5}_{SOD}\left(2\,k^{1}_{SOD}+k^{3}_{SOD}\left(1+\frac{[H_2O_2]_m}{K_i^{H_2O_2}}\right)\right)+[O_2^{\bullet-}]_m\;k^{1}_{SOD}\;k^{3}_{SOD}\left(1+\frac{[H_2O_2]_m}{K_i^{H_2O_2}}\right)} \quad (S191)$$

$$V_{CuZnSOD}=\frac{2\,k^{1}_{SOD}\;k^{5}_{SOD}\left(k^{1}_{SOD}+k^{3}_{SOD}\left(1+\frac{[H_2O_2]_i}{K_i^{H_2O_2}}\right)\right)E^{T}_{CuZnSOD}\,[O_2^{\bullet-}]_i}{k^{5}_{SOD}\left(2\,k^{1}_{SOD}+k^{3}_{SOD}\left(1+\frac{[H_2O_2]_i}{K_i^{H_2O_2}}\right)\right)+[O_2^{\bullet-}]_i\;k^{1}_{SOD}\;k^{3}_{SOD}\left(1+\frac{[H_2O_2]_i}{K_i^{H_2O_2}}\right)} \quad (S192)$$

$$V_{diff_{H_2O_2}}=c_{diff_{H_2O_2}}\left([H_2O_2]_m-[H_2O_2]_i\right) \quad (S193)$$

$$V_{GPX_m}=\frac{E_T^{GPXm}[H_2O_2]_m\,[GSH]_m}{\Phi_1[GSH]_m+\Phi_2[H_2O_2]_m} \quad (S194)$$

$$V_{GPX_i}=\frac{E_T^{GPXi}[H_2O_2]_i\,[GSH]_i}{\Phi_1[GSH]_i+\Phi_2[H_2O_2]_i} \quad (S195)$$

$$V_{GR_m}=\frac{k^{1}_{GR}\;E_T^{GRm}}{1+\frac{K_M^{GSSG}}{[GSSG]}+\frac{K_M^{NADPH}}{[NADPH]_m}+\frac{K_M^{GSSG}}{[GSSG]_m}\frac{K_M^{NADPH}}{[NADPH]_m}} \quad (S196)$$

$$V_{GR_i}=\frac{k^{1}_{GR}\;E_T^{GRi}}{1+\frac{K_M^{GSSG}}{V_{GSS}}+\frac{K_M^{NADPH}}{[NADPH]_i}+\frac{K_M^{GSSG}}{[GSSG]_i}\frac{K_M^{NADPH}}{[NADPH]_i}} \quad (S197)$$

$$V_{GRX_m}=\frac{k_{grx_m}\;K_{eq}^{GRX}\left([GSH]_m\right)^2\;GrxT\;[PSSG]_m}{\left([GSSG]_m+K_{eq}^{GRX}\left([GSH]_m\right)^2\right)\left(\frac{K_{eq}^{GRX}\left([GSH]_m\right)^2\;GrxT}{[GSSG]_m+K_{eq}^{GRX}\left([GSH]_m\right)^2}+K_m^{Grx}\right)\left([PSSG]_m+K_m^{PSSG}\right)} \quad (S198)$$

$$V_{GRX_i}=\frac{k_{grx_i}\;K_{eq}^{GRX}\left([GSH]_i\right)^2\;GrxT\;[PSSG]_i}{\left(V_{GSS}+K_{eq}^{GRX}\left([GSH]_i\right)^2\right)\left(\frac{K_{eq}^{GRX}\left([GSH]_i\right)^2\;GrxT}{[GSSG]_i+K_{eq}^{GRX}\left([GSH]_i\right)^2}+K_m^{Grx}\right)\left([PSSG]_i+K_m^{PSSG}\right)} \quad (S199)$$

$$V_{PSSG_m} = \frac{k^1_{\mathrm{PSH}}\ \mathrm{E}^{\mathrm{PSH}}_T\ (PSSGT - [PSSG]_m)}{\left(1 + \frac{K^{GSH}_M}{[GSH]_m}\right)\left(1 + \frac{[H_2O_2]_m}{K^{H2O2}_{act}}\right)} \quad \text{(S200)}$$

$$V_{PSSG_i} = \frac{k^1_{\mathrm{PSH}}\ \mathrm{E}^{\mathrm{PSH}}_T\ (PSSGT - [PSSG]_i)}{\left(1 + \frac{K^{GSH}_M}{[GSH]_i}\right)\left(1 + \frac{[H_2O_2]_i}{K^{H2O2}_{act}}\right)} \quad \text{(S201)}$$

$$\mathrm{G}_T = \mathrm{G}_T - [\mathrm{GSH}]_{\mathrm{m}} - [\mathrm{GSH}]_i - 2[\mathrm{GSSG}] - [\mathrm{PSSG}]_{\mathrm{m}} - [\mathrm{PSSG}]_{\mathrm{i}} - 2[GSSG]_i \quad \text{(S202)}$$

$$[\mathrm{GSSG}]_i = 0.5\left(\mathrm{G}_T - [\mathrm{GSH}]_{\mathrm{m}} - [\mathrm{GSH}]_i - 2[\mathrm{GSSG}]_{\mathrm{m}} - [\mathrm{PSSG}]_{\mathrm{m}} - [\mathrm{PSSG}]_{\mathrm{i}}\right) \quad \text{(S203)}$$

$$V_{GST} = c_{GST}\frac{\left([GSH]_i - [GSH]_m\right)}{[GSH]_i + k^{GST}_{0.5}} \quad \text{(S204)}$$

$$V_{TxPX_m} = \frac{E^{Prx3m}_T [H_2O_2]_m\ [TrxSH_2]_m}{\Phi_{1Prx}[TrxSH_2]_m + \Phi_{2Prx}[H_2O_2]_m} \quad \text{(S205)}$$

$$V_{TxPX_i} = \frac{E^{Prxi}_T [\mathrm{H_2O_2}]_{\mathrm{i}} [\mathrm{TrxSH_2}]_{\mathrm{i}}}{\Phi_{1Prx}[\mathrm{TrxSH_2}]_{\mathrm{i}} + \Phi_{2Prx}[\mathrm{H_2O_2}]_i} \quad \text{(S206)}$$

$$V_{TxR_m} = \frac{k^1_{\mathrm{Trx}R}\ \mathrm{E}^{Tr\mathrm{xR2m}}_T}{1 + \frac{K^{\mathrm{TrxSS}}_M}{[TrxSS]_m} + \frac{K^{\mathrm{NADPH}}_{Mtrx}}{[NADPH]_m} + \frac{K^{\mathrm{TrxSS}}_M}{[TrxSS]_m}\frac{K^{\mathrm{NADPH}}_{Mtrx}}{[NADPH]_m}} \quad \text{(S207)}$$

$$V_{TxR_i} = \frac{k^1_{\mathrm{TrxR}}\ \mathrm{E}^{\mathrm{TrxRi}}_T}{1 + \frac{K^{\mathrm{TrxSS}}_M}{[TrxSS]_i} + \frac{K^{\mathrm{NADPH}}_{Mtrx}}{[NADPH]_i} + \frac{K^{\mathrm{TrxSS}}_M}{[TrxSS]_i}\frac{K^{\mathrm{NADPH}}_{Mtrx}}{[NADPH]_i}} \quad \text{(S208)}$$

$$[\mathrm{TrxSS}]_{\mathrm{m}} = TrxT_m - [\mathrm{TrxSH_2}]_{\mathrm{m}} \quad \text{(S209)}$$

$$[\mathrm{TrxSS}]_{\mathrm{i}} = TrxT_i - [\mathrm{TrxSH_2}]_{\mathrm{i}} \quad \text{(S210)}$$

$$V_{CAT} = 2k^1_{CAT}E^T_{CAT}[\mathrm{H_2O_2}]_{\mathrm{i}}\,e^{-fr[\mathrm{H_2O_2}]_{\mathrm{i}}} \quad \text{(S211)}$$

1.2.24 Supplementary Table S15. Parameters corresponding to ROS production and scavenging

Symbol	Value	Units	Description
k^1_{SOD}	1.2×10^3	$mM^{-1}ms^{-1}$	SOD second-order rate constant
k^3_{SOD}	24	$mM^{-1}ms^{-1}$	SOD second-order rate constant
k^5_{SOD}	2.4×10^{-4}	ms^{-1}	SOD first-order rate constant
$K_i^{H_2O_2}$	0.5	mM	Inhibition constant for H_2O_2
E^T_{MnSOD}	3.0×10^{-3}	mM	MnSOD matrix concentration
$E^T_{CuZnSOD}$	3.0×10^{-3}	mM	Cu,ZnSOD concentration
$c_{diff_{H_2O_2}}$	2×10^{-4}	ms^{-1}	Diffusion constant for H_2O_2
Φ_1	5.0×10^{-3}	mM ms	GPX activity constant
Φ_2	0.75	mM ms	GPX activity constant
E_T^{GPXm}	1.0×10^{-4}	mM	GPX matrix concentration
E_T^{GPXi}	5.0×10^{-5}	mM	GPX extra-matrix concentration
k^1_{GR}	2.5×10^{-3}	ms^{-1}	Catalytic constant of glutathione reductase (GR)
E_T^{GRm}	9.0×10^{-4}	mM	GR matrix concentration
E_T^{GRi}	9.0×10^{-4}	mM	GR extra-matrix concentration
K_M^{NADPH}	0.015	mM	GR Michaelis constant for NADPH
K_M^{GSSG}	0.06	mM	GR Michaelis constant for GSSG
$[NADPH]_i$	7.5×10^{-2}	mM	Extra-matrix NADPH concentration
G_T	6	mM	Total pool of glutathione
k_{grx_m}	3.6×10^{-4}	$mM\ s^{-1}$	Rate constant of mitochondrial matrix glutaredoxin (GRX) reaction
k_{grx_i}	3.6×10^{-4}	$mM\ s^{-1}$	GRX extra-matrix rate constant
K_{eq}^{GRX}	1.37×10^{-3}	mM^{-1}	GRX equilibrium constant
K_m^{Grx}	0.01	mM	GRX Michaelis constant for GSH

Symbol	Value	Units	Description
K_m^{PSSG}	0.0005	mM	Michaelis constant for glutathionylated proteins
k_{PSH}^1	0.64	ms^{-1}	Rate constant of protein glutathionylation
$\mathrm{E}_T^{\mathrm{PSH}}$	8×10^{-4}	mM	Concentration of proteins that can become glutathionylated
K_M^{GSH}	0.75	mM	Michaelis constant of GSH for glutathionylation
K_{act}^{H2O2}	1×10^{-3}	mM	Activation constant of H_2O_2 for protein glutathionylation
$GrxT$	0.002	mM	Glutaredoxin concentration
c_{GST}	1.5×10^{-8}	ms^{-1}	Rate constant of glutathione transporter
$k_{0.5}^{GST}$	2.6	mM	Transport association constant of GSH
E_T^{Prx3m}	3.0×10^{-3}	mM	Mitochondrial matrix concentration of Trx peroxidase (Prx)
E_T^{Prx3i}	0.1	mM	Prx extra-matrix concentration
Φ_{1Prx}	3.83	mM ms	Constant for TxPX activity
Φ_{2Prx}	1.85	mM ms	Constant for TxPX activity
$\mathrm{E}_T^{Trx\mathrm{R2m}}$	3.5×10^{-4}	mM	Mitochondrial matrix concentration of thioredoxin reductase2 (TrxR2)
$\mathrm{E}_T^{Trx\mathrm{R}i}$	3.5×10^{-4}	mM	TrxR extra-matrix concentration
K_M^{TrxSS}	0.035	mM	TrxR Michaelis constant for oxidized Trx [Trx(SS)]
$\mathrm{K}_{Mtrx}^{NADPH}$	0.012	mM	Trx Michaelis constant for NADPH
k_{TrxR}^1	22.7×10^{-3}	ms^{-1}	TrxR rate constant
$TrxT_m$	0.025	mM	Total pool of mitochondrial matrix thioredoxin
$TrxT_i$	0.05	mM	Total pool of extra-matrix thioredoxin
k_{CAT}^1	17	$mM^{-1}ms^{-1}$	Rate constant of catalase (CAT)
E_{CAT}^T	1.0×10^{-6}	mM	CAT extra-matrix concentration
fr	5.0×10^{-2}	mM^{-1}	CAT hydrogen peroxide inhibition factor

All parameters were taken from (Kembro et al., 2013b).

1.2.25 Mitochondrial NADPH reduction

$$NADPm = C_{NADPm} - \left[NADPH\right]_m \tag{S212}$$

$$V_{IDP\_NADP} = \left(1 + \frac{\left[H^+\right]_m}{k_{m\_IDP}^{H+}}\right)\left(\begin{array}{l} 1 + \frac{\left[ISOC\right]}{k_{m\_IDP}^{ISOC}} + \frac{NADP_m}{k_{m\_IDP}^{NADP}}\left(1 + \frac{k_{i\_IDP}^{NADP}}{NADP_m}\right) + \frac{\left[aKG\right]}{k_{m\_IDP}^{aKG}} + \frac{\left[NADPH_m\right]}{k_{m\_IDP}^{NADPH}} + \ldots \\ \ldots \frac{\left[ISOC\right]}{k_{m\_IDP}^{ISOC}}\frac{NADP_m}{k_{m\_IDP}^{NADP}}\left(1 + \frac{k_{i\_IDP}^{NADP}}{NADP_m}\right) + \frac{\left[aKG\right]}{k_{m\_IDP}^{aKG}}\frac{\left[NADPH_m\right]}{k_{m\_IDP}^{NADPH}} + \ldots \\ \ldots \frac{\left[ISOC\right]}{k_{m\_IDP}^{ISOC}}\frac{\left[NADPH_m\right]}{k_{m\_IDP}^{NADPH}} + \frac{\left[aKG\right]}{k_{m\_IDP}^{aKG}}\frac{NADP_m}{k_{m\_IDP}^{NADP}}\left(1 + \frac{k_{i\_IDP}^{NADP}}{NADP_m}\right) \end{array}\right) \tag{S213}$$

$$V_{IDH_{NADP}} = \frac{V_f^{IDH}\frac{k\left[ISOC\right]}{k_{m\_IDP}^{ISOC}}\frac{NADP_m}{k_{m\_IDP}^{NADP}}\left(1 + \frac{k_{i\_IDP}^{NADP}}{VNADH_m}\right) - V_b^{IDH}\frac{\left[aKG\right]}{k_{m\_IDP}^{aKG}}\frac{\left[NADP_m\right]}{k_{m\_IDP}^{NADPH}}}{V_{IDP\_NADP}} \tag{S214}$$

$$\begin{aligned} V_{THDen} = {} & 1 + \frac{\left[NADH_m\right]}{k_{m\_THD}^{NADHm}} + \frac{NAD}{k_{m\_THD}^{NAD}} + \frac{NADP_m}{k_{m\_THD}^{NADP}} + \frac{\left[NADPH_m\right]}{k_{m\_THD}^{NADPH}} + \frac{\left[NADH_m\right]}{k_{m\_THD}^{NADHm}}\frac{NADP_m}{k_{m\_THD}^{NADP}}e^{(F/10RT).\Delta\mu_H} + \\ & \frac{\left[NADPH_m\right]}{k_{m\_THD}^{NADPH}}\frac{\left[NADH_m\right]}{k_{m\_THD}^{NADHm}}e^{(1-(F/10RT).\Delta\mu_H)} + \frac{NAD}{k_{m\_THD}^{NAD}}\frac{NADP_m}{k_{m\_THD}^{NADP}}e^{(F/10RT).\Delta\mu_H}.e^{(1-(F/10RT).\Delta\mu_H)} + \\ & \frac{\left[NADH_m\right]}{k_{m\_THD}^{NADHm}}\frac{\left[NADPH_m\right]}{k_{m\_THD}^{NADPH}} \end{aligned} \tag{S215}$$

$$V_{THD} = \frac{E_T^{THD}.k_a^{THD}\frac{\left[NADH\right]_m}{k_{m\_THD}^{NADHm}}\frac{NADP_m}{k_{m\_THD}^{NADP}}e^{(F/10RT).\Delta\mu_H} - E_T^{THD}.k_b^{THD}\frac{NAD}{k_{m\_THD}^{NAD}}\frac{\left[NADPH_m\right]}{k_{m\_THD}^{NADPH}}e^{(1-(F/10RT).\Delta\mu_H)}}{V_{THDen}} \tag{S216}$$

1.2.26 Supplementary Table S16. Parameters used in mitochondrial NADPH handling

Symbol	Value	Units	Description
C_{NADPm}	0.1	mM	Sum of NADPH plus $NADP^+$
$k_{m\_IDP}^{H+}$	0.5	mM	Dissociation constant for H^+ of isocitrate dehydrogenase2 (IDH2)
$k_{m\_IDP}^{ISOC}$	3.9×10^{-3}	mM	IDH2 Michaelis constant for ISOC
$k_{m\_IDP}^{NADP}$	6.7×10^{-3}	mM	IDH2 Michaelis constant for NADP
$k_{i\_IDP}^{NADP}$	2×10^{-6}	mM	IDH2 inhibition constant for NADP
$k_{m\_IDP}^{NADPH}$	1.2×10^{-2}	mM	IDH2 Michaelis constant for NADPH

Symbol	Value	Units	Description
$k_{m\_IDP}^{aKG}$	0.51	mM	IDH2 Michaelis constant for αKG
V_{f}^{IDH}	8.7×10-5	mM ms^{-1}	Maximal rate of IDH2 in the forward direction
V_{fb}^{IDH}	5.45×10^{-6}	mM ms^{-1}	Maximal rate of IDH2 in the reverse direction
$k_{m\_THD}^{NADPH}$	0.02	mM	Michaelis constant for NADPH in transhydrogenase (THD)
$k_{m\_THD}^{NADHm}$	0.01	mM	THD Michaelis constant for NADH
$k_{m\_THD}^{NAD}$	0.125	mM	THD Michaelis constant for NAD
$k_{m\_THD}^{NADP}$	0.017	mM	THD Michaelis constant for NADP
E_{T}^{THD}	1.187×10^{-5}	mM	THD enzyme concentration
k_{a}^{THD}	1.17474	ms^{-1}	THD forward catalytic constant
k_{b}^{THD}	10	ms^{-1}	THD reverse catalytic constant

All parameters were taken from (Kembro et al., 2013b)

2 Control analysis of the catabolic network model

2.1 Theoretical approach

Metabolic control analysis (MCA) is a quantitative methodology devoted to answer an important question that arises when one deals with networks of biochemical reactions of arbitrary complexity: what steps control the flux through a metabolic network? how is the concentration of the intermediaries controlled? It has been mainly applied to systems functioning at steady state (Fell, 1996).

The control coefficients describe how a variable or property of the system, typically a metabolic flux or the concentration of a metabolite, will respond to variation of a parameter, typically enzyme concentration or its kinetic properties, e.g. k_{cat}.

In a steady state, the flux control coefficient C_{Ek}^{Ji} is the fractional change in flux for a fractional change in the activity of enzyme, E_k (Kacser and Burns, 1973; Heinrich and Rapoport, 1974):

$$C_{Ek}^{Ji} = \frac{E_k}{J_i}\frac{\partial J_i}{\partial E_k} \tag{S217}$$

Similarly, a metabolite, Mi, concentration control coefficient, C_{Ek}^{Mi}, can be defined:

$$C_{Ek}^{Mi} = \frac{E_k}{M_i} \frac{\partial M_i}{\partial E_k} \quad \text{(S218)}$$

The link between the properties of an enzyme and its potential for flux control is given by the elasticity coefficient, ε. The elasticity coefficient εij for the effect of intermediate metabolite S on the velocity v_i of enzyme Ei is the fractional change in rate of the isolated enzyme for a fractional change, δS, in the amount, [*S*] of substrate S:

$$\varepsilon_S^{vi} = \frac{S}{v_i} \frac{\partial v_i}{\partial S} \quad \text{(S219)}$$

Summation and connectivity theorems. Several theorems constitute the main body of MCA. The summation theorem states that the sum of all flux control coefficients with respect to the activities of each of the enzymatic steps involved in the metabolic pathway being considered, is equal to unity (Kacser and Burns, 1973).

$$\sum_k C_{Ek}^{Ji} = 1 \quad \text{(S220)}$$

This theorem is linked to the concept that the enzymes of the pathway can share the control of the flux. In fact, an important contribution of MCA is to demonstrate that the control of a metabolic pathway can be shared by multiple enzymes, implying that the traditional concept of a single rate-limiting enzyme (a "bottleneck") is inaccurate. This result also illustrates the fact that many rounds of mutation are needed to increase substantially the flux through a pathway (Kacser and Burns, 1981). Moreover, the summation theorem of MCA highlights the fact that the flux control coefficient of an enzyme is not an intrinsic property of that enzyme, but a system property (Fell, 1996). Otherwise, increasing the activity of a rate-controlling enzyme also changes its flux control coefficient and so must be the coefficients of enzymes whose activities have not been changed. The latter accounts for the fact that the summation total for the flux control coefficients remains at 1 at all levels of the enzyme whose activity is being increased.

The connectivity theorems relate the elasticities to the control coefficients. For the flux control coefficients, the theorem reads:

$$\sum_i C_{vi}^{i} \varepsilon_k^{i} = 0 \quad \text{(S221)}$$

This theorem states that the sum over all products of the flux control coefficients (eqn. S217), with respect to the activity of the enzyme catalyzing step i, times the elasticity coefficient of this same enzyme (eqn. S219), is equal to zero. The connectivity theorem is regarded as the most meaningful of the MCA theorems, for it provides the route to understanding how the kinetics of the enzymes (represented by the elasticities) affect the values of the flux control coefficients (Fell, 1992).

A corresponding set of theorems exist for the concentration control coefficients. The summation theorem states that the sum of all metabolite control coefficients with respect to the activities of each of the enzymatic steps is equal to zero (Kacser and Burns, 1973).

$$\sum_{i} C^{M}_{Xi} = 0 \tag{S222}$$

Where M represents any one of the variable metabolites of the pathway, and X_i stands for the activity of enzyme *i*.

The connectivity theorem becomes slightly more complex, in that it has one form when the metabolite, the concentration of which is the subject of the control coefficient (A), is different from the one on the elasticities (B) (Fell, 1992):

$$\sum_{i=1}^{n} C^{A}_{Xi} \varepsilon^{i}_{B} = 0 \quad , \tag{S223}$$

but the following form when they are the same:

$$\sum_{i=1}^{n} C^{A}_{Xi} \varepsilon^{i}_{A} = -1 \tag{S224}$$

2.1.1 Implementation of control analysis of our catabolic network model

Control analysis was performed by applying the method developed by Reder (Reder, 1988) as described in (Cortassa et al., 2009a; Cortassa et al., 2012). In the framework of MCA, Reder developed a generalized linear algebra method that provides a way of analyzing the sensitivity of metabolic systems to perturbations triggered by either a change in the internal state of the system or by the environment. Here, we applied it to a system in which metabolism, ion transport, and mechanical function are coupled. Because the model was constructed in a modular way, the method can be applied with a higher or lower degree of detail or refinement. In this way, and depending on the question to be examined, the extent of control exerted by a module can be applied by zooming in and out.

The departure point of the analysis is the *stoichiometric matrix*, obtained from the set of differential equations of the model. The stoichiometric matrix defines the structural relationships between the processes, and the intermediates participating in the metabolic network under consideration. The information contained in the stoichiometric matrix is independent of both the enzyme kinetics and the parameters that rule the dynamic behavior of the metabolic system.

The second piece of information required to perform control analysis is the *elasticity matrix* defined by the dependence of each process in the metabolic network on the intermediates (e.g., ions or metabolites) included in the model. The elasticity matrix is quantified through the derivatives of the rates of individual processes with respect to each possible effector. Each elasticity coefficient reflects the local property of a process, for example, an enzyme activity with respect to its substrate, which is

linked to the global behavior of the system through the steady state levels of metabolites (substrates or effectors) that intervene in the network dynamics.

By applying matrix algebra, the matrices corresponding to control and response coefficients are obtained. Both kinds of matrix quantify the relationships of control and regulation, respectively, in the network of reactions. The regulation exerted by internal or external effectors to a network can be quantified by the response coefficient (Ainscow and Brand, 1999).

The following matrices' relationships were used in the computation of the flux and metabolite concentration control coefficients:

$$C = Id_r - D_x v\, L \left(N_r\, D_x v\, L\right)^{-1} N_r \qquad \text{(S225)}$$

$$\Gamma = -L \left(N_r\, D_x v\, L\right)^{-1} N_r \qquad \text{(S226)}$$

with C and Γ referring to the flux- and metabolite concentration control coefficients, respectively; Id_r, the identity matrix of dimension *r*, the number of processes in the network under study; $D_x v$ the elasticity matrix; N_r the reduced stoichiometric matrix and L, the link matrix that relates the reduced- to the full stoichiometric matrix of the system. For a more detailed description see (Cortassa et al., 2009a).

The advantages of the matrix method (Reder, 1988) over similar tools developed by other authors (Sauro et al., 1987; Westerhoff and Kell, 1987), is that *(i)* it can be applied to networks of any complexity including conserved cycles or multiple branches, and hierarchical relationships (Kahn and Westerhoff, 1991), and *(ii)* it is not based on the compliance of the theorems of metabolic control analysis and the particular conditions under which the analysis is carried out.

2.1.2 Supplementary Table S17 Comparison of PFK flux control coefficients (FCC) on representative fluxes throughout the catabolic network

Reaction	Finite Difference PFK FCC[a]	Matrix PFK FCC
GLUT4	0.107	0.1054
HK	0.107	0.1054
PFK	0.1151	0.1134
ALD	0.1149	0.1135
GAPD	0.1148	0.1131
PGK	0.1148	0.1131
ENOL	0.1148	0.1131
PK	0.1148	0.1131
LDH	0.1148	0.1134
G6PDH	-0.6111	-0.5988
P6GDH	-0.6111	-0.5988

Reaction	Finite Difference PFK FCC[a]	Matrix PFK FCC
R5PI	-0.4096	-0.3996
Ru5PE	-0.6761	-0.6631
RTAc	-0.4096	-0.3996
RTAc2	-0.4096	-0.3996
RTAl	-0.4096	-0.3996
ALDR1	-1.8862	-1.8587
SoDH	-1.8862	-1.8587
XyDH	-1.1626	-1.1442
FRUT	-1.8862	-1.8587
XyOHT	-1.1626	-1.1442
HydroATP	0.1144	0.1131
NDPcs	0.3878	0.2893
NTPcs	-0.6102	-0.598
GNO_is	0.3741	0.3697
GPa	0.1495	0.1477
GPb	9.5359	9.4245
PGLM	0.3741	0.3697
PyrC	0.1137	0.1437
PyrDH	0.1965	0.0675
CI_Resp	0.0676	0.041
ETFH	-0.1191	-0.1418
leak	0.0044	4.67E-04
MnSOD	0.0378	0.0236
GPXm	0.0377	0.0236
GRm	0.1783	0.0697
GPX	0.0318	0.0192
TxPXm	0.0378	0.0236
TxPX	0.0318	0.0192
TxRm	0.0378	0.0236
TxR	0.0318	0.0192
H2O2em	0.0378	0.0236
Cat	0.0318	0.0192
Cpt1C16	-0.1191	-0.0538
CactC16	-0.1191	-0.0538
Cpt2C16	-0.1191	-0.0538
lcadC16	-0.121	-0.0637

Reaction	Finite Difference PFK FCC[a]	Matrix PFK FCC
lcadC14	-0.1249	-0.0839
lcadC12	-0.1606	-0.1306
lcadC10	-0.1611	-0.1311
lcadC8	-0.1612	-0.1314
crotC6	-0.1191	-0.0538
crotC4	-0.1191	-0.0538

[a] Flux control coefficients were calculated from the finite differences in fluxes and activities after manually changing the Vmax of the PFK ±2.5 and ±5% of its original value (Table S1) and compared with the value obtained by the matrix method applied throughout this work.

References

Ainscow, E.K., and Brand, M.D. (1999). Internal regulation of ATP turnover, glycolysis and oxidative phosphorylation in rat hepatocytes. *Eur J Biochem* 266(3)**,** 737-749.

Bisaccia, F., De Palma, A., Prezioso, G., and Palmieri, F. (1990). Kinetic characterization of the reconstituted tricarboxylate carrier from rat liver mitochondria. *Biochim Biophys Acta* 1019(3)**,** 250-256.

Cortassa, S., Aon, M.A., Iglesias, A.A., Aon, J.C., and Lloyd, D. (2012). *An Introduction to Metabolic and Cellular Engineering.* Singapore: World Scientific Publishers.

Cortassa, S., Aon, M.A., Marban, E., Winslow, R.L., and O'Rourke, B. (2003). An integrated model of cardiac mitochondrial energy metabolism and calcium dynamics. *Biophys J* 84(4)**,** 2734-2755. doi: 10.1016/S0006-3495(03)75079-6.

Cortassa, S., Aon, M.A., O'Rourke, B., Jacques, R., Tseng, H.J., Marban, E., et al. (2006). A computational model integrating electrophysiology, contraction, and mitochondrial bioenergetics in the ventricular myocyte. *Biophys J* 91(4)**,** 1564-1589. doi: 10.1529/biophysj.105.076174.

Cortassa, S., Aon, M.A., Winslow, R.L., and O'Rourke, B. (2004). A mitochondrial oscillator dependent on reactive oxygen species. *Biophys J* 87(3)**,** 2060-2073. doi: 10.1529/biophysj.104.041749.

Cortassa, S., Caceres, V., Bell, L.N., O'Rourke, B., Paolocci, N., and Aon, M.A. (2015). From metabolomics to fluxomics: a computational procedure to translate metabolite profiles into metabolic fluxes. *Biophys J* 108(1)**,** 163-172. doi: S0006-3495(14)03069-0 [pii] 10.1016/j.bpj.2014.11.1857.

Cortassa, S., O'Rourke, B., Winslow, R.L., and Aon, M.A. (2009a). Control and regulation of mitochondrial energetics in an integrated model of cardiomyocyte function. *Biophys J* 96(6)**,** 2466-2478.

Cortassa, S., Sollott, S.J., and Aon, M.A. (2017a). Mitochondrial respiration and ROS emission during beta-oxidation in the heart: An experimental-computational study. *PLoS Comput Biol* 13(6)**,** e1005588. doi: 10.1371/journal.pcbi.1005588.

Cortassa, S., Sollott, S.J., and Aon, M.A. (2017b). "Substrate selection and its impact on mitochondrial respiration and redox," in *Molecular Basis for Mitochondrial Signaling,* ed. T.K. Rostovtseva. Springer International Publishing AG).

Fell, D.A. (1992). Metabolic control analysis: a survey of its theoretical and experimental development. *Biochem J* 286 (Pt 2)**,** 313-330.

Fell, D.A. (1996). *Understanding the Control of Metabolism.* London: Portland Press.

Halestrap, A.P. (1978). Pyruvate and ketone-body transport across the mitochondrial membrane. Exchange properties, pH-dependence and mechanism of the carrier. *Biochem J* 172(3)**,** 377-387.

Heinrich, R., and Rapoport, T.A. (1974). A linear steady-state treatment of enzymatic chains. General properties, control and effector strength. *Eur J Biochem* 42(1)**,** 89-95.

Kacser, H., and Burns, J.A. (1973). The control of flux. *Symp Soc Exp Biol* 27**,** 65-104.

Kacser, H., and Burns, J.A. (1981). The molecular basis of dominance. *Genetics* 97(3-4)**,** 639-666.

Kahn, D., and Westerhoff, H.V. (1991). Control theory of regulatory cascades. *J Theor Biol* 153(2)**,** 255-285.

Karacaoglan, V., and Ozer, I. (2005). Steady-state kinetic properties of sorbitol dehydrogenase from chicken liver. *Comp Biochem Physiol B Biochem Mol Biol* 140(2)**,** 309-312. doi: S1096-4959(04)00358-6 [pii] 10.1016/j.cbpc.2004.10.017.

Kembro, J.M., Aon, M.A., Winslow, R.L., O'Rourke, B., and Cortassa, S. (2013a). Integrating mitochondrial energetics, redox and ROS metabolic networks: A two-compartment model. *Biophysical Journal* 104**,** 332-343.

Kembro, J.M., Aon, M.A., Winslow, R.L., O'Rourke, B., and Cortassa, S. (2013b). Integrating mitochondrial energetics, redox and ROS metabolic networks: a two-compartment model. *Biophys J* 104(2)**,** 332-343. doi: S0006-3495(12)05055-2 [pii] 10.1016/j.bpj.2012.11.3808.

Kubiseski, T.J., Hyndman, D.J., Morjana, N.A., and Flynn, T.G. (1992). Studies on pig muscle aldose reductase. Kinetic mechanism and evidence for a slow conformational change upon coenzyme binding. *J Biol Chem* 267(10)**,** 6510-6517.

Lambeth, M.J., and Kushmerick, M.J. (2002). A computational model for glycogenolysis in skeletal muscle. *Ann Biomed Eng* 30(6)**,** 808-827.

Lueck, J.D., and Fromm, H.J. (1974). Kinetics, mechanism, and regulation of rat skeletal muscle hexokinase. *J Biol Chem* 249(5)**,** 1341-1347.

Mazumder, P.K., O'Neill, B.T., Roberts, M.W., Buchanan, J., Yun, U.J., Cooksey, R.C., et al. (2004). Impaired cardiac efficiency and increased fatty acid oxidation in insulin-resistant ob/ob mouse hearts. *Diabetes* 53(9)**,** 2366-2374. doi: 53/9/2366 [pii].

McIntyre, L.M., Thorburn, D.R., Bubb, W.A., and Kuchel, P.W. (1989). Comparison of computer simulations of the F-type and L-type non-oxidative hexose monophosphate shunts with 31P-NMR experimental data from human erythrocytes. *Eur J Biochem* 180(2)**,** 399-420.

Nishino, T., Yachie-Kinoshita, A., Hirayama, A., Soga, T., Suematsu, M., and Tomita, M. (2009). In silico modeling and metabolome analysis of long-stored erythrocytes to improve blood storage methods. *J Biotechnol* 144(3)**,** 212-223. doi: S0168-1656(09)00355-1 [pii] 10.1016/j.jbiotec.2009.08.010.

O'Brien, M.M., Schofield, P.J., and Edwards, M.R. (1983). Polyol-pathway enzymes of human brain. Partial purification and properties of sorbitol dehydrogenase. *Biochem J* 211(1)**,** 81-90.

Pettit, F.H., Pelley, J.W., and Reed, L.J. (1975). Regulation of pyruvate dehydrogenase kinase and phosphatase by acetyl-CoA/CoA and NADH/NAD ratios. *Biochem Biophys Res Commun* 65(2)**,** 575-582. doi: S0006-291X(75)80185-9 [pii].

Randle, P.J., and Tubbs, P.K. (1979). "Carbohydrate and fatty acid metabolism", in: *Handbook of Physiology.* (ed.) N.S. R.M. Berne, R. Geiger. American Physiology Society).

Reder, C. (1988). Metabolic control theory: a structural approach. *J Theor Biol* 135(2)**,** 175-201.

Rowan, A.N., Newsholme, E.A., and Scrutton, M.C. (1978). Partial purification and some properties of pyruvate carboxylase from the flight muscle of the locust (Schistocerca gregaria). *Biochim Biophys Acta* 522(1)**,** 270-275.

Sauro, H.M., Small, J.R., and Fell, D.A. (1987). Metabolic control and its analysis. Extensions to the theory and matrix method. *Eur J Biochem* 165(1)**,** 215-221.

Schafer, F.Q., and Buettner, G.R. (2001). Redox environment of the cell as viewed through the redox state of the glutathione disulfide/glutathione couple. *Free Radic Biol Med* 30(11)**,** 1191-1212.

van Eunen, K., Simons, S.M., Gerding, A., Bleeker, A., den Besten, G., Touw, C.M., et al. (2013). Biochemical competition makes fatty-acid beta-oxidation vulnerable to substrate overload. *PLoS Comput Biol* 9(8)**,** e1003186. doi: 10.1371/journal.pcbi.1003186 PCOMPBIOL-D-13-00495 [pii].

Wei, A.C., Aon, M.A., O'Rourke, B., Winslow, R.L., and Cortassa, S. (2011). Mitochondrial energetics, pH regulation, and ion dynamics: a computational-experimental approach. *Biophys J* 100(12)**,** 2894-2903. doi: S0006-3495(11)00597-2 [pii] 10.1016/j.bpj.2011.05.027.

Westerhoff, H.V., and Kell, D.B. (1987). Matrix method for determining steps most rate-limiting to metabolic fluxes in biotechnological processes. *Biotechnol Bioeng* 30(1)**,** 101-107. doi: 10.1002/bit.260300115.

Matlab file for main function "WholeCatab_v3.m"

```
function dydt = WholeCatab_v3 (t,kmrgd) %
%% State variables
Cam=kmrgd(1);
ADPm=kmrgd(2);
Dpsim=kmrgd(3);
NADHm=kmrgd(4);
H_mito=kmrgd(5);
Pim=kmrgd(6);
ISOm=kmrgd(7);
aKGm=kmrgd(8);
SCoA=kmrgd(9);
Succ=kmrgd(10);
FUM=kmrgd(11);
MALm=kmrgd(12);
Oaa=kmrgd(13);
C16Carn_cy=kmrgd(14);
C16Carn_m=kmrgd(15);
C16CoA_m=kmrgd(16);
C16EnoylCoA_m=kmrgd(17);
C16OHCoA_m=kmrgd(18);
C16KetoCoA_m=kmrgd(19);
C14CoA_m=kmrgd(20);
C14EnoylCoA_m=kmrgd(21);
C14OHCoA_m=kmrgd(22);
C14KetoCoA_m=kmrgd(23);
C12CoA_m=kmrgd(24);
C12EnoylCoA_m=kmrgd(25);
C12OHCoA_m=kmrgd(26);
C12KetoCoA_m=kmrgd(27);
C10CoA_m=kmrgd(28);
C10EnoylCoA_m=kmrgd(29);
C10OHCoA_m=kmrgd(30);
C10KetoCoA_m=kmrgd(31);
C8CoA_m=kmrgd(32);
C8EnoylCoA_m=kmrgd(33);
C8OHCoA_m=kmrgd(34);
C8KetoCoA_m=kmrgd(35);
C6CoA_m=kmrgd(36);
C6EnoylCoA_m=kmrgd(37);
C6OHCoA_m=kmrgd(38);
C6KetoCoA_m=kmrgd(39);
C4CoA_m=kmrgd(40);
C4EnoylCoA_m=kmrgd(41);
C4OHCoA_m=kmrgd(42);
C4KetoCoA_m=kmrgd(43);
```

```
AcCoA_m=kmrgd(44);
FADH_m=kmrgd(45);
NADPHm=kmrgd(46);
SO2m=kmrgd(47);
SO2i=kmrgd(48);
H2O2m=kmrgd(49);
H2O2i=kmrgd(50);
GSHm=kmrgd(51);
GSHi=kmrgd(52);
GSSGm=kmrgd(53);
TrxSH2m=kmrgd(54);
TrxSH2=kmrgd(55);
GSSGi=kmrgd(56);
PSSGm=kmrgd(57);
PSSGi=kmrgd(58);
Glci=kmrgd(59);
G6P=kmrgd(60);
FbP=kmrgd(61);
G3P=kmrgd(62);
G13bP=kmrgd(63);
GP3=kmrgd(64);
PEP=kmrgd(65);
Pyr=kmrgd(66);
Sor=kmrgd(67);
FRU=kmrgd(68);
GP6=kmrgd(69);
Ru5P=kmrgd(70);
R5P=kmrgd(71);
X5P=kmrgd(72);
S7P=kmrgd(73);
E4P=kmrgd(74);
XyOH=kmrgd(75);
ATPc=kmrgd(76);
NADHc=kmrgd(77);
NADPHc=kmrgd(78);
Maltoside=kmrgd(79);
G1P=kmrgd(80);
Pyrm=kmrgd(81);
CITm=kmrgd(82);

%% Parameters
GLUT4_kcat=2.0*2.65E-5;
NDP=0.75; % total level of pyrimidine nucleotides in cytoplasm (NAD + NADH)
NTP=0.05; % total of pyrimidine phosphate dinucleotides (NADP+ NADPH)
CadTcy=8.9; % total of adenine nucleotides (ATP + ADP)
Glco=9.0;
AMP_c=0.01; %0.1
```

```
GDP=0.1;
Pi_cy=3.0;
% pH=7.4;
HKVmax=2.0*0.0052;
HKKmGLC=1.1E-1;
HKKmAT=0.17;
HKKmG6P=0.0014;
HKKmAD=1.2;
HKKapp=7800;
PFKVmax=2.0*3.5E-4;
PFKKmAT=0.08;
PFKKmpAT=0.25;
PFKKmF6P=0.18;
PFKKmpF6P=20;
PFKKmAD=2.7;
PFKKmpAD=2.7;
PFKKmFBP=4.02;
PFKKmpFBP=4.02;
PFKKeq=242;
PFKLo=13;
PFKKiATP=0.87;
PFKKaAMP=0.015;
PFKKiCit=0.03;
PFK_d=0.01;
PFK_e=0.01;
PFK_f=0.01;
ALDVmax=2.0*1.05E-3; %mM ms-1
ALDKmFBP=0.05;
ALDKmG3P=2.1;
ALDKeq=1.2E-1; %(mM-1)
GAPDVmax=2.0*2.0E-3; %mM s-1
GAPDKmG13BP=0.0008;
GAPDKmG3P=0.0025;
GAPDKmNADH=0.00033;
GAPDKmNAD=0.009;
nGDH=0.64;
GAPDKmPi=0.29;
GAPDKeq=0.089; % adimensional
PGKVmax=2.0*1.25E-3;
PGKKmGP3=1.2;
PGKKmG13BP=0.0022;
PGKKmATP=0.35;
PGKKmADP=0.0008;
PGKKeq=57109; %adimensional
ENOLVmax=2.0*1.1E-3;
ENOLKmPEP=0.37;
ENOLKmGP3=0.12;
```

```
ENOLKeq=0.49; %adimensional
PKVmax=2.0*0.208;
PKKmPEP=0.08;
PKKmAD=0.3;
PKKmPyr=7.05;
PKKmAT=1.13;
PKKmFBP=0.005;
PKKmGDP=0.1;
PKKeq=10304;
LAC=4.0E-2;
LDHVmax=2.0*2.7E-3;
LDHKmPyr=0.0335;
LDHKmNADH=0.002;
LDHKmLAC=17;
LDHKmNAD=0.849;
LDHKeq=16198; %adimensional
Vmaxamil=2.0*1.9E-5;
GLY=110;
Kmamil=5.0;
KmMaltoside=1.0;
VmaxGPa= 2.0*1.0E-3;
VmaxGPb= 2.0*6.24e-4;
KaiGlyf=2.0; % 2.0
KaGlyf=1.7; % 1.7
Kapi=4.0E-1;
KaGlyb=0.15;
Kaig1p=10.1;
KeqGP=0.42;
Kiapi=4.7;
KibGlyf=15;
Kbpi=0.2;
KibGlyb=4.4;
Kbig1p=7.4;
Kibpi=4.6;
Kbg1p=1.5;
KpAMP=1.9E-6;
nH=1.75;
VmaxPGLM=2.0*7.0e-5;
KG1P=0.063;
KG6P=0.03;
KeqPGLM=16.62;
G6PDHVmax=2.0*2.0E-6;
G6PDHKmNADP=0.00367;
G6PDHKmNADPH=0.00312;
G6PDHKmG6P=0.0667;
G6PDHKmATP=0.749;
P6GDHVmax=2.0*5.0E-6;
```

P6GDHk1=2.4E3;
P6GDHk3=2.0E6;
P6GDHk5=48;
P6GDHk7=6.3E2;
P6GDHk9=8.0E2;
P6GDHk2=4.1E2;
P6GDHk4=26;
P6GDHk6=30;
P6GDHk8=36;
P6GDHk10=2.25E2;
R5PIVmax=2.0*1.8E-6;
R5PIk1=6.09E1;
R5PIk2=33.3;
R5PIk3=14.2;
R5PIk4=2.16E1;
Ru5PEVmax=2.0*1.7E-5;
Ru5PEk1=3.91E3;
Ru5PEk2=4.38E2;
Ru5PEk3=3.05E2;
Ru5PEk4=1.49E3;
R5PIKmRu5P=(R5PIk2+R5PIk3)/R5PIk1;
R5PIKmR5P=(R5PIk2+R5PIk3)/R5PIk4;
Ru5PEKmRu5P=(Ru5PEk2+Ru5PEk3)/Ru5PEk1;
Ru5PEKmXu5P=(Ru5PEk2+Ru5PEk3)/Ru5PEk4;
RTAcVmax=2.0*1.35E-6;
RTAck1=2.16E2;
RTAck2=38.0;
RTAck3=34.0;
RTAck4=1.56E2;
RTAck5=3.29E2;
RTAck6=1.75E2;
RTAck7=40;
RTAck8=4.48E1;
RTAc2Vmax=2.0*2.5E-6;
RTAc2k1=2.16E2;
RTAc2k2=38; 3.8;
RTAc2k3=34;
RTAc2k4=1.56E2;
RTAc2k5=3.29E2;
RTAc2k6=1.75E2;
RTAc2k7=40.0;
RTAc2k8=4.48E1;
RTAlVmax=2.0*2.9E-5;
RTAlk1=2.16E2;
RTAlk2=4.53;
RTAlk3=16.3;
RTAlk4=3.0E1;

```
RTAlk5=4.9E2;
RTAlk6=60;
RTAlk7=17;
RTAlk8=7.9E1;
ALDREmax=2.0*1.7E-8;
ALR1V2=0.037;
ALR1V1=0.33;
ALR1KA=6.9E-4;
ALR1KB=4.6E1;
ALR1KP=3.8E2;
ALR1KQ=1.5E-2;
ALR1KIa=4.1E-4;
ALR1KIb=9.2E-2;
ALR1KIp=1.3E3;
ALR1KIq=8.3E-3;
ALR1Keq=ALR1V1/ALR1V2*ALR1KP*ALR1KIq/ALR1KIa/ALR1KB;
DALR1=ALR1KIa*ALR1KB*ALR1V2;
DALR2=ALR1KB*ALR1V2;
DALR3=ALR1KA*ALR1V2;
DALR4=ALR1KQ*ALR1V1/ALR1Keq;
DALR5=ALR1KP*ALR1V1/ALR1Keq;
DALR6=ALR1V2;
DALR7=ALR1KQ*ALR1V1/ALR1KIa/ALR1Keq;
DALR8=ALR1KA*ALR1V2/ALR1KIq;
DALR9=ALR1V1/ALR1Keq;
DALR10=ALR1V2/ALR1KIp;
DALR11=ALR1V1/ALR1KIb/ALR1Keq;
SoDHVmax=2.0*3.0E-7;
SoDHKmFRU=1.0E3;
SoDHKmNADH=1.0E-2;
SoDHKmSorb=9.6;
SoDHKmNAD=2.0;
SoDHKiNAD=2.0E0;
SoDHKiNADH=1.0E-2;
SoDHKeq=3.7E-6*2.5E4;
XyDHVmax=2.0*1.5E-4;
XyDHKmXy=2.0E1;
XyDHKmNADH=1.0E-2;
XyDHKmXyOH=8.08E-1;
XyDHKmNAD=2.0;
XyDHKiNADH=1.0E-2;
XyDHKiNAD=2.0;
XyDHKeq=0.24;
HydrolVmax=0.003;
HydroKmATP=9.0;
NADHDemVmax=2.0E-4;
NADHDemKmNADH=5.0E-2;
```

```
NADHDemKmNAD=0.7;
NADPHDemVmax=0.32;
NADPHDemKmNADPH=0.025;
NADPHDemKmNADP=0.0075;
ktfru=1.0e-6;
ktXyOH=5.0E-6;
kmXyOH=1.5E-5;
kmFru=3.0;
% Mitochondrial processes parameters
KeqAAT=6.6;
PyrCbVmax=0.5E-3;  % (mM s-1)
PyrCbKmPyr=0.23;  % (in mM)
PyrCbKmATP=0.25; % (in mM)
PyrCbK1=2.512E-5; % (mM)
PyrCbK2=1.259E-5; % (mM)
% Pyruvate dehydrogenase
PyrDHVmax=0.0075; % mM ms-1
PyrDHKmCoA=0.006; %(in mM)
PyrDHKmNAD=0.05; % (in mM)
PyrDHKmPyr=0.1; % (in mM)
PyrDHXNAD= 0.04; % (in mM)
PyrDHXCoA= 0.03; % (in mM)
EC_PDPCa= 1.0E-3; % (mM)
f_PDPNAD_H=6.7; % (mM)
n_NAD=0.2;
n_Coa=0.6;
f_PDKAcCoA=5.3; % (in mM)
EC_PDKPyr= 0.1; % (in mM)
EC_PDKATP=0.2;
EC_PDKADP=0.05;
Hi_cy=1.0E-4;
NADt_mat=1.0;
NADm=NADt_mat-NADHm ;
% Pyruvate Carrier
PyC_Vmax=4.125E-4; % mM ms-1
PyC_KmPyr=0.15; % mM;
PyC_Kh1=7.5E-6;
Cic=0.01;
CiCKm_Cic=0.036;
CiCKm_MALc=0.17;
CiCVmax=0.01;
CiCKm_Cim=0.288;
CiCKm_Malm=0.057;
CiCKh1=2.E-4;
CicKh2=7.5E-6;
RhoF1=1.5;
ANTVm=3.15;
```

```
GLUm=1.0E-3;
ASPm=0.2;
MALc=0.2;
RhoREN=2.0E-1;
RhoSDH=1.7E-1;
gh=1.0E-7;
ketf=6.949E16; ;
RhoETF=0.15; ;
kresf=5.765E13;
KeqF1=1.71E6;
Cai=1.0E-4;
beta_matr=1e-5; %Mitochondrial buffering capacity
Cadm=1.5; % total cyto adenon mitochondrial
Conc_NHE=0.00785;
%TCA cycle enzyme activities
KcsicoA=0.056;
KcsacCoA=4.4E-3;
KcsiacCoA=4.4E-3;
KcsOaa=3.3E-3;
KCS=0.06;
kfACO=1.796E-2;
KeqACO=2.22;
kfidh=5.376;
kfKG=0.052;
KfSL=0.056;
kfFH=0.000415*2;
kfMDH=0.01242;
kfAATm=0.00107*2;
KSLeq=3.115;
IDHkh_1=1E-5;
IDHkh_2=9E-4;
IDHkh_1a=4E-5;
IDHkh_2a=7E-5;
MDHKm_MALm=5.6E-2;
MDHKm_NADm=0.056;
MDHKm_Oaam=0.0348;
MDHKm_NADHm=0.0145;
MDHKi_Oaam=0.018;
MDHKi_NADHm=0.2;
MDHKi_MALm=3.5E-2;
KeqMDH=1.0E-4;
V1oV2m=(KeqMDH*MDHKm_NADm*MDHKi_MALm/(MDHKi_Oaam*MDHKm_NADHm)^(1
/2));
MDHKi_NAD=(V1oV2m)/KeqMDH*MDHKi_NADHm*MDHKm_Oaam/MDHKm_MALm;
MDHkhm1=1.131E-5;
MDHkhm2=2.67E1;
MDHkhm3=6.68E-9;
```

```
MDHkhm4=5.62E-6;
MDHkmoff=3.99E-2;
Mg=0.4;
KkgCa= 1.5E-4;
VmPiC=6.0E-1;
Cimat=1.812E-3;
Dpsio=0.0;
RToF=2.670818E1;
VNai=10.0;
kCai=1.5;
k2Cai=1.8E-3;
Vmuni= 0.04459;
VmNaCa=1.0E-7;
fCam = 3.0E-4;
ATPm = Cadm - ADPm;
% Acid-base dissociation equilibria and polynomia
H_mito_1000= H_mito/1000;
Mg_1000=Mg/1000;
KaidADP=6.2E-1;
KaidCa=5.0E-4;
KaPi=1.78E-7;
KaATP=3.31E-7;
KMgATP=6.46E-5;
KaADP=4.17E-7;
KMgADP= 5.62E-4;
KaSUC=6.3E-6;
KaH2O=1.0E-14;
ATP4 = ATPm / ( 1+ H_mito_1000/KaATP + Mg_1000/ KMgATP);
ATPMg= ATP4 * Mg_1000 / KMgATP;
HATP=  ATP4 * H_mito_1000 / KaATP ;
ADP3=  ADPm / ( 1+ H_mito_1000/KaADP + Mg_1000/ KMgADP);
% ADPMg= ADP3 * Mg_1000/KMgADP;
HADP=  ADP3 * H_mito_1000/KaADP ;
H2Pi = Pim/(1+ KaPi/H_mito_1000);
ATP4_c= ATPc/(1+ Hi_cy/1000/KaATP + 1/1000/KMgATP);
ADP3_c= (CadTcy-ATPc)/ (1+ Hi_cy/1000/KaADP + 1/1000/KMgADP);
polyATP=1+ H_mito_1000/KaATP + Mg_1000/KMgATP;
polyADP=1+ H_mito_1000/KaADP + Mg_1000/KMgADP;
polyPi = 1+ H_mito_1000/KaPi;
polyH2O =1+ H_mito_1000/KaH2O;
SUC_poly=1+ H_mito_1000/KaSUC;
% pyruvate carboxylase
PyrCb=(1/(1+H_mito/PyrCbK1+PyrCbK2/H_mito))*PyrCbVmax*Pyrm*ATPMg/(PyrCbKmPyr*A
TPMg+PyrCbKmATP*Pyrm+Pyrm*ATPMg)*(AcCoA_m^2/(PyrCbKACoa^2+AcCoA_m^2));
PyrCb=(1/(1+H_mito/PyrCbK1+PyrCbK2/H_mito))*PyrCbVmax*Pyrm*ATPMg/(PyrCbKmPyr*A
TPMg+PyrCbKmATP*Pyrm+Pyrm*ATPMg);
% beta-oxidation
```

```
sf_cpt1C16=1;
V_cpt1=3.0E-2;
Kmcpt1_C16CoA_cy=0.0138;
Kmcpt1_Carn_cy=0.125;
Kmcpt1_C16Carn_cy=1.36;
Kmcpt1_CoA_cy=0.407;
Kicpt1_MalCoA=0.0091;
Keq_cpt1=0.45;
n_cpt1=2.4799;
MalCoA_cy=0;
Carn_cy= 0.400;
CoA_cy=1.40;
Carn_mat=0.950;
C16CoA_cy= 0.01;
Vf_cact=2.1E1;
Vr_cact= 2.1E-5;
Kmcact_C16Carn_cy=0.01;
Kmcact_Carn_mat=0.130;
Kmcact_C16Carn_mat=0.015;
Kmcact_Carn_cy=0.130;
Kicact_C16Carn_cy=0.056;
Kicact_Carn_cy=0.200;
Keq_cact=1;
sf_cpt2C16=0.85;
V_cpt2=6.517E-3*30;
Kmcpt2_C16Carn_mat=0.051;
Kmcpt2_CoA_mat=0.030;
Kmcpt2_C16CoA_mat=0.038;
Kmcpt2_Carn_mat=0.350;
Keq_cpt2=2.22;
FADt_mat=0.7;
NADt_mat=1.0;
CoAT_mat=1.0;
sf_vlcadC16=1;
sf_vlcadC14=0.80;
sf_vlcadC12=0.42;
V_vlcad=6.67E-4*30;
Kmvlcad_C16CoA_mat=0.0065;
Kmvlcad_C14CoA_mat=0.004;
Kmvlcad_C12CoA_mat=0.0027;
Kmvlcad_FAD_mat=0.00012;
Kmvlcad_C16EnoylCoA_mat=0.00108;
Kmvlcad_C14EnoylCoA_mat=0.00108;
Kmvlcad_C12EnoylCoA_mat=0.00108;
Kmvlcad_FADH_mat=0.0242;
Keq_vlcad=6;
sf_lcadC16=0.9;
```

sf_lcadC14=1;
sf_lcadC12=0.95;
sf_lcadC10=0.85;
sf_lcadC8=0.4;
V_lcad=8.33E-4*30;
Kmlcad_C16CoA_mat=0.0025;
Kmlcad_C14CoA_mat=0.0074;
Kmlcad_C12CoA_mat=0.009;
Kmlcad_C10CoA_mat=0.0243;
Kmlcad_C8CoA_mat=0.123;
Kmlcad_FAD_mat=1.2E-6;
Kmlcad_C16EnoylCoA_mat=0.00108;
Kmlcad_C14EnoylCoA_mat=0.00108;
Kmlcad_C12EnoylCoA_mat= 0.00108;
Kmlcad_C10EnoylCoA_mat= 0.00108;
Kmlcad_C8EnoylCoA_mat= 0.00108;
Kmlcad_FADH_mat=0.0242;
Keq_lcad=6;
sf_mcadC12=0.68;
sf_mcadC10= 0.8;
sf_mcadC8= 0.87;
sf_mcadC6= 1;
sf_mcadC4= 0.12;
V_mcad= 8.35E-2*30;
Kmmcad_C12CoA_mat= 0.0057;
Kmmcad_C10CoA_mat= 0.0054;
Kmmcad_C8CoA_mat= 0.004;
Kmmcad_C6CoA_mat= 0.0094;
Kmmcad_C4CoA_mat= 0.135;
Kmmcad_FAD_mat= 0.00012;
Kmmcad_C12EnoylCoA_mat= 0.00108;
Kmmcad_C10EnoylCoA_mat= 0.00108;
Kmmcad_C8EnoylCoA_mat= 0.00108;
Kmmcad_C6EnoylCoA_mat= 0.00108;
Kmmcad_C4EnoylCoA_mat= 0.00108;
Kmmcad_FADH_mat=0.0242;
Keq_mcad=6;
sf_scadC6=0.3;
sf_scadC4=1;
V_scad= 8.35E-2*30;
Kmscad_C6CoA_mat=0.285;
Kmscad_C4CoA_mat= 0.0107;
Kmscad_FAD_mat= 0.00012;
Kmscad_C6EnoylCoA_mat= 0.00108;
Kmscad_C4EnoylCoA_mat= 0.00108;
Kmscad_FADH_mat=0.0242;
Keq_scad=6;

```
sf_crotC16=0.13;
sf_crotC14=   0.2;
sf_crotC12=   0.25;
sf_crotC10=   0.33;
sf_crotC8=    0.58;
sf_crotC6=    0.8;
sf_crotC4=    1;
V_crot=9.0E-2*30;
Kmcrot_C16EnoylCoA_mat=         0.150;
Kmcrot_C14EnoylCoA_mat=         0.100;
Kmcrot_C12EnoylCoA_mat=         0.025;
Kmcrot_C10EnoylCoA_mat=         0.025;
Kmcrot_C8EnoylCoA_mat= 0.025;
Kmcrot_C6EnoylCoA_mat= 0.025;
Kmcrot_C4EnoylCoA_mat= 0.040;
Kmcrot_C16OHCoA_mat=0.045;
Kmcrot_C14OHCoA_mat=0.045;
Kmcrot_C12OHCoA_mat=0.045;
Kmcrot_C10OHCoA_mat=0.045;
Kmcrot_C8OHCoA_mat=0.045;
Kmcrot_C6OHCoA_mat=0.045;
Kmcrot_C4OHCoA_mat=0.045;
Keq_crot=3.13;
AcacetylCoA_mat=C4KetoCoA_m;
Kicrot_AcacetylCoA_mat=0.0016;
sf_mschadC16=0.6;
sf_mschadC14=0.5;
sf_mschadC12=0.43;
sf_mschadC10=       0.64;
sf_mschadC8=        0.89;
sf_mschadC6=        1;
sf_mschadC4=        0.67;
V_mschad=1.6667E-1*30;
Kmmschad_C16OHCoA_mat=        0.0015;
Kmmschad_C14OHCoA_mat=        0.0018;
Kmmschad_C12OHCoA_mat=        0.0037;
Kmmschad_C10OHCoA_mat=        0.0088;
Kmmschad_C8OHCoA_mat=         0.0163;
Kmmschad_C6OHCoA_mat=         0.0286;
Kmmschad_C4OHCoA_mat=         0.0699;
Kmmschad_NAD_mat=0.0117;
Kmmschad_C16KetoCoA_mat=      0.0014;
Kmmschad_C14KetoCoA_mat=      0.0014;
Kmmschad_C12KetoCoA_mat=      0.0016;
Kmmschad_C10KetoCoA_mat=      0.0023;
Kmmschad_C8KetoCoA_mat=       0.0041;
Kmmschad_C6KetoCoA_mat=       0.0058;
```

```
Kmmschad_C4KetoCoA_mat=        0.0169;
Kmmschad_NADH_mat= 0.054;
Keq_mschad=2.17*10-4;
sf_mckatC16=0.2;
sf_mckatC14=0.2;
sf_mckatC12=0.38;
sf_mckatC10=0.75;
sf_mckatC8=0.81;
sf_mckatC6=1;
sf_mckatC4=0.89;
V_mckat=6.28E-2*30;
Kmmckat_C16KetoCoA_mat=        0.0011;
Kmmckat_C14KetoCoA_mat=        0.0012;
Kmmckat_C12KetoCoA_mat=        0.0013;
Kmmckat_C10KetoCoA_mat=        0.0021;
Kmmckat_C8KetoCoA_mat=         0.0032;
Kmmckat_C6KetoCoA_mat=         0.0067;
Kmmckat_C4KetoCoA_mat=         0.0124;
Kmmckat_CoA_mat=0.0266;
Kmmckat_C16CoA_mat=     0.01383;
Kmmckat_C14CoA_mat=     0.01383;
Kmmckat_C12CoA_mat=     0.01383;
Kmmckat_C10CoA_mat=     0.01383;
Kmmckat_C8CoA_mat=      0.01383;
Kmmckat_C6CoA_mat=      0.01383;
Kmmckat_C4CoA_mat=      0.01383;
Kmmckat_AcCoA_mat=      0.3;
Keqmckat=1051;
sfmtpC16=1.5;
sfmtpC14=0.9;
sfmtpC12=0.81;
sfmtpC10=0.73;
sfmtpC8=0.64;
V_mtp=3.0E-3;
Kmmtp_C16EnoylCoA_mat=0.025;
Kmmtp_C14EnoylCoA_mat=0.025;
Kmmtp_C12EnoylCoA_mat=0.025;
Kmmtp_C10EnoylCoA_mat=0.025;
Kmmtp_C8EnoylCoA_mat=0.025;
Kmmtp_NAD_mat=0.06;
Kmmtp_CoA_mat=0.3;
Kmmtp_C16CoA_mat=       0.01383;
Kmmtp_C14CoA_mat=       0.01383;
Kmmtp_C12CoA_mat=       0.01383;
Kmmtp_C10CoA_mat=       0.01383;
Kmmtp_C8CoA_mat=        0.01383;
Kmmtp_C6CoA_mat=        0.01383;
```

```
Kmmtp_NADH_mat=          0.050;
Kmmtp_AcCoA_mat=0.030;
Kimtp_AcetoacetylCoA_mat= 0.030;
Keqmtp=0.71;
%Antioxidant systems and ROS production
kIDHNADP=8.72E-5;
kTHD=1.1875E-4;
kgrxm=3.6E-3;
kgrx=3.6E-3;
KeqGRX=1.37E-3;
GrxT= 0.002;
Vmimac=3.9085E-6;
shunt= 0.01;
EtCuZnSOD=3e-3;
EtMnSOD=3e-3;
NADPm_T=1.0E-1;
PSSGT= 0.001;         %PSSG total;
kcatPSH= 0.64;
EtPSH=0.8e-3;
kmGSH=0.75;
kactH2O2=1e-3;
KmGrx=0.01;
KmPSSG= 0.0005;
difH2O2_c=8.0E-3;
VGST_c=1.5E-8;
EtGPXm=10e-5;
EtGRm=1e-4;
kcat=1.7E1;
Etcat=5.0E-4;
EtGPX=1.E-2;
kGR=2.5E-3;
EtGR=5.0E-3;
EoTxPXm= 2.5;
EoTxPX=0.1;
Phi2Trx= 1.85;
Phi1Trx= 3.83;
TrxTm=0.025;
TrxT= 0.05;
Etxrm= 0.01;
Etxr=0.005;
KMTrxSS= 0.035;
KMnadph= 0.012;
kcXR= 22.75E-3;
% General parameters and expressions conservation relations
Vlcyt= 4.0;
Vlmat= 1.0;
NAD=NADt_mat-NADHm;
```

```
CoA_mat=CoAT_mat-(SCoA+C16CoA_m+C16EnoylCoA_m+C16OHCoA_m+C16KetoCoA_m+
C14CoA_m+C14EnoylCoA_m+C14OHCoA_m+C14KetoCoA_m+C12CoA_m+C12EnoylCoA_m+
C12OHCoA_m+C12KetoCoA_m+C10CoA_m+C10EnoylCoA_m+C10OHCoA_m+
C10KetoCoA_m+C8CoA_m+C8EnoylCoA_m+C8OHCoA_m+   C8KetoCoA_m+C6CoA_m+
C6EnoylCoA_m+C6OHCoA_m+C6KetoCoA_m+C4CoA_m+C4EnoylCoA_m+C4OHCoA_m+
C4KetoCoA_m+AcCoA_m);
DpH=-log10(1.0E-4)+log10(H_mito);
V4=-2.303*2.670818E1*DpH+Dpsim;
% cytosolic metabolism of glucose
GLUT4V=GLUT4_kcat*(Glco-Glci);
HKV=HKVmax*(ATPc*Glci-(CadTcy-ATPc)*G6P/HKKapp)/(ATPc*Glci+HKKmAT*Glci
+ATPc*HKKmGLC+HKKmAT*HKKmGLC*(1+ G6P/HKKmG6P+(CadTcy-ATPc)/HKKmAD
+(CadTcy-ATPc)*G6P/HKKmAD/HKKmG6P));
%
PFKL=PFKLo*(((1+ATPc/PFKKiATP)/(1+d*ATPc/PFKKiATP))*((1+e*AMP/PFKKaAMP)/(1+A
MP/PFKKaAMP)))^4;
PFKLc=PFKLo*(((1+ATPc/PFKKiATP)/(1+PFK_d*ATPc/PFKKiATP))*((1+PFK_e*AMP_c/
PFKKaAMP)/(1+AMP_c/PFKKaAMP))*((1+kmrgd(89)/PFKKiCit)/(1+PFK_f*kmrgd(89)/PFKKiC
it)))^4;
PFKLc=PFKLo*(((1+ATPc/PFKKiATP)/(1+PFK_d*ATPc/PFKKiATP))*((1+
PFK_e*AMP_c/PFKKaAMP)/(1+AMP_c/PFKKaAMP))*((1+Cic/PFKKiCit)/(1+
PFK_f*Cic/PFKKiCit)))^4;
PFKVmaxr=PFKVmax*PFKKmAD*PFKKmFBP/(PFKKmF6P*PFKKmAT*PFKKeq);
PFKalpha=PFKKmF6P*PFKKmAT/PFKKmpF6P/PFKKmpAT;
PFKDelta=(1+G6P/PFKKmF6P)*(1+ATPc/PFKKmAT)+(CadTcy-
ATPc)/PFKKmAD+FbP/PFKKmFBP*(1+(CadTcy-ATPc)/PFKKmAD);
PFKDeltap=(1+G6P/PFKKmpF6P)*(1+ATPc/PFKKmpAT)+(CadTcy-
ATPc)/PFKKmpAD+FbP/PFKKmpFBP*(1+(CadTcy-ATPc)/PFKKmpAD);
PFKV=(PFKVmax*(ATPc*G6P/PFKKmF6P/PFKKmAT)-PFKVmaxr*((CadTcy-
ATPc)*FbP/PFKKmFBP/PFKKmAD))/PFKDelta*(1+...
   PFKalpha*PFKLc*(PFKDeltap/PFKDelta)^3)/(1+PFKLc*(PFKDeltap/PFKDelta)^4);
%
ALDVmaxr=ALDVmax*ALDKmG3P^2/ALDKmFBP/ALDKeq;
ALDV=(ALDVmax*FbP/ALDKmFBP-
ALDVmaxr*(G3P/ALDKmG3P)^2)/(1+FbP/ALDKmFBP+2*G3P/ALDKmG3P);
%
GAPDVmaxr=GAPDVmax*GAPDKmG13BP/GAPDKmG3P*GAPDKmNADH/GAPDKmNAD
/GAPDKmPi/GAPDKeq;
GAPDDen=1+G3P/GAPDKmG3P+((NDP-NADHc)/GAPDKmNAD)^nGDH
+Pi_cy/GAPDKmPi+G3P/GAPDKmG3P*((NDP-NADHc)/GAPDKmNAD)^nGDH
   +G3P/GAPDKmG3P*((NDP-NADHc)/GAPDKmNAD)^nGDH*Pi_cy/GAPDKmPi
+G13bP/GAPDKmG13BP+(NADHc/GAPDKmNADH)^nGDH+G13bP/GAPDKmG13BP*
(NADHc/GAPDKmNADH)^nGDH;
GAPDHV=(GAPDVmax*G3P/GAPDKmG3P*((NDP-NADHc)
/GAPDKmNAD)^nGDH*Pi_cy/GAPDKmPi-GAPDVmaxr*(G13bP/GAPDKmG13BP
*(NADHc/GAPDKmNADH)^nGDH))/GAPDDen;
%
```

```
PGKVmaxr=PGKVmax*(PGKKmGP3/PGKKmG13BP*PGKKmATP/PGKKmADP/PGKKeq);
PGKDen=1+G13bP/PGKKmG13BP+(CadTcy-ATPc)/PGKKmADP+
G13bP/PGKKmG13BP*(CadTcy-ATPc)/PGKKmADP+GP3/PGKKmGP3+ATPc/PGKKmATP
+GP3/PGKKmGP3*ATPc/PGKKmATP;
PGKV=(PGKVmax*G13bP/PGKKmG13BP*(CadTcy-ATPc)/PGKKmADP -
PGKVmaxr*(GP3/PGKKmGP3 *ATPc/PGKKmATP))/PGKDen;
%
ENOLVmaxr=ENOLVmax*(ENOLKmPEP/ENOLKmGP3/ENOLKeq);
ENOLV=(ENOLVmax*GP3/ENOLKmGP3-
ENOLVmaxr*PEP/ENOLKmPEP)/(1+GP3/ENOLKmGP3+PEP/ENOLKmPEP);
%
PKVmaxr=PKVmax*PFKKmAT*PKKmPyr/PKKmPEP/PKKmAD/PKKeq;
PKL= 1*10^-(6.8)*1000/Hi_cy/((1+ PEP/PKKmPEP+Pyr/PKKmPyr)^4*(1
+FbP/PKKmFBP+GDP/PKKmGDP)^4);
PKrho=1/(1+PKL);
PKV=(PKVmax*((CadTcy-ATPc)*PEP/PKKmPEP/PKKmAD)-PKVmaxr*(ATPc*
Pyr/PKKmPyr/PKKmAT)) /(1+PEP/PKKmPEP+(CadTcy-ATPc)/PKKmAD+(CadTcy-
ATPc)/PKKmAD*PEP/PKKmPEP+Pyr/PKKmPyr+ATPc/PKKmAT
+Pyr/PKKmPyr*ATPc/PKKmAT)*PKrho;
%
LDHVmaxr=LDHVmax*LDHKmLAC*LDHKmNAD/LDHKmNADH/LDHKmPyr/LDHKeq;
LDHV=(LDHVmax*(NADHc*Pyr/LDHKmPyr/LDHKmNADH) -LDHVmaxr*((NDP-NADHc)
*LAC/LDHKmLAC/LDHKmNAD))/(1+Pyr/LDHKmPyr+NADHc/LDHKmNADH
+NADHc/LDHKmNADH*Pyr/LDHKmPyr+(NDP-NADHc)/LDHKmNAD+LAC/LDHKmLAC
+(NDP-NADHc)/LDHKmNAD*LAC/LDHKmLAC);
%
VAmil=(Vmaxamil*GLY/Kmamil-Maltoside/Kmamil)/(1+GLY/Kmamil+Maltoside/KmMaltoside);
VrmaxGPa=VmaxGPa*KaGlyb*Kaig1p/(KaiGlyf*Kapi*KeqGP);
VGPa=(VmaxGPa*Maltoside*Pi_cy/(KaiGlyf*Kapi)-
VrmaxGPa*(Maltoside*G1P/(KaGlyb*Kaig1p))))/(1+Maltoside/KaiGlyf+Pi_cy/Kiapi+
Maltoside/KaGlyb+G1P/Kaig1p+Maltoside/KaGlyf*Pi_cy/Kiapi+Maltoside/KaGlyb*G1P/Kaig1p);
VrmaxGPb=VmaxGPb*KibGlyb*Kbig1p/(KibGlyf*Kbpi*KeqGP);
VGPb=(VmaxGPb*Maltoside*Pi_cy/(KibGlyf*Kbpi)-
VrmaxGPb*(Maltoside*G1P/(KibGlyb*Kbg1p))))/(1+Maltoside/KibGlyf+Pi_cy/Kibpi+
Maltoside/KibGlyb+G1P/Kbig1p+Maltoside/KibGlyf*Pi_cy/Kibpi+Maltoside/KibGlyb*
G1P/Kbig1p)*(AMP_c^nH/KpAMP)/(1+AMP_c^nH/KpAMP);
VmaxrPGLM=VmaxPGLM*KG6P/(KG1P*KeqPGLM);
VPGLM=(VmaxPGLM*G1P/KG1P-VmaxrPGLM*G6P/KG6P)/(1+G6P/KG6P+G1P/KG1P);
%Pentose Phosphate pathway
G6PDHV=G6PDHVmax*G6P*(NTP-NADPHc)/G6PDHKmG6P/G6PDHKmNADP/(1+(NTP-
NADPHc)/G6PDHKmNADP*(1+G6P/G6PDHKmG6P)+NADPHc/G6PDHKmNADPH...
    +ATPc/G6PDHKmATP);
N1=P6GDHk1*P6GDHk3*P6GDHk5*P6GDHk7*P6GDHk9;
N2=P6GDHk2*P6GDHk4*P6GDHk6*P6GDHk8*P6GDHk10;
D1=P6GDHk2*P6GDHk9*(P6GDHk4*P6GDHk6+P6GDHk5*P6GDHk6+P6GDHk5*P6GDHk7);
D2=P6GDHk1*P6GDHk9*(P6GDHk4*P6GDHk6+P6GDHk5*P6GDHk6+P6GDHk5*P6GDHk7);
D3=P6GDHk3*P6GDHk5*P6GDHk7*P6GDHk9;
```

D4=P6GDHk2*P6GDHk4*P6GDHk6*P6GDHk8;
D5=P6GDHk2*P6GDHk10*(P6GDHk4*P6GDHk6+P6GDHk5*P6GDHk6+P6GDHk5*P6GDHk7);
D6=P6GDHk1*P6GDHk3*(P6GDHk5*P6GDHk5+P6GDHk5*P6GDHk9+P6GDHk6*P6GDHk9+P6GDHk7*P6GDHk9);
D7=P6GDHk1*P6GDHk4*P6GDHk6*P6GDHk8;
D8=P6GDHk3*P6GDHk5*P6GDHk7*P6GDHk10;
D9=P6GDHk8*P6GDHk10*(P6GDHk2*P6GDHk4+P6GDHk2*P6GDHk5+P6GDHk2*P6GDHk6+P6GDHk4*P6GDHk6);
D10=P6GDHk1*P6GDHk3*P6GDHk8*(P6GDHk5*P6GDHk6);
D11=P6GDHk3*P6GDHk8*P6GDHk10*(P6GDHk5*P6GDHk6);
P6GDHDen=D1+D2*(NTP-NADPHc)+D3*GP6+D4*Ru5P+D5*NADPHc+D6*GP6*(NTP-NADPHc)+D7*Ru5P*(NTP-NADPHc) +D8*GP6*NADPHc+D9*Ru5P*NADPHc +D10*GP6*(NTP-NADPHc)*Ru5P+D11*GP6*Ru5P*NADPHc;
P6GDHV=P6GDHVmax*(N1*GP6*(NTP-NADPHc)-N2*Ru5P*NADPHc)/P6GDHDen;
R5PIV=R5PIVmax*(R5PIk3*Ru5P/R5PIKmRu5P-R5PIk2*R5P/R5PIKmR5P)/(1 +Ru5P/R5PIKmRu5P+R5P/R5PIKmR5P);
Ru5PEV=Ru5PEVmax*(Ru5PEk3* Ru5P/Ru5PEKmRu5P-Ru5PEk2*X5P/Ru5PEKmXu5P)/(1+Ru5P/Ru5PEKmRu5P+X5P/Ru5PEKmXu5P);
NAc1=RTAck1*RTAck3*RTAck5*RTAck7;
NAc2=RTAck2*RTAck4*RTAck6*RTAck8;
DAc1=RTAck1*RTAck3*(RTAck6+RTAck7);
DAc2=RTAck5*RTAck7*(RTAck2+RTAck3);
DAc3=RTAck2*RTAck4*(RTAck6+RTAck7);
DAc4=RTAck6*RTAck8*(RTAck2+RTAck3);
DAc5=RTAck1*RTAck5*(RTAck3+RTAck7);
DAc6=RTAck4*RTAck8*(RTAck2+RTAck6);
DAc7=RTAck5*RTAck8*(RTAck2+RTAck3);
DAc8=RTAck1*RTAck4*(RTAck6+RTAck7);
RTAcDen=DAc1*X5P+DAc2*R5P+DAc3*G3P+DAc4*S7P+DAc5*X5P*R5P+DAc6*G3P*S7P+DAc7*R5P*S7P +DAc8*G3P*X5P;
RTAcV=RTAcVmax*(NAc1*X5P*R5P-NAc2*G3P*S7P)/RTAcDen;
NAc21=RTAc2k1*RTAc2k3*RTAc2k5*RTAc2k7;
NAc22=RTAc2k2*RTAc2k4*RTAc2k6*RTAc2k8;
DAc21=RTAc2k1*RTAc2k3*(RTAc2k6+RTAc2k7);
DAc22=RTAc2k5*RTAc2k7*(RTAc2k2+RTAc2k3);
DAc23=RTAc2k2*RTAc2k4*(RTAc2k6+RTAc2k7);
DAc24=RTAc2k6*RTAc2k8*(RTAc2k2+RTAc2k3);
DAc25=RTAc2k1*RTAc2k5*(RTAc2k3+RTAc2k7);
DAc26=RTAc2k4*RTAc2k8*(RTAc2k2+RTAc2k6);
DAc27=RTAc2k5*RTAc2k8*(RTAc2k2+RTAc2k3);
DAc28=RTAc2k1*RTAc2k4*(RTAc2k6+RTAc2k7);
RTAc2Den=DAc21*X5P+DAc22*E4P+DAc23*G3P+DAc24*G6P+DAc25*X5P*E4P+DAc26*G3P*G6P+DAc27*E4P*G6P+DAc28*X5P*G3P;
RTAc2V=RTAc2Vmax*(NAc21*X5P*E4P-NAc22*G3P*G6P)/RTAc2Den;
NAl1=RTAlk1*RTAlk3*RTAlk5*RTAlk7;
NAl2=RTAlk2*RTAlk4*RTAlk6*RTAlk8;
DAl1=RTAlk1*RTAlk3*(RTAlk6+RTAlk7);

DAl2=RTAlk5*RTAlk7*(RTAlk2+RTAlk3);
DAl3=RTAlk2*RTAlk4*(RTAlk6+RTAlk7);
DAl4=RTAlk6*RTAlk8*(RTAlk2+RTAlk3);
DAl5=RTAlk1*RTAlk5*(RTAlk3+RTAlk7);
DAl6=RTAlk4*RTAlk8*(RTAlk2+RTAlk6);
DAl7=RTAlk5*RTAlk8*(RTAlk2+RTAlk3);
DAl8=RTAlk1*RTAlk4*(RTAlk6+RTAlk7);
RTAlDen=DAl1*S7P+DAl2*G3P+DAl3*E4P+DAl4*G6P+DAl5*S7P*G3P+DAl6*E4P*G6P+
DAl7*G3P*G6P+DAl8*S7P*E4P;
RTAlV=RTAlVmax*(NAl1*S7P*G3P-NAl2*E4P*G6P)/RTAlDen;
%
ALR1Den=DALR1+DALR2*NADPHc+DALR3*Glci+DALR4*Sor+DALR5*(NTP-
NADPHc)+DALR6*Glci*NADPHc+DALR7*Sor*NADPHc+DALR8*Glci *(NTP-NADPHc) +
DALR9*Sor*(NTP-NADPHc)+DALR10*Glci*NADPHc*Sor +DALR11*Glci*Sor*(NTP-
NADPHc);
ALR1V=ALDREmax*(ALR1V1*Glci*NADPHc-ALR1V2*Sor*(NTP-
NADPHc)/ALR1Keq)/ALR1Den;
SoDHVrmax=SoDHVmax*(SoDHKmFRU*SoDHKmNADH/SoDHKmSorb/SoDHKmNAD/SoDH
Keq);
SoDHV=SoDHVmax*(NDP-NADHc)*Sor/(SoDHKiNAD*SoDHKmSorb+SoDHKmSorb*(NDP-
NADHc)+SoDHKmNAD*Sor+(NDP-NADHc)*Sor) - SoDHVrmax*NADHc*FRU
/(SoDHKiNADH*SoDHKmFRU+SoDHKmFRU*NADHc+SoDHKmNADH*FRU+NADHc*FRU);
XyDHVrmax=XyDHVmax*(XyDHKmXy*XyDHKmNADH/XyDHKmXyOH/XyDHKmNAD
/XyDHKeq);
XyDHV=XyDHVmax*NADHc*X5P/(XyDHKiNADH*XyDHKmXy+XyDHKmXy*NADHc
+XyDHKmNADH*X5P+NADHc*X5P) -XyDHVrmax*(NDP-NADHc)*XyOH/(XyDHKiNAD
XyDHKmXyOH+XyDHKmXyOH(NDP-NADHc)+XyDHKmNAD*XyOH+(NDP-
NADHc)*XyOH);
%
HYdroATPV=HydrolVmax*ATPc/(HydroKmATP+ATPc);
NADHDemV=NADHDemVmax*(NADHc/NADHDemKmNADH-(NDP-
NADHc)/NADHDemKmNAD)/(1+NADHc/NADHDemKmNADH+(NDP-
NADHc)/NADHDemKmNAD);
FRUTV=ktfru*FRU/(kmFru+FRU);
XiOHTV=ktXyOH*XyOH/(kmXyOH+XyOH);
NADPHDemV=NADPHDemVmax*(NADPHc/NADPHDemKmNADPH-(NTP-NADPHc)
/NADPHDemKmNADP)/(1+NADPHc/NADPHDemKmNADPH+(NTP-NADPHc)
/NADPHDemKmNADP);
%% Pyruvate dehydrogenase, pyruvate carrier
Pdh_DP=(1+Cam/EC_PDPCa)*((NADm/NADHm)^n_NAD*f_PDPNAD_H+1);
Pdh_P=(1+(AcCoA_m/CoA_mat)^n_Coa*f_PDKAcCoA)*(1+ATPm/EC_PDKATP)*(1+
EC_PDKADP/ADPm)*(1+EC_PDKPyr/Pyrm);
AlphaPDH=Pdh_DP/(Pdh_P+Pdh_DP);
PyrDHDen=(1+Pyrm/PyrDHKmPyr)*(CoA_mat/PyrDHKmCoA +1+CoAT_mat/PyrDHXCoA*(1
+CoA_mat/CoAT_mat))/(NADm/PyrDHKmNAD+1+NADt_mat/PyrDHXNAD*(1+
NADm/NADt_mat));

```
PyrDH = PyrDHVmax*AlphaPDH*NADm/PyrDHKmNAD*Pyrm/PyrDHKmPyr*CoA_mat
/PyrDHKmCoA/PyrDHDen;
PyrCV=PyC_Vmax*Pyr/((Pyr+PyC_KmPyr)*(1+H_mito/PyC_Kh1));
VAATm=kfAATm*(GLUm*Oaa-aKGm*ASPm/KeqAAT);
% Citrate Carrier
VCiC=CiCVmax*(CITm/CiCKm_Cim*MALc/CiCKm_MALc - MALm/CiCKm_Malm*Cic
/CiCKm_Cic)/((1+MALm/CiCKm_Malm*Cic/CiCKm_Cic+ MALm/CiCKm_Malm+Cic
/CiCKm_Cic+MALc/CiCKm_MALc*CITm/CiCKm_Cim+MALc/CiCKm_MALc+
CITm/CiCKm_Cim)*(1+Hi_cy/CiCKh1+CicKh2/Hi_cy));
%% Metabolic rate expressions TCA cycle and oxidative phosphorilation
VAREN=1.35E18*sqrt(NADHm/NADm);
VNOden=1/((exp(3.0E2/2.670818E1)+2.077E-18*exp(3.0E2/2.670818E1)*VAREN) +  (1.728E-9
+1.059E-26*VAREN)*exp(5.1/2.670818E1*V4));
VHNe=6*RhoREN*(6.394E-13*VAREN-(6.394E-13 + 1.762E-16)*exp(5.1/2.670818E1*V4))
*VNOden;
VNO=0.5*RhoREN*((6.394E-13+2.656E-22*exp(3.0E2/2.670818E1)+8.632E-30*
exp(5.1/2.670818E1*V4))*VAREN-6.394E-13*exp(5.1/2.670818E1*V4))*VNOden;
Kresf_app=kresf/SUC_poly;
VARSDH=2.670818E1*(log(Kresf_app*sqrt(Succ/FUM)));
VHSDHden=RhoSDH/((1+2.077E-18*exp(VARSDH/2.670818E1))*exp(2.0E2/2.670818E1)
+(1.728E-9+ 1.059E-26*exp(VARSDH/2.670818E1))*exp(3.4/2.670818E1*V4))/(1+Oaa/1.5E-1);
VHSDH=4*(6.394E-13*exp(VARSDH/2.670818E1)-(6.394E-13+1.762E-16)*exp(3.4/2.670818E1*
V4))*VHSDHden;
FAD=(FADt_mat-FADH_m);
Ketf_app=ketf/polyH2O;
VARETF=2.670818E1*(log(Ketf_app*sqrt(FADH_m/FAD)));
VETFHden=RhoETF/((1+2.077E-18*exp(VARETF/2.670818E1))*exp(2.0E2/2.670818E1)
+(1.728E-9 + 1.059E-26*exp(VARETF/2.670818E1))*exp(3.4/2.670818E1*V4));
VETFH=4*(6.394E-13*exp(VARETF/2.670818E1)-(6.394E-13+1.762E-16)*exp(3.4/2.670818E1
*V4))*VETFHden;
VO2ETF=5.0E-1*((6.394E-13+2.656E-22*exp(2.0E2/2.670818E1))*exp(VARETF/2.670818E1)
 - (6.394E-13-8.632E-30*exp(VARETF/2.670818E1))*exp(3.4/2.670818E1*V4))*VETFHden;
KATPase_app=KeqF1*H_mito_1000*polyATP*polyH2O/(polyADP*polyPi);
VAF1=KATPase_app/Pim*(ATPMg/(ADP3+HADP));
VHuden=-RhoF1/(exp(1.5E2/2.670818E1) +1.346E-4*exp(1.5E2/2.670818E1)*VAF1 +
(7.739E-7+6.65E-15*VAF1)*exp(3/2.670818E1*V4));
VATPase=((1.656E-6+9.651E-17*exp(1.5E2/2.670818E1)-4.585E-17*exp(3/2.670818E1*V4))
*VAF1-1.656E-8*exp(3/2.670818E1*V4))*VHuden;
Vhu=(3.0E2*1.656E-8+3.0E2*1.656E-8*VAF1-3*(1.656E-8+3.373E-10)*exp(3/2.670818E1
*V4))*VHuden;
VANT=ANTVm*(1-(ATP4_c*ADP3)/(ADP3_c*ATP4))*exp(-Dpsim/2.670818E1)/((1
+ATP4_c/(ADP3_c)*exp(-1.8721E-2*Dpsim))*(1+ADP3/(ATP4)));
V2FRTdsi=2*(Dpsim-Dpsio)/RToF;
Vuniden=(((1+Cai/kCai)^4)+1.1E2/((1+Cai/k2Cai)^(2.8)))*(1-exp(-V2FRTdsi));
Vuni=(Vmuni/kCai*Cai*V2FRTdsi*(1+Cai/kCai)^3)/Vuniden;
VnaCa=VmNaCa*exp(2.5E-1*V2FRTdsi)*Cam/(Cai*((1+9.4/VNai)^3)*(1+3.75E-4/Cam));
Vhleak=gh*(1+100.0*C16CoA_cy^4/(C16CoA_cy+3.5E-2)^4)*V4;
```

```
VCS=KCS*AcCoA_m*Oaa/(KcsiacCoA*KcsOaa*(1+CoA_mat/KcsicoA)+KcsacCoA*(1+
CoA_mat/KcsicoA)*Oaa+KcsOaa*AcCoA_m+AcCoA_m*Oaa);
VACO=kfACO*(CITm-ISOm/KeqACO);
Va=1/((1+ADP3/KaidADP)*(1+Cam/KaidCa));
Vi=1+NADHm/1.9E-1;
Vb=1.0+H_mito/IDHkh_1+IDHkh_2/H_mito;
VIDH=kfidh*1.09E-1/(Vb+9.23E-1/NADm*Vi+((1.52E-2/ISOm)^2)*Va*(1+9.23E-1/NADm*Vi));
VKGa=1/((1+1.2987E1)*(1+Cam/KkgCa));
VKGDH=kfKG*5E-1/(1.0 +  H_mito/IDHkh_1a+IDHkh_2a/H_mito+VKGa*(3.0E1/aKGm)^1.2
+VKGa*(3.87E1/NADm));
KSLeq_app = KSLeq * SUC_poly * polyATP / (polyADP *polyPi );
VSL=KfSL*(SCoA*Pim*ADPm-Succ*(ATP4+HATP)*CoA_mat/KSLeq_app);
VO2SDH=5.0E-1*((6.394E-13+2.656E-22*exp(2.0E2/2.670818E1))*exp(VARSDH/2.670818E1)
-(6.394E-13-8.632E-30*exp(VARSDH/2.670818E1))*exp(3.4/2.670818E1*V4))*VHSDHden;
VFH=kfFH*(FUM-MALm/1);
V26=(1/(1+MDHkhm3/H_mito+MDHkhm3*MDHkhm4/(H_mito^2)))^2;
V27=1/(1+H_mito/MDHkhm1+(H_mito^2)/(MDHkhm1*MDHkhm2))+MDHkmoff;
VMDHm=kfMDH*V26*V27*(MALm*NADm)/((MDHKm_MALm*MDHKi_NAD+
MDHKm_MALm*NADm+MDHKm_NADm*MALm+NADm*MALm+MDHKm_NADm*MALm
* NADHm/MDHKi_NADHm+NADm*MALm*Oaa/MDHKi_Oaam)+V1oV2m/KeqMDH*
(MDHKm_NADHm*Oaa+MDHKm_Oaam*NADHm+Oaa*NADHm+MDHKm_NADHm*NADm
*Oaa/MDHKi_NAD+MALm*Oaa*NADHm/MDHKi_MALm));
%% Ionic transport expressions
Nam=-2/3.0E-4*Cam-ADPm-1.812E-3*Dpsim-H_mito/1.0E-5+Pim+1.01E1;
VBeta1p=(1.6E-1*2.4E1*Hi_cy)/(1.585E-4*2.4E1+2.4E1*Hi_cy+1.585E-4*VNai);
VBeta1n=(9.39E-2*2.4E1*H_mito)/(1.585E-4*2.4E1+2.4E1*H_mito+1.585E-4*Nam);
VBeta2p=(2.52E-2*1.585E-4*Nam)/(1.585E-4*2.4E1+2.4E1*H_mito+1.585E-4*Nam);
VBeta2n=(4.29E-2*1.585E-4*VNai)/(1.585E-4*2.4E1+2.4E1*Hi_cy+1.585E-4*VNai);
pHm=-log10(H_mito)+3;
VNaH=Conc_NHE*(((VBeta1p*VBeta2p)-(VBeta1n*VBeta2n))/(VBeta1p+VBeta1n
+VBeta2p+VBeta2n))/(1+10^(3*(pHm-8.52)));
VPiC=VmPiC/60000*(9.0E1*Pi_cy*1.0E-8/H_mito/(1.106E1*4.084E-5) -9.0E1*H2Pi*
1.0E-4/(1.106E1*4.084E-5))/(1+Pi_cy/1.106E1+1.0E-8/H_mito/4.084E-5+Pi_cy*1.0E-8/H_mito
/(1.106E1*4.084E-5)+H2Pi/1.106E1+1.0E-4/4.084E-5+  H2Pi*1.0E-4/(1.106E1*4.084E-5));
%% Production of oxygen radicals, antioxidant defenses and regeneration of NADPH
VNADPm=NADPm_T-NADPHm;
VdenID_NADP=(1+H_mito/5.0E-1)*(1+ISOm/3.9E-3+VNADPm/6.7E-3*(1+ 2.0E-6/VNADPm)
+aKGm/5.1E-1+NADPHm/1.2E-2+ISOm/3.9E-3*VNADPm/6.7E-3*(1+2.0E-6/VNADPm)
+aKGm/5.1E-1*NADPHm/1.2E-2+ISOm/3.9E-3*NADPHm/1.2E-2+aKGm/
5.1E-1*VNADPm/6.7E-3*(1+2.0E-6/VNADPm));
VIDH_NADP=(kIDHNADP*ISOm/3.9E-3*VNADPm/6.7E-3*(1+2.0E-6/VNADPm)-
kIDHNADP/16*aKGm/5.1E-1*NADPHm/1.2E-2)/VdenID_NADP;
VDNADP=exp(3.7441E-3*V4);
VDNAD=exp(1-3.7441E-3*V4);
VTHDen=1+NADHm/1.0E-2+NADm/1.25E-1+VNADPm/1.7E-2+NADPHm/2.0E-
2+NADHm/1.0E-2*VNADPm/1.7E-2*VDNADP+NADPHm/2.0E-2*NADHm/1.0E-2*VDNAD+
+NADm/1.25E-1*VNADPm/1.7E-2*VDNADP*VDNAD+NADHm/1.0E-2*NADPHm/2.0E-2;
```

```
VTHD=(kTHD*1.174737*NADHm/1.0E-2*VNADPm/1.7E-2*VDNADP-
10*kTHD*NADm/1.25E-1*NADPHm/2.0E-2*VDNAD)/VTHDen;
JH=-VHNe - VHSDH - VETFH + Vhu + VNaH + VPiC + Vhleak + VTHD;
%%
VIMAC=(1.0E-3+1.0E4/(1+1.0E-2/SO2i))*(3.5E-8+Vmimac/(1+exp(7.0E-2*(4.0
+Dpsim))))*Dpsim;
VtrROS=-1.0E-1*(-Dpsim-2.6730818E1*log(SO2m/SO2i))/Dpsim*VIMAC;
VMnSOD=2*1.2E3*2.5E-4*(1.2E3+2.4E1*(1+H2O2m/5.0E-1))*EtMnSOD*SO2m/
(2.5E-4*(2*1.2E3+2.4E1*(1+H2O2m/5.0E-1))+1.2E3*2.4E1*(1+H2O2m/5.0E-1)*SO2m);
VCuZnSOD=2*1.2E3*2.5E-4*(1.2E3+2.4E1*(1+H2O2i/5.0E-1))*EtCuZnSOD*SO2i/
(2.5E-4*(2*1.2E3+2.4E1*(1+H2O2i/5.0E-1))+1.2E3*2.4E1*(1+H2O2i/5.0E-1)*SO2i);
VGPXm=EtGPXm*H2O2m*GSHm/(5.0E-3*GSHm+7.5E-1*H2O2m);
VGRm=kGR*EtGRm/(1+6.0E-2/GSSGm+1.5E-2/NADPHm+6.0E-2/GSSGm*1.5E-2/NADPHm);
VGPX=EtGPX*H2O2i*GSHi/(5.0E-3*GSHi+7.5E-1*H2O2i);
VGR=kGR*EtGR/(1+6.0E-2/GSSGi+1.5E-2/NADPHc+6.0E-2/GSSGi*1.5E-2/NADPHc);
VGRXm=kgrxm*KeqGRX*GSHm^2*GrxT*PSSGm/((GSSGm+KeqGRX*GSHm^2)*(KeqGRX*
GSHm^2*GrxT/(GSSGm+KeqGRX*GSHm^2)+KmGrx)*(PSSGm+KmPSSG));
VGRX=kgrx*KeqGRX*GSHi^2*GrxT*PSSGi/((GSSGi+KeqGRX*GSHi^2)*(KeqGRX*GSHi^2*
GrxT/(GSSGi+KeqGRX*GSHi^2)+KmGrx)*(PSSGi+KmPSSG));
TrxSSm=TrxTm-TrxSH2m;
TrxSS=TrxT-TrxSH2;
VTxPXm = EoTxPXm*H2O2m*TrxSH2m/(Phi2Trx*TrxSH2m+Phi1Trx*H2O2m);
VTxPX = EoTxPX*H2O2i*TrxSH2/(Phi2Trx*TrxSH2+Phi1Trx*H2O2i);
VTxRm = txrm*kcXR/(1+KMTrxSS/TrxSSm+KMnadph/NADPHm+KMTrxSS/TrxSSm*KMnadph
/NADPHm);
VTxR = Etxr*kcXR/(1+KMTrxSS/TrxSS+KMnadph/NADPHc+KMTrxSS/TrxSS*KMnadph
/NADPHc);
VdifH2O2=difH2O2_c*(H2O2m-H2O2i);
Vcat=2*kcat*Etcat*H2O2i*exp(-5.0E-2*H2O2i);
VGST=VGST_c*(GSHi-GSHm)/(GSHi+2.6);
VPSSGm=kcatPSH*EtPSH*(PSSGT-PSSGm)/(1+(kmGSH/GSHm)/(1+(H2O2m/kactH2O2)));
VPSSGi=kcatPSH*EtPSH*(PSSGT-PSSGi)/(1+(kmGSH/GSHi)/(1+(H2O2i/kactH2O2)));
%% beta - Oxidation (from a liver model by van Eunen, …, and Bakker, 2012)

Vcpt1C16=sf_cpt1C16*V_cpt1*((C16CoA_cy*Carn_cy)/(Kmcpt1_C16CoA_cy *
Kmcpt1_Carn_cy)-(C16Carn_cy*CoA_cy)/(Kmcpt1_C16Carn_cy * Kmcpt1_CoA_cy *
Keq_cpt1))/((1+C16CoA_cy/Kmcpt1_C16CoA_cy +C16Carn_cy/Kmcpt1_C16Carn_cy
+(MalCoA_cy/Kicpt1_MalCoA)^(n_cpt1) )*(1+Carn_cy/Kmcpt1_Carn_cy
+CoA_cy/Kmcpt1_CoA_cy));

VcactC16=Vf_cact*((C16Carn_cy*Carn_mat-1.0E-6*(C16Carn_m*Carn_cy)/Keq_cact))
/(C16Carn_cy*Carn_mat+Kmcact_Carn_mat*C16Carn_cy+Kmcact_C16Carn_cy*Carn_mat*(1+
Carn_cy/Kicact_Carn_cy)+Vf_cact/(Vr_cact*Keq_cact)*(Kmcact_Carn_cy*C16Carn_m*(1+
C16Carn_cy/Kicact_C16Carn_cy)+Carn_cy*(Kmcact_C16Carn_mat+C16Carn_m)));

Vcpt2C16=sf_cpt2C16*V_cpt2*((C16Carn_m*CoA_mat)/(Kmcpt2_C16Carn_mat*
Kmcpt2_CoA_mat)-(C16CoA_m*Carn_mat)/(Kmcpt2_C16Carn_mat*Kmcpt2_CoA_mat*
Keq_cpt2))/((1+(C16Carn_m/Kmcpt2_C16Carn_mat+C16CoA_m/Kmcpt2_C16CoA_mat))*
(1+CoA_mat/Kmcpt2_CoA_mat+Carn_mat/Kmcpt2_Carn_mat));
```

VvlcadC16=sf_vlcadC16*V_vlcad*((C16CoA_m *(FADt_mat-FADH_m))/ (Kmvlcad_C16CoA_mat*Kmvlcad_FAD_mat)-(C16EnoylCoA_m*FADH_m) /(Kmvlcad_C16CoA_mat* Kmvlcad_FAD_mat*Keq_vlcad))/((1+(C12CoA_m /Kmvlcad_C12CoA_mat+ C12EnoylCoA_m/Kmvlcad_C12EnoylCoA_mat+ C14CoA_m/Kmvlcad_C14CoA_mat+C14EnoylCoA_m/Kmvlcad_C14EnoylCoA_mat+ C16CoA_m/Kmvlcad_C16CoA_mat+C16EnoylCoA_m/Kmvlcad_C16EnoylCoA_mat))* (1+(FADt_mat-FADH_m)/Kmvlcad_FAD_mat+FADH_m/Kmvlcad_FADH_mat));

VvlcadC14=sf_vlcadC14*V_vlcad*((C14CoA_m *(FADt_mat-FADH_m)) /(Kmvlcad_C14CoA_mat*Kmvlcad_FAD_mat)-(C14EnoylCoA_m*FADH_m) /(Kmvlcad_C14CoA_mat*Kmvlcad_FAD_mat*Keq_vlcad))/((1+(C12CoA_m /Kmvlcad_C12CoA_mat+ C12EnoylCoA_m/Kmvlcad_C12EnoylCoA_mat+C14CoA_m /Kmvlcad_C14CoA_mat+C14EnoylCoA_m/Kmvlcad_C14EnoylCoA_mat+C16CoA_m /Kmvlcad_C16CoA_mat+C16EnoylCoA_m/Kmvlcad_C16EnoylCoA_mat))*(1+ (FADt_mat-FADH_m)/Kmvlcad_FAD_mat+FADH_m/Kmvlcad_FADH_mat

VvlcadC12=sf_vlcadC12*V_vlcad*((C12CoA_m *(FADt_mat-FADH_m)) /(Kmvlcad_C12CoA_mat*Kmvlcad_FAD_mat)- (C12EnoylCoA_m*FADH_m) /(Kmvlcad_C12CoA_mat*Kmvlcad_FAD_mat*Keq_vlcad))/((1+(C12CoA_m /Kmvlcad_C12CoA_mat+ C12EnoylCoA_m/Kmvlcad_C12EnoylCoA_mat +C14CoA_m/Kmvlcad_C14CoA_mat+C14EnoylCoA_m/Kmvlcad_C14EnoylCoA_mat+ C16CoA_m/Kmvlcad_C16CoA_mat+C16EnoylCoA_m/Kmvlcad_C16EnoylCoA_mat))*(1+ (FADt_mat-FADH_m)/Kmvlcad_FAD_mat+FADH_m/Kmvlcad_FADH_mat));

VlcadC16=sf_lcadC16*V_lcad*((C16CoA_m *(FADt_mat-FADH_m))/(Kmlcad_C16CoA_mat* Kmlcad_FAD_mat)-(C16EnoylCoA_m*FADH_m)/(Kmlcad_C16CoA_mat* Kmlcad_FAD_mat*Keq_lcad))/((1+(C8CoA_m/Kmlcad_C8CoA_mat+C8EnoylCoA_m /Kmlcad_C8EnoylCoA_mat+ C10CoA_m/Kmlcad_C10CoA_mat+C10EnoylCoA_m /Kmlcad_C10EnoylCoA_mat+C12CoA_m/Kmlcad_C12CoA_mat+ C12EnoylCoA_m /Kmlcad_C12EnoylCoA_mat+C14CoA_m/Kmlcad_C14CoA_mat+C14EnoylCoA_m /Kmlcad_C14EnoylCoA_mat+C16CoA_m/Kmlcad_C16CoA_mat+C16EnoylCoA_m /Kmlcad_C16EnoylCoA_mat))*(1+(FADt_mat-FADH_m)/Kmlcad_FAD_mat+ FADH_m/Kmlcad_FADH_mat));

VlcadC14=sf_lcadC14*V_lcad*((C14CoA_m *(FADt_mat-FADH_m))/(Kmlcad_C14CoA_mat *Kmlcad_FAD_mat)-(C14EnoylCoA_m*FADH_m)/(Kmlcad_C14CoA_mat*Kmlcad_FAD_mat *Keq_lcad))/((1+(C8CoA_m/Kmlcad_C8CoA_mat+C8EnoylCoA_m/Kmlcad_C8EnoylCoA_mat+ C10CoA_m/Kmlcad_C10CoA_mat+C10EnoylCoA_m/Kmlcad_C10EnoylCoA_mat+ C12CoA_m/Kmlcad_C12CoA_mat+ C12EnoylCoA_m/Kmlcad_C12EnoylCoA_mat+C14CoA_m /Kmlcad_C14CoA_mat+C14EnoylCoA_m/Kmlcad_C14EnoylCoA_mat+C16CoA_m /Kmlcad_C16CoA_mat+C16EnoylCoA_m/Kmlcad_C16EnoylCoA_mat))*(1+(FADt_mat-FADH_m)/Kmlcad_FAD_mat+FADH_m/Kmlcad_FADH_mat));

VlcadC12=sf_lcadC12*V_lcad*((C12CoA_m *(FADt_mat-FADH_m))/(Kmlcad_C12CoA_mat* Kmlcad_FAD_mat)-(C12EnoylCoA_m*FADH_m)/(Kmlcad_C12CoA_mat*Kmlcad_FAD_mat*

Keq_lcad))/((1+(C8CoA_m/Kmlcad_C8CoA_mat+C8EnoylCoA_m/Kmlcad_C8EnoylCoA_mat+ C10CoA_m/Kmlcad_C10CoA_mat+C10EnoylCoA_m/Kmlcad_C10EnoylCoA_mat+C12CoA_m /Kmlcad_C12CoA_mat+C12EnoylCoA_m/Kmlcad_C12EnoylCoA_mat+C14CoA_m /Kmlcad_C14CoA_mat+C14EnoylCoA_m/Kmlcad_C14EnoylCoA_mat+C16CoA_m

/Kmlcad_C16CoA_mat+C16EnoylCoA_m/Kmlcad_C16EnoylCoA_mat))*(1+(FADt_mat-FADH_m)/Kmlcad_FAD_mat+FADH_m/Kmlcad_FADH_mat));

VlcadC10=sf_lcadC10*V_lcad*((C10CoA_m *(FADt_mat-FADH_m))/(Kmlcad_C10CoA_mat* Kmlcad_FAD_mat)-(C10EnoylCoA_m*FADH_m)/(Kmlcad_C10CoA_mat*Kmlcad_FAD_mat *Keq_lcad))/((1+(C8CoA_m/Kmlcad_C8CoA_mat+C8EnoylCoA_m/Kmlcad_C8EnoylCoA_mat+ C10CoA_m/Kmlcad_C10CoA_mat+C10EnoylCoA_m/Kmlcad_C10EnoylCoA_mat+C12CoA_m /Kmlcad_C12CoA_mat+ C12EnoylCoA_m/Kmlcad_C12EnoylCoA_mat+C14CoA_m /Kmlcad_C14CoA_mat+C14EnoylCoA_m/Kmlcad_C14EnoylCoA_mat+C16CoA_m /Kmlcad_C16CoA_mat+C16EnoylCoA_m/Kmlcad_C16EnoylCoA_mat))*(1+(FADt_mat-FADH_m)/Kmlcad_FAD_mat+FADH_m/Kmlcad_FADH_mat));

VlcadC8=sf_lcadC8*V_lcad*((C8CoA_m *(FADt_mat-FADH_m))/(Kmlcad_C8CoA_mat *Kmlcad_FAD_mat)-(C8EnoylCoA_m*FADH_m)/(Kmlcad_C8CoA_mat*Kmlcad_FAD_mat* Keq_lcad))/((1+(C8CoA_m/Kmlcad_C8CoA_mat+C8EnoylCoA_m/Kmlcad_C8EnoylCoA_mat+ C10CoA_m/Kmlcad_C10CoA_mat+C10EnoylCoA_m/Kmlcad_C10EnoylCoA_mat+C12CoA_m/Kmlcad_C12CoA_mat+ C12EnoylCoA_m/Kmlcad_C12EnoylCoA_mat+C14CoA_m /Kmlcad_C14CoA_mat+C14EnoylCoA_m/Kmlcad_C14EnoylCoA_mat+C16CoA_m /Kmlcad_C16CoA_mat+C16EnoylCoA_m/Kmlcad_C16EnoylCoA_mat))*(1+(FADt_mat-FADH_m)/Kmlcad_FAD_mat+FADH_m/Kmlcad_FADH_mat));

VmcadC4=sf_mcadC4*V_mcad*((C4CoA_m *(FADt_mat-FADH_m))/(Kmmcad_C4CoA_mat *Kmmcad_FAD_mat)-(C4EnoylCoA_m*FADH_m)/(Kmmcad_C4CoA_mat*Kmmcad_FAD_mat* Keq_mcad))/((1+(C4CoA_m/Kmmcad_C4CoA_mat+C4EnoylCoA_m/Kmmcad_C4EnoylCoA_mat +C6CoA_m/Kmmcad_C6CoA_mat+C6EnoylCoA_m/Kmmcad_C6EnoylCoA_mat+C8CoA_m /Kmmcad_C8CoA_mat+ C8EnoylCoA_m/Kmmcad_C8EnoylCoA_mat+C10CoA_m /Kmmcad_C10CoA_mat+C10EnoylCoA_m/Kmmcad_C10EnoylCoA_mat+C12CoA_m /Kmmcad_C12CoA_mat+C12EnoylCoA_m/Kmmcad_C12EnoylCoA_mat))*(1+(FADt_mat-FADH_m)/Kmmcad_FAD_mat+FADH_m/Kmmcad_FADH_mat));

VmcadC6=sf_mcadC6*V_mcad*((C6CoA_m *(FADt_mat-FADH_m))/(Kmmcad_C6CoA_mat* Kmmcad_FAD_mat)-(C6EnoylCoA_m*FADH_m)/(Kmmcad_C6CoA_mat*Kmmcad_FAD_mat* Keq_mcad))/((1+(C4CoA_m/Kmmcad_C4CoA_mat+C4EnoylCoA_m/Kmmcad_C4EnoylCoA_mat +C6CoA_m/Kmmcad_C6CoA_mat+C6EnoylCoA_m/Kmmcad_C6EnoylCoA_mat+C8CoA_m /Kmmcad_C8CoA_mat+ C8EnoylCoA_m/Kmmcad_C8EnoylCoA_mat+C10CoA_m /Kmmcad_C10CoA_mat+C10EnoylCoA_m/Kmmcad_C10EnoylCoA_mat+C12CoA_m /Kmmcad_C12CoA_mat+C12EnoylCoA_m/Kmmcad_C12EnoylCoA_mat))*(1+(FADt_mat-FADH_m)/Kmmcad_FAD_mat+FADH_m/Kmmcad_FADH_mat));

VmcadC8=sf_mcadC8*V_mcad*((C8CoA_m *(FADt_mat-FADH_m))/(Kmmcad_C8CoA_mat* Kmmcad_FAD_mat)-(C8EnoylCoA_m*FADH_m)/(Kmmcad_C8CoA_mat*Kmmcad_FAD_mat* Keq_mcad))/((1+(C4CoA_m/Kmmcad_C4CoA_mat+C4EnoylCoA_m/Kmmcad_C4EnoylCoA_mat +C6CoA_m/Kmmcad_C6CoA_mat+C6EnoylCoA_m/Kmmcad_C6EnoylCoA_mat+C8CoA_m /Kmmcad_C8CoA_mat+ C8EnoylCoA_m/Kmmcad_C8EnoylCoA_mat+C10CoA_m /Kmmcad_C10CoA_mat+C10EnoylCoA_m/Kmmcad_C10EnoylCoA_mat+C12CoA_m /Kmmcad_C12CoA_mat+C12EnoylCoA_m/Kmmcad_C12EnoylCoA_mat))*(1+(FADt_mat-FADH_m)/Kmmcad_FAD_mat+FADH_m/Kmmcad_FADH_mat));

VmcadC10=sf_mcadC10*V_mcad*((C10CoA_m *(FADt_mat-FADH_m)) /(Kmmcad_C10CoA_mat*Kmmcad_FAD_mat)-(C10EnoylCoA_m*FADH_m) /(Kmmcad_C10CoA_mat*Kmmcad_FAD_mat*Keq_mcad))/((1+(C4CoA_m

/Kmmcad_C4CoA_mat+C4EnoylCoA_m/Kmmcad_C4EnoylCoA_mat+C6CoA_m /Kmmcad_C6CoA_mat+C6EnoylCoA_m/Kmmcad_C6EnoylCoA_mat+C8CoA_m /Kmmcad_C8CoA_mat+ C8EnoylCoA_m/Kmmcad_C8EnoylCoA_mat+C10CoA_m /Kmmcad_C10CoA_mat+C10EnoylCoA_m/Kmmcad_C10EnoylCoA_mat+C12CoA_m /Kmmcad_C12CoA_mat+C12EnoylCoA_m/Kmmcad_C12EnoylCoA_mat))*(1+(FADt_mat-FADH_m)/Kmmcad_FAD_mat+FADH_m/Kmmcad_FADH_mat));

VmcadC12=sf_mcadC12*V_mcad*((C12CoA_m *(FADt_mat-FADH_m)) /(Kmmcad_C12CoA_mat*Kmmcad_FAD_mat)-(C12EnoylCoA_m*FADH_m) /(Kmmcad_C12CoA_mat*Kmmcad_FAD_mat*Keq_mcad))/((1+(C4CoA_m/Kmmcad_C4CoA_mat +C4EnoylCoA_m/Kmmcad_C4EnoylCoA_mat+C6CoA_m/Kmmcad_C6CoA_mat+ C6EnoylCoA_m/Kmmcad_C6EnoylCoA_mat+C8CoA_m/Kmmcad_C8CoA_mat+ C8EnoylCoA_m/Kmmcad_C8EnoylCoA_mat+C10CoA_m/Kmmcad_C10CoA_mat+ C10EnoylCoA_m/Kmmcad_C10EnoylCoA_mat+C12CoA_m/Kmmcad_C12CoA_mat+ C12EnoylCoA_m/Kmmcad_C12EnoylCoA_mat))*(1+(FADt_mat-FADH_m)/Kmmcad_FAD_mat +FADH_m/Kmmcad_FADH_mat));

VscadC4=sf_scadC4*V_scad*((C4CoA_m *(FADt_mat-FADH_m))/(Kmscad_C4CoA_mat* Kmscad_FAD_mat)-(C4EnoylCoA_m*FADH_m)/(Kmscad_C4CoA_mat*Kmscad_FAD_mat* Keq_scad))/((1+(C4CoA_m/Kmscad_C4CoA_mat+C4EnoylCoA_m/Kmscad_C4EnoylCoA_mat+ C6CoA_m/Kmscad_C6CoA_mat+C6EnoylCoA_m/Kmscad_C6EnoylCoA_mat))*(1+(FADt_mat-FADH_m)/Kmscad_FAD_mat+FADH_m/Kmscad_FADH_mat));

VscadC6=sf_scadC6*V_scad*((C6CoA_m *(FADt_mat-FADH_m))/(Kmscad_C6CoA_mat *Kmscad_FAD_mat)-(C6EnoylCoA_m*FADH_m)/(Kmscad_C6CoA_mat*Kmscad_FAD_mat *Keq_scad))/((1+(C4CoA_m/Kmscad_C4CoA_mat+C4EnoylCoA_m/Kmscad_C4EnoylCoA_mat+ C6CoA_m/Kmscad_C6CoA_mat+C6EnoylCoA_m/Kmscad_C6EnoylCoA_mat))*(1+(FADt_mat-FADH_m)/Kmscad_FAD_mat+FADH_m/Kmscad_FADH_mat));

VcrotC4=sf_crotC4*V_crot*(C4EnoylCoA_m/Kmcrot_C4EnoylCoA_mat-C4OHCoA_m /(Kmcrot_C4OHCoA_mat*Keq_crot))/((1+(C4EnoylCoA_m/Kmcrot_C4EnoylCoA_mat+ C4OHCoA_m/Kmcrot_C4OHCoA_mat+C6EnoylCoA_m/Kmcrot_C6EnoylCoA_mat+ C6OHCoA_m/Kmcrot_C6OHCoA_mat+C8EnoylCoA_m/Kmcrot_C8EnoylCoA_mat+ C8OHCoA_m/Kmcrot_C8OHCoA_mat+C10EnoylCoA_m/Kmcrot_C10EnoylCoA_mat+ C10OHCoA_m/Kmcrot_C10OHCoA_mat+C12EnoylCoA_m/Kmcrot_C12EnoylCoA_mat+ C12OHCoA_m/Kmcrot_C12OHCoA_mat+C14EnoylCoA_m/Kmcrot_C14EnoylCoA_mat+ C14OHCoA_m/Kmcrot_C14OHCoA_mat+C16EnoylCoA_m/Kmcrot_C16EnoylCoA_mat+ C16OHCoA_m/Kmcrot_C16OHCoA_mat))+(AcacetylCoA_mat/Kicrot_AcacetylCoA_mat));

VcrotC6=sf_crotC6*V_crot*(C6EnoylCoA_m/Kmcrot_C6EnoylCoA_mat-C6OHCoA_m /(Kmcrot_C6OHCoA_mat*Keq_crot))/((1+(C4EnoylCoA_m/Kmcrot_C4EnoylCoA_mat+ C4OHCoA_m/Kmcrot_C4OHCoA_mat+C6EnoylCoA_m/Kmcrot_C6EnoylCoA_mat+ C6OHCoA_m/Kmcrot_C6OHCoA_mat+C8EnoylCoA_m/Kmcrot_C8EnoylCoA_mat+ C8OHCoA_m/Kmcrot_C8OHCoA_mat+C10EnoylCoA_m/Kmcrot_C10EnoylCoA_mat+ C10OHCoA_m/Kmcrot_C10OHCoA_mat+C12EnoylCoA_m/Kmcrot_C12EnoylCoA_mat+ C12OHCoA_m/Kmcrot_C12OHCoA_mat+C14EnoylCoA_m/Kmcrot_C14EnoylCoA_mat+ C14OHCoA_m/Kmcrot_C14OHCoA_mat+C16EnoylCoA_m/Kmcrot_C16EnoylCoA_mat+ C16OHCoA_m/Kmcrot_C16OHCoA_mat))+(AcacetylCoA_mat/Kicrot_AcacetylCoA_mat));

VcrotC8=sf_crotC8*V_crot*(C8EnoylCoA_m/Kmcrot_C8EnoylCoA_mat-C8OHCoA_m /(Kmcrot_C8OHCoA_mat*Keq_crot))/((1+(C4EnoylCoA_m/Kmcrot_C4EnoylCoA_mat+ C4OHCoA_m/Kmcrot_C4OHCoA_mat+C6EnoylCoA_m/Kmcrot_C6EnoylCoA_mat+ C6OHCoA_m/Kmcrot_C6OHCoA_mat+C8EnoylCoA_m/Kmcrot_C8EnoylCoA_mat+ C8OHCoA_m/Kmcrot_C8OHCoA_mat+C10EnoylCoA_m/Kmcrot_C10EnoylCoA_mat+ C10OHCoA_m/Kmcrot_C10OHCoA_mat+C12EnoylCoA_m/Kmcrot_C12EnoylCoA_mat+ C12OHCoA_m/Kmcrot_C12OHCoA_mat+C14EnoylCoA_m/Kmcrot_C14EnoylCoA_mat+ C14OHCoA_m/Kmcrot_C14OHCoA_mat+C16EnoylCoA_m/Kmcrot_C16EnoylCoA_mat+ C16OHCoA_m/Kmcrot_C16OHCoA_mat))+(AcacetylCoA_mat/Kicrot_AcacetylCoA_mat));

VcrotC10=sf_crotC10*V_crot*(C10EnoylCoA_m/Kmcrot_C10EnoylCoA_mat-C10OHCoA_m /(Kmcrot_C10OHCoA_mat*Keq_crot))/((1+(C4EnoylCoA_m/Kmcrot_C4EnoylCoA_mat+ C4OHCoA_m/Kmcrot_C4OHCoA_mat+C6EnoylCoA_m/Kmcrot_C6EnoylCoA_mat+ C6OHCoA_m/Kmcrot_C6OHCoA_mat+C8EnoylCoA_m/Kmcrot_C8EnoylCoA_mat+ C8OHCoA_m/Kmcrot_C8OHCoA_mat+C10EnoylCoA_m/Kmcrot_C10EnoylCoA_mat+ C10OHCoA_m/Kmcrot_C10OHCoA_mat+C12EnoylCoA_m/Kmcrot_C12EnoylCoA_mat+ C12OHCoA_m/Kmcrot_C12OHCoA_mat+C14EnoylCoA_m/Kmcrot_C14EnoylCoA_mat+ C14OHCoA_m/Kmcrot_C14OHCoA_mat+C16EnoylCoA_m/Kmcrot_C16EnoylCoA_mat+ C16OHCoA_m /Kmcrot_C16OHCoA_mat))+(AcacetylCoA_mat/Kicrot_AcacetylCoA_mat));

VcrotC12=sf_crotC12*V_crot*(C12EnoylCoA_m/Kmcrot_C12EnoylCoA_mat-C12OHCoA_m /(Kmcrot_C12OHCoA_mat*Keq_crot))/((1+(C4EnoylCoA_m/Kmcrot_C4EnoylCoA_mat+ C4OHCoA_m/Kmcrot_C4OHCoA_mat+C6EnoylCoA_m/Kmcrot_C6EnoylCoA_mat+ C6OHCoA_m/Kmcrot_C6OHCoA_mat+C8EnoylCoA_m/Kmcrot_C8EnoylCoA_mat+ C8OHCoA_m/Kmcrot_C8OHCoA_mat+C10EnoylCoA_m/Kmcrot_C10EnoylCoA_mat+ C10OHCoA_m/Kmcrot_C10OHCoA_mat+C12EnoylCoA_m/Kmcrot_C12EnoylCoA_mat+ C12OHCoA_m/Kmcrot_C12OHCoA_mat+C14EnoylCoA_m/Kmcrot_C14EnoylCoA_mat+ C14OHCoA_m/Kmcrot_C14OHCoA_mat+C16EnoylCoA_m/Kmcrot_C16EnoylCoA_mat+ C16OHCoA_m/Kmcrot_C16OHCoA_mat))+(AcacetylCoA_mat/Kicrot_AcacetylCoA_mat));

VcrotC14=sf_crotC14*V_crot*(C14EnoylCoA_m/Kmcrot_C14EnoylCoA_mat-C14OHCoA_m /(Kmcrot_C14OHCoA_mat*Keq_crot))/((1+(C4EnoylCoA_m/Kmcrot_C4EnoylCoA_mat+ C4OHCoA_m/Kmcrot_C4OHCoA_mat+C6EnoylCoA_m/Kmcrot_C6EnoylCoA_mat+C6OHCoA_ m/Kmcrot_C6OHCoA_mat+C8EnoylCoA_m/Kmcrot_C8EnoylCoA_mat+C8OHCoA_m /Kmcrot_C8OHCoA_mat+C10EnoylCoA_m/Kmcrot_C10EnoylCoA_mat+C10OHCoA_m /Kmcrot_C10OHCoA_mat+C12EnoylCoA_m/Kmcrot_C12EnoylCoA_mat+C12OHCoA_m /Kmcrot_C12OHCoA_mat+C14EnoylCoA_m/Kmcrot_C14EnoylCoA_mat+C14OHCoA_m /Kmcrot_C14OHCoA_mat+C16EnoylCoA_m/Kmcrot_C16EnoylCoA_mat+C16OHCoA_m /Kmcrot_C16OHCoA_mat))+(AcacetylCoA_mat/Kicrot_AcacetylCoA_mat));

VcrotC16=sf_crotC16*V_crot*(C16EnoylCoA_m/Kmcrot_C16EnoylCoA_mat-C16OHCoA_m /(Kmcrot_C16OHCoA_mat*Keq_crot))/((1+(C4EnoylCoA_m/Kmcrot_C4EnoylCoA_mat+C4OHCo A_m/Kmcrot_C4OHCoA_mat+C6EnoylCoA_m/Kmcrot_C6EnoylCoA_mat+C6OHCoA_m /Kmcrot_C6OHCoA_mat+C8EnoylCoA_m/Kmcrot_C8EnoylCoA_mat+C8OHCoA_m /Kmcrot_C8OHCoA_mat+C10EnoylCoA_m/Kmcrot_C10EnoylCoA_mat+C10OHCoA_m /Kmcrot_C10OHCoA_mat+C12EnoylCoA_m/Kmcrot_C12EnoylCoA_mat+C12OHCoA_m /Kmcrot_C12OHCoA_mat+C14EnoylCoA_m/Kmcrot_C14EnoylCoA_mat+C14OHCoA_m /Kmcrot_C14OHCoA_mat+C16EnoylCoA_m/Kmcrot_C16EnoylCoA_mat+C16OHCoA_m /Kmcrot_C16OHCoA_mat))+(AcacetylCoA_mat/Kicrot_AcacetylCoA_mat));

VmschadC4=sf_mschadC4*V_mschad*((C4OHCoA_m *(NADt_mat-NADHm)) /(Kmmschad_C4OHCoA_mat*Kmmschad_NAD_mat)-(C4KetoCoA_m*NADHm) /(Kmmschad_C4OHCoA_mat*Kmmschad_NAD_mat*Keq_mschad))/((1+(C4OHCoA_m /Kmmschad_C4OHCoA_mat+C4KetoCoA_m/Kmmschad_C4KetoCoA_mat+C6OHCoA_m /Kmmschad_C6OHCoA_mat+C6KetoCoA_m/Kmmschad_C6KetoCoA_mat+C8OHCoA_m /Kmmschad_C8OHCoA_mat+C8KetoCoA_m/Kmmschad_C8KetoCoA_mat+C10OHCoA_m /Kmmschad_C10OHCoA_mat+C10KetoCoA_m/Kmmschad_C10KetoCoA_mat+C12OHCoA_m /Kmmschad_C12OHCoA_mat+C12KetoCoA_m/Kmmschad_C12KetoCoA_mat+C14OHCoA_m /Kmmschad_C14OHCoA_mat+C14KetoCoA_m/Kmmschad_C14KetoCoA_mat+C16OHCoA_m /Kmmschad_C16OHCoA_mat+C16KetoCoA_m/Kmmschad_C16KetoCoA_mat))*(1+(NADt_mat-NADHm)/Kmmschad_NAD_mat+NADHm/Kmmschad_NADH_mat));

VmschadC6=sf_mschadC6*V_mschad*((C6OHCoA_m *(NADt_mat-NADHm)) /(Kmmschad_C6OHCoA_mat*Kmmschad_NAD_mat)-(C6KetoCoA_m*NADHm) /(Kmmschad_C6OHCoA_mat*Kmmschad_NAD_mat*Keq_mschad))/((1+(C4OHCoA_m /Kmmschad_C4OHCoA_mat+C4KetoCoA_m/Kmmschad_C4KetoCoA_mat+C6OHCoA_m /Kmmschad_C6OHCoA_mat+C6KetoCoA_m/Kmmschad_C6KetoCoA_mat+C8OHCoA_m /Kmmschad_C8OHCoA_mat+C8KetoCoA_m/Kmmschad_C8KetoCoA_mat+C10OHCoA_m /Kmmschad_C10OHCoA_mat+C10KetoCoA_m/Kmmschad_C10KetoCoA_mat+C12OHCoA_m /Kmmschad_C12OHCoA_mat+C12KetoCoA_m/Kmmschad_C12KetoCoA_mat+C14OHCoA_m /Kmmschad_C14OHCoA_mat+C14KetoCoA_m/Kmmschad_C14KetoCoA_mat+C16OHCoA_m /Kmmschad_C16OHCoA_mat+C16KetoCoA_m/Kmmschad_C16KetoCoA_mat))*(1+(NADt_mat-NADHm)/Kmmschad_NAD_mat+NADHm/Kmmschad_NADH_mat));

VmschadC8=sf_mschadC8*V_mschad*((C8OHCoA_m *(NADt_mat-NADHm)) /(Kmmschad_C8OHCoA_mat*Kmmschad_NAD_mat)-(C8KetoCoA_m*NADHm) /(Kmmschad_C8OHCoA_mat*Kmmschad_NAD_mat*Keq_mschad))/((1+(C4OHCoA_m /Kmmschad_C4OHCoA_mat+C4KetoCoA_m/Kmmschad_C4KetoCoA_mat+ C6OHCoA_m /Kmmschad_C6OHCoA_mat+C6KetoCoA_m/Kmmschad_C6KetoCoA_mat+C8OHCoA_m /Kmmschad_C8OHCoA_mat+C8KetoCoA_m/Kmmschad_C8KetoCoA_mat+C10OHCoA_m /Kmmschad_C10OHCoA_mat+C10KetoCoA_m/Kmmschad_C10KetoCoA_mat+C12OHCoA_m /Kmmschad_C12OHCoA_mat+C12KetoCoA_m/Kmmschad_C12KetoCoA_mat+C14OHCoA_m /Kmmschad_C14OHCoA_mat+C14KetoCoA_m/Kmmschad_C14KetoCoA_mat+C16OHCoA_m /Kmmschad_C16OHCoA_mat+C16KetoCoA_m/Kmmschad_C16KetoCoA_mat))*(1+(NADt_mat-NADHm)/Kmmschad_NAD_mat+NADHm/Kmmschad_NADH_mat));

VmschadC10=sf_mschadC10*V_mschad*((C10OHCoA_m *(NADt_mat-NADHm)) /(Kmmschad_C10OHCoA_mat*Kmmschad_NAD_mat)-(C10KetoCoA_m*NADHm) /(Kmmschad_C10OHCoA_mat*Kmmschad_NAD_mat*Keq_mschad))/((1+(C4OHCoA_m /Kmmschad_C4OHCoA_mat+C4KetoCoA_m/Kmmschad_C4KetoCoA_mat+C6OHCoA_m /Kmmschad_C6OHCoA_mat+C6KetoCoA_m/Kmmschad_C6KetoCoA_mat+C8OHCoA_m /Kmmschad_C8OHCoA_mat+C8KetoCoA_m/Kmmschad_C8KetoCoA_mat+C10OHCoA_m /Kmmschad_C10OHCoA_mat+C10KetoCoA_m/Kmmschad_C10KetoCoA_mat+C12OHCoA_m /Kmmschad_C12OHCoA_mat+C12KetoCoA_m/Kmmschad_C12KetoCoA_mat+C14OHCoA_m /Kmmschad_C14OHCoA_mat+C14KetoCoA_m/Kmmschad_C14KetoCoA_mat+C16OHCoA_m /Kmmschad_C16OHCoA_mat+C16KetoCoA_m/Kmmschad_C16KetoCoA_mat))*(1+(NADt_mat-NADHm)/Kmmschad_NAD_mat+NADHm/Kmmschad_NADH_mat));

VmschadC12=sf_mschadC12*V_mschad*((C12OHCoA_m *(NADt_mat-NADHm)) /(Kmmschad_C12OHCoA_mat*Kmmschad_NAD_mat)-(C12KetoCoA_m*NADHm) /(Kmmschad_C12OHCoA_mat*Kmmschad_NAD_mat*Keq_mschad))/((1+(C4OHCoA_m /Kmmschad_C4OHCoA_mat+C4KetoCoA_m/Kmmschad_C4KetoCoA_mat+C6OHCoA_m /Kmmschad_C6OHCoA_mat+C6KetoCoA_m/Kmmschad_C6KetoCoA_mat+C8OHCoA_m /Kmmschad_C8OHCoA_mat+C8KetoCoA_m/Kmmschad_C8KetoCoA_mat+C10OHCoA_m /Kmmschad_C10OHCoA_mat+C10KetoCoA_m/Kmmschad_C10KetoCoA_mat+C12OHCoA_m /Kmmschad_C12OHCoA_mat+C12KetoCoA_m/Kmmschad_C12KetoCoA_mat+C14OHCoA_m /Kmmschad_C14OHCoA_mat+C14KetoCoA_m/Kmmschad_C14KetoCoA_mat+C16OHCoA_m /Kmmschad_C16OHCoA_mat+C16KetoCoA_m/Kmmschad_C16KetoCoA_mat))*(1+(NADt_mat-NADHm)/Kmmschad_NAD_mat+NADHm/Kmmschad_NADH_mat));

VmschadC14=sf_mschadC14*V_mschad*((C14OHCoA_m *(NADt_mat-NADHm)) /(Kmmschad_C14OHCoA_mat*Kmmschad_NAD_mat)-(C14KetoCoA_m*NADHm) /(Kmmschad_C14OHCoA_mat*Kmmschad_NAD_mat*Keq_mschad))/((1+(C4OHCoA_m /Kmmschad_C4OHCoA_mat+C4KetoCoA_m/Kmmschad_C4KetoCoA_mat+C6OHCoA_m /Kmmschad_C6OHCoA_mat+C6KetoCoA_m/Kmmschad_C6KetoCoA_mat+C8OHCoA_m /Kmmschad_C8OHCoA_mat+C8KetoCoA_m/Kmmschad_C8KetoCoA_mat+C10OHCoA_m /Kmmschad_C10OHCoA_mat+C10KetoCoA_m/Kmmschad_C10KetoCoA_mat+C12OHCoA_m /Kmmschad_C12OHCoA_mat+C12KetoCoA_m/Kmmschad_C12KetoCoA_mat+C14OHCoA_m /Kmmschad_C14OHCoA_mat+C14KetoCoA_m/Kmmschad_C14KetoCoA_mat+C16OHCoA_m /Kmmschad_C16OHCoA_mat+C16KetoCoA_m/Kmmschad_C16KetoCoA_mat))*(1+(NADt_mat-NADHm)/Kmmschad_NAD_mat+NADHm/Kmmschad_NADH_mat));

VmschadC16=sf_mschadC16*V_mschad*((C16OHCoA_m *(NADt_mat-NADHm)) /(Kmmschad_C16OHCoA_mat*Kmmschad_NAD_mat)-(C16KetoCoA_m*NADHm) /(Kmmschad_C16OHCoA_mat*Kmmschad_NAD_mat*Keq_mschad))/((1+(C4OHCoA_m /Kmmschad_C4OHCoA_mat+C4KetoCoA_m/Kmmschad_C4KetoCoA_mat+C6OHCoA_m /Kmmschad_C6OHCoA_mat+C6KetoCoA_m/Kmmschad_C6KetoCoA_mat+C8OHCoA_m /Kmmschad_C8OHCoA_mat+C8KetoCoA_m/Kmmschad_C8KetoCoA_mat+C10OHCoA_m /Kmmschad_C10OHCoA_mat+C10KetoCoA_m/Kmmschad_C10KetoCoA_mat+C12OHCoA_m /Kmmschad_C12OHCoA_mat+C12KetoCoA_m/Kmmschad_C12KetoCoA_mat+C14OHCoA_m /Kmmschad_C14OHCoA_mat+C14KetoCoA_m/Kmmschad_C14KetoCoA_mat+C16OHCoA_m /Kmmschad_C16OHCoA_mat+C16KetoCoA_m/Kmmschad_C16KetoCoA_mat))*(1+(NADt_mat-NADHm)/Kmmschad_NAD_mat+NADHm/Kmmschad_NADH_mat));

VmckatC16= sf_mckatC16*V_mckat*((C16KetoCoA_m*CoA_mat)/(Kmmckat_C16KetoCoA_mat *Kmmckat_CoA_mat)-(C14CoA_m*AcCoA_m)/(Kmmckat_C16KetoCoA_mat* Kmmckat_CoA_mat*Keqmckat))/(1+((C16KetoCoA_m/Kmmckat_C16KetoCoA_mat+C16CoA_m /Kmmckat_C16CoA_mat+C14KetoCoA_m/Kmmckat_C14KetoCoA_mat+C14CoA_m /Kmmckat_C14CoA_mat+C12KetoCoA_m/Kmmckat_C12KetoCoA_mat+C12CoA_m /Kmmckat_C12CoA_mat+C10KetoCoA_m/Kmmckat_C10KetoCoA_mat+C10CoA_m /Kmmckat_C10CoA_mat+C8KetoCoA_m/Kmmckat_C8KetoCoA_mat+C8CoA_m /Kmmckat_C8CoA_mat+C6KetoCoA_m/Kmmckat_C6KetoCoA_mat+C6CoA_m /Kmmckat_C6CoA_mat+C4KetoCoA_m/Kmmckat_C4KetoCoA_mat+C4CoA_m /Kmmckat_C4CoA_mat)+AcCoA_m/Kmmckat_AcCoA_mat)*(1+CoA_mat/Kmmckat_CoA_mat +AcCoA_m/Kmmckat_AcCoA_mat));

VmckatC14= f_mckatC14*V_mckat*((C14KetoCoA_m*CoA_mat)/(Kmmckat_C14KetoCoA_mat
*Kmmckat_CoA_mat)-(C12CoA_m*AcCoA_m)/(Kmmckat_C14KetoCoA_mat*
Kmmckat_CoA_mat*Keqmckat))/(1+((C16KetoCoA_m/Kmmckat_C16KetoCoA_mat+C16CoA_m
/Kmmckat_C16CoA_mat+C14KetoCoA_m/Kmmckat_C14KetoCoA_mat+C14CoA_m
/Kmmckat_C14CoA_mat+C12KetoCoA_m/Kmmckat_C12KetoCoA_mat+C12CoA_m
/Kmmckat_C12CoA_mat+C10KetoCoA_m/Kmmckat_C10KetoCoA_mat+C10CoA_m
/Kmmckat_C10CoA_mat+C8KetoCoA_m/Kmmckat_C8KetoCoA_mat+C8CoA_m
/Kmmckat_C8CoA_mat+C6KetoCoA_m/Kmmckat_C6KetoCoA_mat+C6CoA_m
/Kmmckat_C6CoA_mat+C4KetoCoA_m/Kmmckat_C4KetoCoA_mat+C4CoA_m
/Kmmckat_C4CoA_mat)+AcCoA_m/Kmmckat_AcCoA_mat)*(1+CoA_mat/Kmmckat_CoA_mat
+AcCoA_m/Kmmckat_AcCoA_mat));

VmckatC12= sf_mckatC12*V_mckat*((C12KetoCoA_m*CoA_mat)
/(Kmmckat_C12KetoCoA_mat*Kmmckat_CoA_mat)-(C10CoA_m*AcCoA_m)
/(Kmmckat_C12KetoCoA_mat*Kmmckat_CoA_mat*Keqmckat))/(1+((C16KetoCoA_m
/Kmmckat_C16KetoCoA_mat+C16CoA_m/Kmmckat_C16CoA_mat+C14KetoCoA_m
/Kmmckat_C14KetoCoA_mat+C14CoA_m/Kmmckat_C14CoA_mat+C12KetoCoA_m
/Kmmckat_C12KetoCoA_mat+C12CoA_m/Kmmckat_C12CoA_mat+C10KetoCoA_m
/Kmmckat_C10KetoCoA_mat+C10CoA_m/Kmmckat_C10CoA_mat+C8KetoCoA_m
/Kmmckat_C8KetoCoA_mat+C8CoA_m/Kmmckat_C8CoA_mat+C6KetoCoA_m
/Kmmckat_C6KetoCoA_mat+C6CoA_m/Kmmckat_C6CoA_mat+C4KetoCoA_m
/Kmmckat_C4KetoCoA_mat+C4CoA_m/Kmmckat_C4CoA_mat)+AcCoA_m
/Kmmckat_AcCoA_mat)*(1+CoA_mat/Kmmckat_CoA_mat+AcCoA_m/Kmmckat_AcCoA_mat));

VmckatC10= sf_mckatC10*V_mckat*((C10KetoCoA_m*CoA_mat)
/(Kmmckat_C10KetoCoA_mat*Kmmckat_CoA_mat)-(C8CoA_m*AcCoA_m)
/(Kmmckat_C10KetoCoA_mat*Kmmckat_CoA_mat*Keqmckat))/(1+((C16KetoCoA_m
/Kmmckat_C16KetoCoA_mat+C16CoA_m/Kmmckat_C16CoA_mat+C14KetoCoA_m
/Kmmckat_C14KetoCoA_mat+C14CoA_m/Kmmckat_C14CoA_mat+C12KetoCoA_m
/Kmmckat_C12KetoCoA_mat+C12CoA_m/Kmmckat_C12CoA_mat+C10KetoCoA_m
/Kmmckat_C10KetoCoA_mat+C10CoA_m/Kmmckat_C10CoA_mat+C8KetoCoA_m
/Kmmckat_C8KetoCoA_mat+C8CoA_m/Kmmckat_C8CoA_mat+C6KetoCoA_m
/Kmmckat_C6KetoCoA_mat+C6CoA_m/Kmmckat_C6CoA_mat+C4KetoCoA_m
/Kmmckat_C4KetoCoA_mat+C4CoA_m/Kmmckat_C4CoA_mat)+AcCoA_m
/Kmmckat_AcCoA_mat)*(1+CoA_mat/Kmmckat_CoA_mat+AcCoA_m/Kmmckat_AcCoA_mat));

VmckatC8= sf_mckatC8*V_mckat*((C8KetoCoA_m*CoA_mat)
/(Kmmckat_C8KetoCoA_mat*Kmmckat_CoA_mat)-(C6CoA_m*AcCoA_m)
/(Kmmckat_C8KetoCoA_mat*Kmmckat_CoA_mat*Keqmckat))/(1+((C16KetoCoA_m
/Kmmckat_C16KetoCoA_mat+C16CoA_m/Kmmckat_C16CoA_mat+C14KetoCoA_m
/Kmmckat_C14KetoCoA_mat+C14CoA_m/Kmmckat_C14CoA_mat+C12KetoCoA_m
/Kmmckat_C12KetoCoA_mat+C12CoA_m/Kmmckat_C12CoA_mat+C10KetoCoA_m
/Kmmckat_C10KetoCoA_mat+C10CoA_m/Kmmckat_C10CoA_mat+C8KetoCoA_m
/Kmmckat_C8KetoCoA_mat+C8CoA_m/Kmmckat_C8CoA_mat+C6KetoCoA_m
/Kmmckat_C6KetoCoA_mat+C6CoA_m/Kmmckat_C6CoA_mat+C4KetoCoA_m
/Kmmckat_C4KetoCoA_mat+C4CoA_m/Kmmckat_C4CoA_mat)+AcCoA_m
/Kmmckat_AcCoA_mat)*(1+CoA_mat/Kmmckat_CoA_mat+AcCoA_m/Kmmckat_AcCoA_mat));

VmckatC6= sf_mckatC6*V_mckat*((C6KetoCoA_m*CoA_mat)
/(Kmmckat_C6KetoCoA_mat*Kmmckat_CoA_mat)-(C4CoA_m*AcCoA_m)
/(Kmmckat_C6KetoCoA_mat*Kmmckat_CoA_mat*Keqmckat))/(1+((C16KetoCoA_m
/Kmmckat_C16KetoCoA_mat+C16CoA_m/Kmmckat_C16CoA_mat+C14KetoCoA_m
/Kmmckat_C14KetoCoA_mat+C14CoA_m/Kmmckat_C14CoA_mat+C12KetoCoA_m
/Kmmckat_C12KetoCoA_mat+C12CoA_m/Kmmckat_C12CoA_mat+C10KetoCoA_m
/Kmmckat_C10KetoCoA_mat+C10CoA_m/Kmmckat_C10CoA_mat+C8KetoCoA_m
/Kmmckat_C8KetoCoA_mat+C8CoA_m/Kmmckat_C8CoA_mat+C6KetoCoA_m
/Kmmckat_C6KetoCoA_mat+C6CoA_m/Kmmckat_C6CoA_mat+C4KetoCoA_m
/Kmmckat_C4KetoCoA_mat+C4CoA_m/Kmmckat_C4CoA_mat)+AcCoA_m
/Kmmckat_AcCoA_mat)*(1+CoA_mat/Kmmckat_CoA_mat+AcCoA_m/Kmmckat_AcCoA_mat));

VmckatC4= sf_mckatC4*V_mckat*((C4KetoCoA_m*CoA_mat)
/(Kmmckat_C4KetoCoA_mat*Kmmckat_CoA_mat)-(AcCoA_m*AcCoA_m)
/(Kmmckat_C4KetoCoA_mat*Kmmckat_CoA_mat*Keqmckat))/(1+((C16KetoCoA_m
/Kmmckat_C16KetoCoA_mat+C16CoA_m/Kmmckat_C16CoA_mat+C14KetoCoA_m
/Kmmckat_C14KetoCoA_mat+C14CoA_m/Kmmckat_C14CoA_mat+C12KetoCoA_m
/Kmmckat_C12KetoCoA_mat+C12CoA_m/Kmmckat_C12CoA_mat+C10KetoCoA_m
/Kmmckat_C10KetoCoA_mat+C10CoA_m/Kmmckat_C10CoA_mat+C8KetoCoA_m
/Kmmckat_C8KetoCoA_mat+C8CoA_m/Kmmckat_C8CoA_mat+C6KetoCoA_m
/Kmmckat_C6KetoCoA_mat+C6CoA_m/Kmmckat_C6CoA_mat+C4KetoCoA_m
/Kmmckat_C4KetoCoA_mat+C4CoA_m/Kmmckat_C4CoA_mat)+AcCoA_m
/Kmmckat_AcCoA_mat)*(1+CoA_mat/Kmmckat_CoA_mat+AcCoA_m/Kmmckat_AcCoA_mat));

%MTP

VmtpC16=sfmtpC16*V_mtp*((C16EnoylCoA_m*(NADt_mat-NADHm)*CoA_mat)
/(Kmmtp_C16EnoylCoA_mat*Kmmtp_NAD_mat*Kmmtp_CoA_mat)-C14CoA_m*NADHm*
AcCoA_m/(Kmmtp_C16EnoylCoA_mat*Kmmtp_NAD_mat*Kmmtp_CoA_mat*Keqmtp))/((1+
(C16EnoylCoA_m/Kmmtp_C16EnoylCoA_mat+C16CoA_m/Kmmtp_C16CoA_mat+
C14EnoylCoA_m/Kmmtp_C14EnoylCoA_mat+C14CoA_m/Kmmtp_C14CoA_mat+
C12EnoylCoA_m/Kmmtp_C12EnoylCoA_mat+C12CoA_m/Kmmtp_C12CoA_mat+
C10EnoylCoA_m/Kmmtp_C10EnoylCoA_mat+C10CoA_m/Kmmtp_C10CoA_mat +
C8EnoylCoA_m/Kmmtp_C8EnoylCoA_mat+C8CoA_m/Kmmtp_C8CoA_mat)+C6CoA_m
/Kmmtp_C6CoA_mat+AcacetylCoA_mat/Kimtp_AcetoacetylCoA_mat)*(1+(NADt_mat-
NADHm)/Kmmtp_NAD_mat+NADHm/Kmmtp_NADH_mat)*(1+CoA_mat/Kmmtp_CoA_mat+
AcCoA_m/Kmmtp_AcCoA_mat));

VmtpC14=sfmtpC14*V_mtp*((C14EnoylCoA_m*(NADt_mat-NADHm)*CoA_mat)
/(Kmmtp_C14EnoylCoA_mat*Kmmtp_NAD_mat*Kmmtp_CoA_mat)-C12CoA_m*NADHm*
AcCoA_m/(Kmmtp_C14EnoylCoA_mat*Kmmtp_NAD_mat*Kmmtp_CoA_mat*Keqmtp))/((1+
(C16EnoylCoA_m/Kmmtp_C16EnoylCoA_mat+C16CoA_m/Kmmtp_C16CoA_mat+
C14EnoylCoA_m/Kmmtp_C14EnoylCoA_mat+C14CoA_m/Kmmtp_C14CoA_mat+
C12EnoylCoA_m/Kmmtp_C12EnoylCoA_mat+C12CoA_m/Kmmtp_C12CoA_mat+
C10EnoylCoA_m/Kmmtp_C10EnoylCoA_mat+C10CoA_m/Kmmtp_C10CoA_mat +
C8EnoylCoA_m/Kmmtp_C8EnoylCoA_mat+C8CoA_m/Kmmtp_C8CoA_mat) + C6CoA_m
/Kmmtp_C6CoA_mat+AcacetylCoA_mat/Kimtp_AcetoacetylCoA_mat)*(1+NADt_mat-
NADHm/Kmmtp_NAD_mat+NADHm/Kmmtp_NADH_mat)*(1+CoA_mat/Kmmtp_CoA_mat+
AcCoA_m/Kmmtp_AcCoA_mat));

```
VmtpC12=sfmtpC12*V_mtp*((C12EnoylCoA_m*(NADt_mat-
NADHm)*CoA_mat)/(Kmmtp_C12EnoylCoA_mat*Kmmtp_NAD_mat*Kmmtp_CoA_mat)-
C10CoA_m*NADHm*AcCoA_m/(Kmmtp_C12EnoylCoA_mat*Kmmtp_NAD_mat*
Kmmtp_CoA_mat*Keqmtp))/((1+(C16EnoylCoA_m/Kmmtp_C16EnoylCoA_mat+C16CoA_m
/Kmmtp_C16CoA_mat+C14EnoylCoA_m/Kmmtp_C14EnoylCoA_mat+C14CoA_m
/Kmmtp_C14CoA_mat+C12EnoylCoA_m/Kmmtp_C12EnoylCoA_mat+C12CoA_m
/Kmmtp_C12CoA_mat+C10EnoylCoA_m/Kmmtp_C10EnoylCoA_mat+C10CoA_m
/Kmmtp_C10CoA_mat +C8EnoylCoA_m/Kmmtp_C8EnoylCoA_mat+C8CoA_m
/Kmmtp_C8CoA_mat) +C6CoA_m/Kmmtp_C6CoA_mat+AcacetylCoA_mat
/Kimtp_AcetoacetylCoA_mat)*(1+NADt_mat-NADHm/Kmmtp_NAD_mat+NADHm
/Kmmtp_NADH_mat)*(1+CoA_mat/Kmmtp_CoA_mat+AcCoA_m/Kmmtp_AcCoA_mat) );

VmtpC10=sfmtpC10*V_mtp*((C10EnoylCoA_m*(NADt_mat-NADHm)*CoA_mat)
/(Kmmtp_C10EnoylCoA_mat*Kmmtp_NAD_mat*Kmmtp_CoA_mat)-C8CoA_m*NADHm*
AcCoA_m/(Kmmtp_C10EnoylCoA_mat*Kmmtp_NAD_mat*Kmmtp_CoA_mat*Keqmtp))/((1+
(C16EnoylCoA_m/Kmmtp_C16EnoylCoA_mat+C16CoA_m/Kmmtp_C16CoA_mat+
C14EnoylCoA_m/Kmmtp_C14EnoylCoA_mat+C14CoA_m/Kmmtp_C14CoA_mat+
C12EnoylCoA_m/Kmmtp_C12EnoylCoA_mat+C12CoA_m/Kmmtp_C12CoA_mat+
C10EnoylCoA_m/Kmmtp_C10EnoylCoA_mat+C10CoA_m/Kmmtp_C10CoA_mat +
C8EnoylCoA_m/Kmmtp_C8EnoylCoA_mat+C8CoA_m/Kmmtp_C8CoA_mat) +C6CoA_m
/Kmmtp_C6CoA_mat+AcacetylCoA_mat/Kimtp_AcetoacetylCoA_mat)*(1+NADt_mat-
NADHm/Kmmtp_NAD_mat+NADHm/Kmmtp_NADH_mat)*(1+CoA_mat/Kmmtp_CoA_mat+
AcCoA_m/Kmmtp_AcCoA_mat) );

VmtpC8=sfmtpC8*V_mtp*((C8EnoylCoA_m*(NADt_mat-NADHm)*CoA_mat)
/(Kmmtp_C8EnoylCoA_mat*Kmmtp_NAD_mat*Kmmtp_CoA_mat)-C6CoA_m*NADHm*
AcCoA_m/(Kmmtp_C8EnoylCoA_mat*Kmmtp_NAD_mat*Kmmtp_CoA_mat*Keqmtp))/((1+
(C16EnoylCoA_m/Kmmtp_C16EnoylCoA_mat+C16CoA_m/Kmmtp_C16CoA_mat+
C14EnoylCoA_m/Kmmtp_C14EnoylCoA_mat+C14CoA_m/Kmmtp_C14CoA_mat+
C12EnoylCoA_m/Kmmtp_C12EnoylCoA_mat+C12CoA_m/Kmmtp_C12CoA_mat+
C10EnoylCoA_m/Kmmtp_C10EnoylCoA_mat+C10CoA_m/Kmmtp_C10CoA_mat
+C8EnoylCoA_m/Kmmtp_C8EnoylCoA_mat+C8CoA_m/Kmmtp_C8CoA_mat)+C6CoA_m
/Kmmtp_C6CoA_mat+AcacetylCoA_mat/Kimtp_AcetoacetylCoA_mat)*(1+NADt_mat-
NADHm/Kmmtp_NAD_mat+NADHm/Kmmtp_NADH_mat)*(1+CoA_mat/Kmmtp_CoA_mat+
AcCoA_m/Kmmtp_AcCoA_mat) );

%% Ordinary differential equations
dydt=[fCam*(Vuni-VnaCa);                  %Cam (1)
VANT+PyrCb-VATPase-VSL;                   %ADPm (2)
-(-VHNe-VHSDH-VETFH+Vhu+VANT+Vhleak+VnaCa+2*Vuni+VIMAC)/Cimat;% Dpsim (3)
-VNO+VIDH+VKGDH+VMDHm-VTHD+PyrDH+(NADHDemV+VmtpC16+VmschadC16
+VmtpC14+VmschadC14+VmtpC12+VmschadC12+VmtpC10+VmschadC10+VmtpC8
+VmschadC8+VmschadC6+VmschadC4)*Vlcyt/Vlmat;              %NADHm (4)
beta_matr*(JH);                 %Hm     (5)
-VATPase+VPiC-VSL+PyrCb;                  %Pim    (6)
% VACO-VIDH;                    %ISO    (7)
VACO-VIDH-VIDH_NADP;                      %ISO    (7)
VIDH+VAATm-VKGDH+VIDH_NADP;               %aKG    (8)
```

VKGDH-VSL; %SCoA (9)
VSL-VO2SDH; %Succ (10)
VO2SDH-VFH; %FUM (11)
VFH-VMDHm; %MAL (12)
% VFH-VMDHm+VOGC*Vlcyt/Vlmat; %MAL (12)
VMDHm-VCS-VAATm+PyrCb; %Oaa (13)
(Vcpt1C16-VcactC16); %C16AcylCarn_cy (14)
(VcactC16-Vcpt2C16)*Vlcyt/Vlmat; %C16Carn_mat (15)
(Vcpt2C16-VvlcadC16-VlcadC16)*Vlcyt/Vlmat; %C16CoA_mat (16)
(VvlcadC16+VlcadC16-VcrotC16-VmtpC16)*Vlcyt/Vlmat; %C16EnoylCoA_mat (17)
(VcrotC16-VmschadC16)*Vlcyt/Vlmat; %C16OHCoA_mat (18)
(VmschadC16-VmckatC16)*Vlcyt/Vlmat; %C16KetoCoA_mat (19)
(VmtpC16+VmckatC16-VvlcadC14-VlcadC14)*Vlcyt/Vlmat; %C14CoA_mat (20)
(VvlcadC14+VlcadC14-VcrotC14-VmtpC14)*Vlcyt/Vlmat; %C14EnoylCoA_mat (21)
(VcrotC14-VmschadC14)*Vlcyt/Vlmat; %C14OHCoA_mat (22)
(VmschadC14-VmckatC14)*Vlcyt/Vlmat; %C14KetoCoA_mat (23)
(VmtpC14+VmckatC14-VvlcadC12-VlcadC12-VmcadC12)*Vlcyt/Vlmat; %C12CoA_mat (24)
(VvlcadC12+VlcadC12+VmcadC12-VcrotC12-VmtpC12)*Vlcyt/Vlmat; %C12EnoylCoA_mat (25)
(VcrotC12-VmschadC12)*Vlcyt/Vlmat; %C12OHCoA_mat (26)
(VmschadC12-VmckatC12)*Vlcyt/Vlmat; %C12KetoCoA_mat (27)
(VmtpC12+VmckatC12-VmcadC10-VlcadC10)*Vlcyt/Vlmat; %C10CoA_mat (28)
(VmcadC10+VlcadC10-VcrotC10-VmtpC10)*Vlcyt/Vlmat; %C10EnoylCoA_mat (29)
(VcrotC10-VmschadC10)*Vlcyt/Vlmat; %C10OHCoA_mat (30)
(VmschadC10-VmckatC10)*Vlcyt/Vlmat; %C10KetoCoA_mat (31)
(VmtpC10+VmckatC10-VmcadC8-VlcadC8)*Vlcyt/Vlmat; %C8CoA_mat (32)
(VmcadC8+VlcadC8-VcrotC8-VmtpC8)*Vlcyt/Vlmat; %C8EnoylCoA_mat (33)
(VcrotC8-VmschadC8)*Vlcyt/Vlmat; %C8OHCoA_mat (34)
(VmschadC8-VmckatC8)*Vlcyt/Vlmat; %C8KetoCoA_mat (35)
(VmtpC8+VmckatC8-VmcadC6-VscadC6)*Vlcyt/Vlmat; %C6CoA_mat (36)
(VmcadC6+VscadC6-VcrotC6)*Vlcyt/Vlmat; %C6EnoylCoA_mat (37)
(VcrotC6-VmschadC6)*Vlcyt/Vlmat; %C6OHCoA_mat (38)
(VmschadC6-VmckatC6)*Vlcyt/Vlmat; %C6KetoCoA_mat (39)
(VmckatC6-VmcadC4-VscadC4)*Vlcyt/Vlmat; %C4CoA_mat (40)
(VmcadC4+VscadC4-VcrotC4)*Vlcyt/Vlmat; %C4EnoylCoA_mat (41)
(VcrotC4-VmschadC4)*Vlcyt/Vlmat; %C4OHCoA_mat (42)
(VmschadC4-VmckatC4)*Vlcyt/Vlmat; %C4KetoCoA_mat (43)
(VmtpC16+VmckatC16+VmtpC14+VmckatC14+VmtpC12+VmckatC12+VmtpC10+VmckatC10+VmtpC8+VmckatC8+VmckatC6+2*VmckatC4)*Vlcyt/Vlmat+PyrDH-VCS;% AcCoA_mat (44)
(VvlcadC16+VlcadC16+VvlcadC14+VlcadC14+VvlcadC12+VlcadC12+VmcadC12+VlcadC10+VmcadC10+VlcadC8+VmcadC8+VmcadC6+VscadC6+VmcadC4+VscadC4)*Vlcyt/Vlmat-VO2ETF; % FADH_m_mat (45)
VIDH_NADP+VTHD-VGRm-VTxRm; %NADPH (46)
shunt*(VNO+VO2SDH+VO2ETF)-VMnSOD-VtrROS;%SO2m (47)
Vlmat/Vlcyt*VtrROS-VCuZnSOD; %SO2i (48)
VMnSOD-VdifH2O2-VGPXm-VTxPXm; %H2O2m (49)
VCuZnSOD+Vlmat/Vlcyt*VdifH2O2-VGPX-VTxPX-Vcat; %H2O2i (50)
VGRm-VGPXm-VGRXm+VGST-VPSSGm; %GSHm (51)

```
VGR-VGPX-VGRX-Vlmat/Vlcyt*VGST-VPSSGi;  %GSHi   (52)
5.0D-1*(VGPXm-VGRm)+VGRXm;              %GSSGm  (53)
VTxRm-VTxPXm;                           %TrxSH2m  (54)
VTxR-VTxPX;                             %TrxSH2    (55)
5.0D-1*(VGPX-VGR)+VGRX+0.5*Vlmat/Vlcyt*VGST;     %GSSGi     (56)
VPSSGm-VGRXm;                           %PSSGm     (57)
VPSSGi-VGRX;                            %PSSGi    (58)
GLUT4V-HKV-ALR1V;                       %Glc            (59)
HKV-PFKV-G6PDHV+RTAlV+RTAc2V+VPGLM;     %G6P+F6P          (60)
PFKV-ALDV;                              %F16BP           (61)
2*ALDV-GAPDHV+RTAcV-RTAlV+RTAc2V;       %G3P             (62)
GAPDHV-PGKV;                            %G13BP           (63)
PGKV-ENOLV;                             %2PG + 3PG (name GP3) (64)
ENOLV-PKV;                              %PEP             (65)
PKV-LDHV-PyrCV;                         %Pyr              (66)
ALR1V-SoDHV;                            %Sorbitol         (67)
SoDHV-FRUTV;                            %Fructose         (68)
G6PDHV-P6GDHV;                          %GO6P (69)
P6GDHV-R5PIV-Ru5PEV;                    %Ru5P (Ribulose 5P) (70)
R5PIV-RTAcV;                            %R5P (Ribose5P)     (71)
Ru5PEV-RTAcV-RTAc2V-XyDHV;              %X5P  (Xylulose5P)  (72)
RTAcV-RTAlV;                            %S7P (Sedoheptulose 7P) (73)
RTAlV-RTAc2V;                           %E4P  (Erythrose 4P) (74)
XyDHV-XiOHTV;                           %Xilitol           (75)
-HKV-PFKV+PGKV+PKV-HYdroATPV+VANT*Vlmat/Vlcyt;      %ATP                (76)
+GAPDHV-LDHV+SoDHV-XyDHV-NADHDemV;      %NADH cyto         (77)
+G6PDHV+P6GDHV-ALR1V-NADPHDemV;         %NADPH cyto         (78)
+VAmil-VGPa-VGPb;                       %Manosides          (79)
+VGPa+VGPb-VPGLM;                       % G1P               (80)
+PyrCV*Vlcyt/Vlmat-PyrDH-PyrCb;         %Pyr in mito        (81)
VCS-VACO-VCiC;];                        % CIT_m             (82)
return
```

Running file "Simulations_WholeCatab_v3.m"

```
y0=[0.000260;0.6974197;178.9;0.1063;3.5077e-05;0.02368;7.89030e-05;0.00305589;
0.0173849568;5.3303e-06;0.019637;0.00026358;1.26834e-06;0.4125;12.890;0.060346;0.00020047;
3.5266e-05;0.00524036;0.01876866;6.2341e-05;6.2460e-06;0.000922;0.0032564;1.0818e-05;
5.7067e-06;0.000834437;0.00297124;9.87053e-06;7.5012e-06;0.0010892;0.00388849;1.29179e-05;
1.845966e-05;0.0026756;0.009542865;3.1702e-05;0.00017573;0.0261349;0.093912;0.000311989;
0.001096287;0.16306;0.58598;0.699612;0.026291;3.06032e-07;1.0431e-10;7.95128e-07;9.5004e-10
;0.310514;0.310514;0.7635947;0.024757;0.0499999;0.002079;0.000561024;1.7666759e-06;
0.4219823;0.731687;0.016394;0.0010467;0.0015676;0.1080516;0.0002864;0.0085887;0.170537;
0.0080778;2.0797e-06;0.026169;0.020051;0.0477454;0.3933296;0.02151581;2.04476e-06; 5.06934;
0.05237;0.03846;0.0028666;0.18998;0.0087969;0.0009419];
options=odeset('RelTol',1e-12,'AbsTol',1e-16);
ode=@(t,kmrgd) WholeCatab_v3(t,kmrgd); %
```

```
tspan= [0; 5.0E12];
sol=ode15s(ode,tspan,y0,options); %
```
